# A systematic review of the effects of nanoplastics on fish

**DOI:** 10.3389/ftox.2025.1530209

**Published:** 2025-05-30

**Authors:** Asok K. Dasmahapatra, Joydeep Chatterjee, Paul B. Tchounwou

**Affiliations:** ^1^ Department of BioMolecular Science, Environmental Toxicology Division, University of Mississippi, Oxford, MS, United States; ^2^ Department of Biology, University of Texas-Arlington, Arlington, TX, United States; ^3^ RCMI Center for Urban Health Disparities Research and Innovation, School of Computer, Mathematical and Natural Sciences, Morgan State University, Baltimore, MD, United States

**Keywords:** nanoplastics, fish, oxidative stress, genotoxic effects, development, intergenerational effects

## Abstract

The global concern about plastics has been amplified due to their widespread contamination in the environment and their ability to cross biological barriers in living organisms. However, our understanding of their bioaccumulation, toxicity, and interaction with other environmental pollutants remains limited. Plastics are classified into three categories: macro-(MAP > 5 mm), micro-(MIP, <5 mm), and nanoplastics (NAP≤ 100 nm). Among these, NAPs have superior sorption capacity, a large surface area, and a greater ability to release co-contaminants into tissues, resulting in more complex and harmful effects compared to MAPs and MIPs. To assess the toxic effects of NAPs, particularly their genotoxicity in fish, we carried out a bibliographic search in PubMed using the search terms “nanoplastics” and “fish,” which yielded 233 articles. These studies focused on various polymers including polyamide (PA), polycarbonate (PC), polyethylene (PE), polyethylene terephthalate (PET), polymethylmethacrylate (PMMA), polypropylene (PPP), polystyrene (PS), and polyvinyl chloride (PVC). We further refined our search by including fish species such as common carp, fathead minnows, medaka, tilapia, trout, and zebrafish and selected 114 articles for review. This article provides a comprehensive overview of the current state of knowledge on the effects of NAPs on fishes, emphasizing their interaction with co-contaminants including metals, polycyclic aromatic hydrocarbons, pharmaceuticals, pesticides, antibiotics, plastic additives, and endocrine disruptors found in the aquatic environments. Our findings indicate that among fish species, zebrafish (∼68%) is the most frequently studied, while PS (∼89%) is the most commonly encountered NAP in the aquatic ecosystems. Despite substantial experimental variability, our systematic review highlights that NAPs accumulate in various tissues of fish including the skin, muscle, gill, gut, liver, heart, gonads, and brain across all developmental stages, from embryos to adults. NAP exposure leads to significant adverse effects including increased oxidative stress, decreased locomotor and foraging activities, altered growth, immunity, lipid metabolism, and induced neurotoxicity. Furthermore, NAP exposure modulates estrogen–androgen–thyroid–steroidogenesis (EATS) pathways and shows potential intergenerational effects. Although the USEPA and EU are aware of the global impacts of plastic pollution, the prolonged persistence of plastics continues to pose a significant risk to both aquatic life and human health.

## 1 Introduction

Plastic particles are introduced into the environment through industrial activities, human practices, and inadequate waste management systems (Chen et al., 2017a; Gigault et al., 2018; Cox et al., 2019; Ebere et al., 2019; Strungaru et al., 2019; Kokalj et al., 2021). In recent decades, plastic pollution has emerged as the second largest environmental challenge, ranking among global threats such as ocean acidification, climate change, and ozone depletion (Amaral-Zettler et al., 2015; Ma et al., 2016; Vethaak and Leslie, 2016; Schymanski et al., 2018; Alimba and Faggio, 2019). The predominant source of plastic pollution stems from poor waste management practices including garbage dumping, improper disposal of waste, and runoff from industrial or agricultural activities (Leslie et al., 2017; Mahon et al., 2017; Triebskorn et al., 2019). The onset of the COVID-19 pandemic further exacerbated plastic contamination with the widespread use of personal protective equipment (e.g., face masks) and single-use packaging materials, contributing to a significant rise in plastic waste (Aragaw, 2020; Fadare and Okoffo, 2020; Yudell et al., 2020; Patricio Silva et al., 2021; Vanapalli et al., 2021; Afrin et al., 2022; Cho et al., 2022). Plastic waste once released into the environment does not decompose rapidly. Instead, it undergoes gradual decomposition, involving photolysis, oxidation, abrasion, hydrolysis, and biodegradation over an extended period of time (Sudhakar et al., 2007; Watters et al., 2010; Andrady, 2011; Maity and Pramanick, 2020). Larger plastic particles eventually break down into microplastics (MIPs; diameter ranging between 100 and 50,00,000 nm) and nanoplastics (NAPs, diameter ≤100 nm) through mechanisms such as wave action, mechanical wear and tear, photooxidation, and microbial degradation (O’Brine and Thompson, 2010; Lambert et al., 2013; Cozar et al., 2014; Gigault et al., 2016; Lambert and Wagner, 2016). NAPs are potentially more hazardous than MIPs (Rochman et al., 2013; Almeida et al., 2019; Domenech et al., 2020; Liang et al., 2021; Yang and Wang, 2022; Yang and Wang, 2023; Huang et al., 2023; Huang et al., 2023). The European Food Safety Authority (EFSA) has indicated that particles less than 150 µm (150,000 nm) in diameter may cross the intestinal mucosal barrier, while particles less than 1.5 µm (1,500 nm) in diameter can be transported into deeper tissues, including vital organs. Several types of MIPs (<50,00,000 nm), including polystyrene (PS), polyvinyl chloride (PVC), polyethylene (PE), polyethylene terephthalate (PET), polymethyl methacrylate (PMMA), polyoxymethylene, and polypropylene (PPP), have been found in various environmental compartments (de Sa et al., 2018) and have also been detected in the liver tissue of individuals with liver cirrhosis (Horvatits et al., 2022).

NAPs, often used as raw materials in products such as facial cleaners, scrubs, toothpaste, and other personal care items, are unintentional byproducts of plastic degradation and manufacturing processes (Enfrin et al., 2020; Kim, 2021; Kim et al., 2021). These particles, typically less than 1,000 nm in size, exhibit colloidal behavior and possess distinct chemical and physical characteristics compared to bulk plastics (Sharifi et al., 2012; Chen et al., 2017b; Pitt et al., 2018a; Lee et al., 2019). Due to their small size and high surface area, NAPs are highly efficient at both physical and chemical absorption of other environmental contaminants (Hartmann et al., 2017; Lee et al., 2019; Trevisan et al., 2019; Bhagat et al., 2020; Bhagat et al., 2021). Moreover, they are easily transferred through the food chain (Chae et al., 2018). Once absorbed into the body, NAPs can spread into the organs, including the brain and gonads, by overcoming the biological barriers (Lehner et al., 2019). Therefore, understanding their environmental fate, bioavailability, intake, and the potential effects on different organisms, is critical (Parenti et al., 2019; Lins et al., 2022) for humans. The persistence and degradation of macro- and MIPs contribute to the increase in NAPs in aquatic environments, including seas (Thompson et al., 2004; Cole et al., 2011; Harshvardhan and Jha, 2013; Earni-Cassola et al., 2019; Gigault et al., 2016), shorelines (Browne, 2011), estuaries (Saedi and Thompson, 2014), beach sediments (Imhof et al., 2013), lakes (Eriksen et al., 2013; Free et al., 2014), and freshwater ecosystems (Wagner et al., 2014; Vendel et al., 2017; Brandts et al., 2018; Pitt et al., 2018a; b; Parenti et al., 2019; Barria et al., 2020). These particles not only pose a direct toxicological threat but can also adsorb harmful chemicals, further enhancing their potential for inflicting biological harm (Jinhui et al., 2019; Campanale et al., 2020; Gonzalez-Fernandez et al., 2021). In aquatic organisms, such as zebrafish, NPs can be ingested and bio-fragmented within the body, potentially leading to toxicity and other physiological disruptions (Jovanovic, 2017; Khan and Ali, 2023; Barria et al., 2020; Duan et al, 2020).

Although PS is often used in risk assessments due to its commercial availability and varied sizes and surface charges, other plastics such as PE and PPP are also prevalent in environmental debris but have been less studied (Koelmans et al., 2019; de Ruijter et al., 2020). The current research gap necessitates a more comprehensive investigation of NAPs from various plastic types to assess their toxicity and ecological impacts. The aim of this systematic review is to evaluate the toxicological potential of NAPs in relation to plastic type, particle size, and their ability to adsorb hydrophobic pollutants, with a particular focus on the genotoxic effects in aquatic organisms such as fish. We hypothesize that NAPs upon crossing biological barriers and entering cells may trigger oxidative stress, induce DNA damage, and enhance the bioactivity of adsorbed contaminants. These processes may disrupt critical biological functions, including digestion, metabolism, neural activity and behavior, reproduction, and development, and potentially lead to intergenerational/transgenerational effects that could have significant implications on human health.

## 2 Materials and methods

### 2.1 Literature search strategy

We conducted a comprehensive literature search to find journal articles that examine the toxic effects of NAPs on fish, with a special focus on the impacts at the molecular level. The electronic search was performed in PubMed (http://www.ncbi.nlm.nih.gov/pubmed) until 29 February 2024, using the following search terms: “nanoplastics,” “fish,” and the different polymers of NAPs found in the aquatic environment (e.g., PA, PC, PE, PET, PMMA, PPP, PS, and PVC) (Table 1). The search also included the common names of the six fish species: common carp, fathead minnows, medaka, tilapia, trout, and zebrafish, previously followed in the studies by Dasmahapatra et al. (2023), Dasmahapatra et al. (2024). PubMed was selected as the primary database due to its reputation as a reliable and authoritative source for peer-reviewed scientific literature.

**TABLE 1:** Chemical structures of plastic polymers followed in this review.

Serial number	Common name and molecular formula	IUPAC name	Chemical structure	Molecular weight (Da)/molar mass (g/mol)
1	Polyamide	Poly [imino (alkanedioyl)]	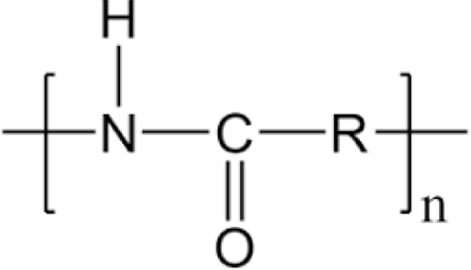	10,000–50,000 Da
2	Polycarbonate (C_16_H_18_O_5_)	Acrylonitrile–butadiene–styrene	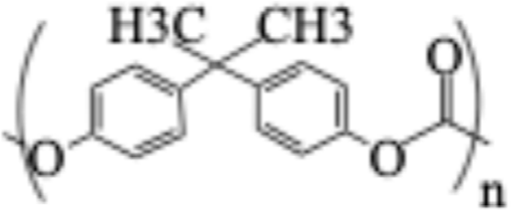	290.32
3	Polyethylene (C_2_H_4_)	Poly (methylene)	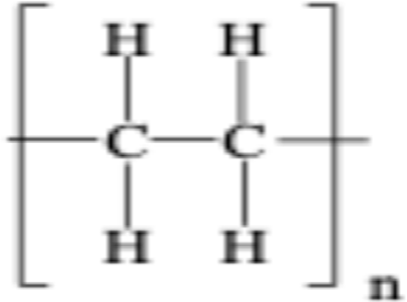	28.05
4	Polyethylene terephthalate (C_10_H_12_O_6_)	Ploy (ethyl benzene-1,4-dicarboxylate)	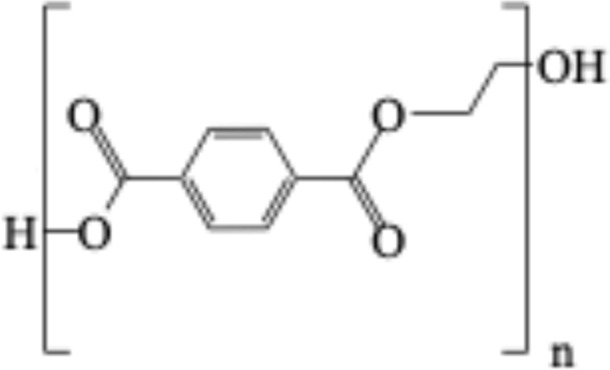	228.19
5	Polypropylene (C_22_H_42_O_3_)	Poly (1-methylethylene)	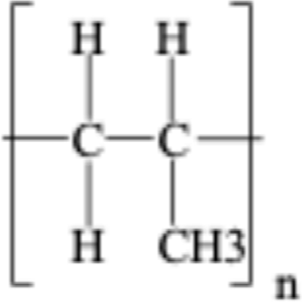	354.56
6	Polyethylene methacrylate (C_5_H_10_O_2_)	Poly (methyl 2-methylpropenoate)	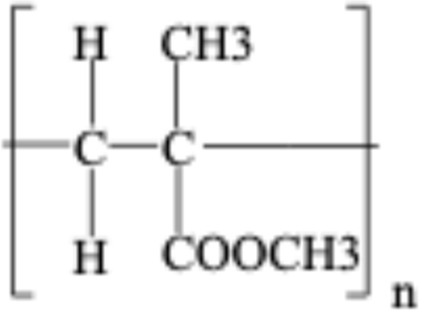	102.13
7	Polystyrene (CH_2_CH(C_6_H_5_)	Poly (1-phenylethylene)	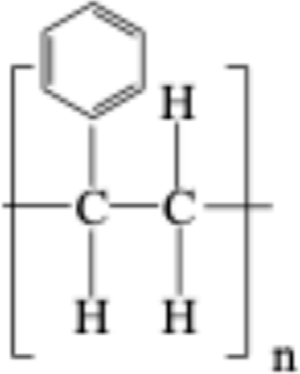	2.01
8	Polyvinyl chloride (C_2_H_3_Cl)	Poly (1-chloroethylene)	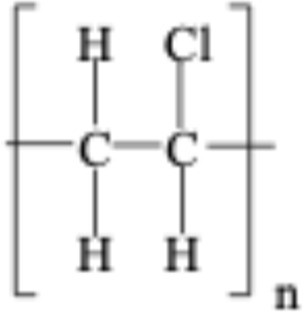	62.49

LDPE = Low-density polyethylene; PA = polyamide; PC = polycarbonate; PE = polyethylene, PET = polyethylene terephthalate; PMMA = polyethylene methacrylate; PPP = polypropylene, PS = polystyrene; PVC = polyvinyl chloride. In two articles, part of the studies used plastic sizes ≤ 100 nm, and part of the studies used plastic sizes ≥ 100 nm. For this reason, these articles are mentioned in the exclusion as well as in the inclusion boxes.

**FIGURE 1:**
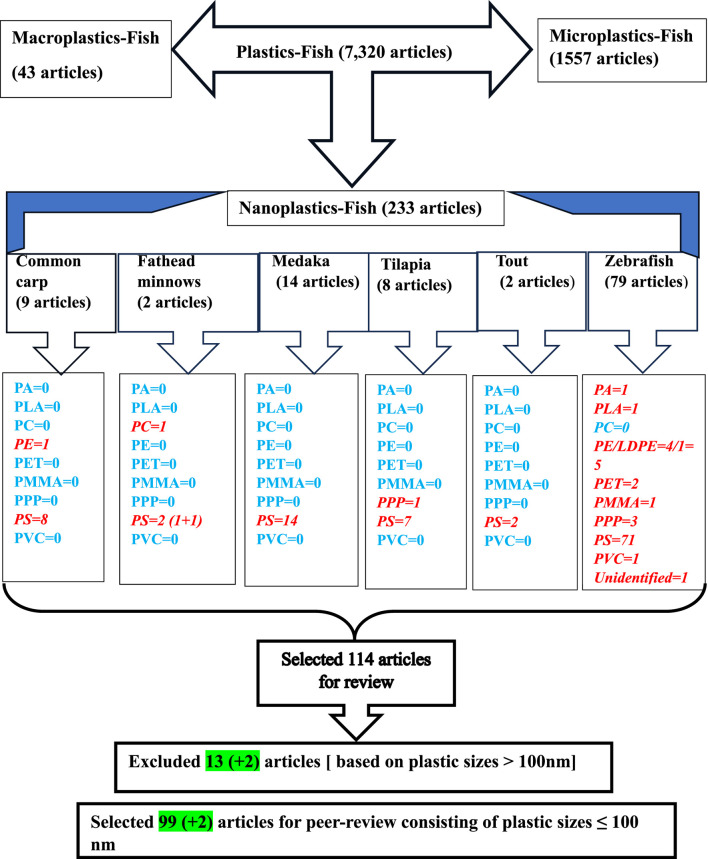
Flow chart of the literature search in PubMed (http://www.ncbi.nlm.nih.gov/pubmed).

For this review, we focused primarily on bony fish, with the selected species serving as representative examples of the class Osteichthyes (Figure 1). The term carp was used to refer collectively to several species, including common carp (*Cyprinus carpio*), grass carp (*Ctenopharyngodon idella*), silver carp (*Hypophthalmichthys molitrix*) and tooth carp (*Aphaniops hormuzensis*) (Estrela et al., 2021; Guimaraes et al., 2021; Hamed et al., 2022; Liu S. et al., 2022; Wu et al., 2022; Zhang X. et al., 2022; Saemi-Komsari et al., 2023; Li Z. et al., 2024; Zhang et al., 2024a). Similarly, the term medaka encompassed Chinese rice fish (*Oryzias sinensis*), Hainan medaka (*Oryzias curvinotus*), Japanese medaka (*Oryzias latipes*), and marine medaka (*Oryzias melastigma*) (Chae et al., 2018; Kang et al., 2021; Zhang et al., 2021; Zhang et al., 2024 YT.; He et al., 2022; Chen Y. et al., 2023; Gao D. et al., 2023; Li X. et al., 2023; Wang F. et al., 2023; Yu et al., 2023; Zhou et al., 2023a; Zhou et al., 2023b; Li X. et al., 2024). The term tilapia was used to refer to various species such as red tilapia (*Oreochromis niloticus*), Nile tilapia (*Oreochromis niloticus*), and Mozambique tilapia (*O. mossambicus*) (Ding et al., 2018; Pang et al., 2021; Hao et al., 2023; Wang W. et al., 2023; Zheng and Wang, 2024; Zheng et al., 2024).

The search yielded 114 peer-reviewed articles that highlight potential developmental, reproductive, neurological, immunological, and behavioral disorders in fish exposed to NAPs (Figure 1; Tables 2–9). A comprehensive summary of the findings has been compiled in Supplementary Table S1, which has been deposited in a public repository [Figshare (https://figshare.com) for reference and future update, if necessary.

**TABLE 2:** List of authors who studied the effects of NAPs on fish.

Serial number	Authors	Polymer	Fish/stage of Development	Sizes	Concentration /dose	Duration	Mode of exposure /additives
1	Aliakbarzadeh et al., (2023)	PS	Zebrafish (Danio rerio)/adults	20-80 nm (average 57.5 nm)	0.1, 1, 10 and 100 µg/L	45 days	Waterborne/4- nonylphenol (1µg/L)
2	Barreto et al., (2021)	PS	Zebrafish (Danio rerio)/embryos	60 nm	0.015, 1.5, and 150 mg/L	96 h	Waterborne/SIM (0.015-150 µg/L)
3	Barreto et al., (2023)	PS	Zebrafish (Danio rerio)/embryos	44 nm	0.015, 1.5 mg/L	96-120 hpf	Waterborne/DPH (0.01 and 10 mg/L)
4	Bashirova et al., (2023)	PET	Zebrafish (Danio rerio)/embryos (6 and 72 hpf)	Hydrodynamic diameter 70±5 nm	to 5, 10, 50, 100, 200 mg/L	Until 96- 120 hpf	Waterborne
5	Bhagat et al., (2022)	PS	Zebrafish (Danio rerio)/embryos	50 nm	1 mg/L	96 h	Waterborne/ nAL2O3(1 mg/L) and nCeO3 (1 mg/L)
6	Brun et al., (2019)	PS	Zebrafish (Danio rerio) /embryos (72 hpf)	25 nm	20 mg/L	Until 120 hpf	Waterborne
7	Chackal et al., (2022)	PS	Zebrafish (Danio rerio)/embryos	100 nm	2.5 and 25 µg/L	Until 7 dpf	Waterborne/BDE-47 (10 ng/L)
8	Chae et al., (2018)	PS	Chinese rice fish (Oryzias sinensis)/ adults (F0) and larvae (F1)	60.39, 57.45, 57.29 nm	5 mg/L	Adults (F0) exposed for 7 days; larvae (F1) exposed for 24 h	Waterborne
9	Chen et al., (2017a)	PS	Zebrafish (Danio rerio)/embryos	47 and 41000 nm	1 mg/L	120 h	Waterborne /EE2 (2 and 20 µg/L)
10	Chen et al., (2017b)	PS	Zebrafish (Danio rerio)/ adults (6 months old)	47 nm	1 mg/L	3 days	Waterborne/BPA (0.78 µg/L)
11	Chen et al., (2022)	PS	Marine medaka (Oryzias melastigma) /embryos	50, 500, and 6000 nm	106 particles/L	19 days	Waterborne
12	Chen et al., (2023a)	PS-NH2 and PS-COOH	Marine medaka (Oryzias melastigma) /embryos	80 nm	10 µg/L	10 days with additional 10 days depuration	Waterborne (regular or acidified sea water)
13	Chen et al., (2023b)	PS, UV-PS, O3-PS	Zebrafish (Danio rerio)/embryos (8 hpf)	80 nm	0.5 and 5 mg/L	Until 120 hpf	Waterborne/ penicillin (1 and 10 µg/L)
14	Chen et al., (2023c)	PS	Zebrafish (Danio rerio)/embryos	50 nm	0.1, 1, 5, 10, 20, 30, and 50 mg/L	Until 120 hpf; evaluated on 5th, 7th, and 12th day	Waterborne/ Sodium nitroprusside (0.1,1, 10, 20, 30 and 40 µM)
15	Chen et al., (2024)	PS	Zebrafish (Danio rerio)/embryos (8hpf)	80, 200, 500 nm	0.1, 0.5, 1, 5, 10, 25, and 50 mg/L	120 hpf, depurate 10 days	Waterborne
16	Cheng et al., (2022)	PS	Zebrafish (Danio rerio)/embryos	50, 100 nm and micro-PS	0.1, 0.5, 2 and 10 mg/L	120 hpf	Waterborne
17	Clark et al., (2023a)	PS-Pd	Rainbow trout (Oncorhynchus mykiss)/juvenile	200 nm	10 mg/kg food	3 and 7 days; depurated 7 days	Dietary
18	Clark et al., (2023b)	PS	Rainbow trout (Oncorhynchus mykiss)/juvenile	35±8 nm	5.9 µg/g food	3,7,14 days	Dietary
19	Dai et al., (2023)	PS	Zebrafish (Danio rerio)/embryos	20 nm	2, 5, and 8 mg/L	22, 46, and 72 hpf	Waterborne
20	Deng et al., (2023)	PS	Zebrafish (Danio rerio)/adults	100 nm	500 ng/mL	28 days	Waterborne
21	De Souza Teodoro et al., (2024)	PET	Zebrafish (Danio rerio)/embryos	68.06-955 nm and 1305000-2032000 nm	0.5, 1, 5, 10, and 20 mg/L	6 days	Waterborne
22	Ding et al., (2018)	PS	Red Tilapia (Oreochromis niloticus)/juveniles	100 nm	1, 10, 100 µg/L	14 days	Waterborne
23	Ding et al., (2020)	PS	Red Tilapia (Oreochromis niloticus)/juveniles	300, 5000, 7000-9000 nm	100 µg/L	6 and 14 days	Waterborne
24	Du et al., (2024)	PS	Zebrafish (Danio rerio)/adults	50-100 nm	1000 µg/L	21 days	Waterborne/dietary exposure to high fat diet (24% crude fat)
25	Duan et al., (2023)	PS	Zebrafish (Danio rerio)/embryos (4 hpf)	50 nm	0.1, 0.5, and 1 mg/L	72 h	Waterborne
26a	Elizalde-Velazquez et al., (2020)	PS	Fathead minnows (Pimephales promelas)/ adult (males)	50 nm	5 µg/L (0.1 ml injected volume)	48 h	IP
26b	Elizalde-Velazquez et al., (2020)	PS	Fathead minnows (Pimephales promelas)/ adult (males)	50 nm	5 µg/L	48 h	Trophic transfer (fed with daphnia which were consumed PS-exposed green algae)
27	Estrela et al., (2021)	PS	Grass carp (Ctenopharyngodon idella)/juveniles	23.03±0.266 nm	760 µg/L	72 h	Waterborne/ZnO2 (760 µg/L)
28	Feng et al., (2022)	PS	Zebrafish (Danio rerio)/embryos	100 nm	100, 200, and 400 mg/L	96 h	Waterborne
29	Gao et al., (2023a)	PS	Hainan medaka (Oryzias curvinotus)	80 nm	200 µg/L	7 days	Waterborne/F53B (500 µg/L)
30	Gao et al., (2023b)	PS	Zebrafish (Danio rerio) /embryos (3 hpf)	80 nm	5, 10, 25, 50, 100 µg/L	96 hpf	Waterborne/APAP (2-8 mM)
31	Geum and Yeo, (2022)	PS	Zebrafish (Danio rerio)/embryos	50 nm	5 mg/L	4,8,12,24,32, 48, 72 hpf	Waterborne/PHE (0.5 and 1 mg/L and mucin from jelly fish (50 µg/L)
32a	Greven et al., (2016)	PC	Fathead minnows (Pimephales promelas)/ neutrophils of adults	158.7 nm	0.025, 0.05, 0.1, 0.2, and 100 µg/mL	2h	In vitro
32b	Greven et al., (2016)	PS	Fathead minnows (Pimephales promelas)/ neutrophils of adults	41 nm	0.025, 0.05, 0.1, 0.2, and 100 µg/mL	1-2 h	In vitro
33	Guimaraes et al., (2021)	PS	Grass carp (Ctenopharyngodon Idella)/juveniles	23.03±0.266 nm (20-26 nm)	0.04 ng/L, 34 ng/L, and 34 µg/L	20 days	Waterborne
34	Habumugisha et al., (2023)	PS	Zebrafish (Danio rerio)/adults (males)	50 nm	5, 10, 15 mg/L	30 days; depurated 16 days; evaluated; evaluated on 3, 6, 12, 18, 24, 30, 34, 38, 42, and 46 days.	Waterborne
35	Hamed et al., (2022)	PE	Common carp (Cyprinus carpio)/juvenile	100 nm and > 100 nm	100 mg/L	15 days	Waterborne
36	Hao et al., (2023)	PS	Tilapia (Oreochromis niloticus)/ juveniles	86 and 185 nm	1 mg/L	21 days, depurated 7 days	Waterborne
37	He et al., (2021).	PS	Zebrafish (Danio rerio)/adults (males and females)	46 and 5800 nm	2 mg/L	21 days	Waterborne /TPhP (0.08, 0.5, 0.7, 1, 1.2, 1.5 mg/L)
38	He et al., (2022)	PS	Marine medaka (Oryzias melastigma)/adults	100 nm	3.45 mg/g	30 days [F0]. [F1 offspring were evaluated 60 dph without any exposure)	Dietary [F0]/ /SMG (94.62 mg/g)
39	Kang et al., (2021)	PS	Marine medaka (Oryzias melastigma)/larvae (7 dph)	50 nm and 45 µm (45,000 nm)	10 µg/mL and 2.5 µg/mL	24 h (10µg/L). 1, 7, 14, and 120 days (2.5 µg/mL)	Waterborne
40	Kantha et al., (2022)	PS	Zebrafish (Danio rerio)/ embryos	25 nm	10, 25, and 50 mg/L	96 h	Waterborne
41	Khan and Ali (2023)	PE	Zebrafish (Danio rerio)/ adults	10-100 µm (10,000-100,000 nm)	Unknown	24h	Waterborne
42	Lee et al., (2019)	PS	Zebrafish (Danio rerio)/ embryos	50, 200, 500 nm	0.1 mg/L	6, 24, 96 h	Waterborne
43	Lee et al., (2022)	PPP	Zebrafish (Danio rerio)/ embryos (24 hpf and 72 hpf)	562.15±118.47 nm	50 mg/L	24 h	Waterborne
44	Li et al., (2023a)	PS	Zebrafish (Danio rerio)/ adults	80 nm	15 and 150 mg/L	28 days	Waterborne/vitamin D (280 and 2800 IU/kg)
45	Li et al., (2023b)	PS	Marine medaka (Oryzias melastigma) /juveniles (2 months old)	100 nm	1 mg/L	30 days	Waterborne/SMX (100µg/L)
46	Li et al., (2023c)	PE	Zebrafish (Danio rerio)/adults	70 and 13500 nm	20 mg/L	21 days	Waterborne/PEMIP (20 mg/L)
47	Li et al., (2024a)	PS	Grass carp (Ctenopharyngodon idella)/juveniles	80 nm	10, 100, 1000 µg/L	8 days; coexposure 3 days with 5 days preexposure with PS	Waterborne/Aeromonas hydrophilia (2X107CFU/mL)
48	Li et al., (2024b)	PS	Marine medaka (Oryzias melastigma) / larvae (3 dph)	70, 500 and 2000 nm	20, 200, and 2000 /L	90 days	Trophic transfer (fed to rotifers and the rotifers were fed by the fish)
49	Lin et al., (2023)	PS	Zebrafish (Danio rerio)/adults (males and females)	70 nm	2 mg/L	21 days	Waterborne/DES (1,10, 100 ng/L)
50	Ling et al., (2022)	PS	Zebrafish (Danio rerio)/adults (males and females)	70 nm	100µg/L	90 days	Waterborne /MCLR (0.9, 4.5, and 22.5 µg/L)
51	Liu et al., (2021)	PS	Zebrafish (Danio rerio)/embryos	100 nm	10 µg/L	Until 120 hpf	Waterborne/BMDBM (1,10, and 100 µg/L)
52	Liu et al., (2022a)	PS	Grass carp (Ctenopharyngodon idella)/juveniles	80 nm	20, 200, 2000 µg/L	7 days	Waterborne/TC (5000 µg/L)
53	Liu et al., (2022b)	PS	Zebrafish (Danio rerio)/embryos	100 nm	10 µg/L	144h and depurated 72 h	Waterborne/AV0 (10 µg/L)
54a	Manuel et al., (2022)	PMMA	Zebrafish (Danio rerio)/embryos	32 nm	0.001, 0.01,0.1, 1, 10, 100 mg/L	96 h	Waterborne
54b	Manuel et al., (2022)	PS	Zebrafish (Danio rerio)/embryos	22 nm	0.001, 0.01, 0.1, 1, 10, 100 mg/L	96 h	Waterborne
55	Martin et al., (2023)	PS	Zebrafish (Danio rerio)/embryos	30 and 100 nm	0.1, 1, and 10 mg/L	96 h	Waterborne
56	Martinez-Alvarez et al., (2022)	PS	Zebrafish (Danio rerio)/embryos	50, 500, and 4500 nm	0.069 µg/L- 50.1 mg/L	120 h	Waterborne /B(a)P (0.1-10 mg/L)
57	Martin-Folgar et al., (2023)	PS	Zebrafish (Danio rerio)/embryos	30 nm	0.1, 0.5 and 3 mg/L	120 hpf	Waterborne
58a	Monikh et al., (2022)	PE	Zebrafish (Danio rerio) /embryos (6 hpf)	50 nm	3X1010 particles/L (0.000 25 mg/L)	24 h	Waterborne
58b	Monikh et al., (2022)	PPP	Zebrafish (Danio rerio) /embryos (6 hpf)	50 nm	3X1010 particles/L (.00022 mg/L)	24h	Waterborne
58c	Monikh et al., (2022)	PS	Zebrafish (Danio rerio) /embryos (6 hpf)	200 and 600 nm	3X1010 particles/L (PS 200 nm =0.13 mg/L; PS 600=3.5 mg/L)	24 h	Waterborne/B(a)P (10 µg/L)
58d	Monikh et al., (2022)	PVC	Zebrafish (Danio rerio) /embryos (6 hpf)	200 nm	3X1010 particle/L (0.17 mg/L)	24 h	Waterborne/B(a)P (10 µg/L)
59	Pang et al., (2021)	PS	Tilapia (Oreochromis mossambicus)/larvae	100 nm	20 mg/L	7 days and depurated 7 days	Waterborne
60	Parenti et al., (2019)	PS	Zebrafish (Danio rerio) /embryos (72 hpf)	500 nm	1 mg/L	2 days (until 120 hpf)	Waterborne
61	Park and Kim (2022)	PS	Zebrafish (Danio rerio) /embryos (1 dpf)	400 and 1000 nm	7.5-60 mg/L	3 days	Waterborne
62	Pedersen et al., (2020)	PS	Zebrafish (Danio rerio) /embryos (6 hpf)	50, 200 nm	10, 100, 1000, 10,000 µg/L	Until 120 hpf	Waterborne
63	Pitt et al., (2018a)	PS	Zebrafish (Danio rerio) /embryos (6 hpf)	51 nm	0.1, 1, and 10 mg/L	120 h	Waterborne
64	Pitt et al., (2018b)	PS	Zebrafish (Danio rerio)/adults	42 nm	1 mg/g	7 days	Dietary
65	Saemi-Komsari et al., (2023)	PS	Tooth Carp (Aphaniops hormuzensis)/ adults	100-300 nm (average 185 nm)	1, 5,10,25, 100, 200 mg/L and 1.1. 0.1, 1, 5 mg/L	96h (waterborne)/3, 14, 28 days (dietary exposure)	Waterborne and dietary/ TCS (0.5 mg/kg)
66	Santos et al., (2022)	PS	Zebrafish (Danio rerio)/embryos	44 nm	0.015, 1.5,15, and 150 mg/L	96-120 hpf	Waterborne/PHN (0.2, 2, and 20 mg/L)
67	Santos et al., (2024)	PS	Zebrafish (Danio rerio)/embryos	23.03 ±0.266 nm	0.04 ng/l, 34 ng/L and 34 µg/L	144 hpf	Waterborne
68	Sarasamma et al., (2020)	PS	Zebrafish (Danio rerio)/adults	70 nm	0.5, 1.5 and 5 mg/L	7 days, 30 days, 7 weeks	Waterborne
69	Sendra et al., (2021)	PS	Zebrafish (Danio rerio) /larvae (120 hpf)	50, 1000, 50,000 nm	10 mg/L	7 days	Waterborne
70	Senol et al., (2023)	PS	Zebrafish (Danio rerio)/adults	134±2.9 nm	25 mg/L	96 h	Waterborne at 28°, 29°, and 30° C
71	Sokmen et al., (2020)	PS	Zebrafish (Danio rerio)/embryos	20 nm	3 nL of 270 mg/L	120 h	Injected to fertilized eggs
72	Sulukan et al., (2022a)	PS	Zebrafish (Danio rerio) /embryos (4 hpf)	20 nm	3 nL of 270 mg/L	Grown 6 months and evaluated F1 offspring	Injected to fertilized eggs
73	Sulukan et al., (2022b)	PS	Zebrafish (Danio rerio)/adults	100 nm	25 mg/L	96 h	Waterborne at 28°, 29°, and 30° C
74	Suman et al., (2023)	PS	Zebrafish (Danio rerio)/embryos	500 nm	0.1, 1, and 10 mg/L	6 days	Waterborne
75	Sun et al., (2021)	PE	Zebrafish (Danio rerio) /embryos (6 hpf)	Hydrodynamic size 191.10 ±3.13 nm	25, 50, 100, 200, 400, 600, 800, 1000 µg/mL	48-96 h	Waterborne
76a	Tamayo-Belda et al. (2023))	LDPE	Zebrafish (Danio rerio) /embryos (4 hpf)	164-91 nm	0.001, 0.01,0.1,1, and 10 mg/L	4h-96 h	Waterborne
76b	Tamayo-Belda et al. (2023))	PLA	Zebrafish (Danio rerio) /embryos (4 hpf)	122-712 nm	0.001, 0.01,0.1,1, and 10 mg/L	4h-96 h	Waterborne
76c	Tamayo-Belda et al. (2023))	PPP	Zebrafish (Danio rerio) /embryos (4 hpf)	164-220 nm	0.001, 0.01,0.1,1, and 10 mg/L	4h-96 h	Waterborne
76d	Tamayo-Belda et al. (2023))	PS	Zebrafish (Danio rerio) /embryos (4 hpf)	91-825 nm	0.001, 0.01,0.1,1, and 10 mg/L	4h-96 h	Waterborne
77	Teng et al., (2022a)	PS-NH2 PS-COOH	Zebrafish (Danio rerio)/embryos	30-51 nm	30 and 50 mg/L	120 h	Waterborne
78	Teng et al., (2022b)	PS	Zebrafish (Danio rerio)/juveniles and adults	44 nm	1, 10, and 100 µg/L	30 and 60 days	Waterborne
79	Teng et al., (2023)	PS	Zebrafish (Danio rerio)/ adults	80 nm	15 and 150 µg/L	21 days	Waterborne/ vit D (280-2800 IU/kg, via food)
80	Trevisan et al., (2019)	PS	Zebrafish (Danio rerio) /embryos (6 hpf)	44 nm	1.1. 1, 10 mg/L	96 h	Waterborne/PAH (5.07-25.36 µg/L)
81	Trevisan et al., (2020)	PS	Zebrafish (Danio rerio)/embryos	44 nm	1 mg/L	7 days	Waterborne/PAH (5.073 ng/mL)
82	Van Pomeren et al., (2017)	PS	Zebrafish (Danio rerio)/embryos	25, 50, 250,700 nm	5-50 mg/L	48 h	Waterborne
83	Varshney et al., (2023)	PS	Zebrafish (Danio rerio)/embryos	15 nm	50 mg/L	96 h	Waterborne/ p, p’-DDE (100 µg/L)
84	Wang et al, (2022)	PS	Zebrafish (Danio rerio)/embryos	80 nm	0.05, 0.1, 1, and 10 mg/L	120 hpf	Waterborne/BDE-47 (0.1 mg/L)
85	Wang et al., (2023a)	PS	Marine medaka (Oryzias melastigma) / adults	100 nm	5 mg/ g food	30 days	Feeding/ SMG (0.5 and 5 mg/g food)
86	Wang et al., (2023b)	PS	Tilapia/juveniles	100, 500, and 5,000 nm	1, 10, 100 µg/L	7 days	Waterborne
87	Wang et al, (2023c)	PS	Zebrafish (Danio rerio)/embryos	80 nm	0.05, 0.1, 1, 5, and 10 mg/L	12-120hpf	Waterborne/BDE-47 (0.1 and 10 mg/L)
88	Wang et al., (2023d)	PS-COOH	Zebrafish (Danio rerio)/embryos	50 nm	1, 5, and 10 mg/L	144 h	Waterborne
89	Wang et al., (2023e)	Nanoplastics (NAPs)	Zebrafish (Danio rerio)/adults (120 dpf)	100 nm	1 mg/L	45 days	Waterborne/BPAF (200 µg/L)
90	Wu et al., (2021)	PS	Zebrafish (Danio rerio)/adults	70 nm	100 µg/L	45 days; F1 embryos were evaluated without any further exposure	Waterborne/MCLR (0.9, 4.5, and 22.5 µg/L)
91	Wu et al., (2022)	PS	Carp /adult	50, 100, and 400 nm	1000 µg/L	28 days	Waterborne
92	Wu et al., (2023)	PPP	Tilapia (Oreochromis niloticus)/juveniles	100 nm and 100 µm (100,000 nm)	1, 10, and 100 mg/L	21 days	Waterborne
93	Xie et al., (2021)	PS	Zebrafish (Danio rerio)/adults	80 and 8000 nm	1 mg/L (80 nm); 10 µg/L (8000 nm)	21 days	Waterborne
94	Yang et al., (2023)	PS	Zebrafish (Danio rerio)/adults	100 and 20,000 nm	100 and 1000 µg/L	4 days, depurate 3 days	Waterborne
95	Ye et al., (2024)	PS	Zebrafish (Danio rerio)/adults	50 nm	1 mg/L	21 days	Waterborne/ homosolate (0.0262-262 µg/L)
96	Yu et al., (2022a)	PS	Zebrafish (Danio rerio)/adults	100 nm	20 and 200 µg/L	3 weeks	Waterbone/lead (50 µg/L)
97	Yu et al., (2022b)	PS	Zebrafish (Danio rerio)/adults	40-54 nm; 394-407 nm; 4,000-8,000 nm; 45,000-85,000 nm; 158,000-234,000 nm	60-338 µg/L	30 days	Waterborne/tetracycline (100 µg/L)
98	Yu et al., (2023)	PS	Marine medaka (Oryzias melastigma) / embryos (6hpf)	50 nm	55 µg/L	21 days	Waterborne /BPA (100 µg/L)
99a	Zhang et al., (2020)	PS	Zebrafish (Danio rerio)/embryos	70 ± 9.21 nm	Injected 0.52 nL of 1000, 3000, and 5000 mg/L	Hatched larvae depurate 4 weeks	Injected to eggs
99b	Zhang et al., (2020)	PS	Zebrafish (Danio rerio)/embryos	70 ± 9.21 nm	0.5 and 5 mg/L	Until the hatching, depurate 4 weeks	Waterborne
100	Zhang et al., (2021)	PS	Marine medaka (Oryzias melastigma)	100 nm	5 mg/g food	30 days	Feeding/SMG 0.5, and 5 mg/g
101a	Zhang et al., (2022b)	PS	Grass carp (Ctenopharyngodon idella)/ embryos (12hpf)	80 and 8000 nm	5, 15, and 45 µg/L	2-8 h	Waterborne
101b	Zhang et al., (2022b)	PS	Grass carp (Ctenopharyngodon idella)/ larvae (24 hph)	50 and 5000 nm (green fluorescence). 1000 and 5000 (red fluorescence	10 µg/L	12-96 h	Waterborne
102	Zhang et al., (2022c)	Polyamide (PA)	Zebrafish (Danio rerio)/embryos	5-50 µm (5,000-50,000 nm)	1, 10, and 20 mg/L	2hpf-10dpf	Waterborne
103	Zhang et al., (2023)	PS	Zebrafish (Danio rerio)/adults	100 nm	1 mg/L	30 days	Waterborne/arsenic (200 µg/L)
104	Zhang et al., (2024a)	PS	Silver carp (Hypophthalmichthys molitrix)/ adults	80 nm	10 and 1000 µg/L	96 h	Waterborne/Microcystin-LR (1µg/L)
105	Zhang et al., (2024b)	PS-plain, PS-COOH, PS-NH2	Marine medaka (Oryzias melastigma) /adults (10-12 months old)	Z-average of plain PS =244.0±11.6 nm, PS-COOH =294.7±8.6 nm, and PS-NH2 = 277.0±15.9 nm	3.62 mg/g of food	30 days, depurated for 21 days	Feeding/SMZ (4.62 mg/g food)
106	Zhang et al., (2024c)	PS	Zebrafish (Danio rerio)/adults	100 nm	1 ng/L	30 days	Waterborne/arsenic (1 mg/L)
107	Zhao et al., (2021)	PS	Zebrafish (Danio rerio)/adults (males and females)	54.5 ±2.8 nm	10 mg/L	120 days; evaluated F0 and F1 larvae without further exposure	Waterborne/TDCIPP (0.47, 2.64, or 12.78 µg/L)
108	Zheng and Wang (2024)	PS	Tilapia (Oreochromis niloticus)/larvae	80 nm and 20 µm (20,000 nm)	100 µg/L	28 days	Waterborne
109	Zheng et al., (2024)	PS	Tilapia (Oreochromis niloticus)/larvae	80, 2000, 20,000 nm	100 µg/L	28 days	Waterborne
110	Zhou et al., (2023a)	PS	Japanese medaka (Oryzias latipes)/ adults	100 nm	10, 104, 106 particles/ L (1.79589 X1013 particles/10 mg concentration)	3 months	Waterborne
111	Zhou et al., (2023b)	PS	Japanese medaka (Oryzias latipes)/ larvae (9 dph) and adults (60 dph)	100 nm	Larvae= (1014 items/L or 55 mg/L). Adults= (10 items/L or 5.5X10-12 mg/L; 104/L or 5.5X10-9 mg/L; 106 items/L or 5.5X10-7 mg/L)	Larvae 48 h. Adults 3 months.	Waterborne
112	Zhou et al., (2023c)	PS	Zebrafish (Danio rerio)/embryos	100, 500, 1000 nm	10 mg/L or 2.2 X1012 particles/L for 100 nm;1.76X1010 particles/L for 500 nm; 2.2X109 particle/L for 1000 nm.	5 days	Waterborne
113	Zhou et al., (2023d)	PS	Zebrafish (Danio rerio)/adults	50 ±3 nm	1 mg/L	4 weeks	Waterborne
114.	Zuo et al., (2021)	PS	Zebrafish (Danio rerio)/adults	70 nm	100µg/L	21 days; F1 (120 hpf) were evaluated without further exposure	Waterborne/MCLR (0.9, 4.5, and 22.5 µg/L)

Blocks highlighted in yellow are coexposure studies. Elizalde-Velazquez et al. (2020) used two different methods ofexposure (injection and trophic transfer) of PS and mentioned inone article. Greven et al. (2016) studied the effects of PC and PS in one article. Manuel et al. (2022) reported the effects of PMMA and PS inzebrafish in one article. Monikh et al. (2022) reported the effects of PE, PPP, PS, and PVC in one article. Tamayo-Belda et al. (2023) reported the effects of PLA, PP, PS, and LDPE in one article. Zhang et al. (2020) used two different methods of exposure (injection and waterborne) of PS and mentioned in one article. Zhang C. et al. (2022) used two different life stages of zebrafish(embryo larvae) for PS exposure and described in one article. Wang L. et al. (2023) did not mention the type of NAPs used inthe experiment.AVO = avobenzone; BDE-47 = Polybrominated diphenyl ether: BMDBM = methoxydibenzoylmethane; BPA = bisphenol A; EE2 =17 α-ethynyl estradiol; IP = intraperitoneal injection; LDPE = lowdensitypolyethylene; MCLR = microcystin-LR; PA = polyamide; PC = polycarbonate; PE = polyethylene; PET = polyethyleneterephthalate; PHN = phenmedipham; PLA = polylactic acid; PMMA = polymethylmethacrylate; PPP = polypropylene; PS =polystyrene; SIM = simvastatin; SMZ = sulfamethazine; TDCIPP = tris (1,3-dichloro-2-propyl) phosphate; TPhP =triphenyl phosphate; TC = tetracycline; TCS = triclosan.

**TABLE 3:** Articles excluded from reviews (based on the size and the mode of exposure).

	Fish	Polymer (name)	Sizes (mode of exposure)	Developmental stages	References
1	Zebrafish	PA	∼32, 500 nm	Embryos (2 hpf)	Zhang et al. (2022c)
2a	Fathead minnows	PC	158.7 nm (*in vitro*)	Adults (neutrophils)	Greven et al. (2016)
2b	Fathead minnows	PC	41 nm (*in vitro*)	Adults (neutrophils)	Greven et al. (2016)
3	Zebrafish	PE	191.10 ± 3.13 nm	Embryos (6 hpf)	Sun et al. (2021)
4	Zebrafish	PE	10,000–100,000 nm	Adults (8–10 months old)	Khan and Ali (2023)
5	Zebrafish	PPP	562.15 ± 118.47 nm	Embryos (24 hpf and 72 hpf)	Lee et al. (2022)
6a	Zebrafish	PPP	164–220 nm	Embryos (4 hpf	Tamayo-Belda et al. (2023)
6b	Zebrafish	PLA	122–712 nm	Embryos (4 hpf	Tamayo-Belda et al. (2023)
7	Marine medaka (*Oryzias melastigma*)	PS	244–277 nm	Adult	Zhang et al. (2024b)
8	Rainbow trout (*Oncorhynchus mykiss*)	PS	∼200 nm	Juveniles	Clark et al. (2023a)
9	Red tilapia (*Oreochromis niloticus*)	PS	300, 500, and 7,000–9,000 nm	Juveniles	Ding et al. (2020)
10	Zebrafish	PS	500 nm	Embryos (72 hpf)	Parenti et al. (2019)
11a	Zebrafish	PS	200 and 600 nm	Embryos (6 hpf)	Monikh et al. (2022)
11b	Zebrafish	PVC	200 nm	Embryos	Monikh et al. (2022)
12	Zebrafish	PS	400–1,000 nm	Embryos (1 dpf)	Park and Kim (2022)
13	Zebrafish	PS	500 nm	Embryos	Suman et al. (2023)
14	Zebrafish	PS	134 ± 2.9 nm	Adult	Senol et al. (2023)
15	Zebrafish	Nanoplastics	100 nm	Adults (120 dpf)	Wang et al. (2023e)

Greven et al. (2016) studied the effects of PC and PS on RBCs of adult fathead minnows in vitro. Monikh et al. (2022) studied the effects of PS and PVC on zebrafish and included in one article. Tamayo-Belda et al. (2023) described the effects of PPP and PLA on zebrafish embryos in one article; Wang L. et al. (2023) did not mention the types of NAPs used in this study.

**TABLE 4:** Articles included both MIPs and NAPs during investigations.

	Fish	Polymer	MIP (size)	NAP (size)	Developmental stage	References
1	Common carp (*Cyprinus carpio*)	PE	>5 mm->100 nm	<100 nm	Juvenile	Hamed et al. (2022)
2	Zebrafish	PE	13.5 µm (13,500 nm)	70 nm	Adult	Li et al. (2023c)
3	Zebrafish	PET	>100 nm −2032 µm (20,32,000 nm)	68.06–100 nm	Embryos	de Souza Toedoro et al. (2024)
4	Carp	PS	400 nm	50 and 100 nm	Adult	Wu et al. (2022)
5a	Grass carp (*Ctenopharyngodon idella*)	PS	8 µm (8,000 nm)	80 nm	Embryos	Zhang et al. (2022b)
5b	Grass carp (*Ctenopharyngodon idella*)	PS	5 µm (8,000 nm)	50 nm	Larvae	Zhang et al. (2022b)
6	Tooth carp (*Aphaniops hormuzensis*)	PS	300 nm	100 nm	Adult	Saemi-Komsari et al. (2023)
7	Marine medaka (*Oryzias melastigma*)	PS	500 and 6,000 nm	50 nm	Embryos	Chen et al. (2022)
8	Marine medaka (*Oryzias melastigma*)	PS	45 µm (45,000 nm)	50 nm	Larvae (7 dph)	Kang et al. (2021)
9	Marine medaka (*Oryzias melastigma*)	PS	500 nm and 2 µm (2,000 nm)	70 nm	Larvae (3 dph)	Li et al. (2024b)
10	Tilapia (*Oreochromis niloticus*)	PPP	100 µm (100,000 nm)	100 nm	Juveniles	Wu et al. (2023)
11	Tilapia (*Oreochromis niloticus*)	PS	2 and 20 µm (2,000 and 20,000 nm)	80 nm	Larvae	Zheng et al. (2024)
12	Nile tilapia (*Oreochromis niloticus*)	PS	185 nm	100 nm	Juveniles	Hao et al. (2023)
13	Nile tilapia (*Oreochromis niloticus*)	PS	500 and 5,000 nm	100 nm	Juveniles	Wang et al. (2023b)
14	Zebrafish	PS	41 µm (41,000 nm)	47 nm	Embryos	Chen et al. (2017a)
15	Zebrafish	PS	250 and 700 nm	25 and 50 nm	Embryos	Van Pomeren et al. (2017)
16	Zebrafish	PS	200 and 500 nm	50 nm	Embryos	Lee et al. (2019)
17	Zebrafish	PS	200 nm	50 nm	Embryos	Pedersen et al. (2020)
18	Zebrafish	PS	500 and 4,500 nm	50 nm	Embryos	Martinez-Alvarez et al. (2022)
19	Zebrafish	PS	500 and 1,000 nm	100 nm	Embryos	Zhou et al. (2023c)
20	Zebrafish	PS	200 and 500 nm	80 nm	Embryos and larvae	Chen et al. (2024)
21	Zebrafish	PS	1,000 nm and 50 µm	50 nm	Larvae	Sendra et al. (2021)
22	Zebrafish	PS	5,800 nm	46 nm	Adults (male and female)	He et al. (2021)
23	Zebrafish	PS	8,000 nm	80 nm	Adults	Xie et al. (2021)
24	Zebrafish	PS	394–407 nm, 4–8 μm, (4,000–8,000 nm), 45–85 µm (45,000–85,000 nm), and 158–234 µm (158,000–234,000 nm)	40–54 nm	Adults	Yu et al. (2022b)
25	Zebrafish	PS	20 µm (20,000 nm)	100 nm	Adults	Yang et al. (2023)
26a	Zebrafish	PS	122, 220, 712, and 825 nm	91 nm	Embryos (4 hpf–96 hpf)	Tamayo-Belda et al. (2023)
26b	Zebrafish	LDPE	164,106, 342, and 122 nm	91 nm	Embryos (4 hpf–96 hpf)	Tamayo-Belda et al. (2023)

MIP , microplastics (diameter of the polymer is > 100 nm); NAPs , nanoplastics (diameter of the polymer is ≤100 nm); Tamayo-Belda et al. (2023) measured the diameter of the plastic every day during the exposure period (day 0, day 1, day 2, day 3, and day 4).

**TABLE 5:** Accumulation of nanoplastics in the specific organs of fish at various stages of development.

Name of the plastics	Fish	Developmental stages	Nanoplastic size/diameter	Mode of exposure/duration	Accumulated (tissues/organs) or studied organs	References
PE	Common carp (*Cyprinus carpio)*	Juveniles	100 nm	Waterborne-(15 mg/L)-15 days)	Brain and eye	Hamed et al. (2022)
LDPE	Zebrafish (*Danio rerio*)	Embryos (4 hpf)	91 nm	Waterborne (0.001, 0.01, 0.1, 1, 10, and 10 mg/L), 96 hpf	Vitelline membrane	Tamayo-Belda et al. (2023)
PE	Zebrafish (*Danio rerio*)	Embryos (6 hpf)	50 nm	Waterborne (3 × 10^10^ particles/L or 0.00025 mg/L), 24 h	Whole embryo	Monikh et al. (2022)
PE	Zebrafish (*Danio rerio*)	Adults (3 months)	70 nm	Waterborne (20 mg/L), 21 days	Gill/gut/intestine /liver	Li et al. (2023c)
PET	Zebrafish (*Danio rerio*)	Embryos (6 and 72 hpf)	70 ± 5 nm	Waterborne (5, 10, 50, 100, and 200 mg/L), until 96–120 hpf	Liver, intestine, and kidney	Bashirova et al. (2023)
PET	Zebrafish (*Danio rerio*)	Embryos	68.06 nm and above	Waterborne (0.5, 1, 5, 10, and 20 mg/L), 6 days	Chorion surface	de Souza Toedoro et al. (2024)
PMMA	Zebrafish (*Danio rerio*)	Embryos	32 nm	Waterborne (0.001, 0.01, 0.1, 1, 10, and 100 mg/L), 96 h	Whole embryo	Manuel et al. (2022)
PPP	Tilapia (*Oreochromis niloticus*)	Juveniles (10 ± 1 g; length 13 ± 1 cm)	100 nm	Waterborne (0.001, 0.01, and 0.1 mg/L), 21 days	Liver	Wu et al. (2023)
PPP	Zebrafish (*Danio rerio*)	Embryos (6 hpf)	50 nm	Waterborne (3 × 10^10^ particles/L or 0.000022 mg/L), 24 h	Whole embryos	Monikh et al. (2022)
PS	Carp	Adults	50 and 100 nm	Waterborne (0.1 mg/L), 28 days	Heart	Wu et al. (2022)
PS	Grass carp (*Ctenopharyngodon idella*)	Embryos (12 hpf)	50–80 nm	Waterborne (0.005–0.045 mg/L); 2,4, and 8 h	On the chorion	Zhang et al. (2022b)
PS	Grass carp (*Ctenopharyngodon idella*)	Juveniles	23.03 ± 0.266 nm	Waterborne (0.76 mg/L), 72 h	Blood/liver/brain	Estrela et al. (2021)
PS	Grass carp (*Ctenopharyngodon idella*)	Juveniles	20–26 nm	Waterborne (0.00000004–0.034 mg/L), 20 days	Liver/brain	Guimaraes et al. (2021)
PS	Grass carp (*Ctenopharyngodon idella*)	Juveniles	80 nm	Waterborne, 0.02, 0.2, and 2 mg/L (7 days)	Liver and intestine	Liu et al. (2022a)
PS	Grass carp (*Ctenopharyngodon idella*)	Juveniles	80 nm	Waterborne (0.01, 0.1, and 1 mg/L), 8 days	Gut/intestine	Li et al. (2024a)
PS	Silver carp (*Hypophthalmichthys molitrix*)	Adults	80 nm	Waterborne (0.01 and 1 mg/L), 96 h	Gut/intestine/liver	Zhang et al. (2024a)
PS	Tooth carp (*Aphaniops hormuzensis)*	Adult	100 nm	Waterborne (1, 5, 10, 25, 50, 100, and 200 mg/L), 96 h Diet (0.01, 0.1, 1, and 5 mg/kg), 3, 14, and 28 days	Gut, gill, liver, muscle, and skin	Saemi-Komsari et al. (2023)
PS	Fathead minnows (*Pimephales promelas*)	Adult males	50 nm	IP-injected (0.1 mL of 0.005 mg/L), 48 h	Liver and head kidney	Elizalde-Velazquez et al. (2020)
PS	Fathead minnows (*Pimephales promelas*)	Adult males	50 nm	Trophic transfer (0.005 mg/L), 48 h	Liver and head kidney	Elizalde-Velazquez et al. (2020)
PS	Chinese rice fish (*Oryzias sinensis*)	Adults and F1 larvae	57.29–60.39 nm	Waterborne (5 mg/L); (adults 7 days; F1 larvae 24 h)	Yolk sac	Chae et al. (2018)
PS	Hainan medaka (*Oryzias curvinotus*)	Adults	80 nm	Waterborne (0.2 mg/L), 7 days	Gills and intestine	Gao et al. (2023a)
PS	Japanese medaka (*Oryzias latipes*)	Adults	100 nm	10, 10^4^, and 10^6^ particles/L (1.79589 × 10^13^ particles/10 mg concentration)	Gut	Zhou et al. (2023b)
PS	Japanese medaka (*Oryzias latipes*)	Adults	100 nm	Waterborne (10, 10^4^, and 10^6^ particles/L) or (5.5 × 10^−12^, 5.5 × 10^−9^, and 5.5 × 10^−7^ mg/L), 3 months	Gonads (ovary/testis)	Zhou et al. (2023a)
PS	Japanese medaka (*Oryzias latipes*)	Larvae (9 dph)	100 nm	Waterborne (10^14^ items/L or 55 mg/L), 48 h	Gut	Zhou et al. (2023b)
PS	Japanese medaka (*Oryzias latipes*)	Adults (60 dph)	100 nm	Waterborne (5.5 × 10^−12^ mg/L, 5.5 × 10^−9^ mg/L, and 5.5 × 10^−7^ mg/L), 90 days	Gut	Zhou et al. (2023b)
PS	Marine medaka (*Oryzias melastigma*)	Embryos	PS (50 nm)	Waterborne (10^6^ particles/L), 19 days	Whole embryo	Chen et al. (2022)
PS	Marine medaka (*Oryzias melastigma*)	Embryos	PS-NH_2_ (80 nm); PS-COOH (80 nm)	Waterborne (0.01 mg/L), 10 days (depurated for 10 days)	Gastrointestinal tract and intestinal villi	Chen et al. (2023a)
PS	Marine medaka (*Oryzias melastigma*)	Embryos (6 hpf)	50 nm	Waterborne (0.055 mg/L), 21 days	Abdominal area/liver/heart	Yu et al. (2023)
PS	Marine medaka (*Oryzias melastigma*)	Larvae (7 dph)	50 nm	Waterborne (0.0025–0.01 mg/L); 1, 7, 14, and 120 dph	Gut	Kang et al. (2021)
PS	Marine medaka (*Oryzias melastigma*)	Larvae (3 dph)	70 nm	Trophic transfer (0.02, 0.2, and 2 mg/L), 90 days	Intestine/liver /muscle/gonad	Li et al. (2024b)
PS	Marine medaka (*Oryzias melastigma*)	Juveniles (2 months)	100 nm	Waterborne (1 mg/L), 30 days	Intestine	Li et al. (2023b)
PS	Marine medaka (*Oryzias melastigma*)	Adults	100 nm	Waterborne (5 mg/g), 30 days	Gut/intestine	Zhang et al. (2021)
PS	Marine medaka (*Oryzias melastigma*)	Adults	100 nm	Dietary (3.45 mg/g), 30 days	Gut/liver of 60 dph F1 larvae	He et al. (2022)
PS	Marine medaka (*Oryzias melastigma*)	Adults (4 months)	100 nm	Dietary (5 mg/g), 30 days (depurated for 21 days)	Gut	Wang et al. (2023a)
PS	Rainbow trout (*Oncorhynchus mykiss*)	Juvenile	35 ± 8 nm	Dietary (0.0059 mg/g food); 3, 7, and 14 days	Hind intestine and liver	Clark et al. (2023b)
PS	Red tilapia (*Oreochromis niloticus)*	Juveniles	100 nm	Waterborne (0.001, 0.01, and 0.1 mg/L), 14 days	Gut, gills, liver, and brain	Ding et al. (2018)
PS	Tilapia (*Oreochromis niloticus*)	Larvae	80 nm	Waterborne (0.1 mg/L), 28 days	Gills	Zheng and Wang (2024)
PS	Tilapia (*Oreochromis niloticus*)	Larvae	80 nm	Waterborne (0.1 mg/L), 28 days	Gills	Zheng et al. (2024)
PS	Tilapia (*Oreochromis niloticus*)	Larvae (4 weeks old)	100 nm	Waterborne (20 mg/L), 7 days (depurated for 7 days)	Whole fish	Pang et al. (2021)
PS	Tilapia (*Oreochromis niloticus*)	Juveniles	86 nm	Waterborne (1 mg/L), 21 days (depurated 7 days)	Gill, stomach, intestine, liver, and muscle	Hao et al. (2023)
PS	Tilapia (*Oreochromis niloticus*)	Juveniles	100 nm	Waterborne (1, 10, and 100 mg/L), 7 days	Gill, liver, intestine, and muscle	Wang et al. (2023b)
PS	Zebrafish (*Danio rerio*)	Embryos (3 hpf)	47 nm	Waterborne (1 mg/L), 120 h	Whole embryo	Chen et al. (2017a)
PS	Zebrafish (*Danio rerio*)	Embryos	25 and 50 nm	Waterborne (25 mg/L; 25 nm) (50 mg/L; 50 nm); 0–48 hpf, 24–72 hpf, and 72–120 hpf	Chorion (0 hpf); eye (72 hpf)	Van Pomeren et al. (2017)
PS	Zebrafish (*Danio rerio*)	Embryos (6 hpf)	51 nm	Waterborne (0.1, 1, and 10 mg/L), 120 hpf	Yolk sac, GI tract, gall bladder, liver, pancreas, heart, and brain	Pitt et al. (2018a)
PS	Zebrafish (*Danio rerio*)	Embryos (72 hpf)	25 nm	Waterborne (20 mg/L), 72–120 hpf, 48 h	Intestine, pancreas, and gall bladder	Brun et al. (2019)
PS	Zebrafish (*Danio rerio*)	Embryos	50 nm	Waterborne (0.1 mg/L); 6, 24, and 96 hpf	Whole body	Lee et al. (2019)
PS	Zebrafish (*Danio rerio*)	Embryos (6 hpf)	44 nm	Waterborne (0.1, 1, and 10 mg/L), 96 hpf	Whole body	Trevisan et al. (2019)
PS	Zebrafish (*Danio rerio*)	Embryos (6 hpf)	50 nm	Waterborne (0.01, 0.1, 1, and 10 mg/L), 120 hpf	GI tract, eye, liver, and cranial region	Pedersen et al. (2020)
PS	Zebrafish (*Danio rerio*)	Embryos	20 nm	Microinjected to eggs (3 µL of 270 mg/L), 120 hpf	Brain	Sokmen et al. (2020)
PS	Zebrafish (*Danio rerio*)	Embryos	44 nm	Waterborne (1 mg/L), 7 days	Yolk sac and brain	Trevisan et al. (2020)
PS	Zebrafish (*Danio rerio*)	Embryos	70 ± 9.21 nm	Microinjected to eggs (0.52 nL of 1,000, 3,000, and 5,000 mg/L), 4 weeks	Maximum in the yolk sac and followed by brain > eyes > gut > swim bladder (maximum accumulation in the trunk region	Zhang et al. (2020)
PS	Zebrafish (*Danio rerio*)	Embryos	70 ± 9.21 nm	Waterborne (0.5 and 5 mg/L), exposed until hatching and depurated for 4 weeks	Maximum accumulation in the brain and eyes	Zhang et al. (2020)
PS	Zebrafish (*Danio rerio*)	Embryos	60 nm	Waterborne (0.015, 1.5, and 150 mg/L, 96 h	Whole embryos	Barreto et al. (2021)
PS	Zebrafish (*Danio rerio*)	Embryos (2 hpf)	100 nm	Waterborne (0.01 mg/L); 12 h (depurated 120 hpf)	Whole embryos	Liu et al. (2021)
PS	Zebrafish (*Danio rerio*)	Embryos	50 nm	Waterborne 1 mg/L (96 h)	Whole embryo	Bhagat et al. (2022)
PS	Zebrafish (*Danio rerio*)	Embryos	100 nm	Waterborne (0.0025 and 0.025 mg/L) 7 days	Anterior part containing the yolk sac and digestive tract	Chackal et al. (2022)
PS	Zebrafish (*Danio rerio*)	Embryos	50 and 100 nm	Waterborne ((0.1, 0.5, 2 and 10 mg/L), 120 hpf	Intestine and areas of excretion	Cheng et al. (2022)
PS	Zebrafish (*Danio rerio*)	Embryos	100 nm	Waterborne (100, 200, and 400 mg/L), 96 h	Whole embryo	Feng et al. (2022)
PS	Zebrafish (*Danio rerio*)	Embryos	50 nm	Waterborne (5 mg/L), 4–96 hpf	Surface of the chorion and the embryos	Geum and Yeo, (2022)
PS	Zebrafish (*Danio rerio*)	Embryos	25 nm	Waterborne (10, 25, and 50 mg/L), 96 hpf	Whole embryo	Kantha et al. (2022)
PS	Zebrafish (*Danio rerio*)	Embryos (2 hpf)	100 nm	Waterborne (0.01 mg/L) (144 hpf, depurated for 3 days)	Whole embryo	Liu et al. (2022b)
PS	Zebrafish (*Danio rerio*)	Embryos	22 nm	Waterborne (0.001, 0.01, 0.1, 1, 10, and 100 mg/L), 96 hpf	Whole embryo	Manuel et al. (2022)
PS	Zebrafish (*Danio rerio*)	Embryos	50 nm	Waterborne (0.000069, 0.00069, 0.069, 0.687, and 6.87 mg/L), 120 hpf	Chorion, eye, tail, and yolk sac	Martinez-Alvarez et al. (2022)
PS	Zebrafish (*Danio rerio*)	Embryos	44 nm	Waterborne (0.015, 0.15, 1.5, 15, and 150 mg/L), 96–120 hpf	Whole embryo	Santos et al. (2022)
PS	Zebrafish (*Danio rerio*)	Embryos (4 hpf)	20 nm	Injected (3 nL of 270 mg/L); grown for 6 months; F1 embryos were evaluated	Whole embryo	Sulukan et al. (2022a)
PS	Zebrafish (*Danio rerio*)	Embryos	PS-NH_2_ (50 nm fluorescent) PS-COOH (30 nm fluorescent) PS-NH_2_ (51 nm, unlabeled) (+ve charge) PS-COOH (50 nm unlabeled) (-ve charge)	Waterborne (30 and 50 mg/L to labeled or unlabeled PS-NH_2_ or PS-COOH), 120 hpf	GI tract, pericardium, and brain	Teng et al. (2022a)
PS	Zebrafish (*Danio rerio*)	Embryos	80 nm	Waterborne (0.05 mg/L, 0.1 mg/L, 1 mg/L, 5 mg/L, and 10 mg/L) (120 hpf)	Surface of the chorion, brain, gills, mouth, trunk, heart, liver, and digestive tract	Wang et al. (2022)
PS	Zebrafish (*Danio rerio*)	Embryos	44 nm	Waterborne (0.015 and 1.5 mg/L), 96–120 h	Whole embryo	Barreto et al. (2023)
PS	Zebrafish (*Danio rerio*)	Embryos (8 hpf)	80 nm	Waterborne (0.5 and 5 mg/L), 96 hpf	Yolk sac, eye, head, and nerve tubes	Chen et al. (2023b)
PS	Zebrafish (*Danio rerio*)	Embryos	50 nm	Waterborne (0.1, 1, 5, 10, 20, 30, and 50 mg/L), 5 days	Whole embryo	Chen et al. (2023c)
PS	Zebrafish (*Danio rerio*)	Embryos	20 nm	Waterborne (2, 5, and 8 mg/L); 22, 46, and 70 h	Whole embryo	Dai et al. (2023)
PS	Zebrafish (*Danio rerio*)	Embryos	50 nm	Waterborne (0.1, 0.5, and 1 mg/L); 4–72 h at 24°C, 27°C, and 30°C	Chorion, abdomen, circulatory system, intestinal tract, and excretory regions	Duan et al. (2023)
PS	Zebrafish (*Danio rerio*)	Embryos (3 hpf)	80 nm	Waterborne (0.005, 0.01, 0.025, 0.05, and 0.1 mg/L), 96 h	Whole embryo	Gao et al. (2023b)
PS	Zebrafish (*Danio rerio*)	Embryos	30 and 100 nm	Waterborne (0.1, 1, and 10 mg/L), 96 h	Chorion, head, trunk, and in the yolk	Martin et al. (2023)
PS	Zebrafish (*Danio rerio*)	Embryos	30 nm	Waterborne (0.1, 0.5, and 3 mg/L), 120 hpf	Whole embryo	Martin-Folgar et al. (2023)
PS	Zebrafish (*Danio rerio*)	Embryos (4 hpf)	PS (91, nm)	Waterborne (0.001, 0.01, 0.1, 1, 10, and 10 mg/L), 96 hpf	Vitelline membrane	Tamayo-Belda et al. (2023)
PS	Zebrafish (*Danio rerio*)	Embryos (2 hpf)	15 nm	Waterborne (50 mg/L), 96 h	GI tract, pericardium, eye, and cranial regions	Varshney et al. (2023)
PS	Zebrafish (*Danio rerio*)	Embryos	80 nm	Waterborne (0.05, 0.1, 1, 5, and 10 mg/L), 120 hpf	Gills, GI, liver, and heart	Wang et al. (2023c)
PS	Zebrafish (*Danio rerio*)	Embryos (4 hpf)	50 nm	Waterborne (1, 5, and 10 mg/L), 144 hpf	Whole embryo	Wang et al. (2023d)
PS	Zebrafish (*Danio rerio*)	Embryos (4 hpf)	100 nm	Waterborne (10 mg/L), 5 days	Chorion, brain, yolk sac, muscle, GI tract, pancreas, gall bladder, liver, and swim bladder	Zhou et al. (2023c)
PS	Zebrafish (*Danio rerio*)	Embryos (8 hpf)	80 nm	Waterborne (0.1, 0.5, 1, 5, 10, 25, and 50 mg/L); 120 hpf; some were depurated for 10 days	Chorion, eye, brain, and dorsal trunk	Chen et al. (2024)
PS	Zebrafish (*Danio rerio*)	Embryos	PS (23.03 ± 0.266 nm)	Waterborne (0.00000004 mg/L, 0.000034 mg/L, and 0.034 mg/L), 144 hpf	In embryos, accumulation occurred in the chorion, muscle, gills, and head of the fish; in larvae, accumulation occurred in the digestive system, gills, and somite	Santos et al. (2024)
PS	Zebrafish (*Danio rerio*)	Larvae (120 hpf)	50 nm	Waterborne (10 mg/L), 24 h–7 days	Gut, skin, caudal fin, and eyes	Sendra et al. (2021)
PS	Zebrafish (*Danio rerio*)	Adults (6 months old)	47 nm	Waterborne 1 mg/L (3 days)	Viscera, gills, head, and muscle	Chen et al. (2017b)
PS	Zebrafish (*Danio rerio*)	Adults	42 nm	Dietary (1 mg/L); 7 days; F1 larvae were evaluated	Yolk sac, GI tract, liver, pancreas, and gall bladder	Pitt et al. (2018b)
PS	Zebrafish (*Danio rerio*)	Adults (6 months old)	70 nm	Waterborne (0.5, 1.5, and 5 mg/L); 7 days, 30 days, and 7 weeks	Gonads, intestine, liver, and brain tissues (observed after 30 days of exposure)	Sarasamma et al. (2020)
PS	Zebrafish (*Danio rerio*)	Adults (male and female)	46 nm	Waterborne (2 mg/L), 21 days	Gonads	He et al. (2021)
PS	Zebrafish (*Danio rerio*)	Adults (male and female)	70 nm	Waterborne (0.1 mg/L), 45 days; F1 embryos were evaluated	Whole embryos (F1)	Wu et al. (2021)
PS	Zebrafish (*Danio rerio*)	Adults	80 nm	Waterborne (1 mg/L), 21 days	Gut	Xie et al. (2021)
PS	Zebrafish (*Danio rerio*)	Adults (90 days old)	54.5 ± 2.8 nm	Waterborne (10 mg/L), 120 days; both F0 parents and F1 embryos were evaluated	F0 = gut > gills > gonad > liver F1 = whole embryo/larvae	Zhao et al. (2021)
PS	Zebrafish (*Danio rerio*)	Adults (90 days old)	70 nm	Waterborne (0.1 mg/L), 21 days; F1 larvae were evaluated at 120 hpf	Testis and ovary (F1 larvae)	Zuo et al. (2021)
PS	Zebrafish (*Danio rerio*)	Adults (male and female)	70 nm	Waterborne (0.1 mg/L), 3 months	Liver	Ling et al. (2022)
PS	Zebrafish (*Danio rerio*)	Adults (3 months old)	100 nm	Waterborne (25 mg/L); 96 h at 28°C, 29°C, and 30°C	Brain	Sulukan et al. (2022b)
PS	Zebrafish (*Danio rerio*)	Juveniles and adults	44 nm	Waterborne (0.001, 0.01, and 0.1 mg/L); 30 and 60 days	Gut–brain axis	Teng et al. (2022b)
PS	Zebrafish (*Danio rerio*)	Adults	100 nm	Waterborne (0.02 and 0.2 mg/L), 3 weeks	Intestine	Yu et al. (2022a)
PS	Zebrafish (*Danio rerio*)	Adults	40–54 nm	Waterborne (0.06–0.186 mg/L), 30 days	Intestine	Yu et al. (2022b)
PS	Zebrafish (*Danio rerio*)	Adults	20–80 nm	Waterborne (0.0001, 0.001, 0.01, and 0.1 mg/L), 45 days	Brain	Aliakbarzadeh et al. (2023)
PS	Zebrafish (*Danio rerio*)	Adults	100 nm	Waterborne (0.5 mg/L0), 28 days	Liver	Deng et al. (2023)
PS	Zebrafish (*Danio rerio*)	Adults (male, 4 months old)	50 nm	Waterborne (5, 10, and 15 mg/L), exposed for 30 days and depurated for 16 days	Intestine > liver > gill> muscle > brain	Habumugisha et al. (2023)
PS	Zebrafish (*Danio rerio*)	Adults	80 nm	Waterborne (15 and 150 mg/L) (21 days)	Liver	Li et al. (2023a)
PS	Zebrafish (*Danio rerio*)	Adults (5 months old; male and female)	70 nm	Waterborne (2 mg/L), 21 days	Gonads (testis and ovary)	Lin et al. (2023)
PS	Zebrafish (*Danio rerio*)	Adults	100 nm	Waterborne (0.1 and 1 mg/L); 4 days (depurated for 3 days)	Gut	Yang et al. (2023)
PS	Zebrafish (*Danio rerio*)	Adults	100 nm	Waterborne (1 mg/L), 30 days	Brain	Zhang et al. (2023)
PS	Zebrafish (*Danio rerio*)	Adults	(50 ± 3 nm)	Waterborne (1 mg/L), 4 weeks	Brain	Zhou et al. (2023d)
PS	Zebrafish (*Danio rerio*)	Adults	50–100 nm	Waterborne/dietary exposure to a high-fat diet (21 days)	Gut	Du et al. (2024)
PS	Zebrafish (*Danio rerio*)	Adults	50 nm	Waterborne (1 mg/L), 21 days	Liver, brain, and gonads (testis and ovary)	Ye et al. (2024)
PS	Zebrafish (*Danio rerio*)	Adults	100 nm	Waterborne (1 mg/L), 30 days	Blood, intestine, and brain	Zhang et al. (2024c)

Elizalde-Velazquez et al. (2020) used two different methods of exposure (injection and trophic transfer) of PS in fathead minnows and mentioned it in one article. Manuel et al. (2022) reported the effects of PMMA, and PS in zebrafish in one article. Monikh et al. (2022) reported the effects of PE, and PPP in one article in zebrafish. Tamayo-Belda et al. (2023) reported the effects of PS, and LDPE in one article in zebrafish. Zhang et al. (2020) used two different methods of exposure (injection and waterborne) of PS in zebrafish and mentioned in one article.

**TABLE 6:** Effects of NAPs on fish targeting toxicological endpoints.

Fish	Plastic polymers	Developmental stage	Observed effects	References
Common carp (*Cyprinus carpio*)	PE	Juvenile	1. The AChE and MAO activities and the NO concentration decreased significantly 2. Varying degrees of necrosis, fibrosis, changes in blood capillaries, tissue detachment, edema, degenerated connective tissue, and necrosis of large cerebellar neurons and ganglion cells were observed in the tectum (brain) 3. Induced necrosis, degeneration, vacuolation, and curvature in the inner layer of the retina	Hamed et al. (2022)
Carp	PS	Adults	1. Induced myocardial injury 2. Induced apoptosis in the myocytes 3. Increase in protein contents of TLR4 and NOX2 4. Promoted the levels of H_2_O_2_ and MDA and inhibited the antioxidant capacity (CAT, SOD, and GPx enzymatic activity and GSH and T-AOC content) in the myocardial tissue	Wu et al. (2022)
Grass carp (*Ctenopharyngodon idella*)	PS	Embryos	1. Accumulated on the surface of the chorion 2. No embryo mortality 3. No difference in embryonic heart rates	Zhang et al. (2022b)
Grass carp (*Ctenopharyngodon idella*)	PS	Juveniles	1. HSI enhanced 2. No effect on locomotor activities 3. Increased AChE activity and LPO content in the brain; no change in nitrate production 4. Stimulated the antioxidant activity of the brain and intestine (increase in GSH and MDA contents; SOD, CAT, and GST activities; and diphenyl-1-pycrilhydrazil [DPPH] radical scavenging activity 5. No effect on NO production in the brain 6. Induced DNA damage in erythrocytes 7. Induced lesions in the gills and intestine 8. A concentration-dependent histological damage (increase in vacuoles) of the gut	Estrela et al. (2021), Guimaraes et al. (2021), Liu et al. (2022a), Li et al. (2024a)
Silver carp (*Hypophthalmichthys molitrix*)	PS	Adults	1. Increase in the hepatocyte space 2. The diversity and richness in gut microbiota are increased 3. Imbalance induced in glycerophospholipid metabolism	Zhang et al. (2024a)
Tooth carp (*Aphaniops hormuzensis*)	PS	Adults	1. The 96-h LC_50_ for PS is 19.3 mg/L 2. Accumulated in the gut, gill, liver, muscle, and skin after 28 days of dietary exposure	Saemi-Komsari et al. (2023)
Fathead minnows (*Pimephales promelas*)	PS	Adult (male)	1. Immunomodulatory effects on the liver and head kidney	Elizalde-Velazquez et al. (2020)
Chinese rice fish (*Oryzias sinensis*)	PS	Adults and F1 embryos	1. Locomotive activities were affected	Chae et al. (2018)
Hainan medaka (*Oryzias curvinotus*)	PS	Adults	1. Fusion of the gill lamellae 2. Appearance of eosinophilic vesicles and vacuolization in the liver 3. Erosion of intestinal villi 4. No effect was observed on the MDA content and SOD activity in the gills and muscle, while CAT activity decreased in the gills and increased in the muscle 5. SOD and CAT activities remained unaltered in the liver and intestine 6. Disrupted gut microbial community	Gao et al. (2023a)
Japanese medaka (*Oryzias latipes*)	PS	Adult	1. Concentration-dependent mortality, with no effect on body length, body mass, and eye diameter 2. In the gut, widening of the lamina propria, shortening and swelling of villi, edema, fusion, and cracking of villi are observed 3. The lipase and chymotrypsin activities in the gut were significantly higher; however, trypsin activity increased at lower concentrations, while it decreased at higher concentrations 4. The SOD and alkaline phosphatase activities and d-lactate content reduced in the gut, while the CAT, lysozyme, and diamine oxidase activities and MDA content increased 5. Disrupted gut microbial community 6. In the testis and ovary, a concentration-dependent decrease was observed in the enzymatic activities of CAT and GPx and in LZM and MDA contents, while SOD activity was increased in the testis and decreased in the ovary 7. Concentration-dependent inhibition in spermatogenesis (mature sperms were slightly decreased) and oogenesis (increase in primary oocytes and decrease in mature spawning follicles)	Zhou et al. (2023a), Zhou et al. (2023b)
Marine medaka (*Oryzias melastigma*)	PS, PS-NH2, and PS-COOH	Embryos	1. Concentration-dependent effects on mortality 2. Delayed hatching 3. Increased cardiac rates 4. Induced morphological abnormalities (craniofacial deformities, yolk sac edema, fin deformities, spinal deformity, pericardial edema, cardiac stretch hemorrhaging, spinal curvature, and fin deformities) 5. Liver histopathology indicates inflammatory responses (vacuolation, apoptosis, and necrosis) 6. Induced myocardial wall thinning and reduced myocardial fiber and irregularity in cardiac morphology 7. Disruption of swimming velocity	Chen et al. (2023a), Yu et al. (2023)
Marine medaka (*Oryzias melastigma*)	PS	Larvae	1. No effect on the body length, weight, condition factor, and eye diameter 2. Increased diamine oxidase activity in the gut 3. HSI increased and GSI decreased in male and female fish 4. Hepatocyte vacuolation, hyaline degeneration, and lipid accumulation in the liver 5. Increased SOD, CAT, and GST activities in the gut and liver tissues, while the ROS levels decreased in the gut and increased in the liver 6. Hepatic protein, sugar, glycogen, and lactate contents were reduced, and triglyceride (TG) contents were increased in a concentration-dependent manner 7. The fiber density and diameter in the muscle were decreased in a concentration-dependent manner; however, TG and lactate contents in the muscle increased and the total sugar and glycogen contents decreased 8. Fecundity reduced, and no alterations in the fertilization rate were observed 9. Disrupted gut microbial community	Kang et al. (2021), Li et al. (2024b)
Marine medaka (*Oryzias melastigma*)	PS	Juveniles	1. Volume of the intestinal mucus tended to increase 2. Decrease in goblet cell numbers 3. Disrupted gut microbial community	Li et al. (2023b)
Marine medaka (*Oryzias melastigma*)	PS	Adult	1. No significant effects on the mortality, deformities, weight, and condition factors 2. Bodyweight reduced in F1 offspring 3. Disrupted gut microbial community	Zhang et al. (2021); He et al. (2022), Wang et al. (2023a); Zhang et al. (2024b)
Rainbow trout (*Oncorhynchus mykiss*)	PS	Juveniles	1. Accumulation occurred in the hind intestine and then the particles transported to the liver	Clark et al. (2023b)
Tilapia (*Oreochromis niloticus*)	PPP	Juveniles	1. No effect on the HSI 2. Significant effects on glycerophospholipid, arginine, and proline metabolism and aminoacyl-tRNA biosynthesis	Wu et al. (2023)
Tilapia (*Oreochromis niloticus*)	PS	Larvae	1. In gills, the number of fibroblasts, macrophages, natural killer cells, and B-cells reduced, while the number of H^+^ATPase-rich cells increased 2. Chromatin marginalization and apoptosis induced in gill cells	Zheng and Wang (2024), Zheng et al. (2024)
Tilapia (*Oreochromis niloticus*)	PS	Juveniles	1. No observed mortality 2. Passes through the intestinal wall and is delivered to other tissues 3. In the intestine, mucosal layer thinning was observed, epithelial cells were disordered, submucosal cells induced edema, and eosinophilic infiltrations were observed 4. Diamine oxidase activity and d-lactate content of the intestinal wall increased 5. The SOD and GPx activities and the MDA content in the gut increased; while in the liver, the GSH content remained unaltered, MDA content increased, and SOD activity reduced 6. Induced hepatic steatosis; the EROD (cyp1a) and BFCOD (cyp3a) activities were altered in a nonlinear fashion 7. AChE activities in the brain were reduced 8. Induced dysbiosis in gut microbial communities	Ding et al. (2018), Ding et al. (2020); Hao et al. (2023); Wang et al. (2023b)
Zebrafish (*Danio rerio*)	LDPE	Embryos	1. No significant effect on heart rates; during the light phase, slight effects on larval movement were observed	Tamayo-Belda et al. (2023)
Zebrafish (*Danio rerio*)	PE	Embryos	1. Delayed hatching 2. Reduced larval body length	Monikh et al. (2022)
Zebrafish (*Danio rerio*)	PE	Adults	1. No mortality observed 2. In gills, GSH content and SOD activity remained unaltered, while CAT activity and LPO increased 3. In the intestine/gut, GSH content and GST activity were increased, LPO levels decreased, CAT activity remained unaltered, and SOD activity showed inconsistent alterations 4. In the liver, GST activity increased and SOD activity and LPO levels showed inconsistent alterations 5. The AChE activity in the gill and gut showed inconsistent alterations, while in the liver, AChE activity tended to reduce after initial exposure 6. Disruption of gut microbial community	Khan and Ali (2023); Li et al. (2023c)
Zebrafish (*Danio rerio*)	PET	Embryos	1. Concentration-dependent reduction in hatching with enhanced mortality and heartrates 2. Diminished spontaneous tail coiling 3. Reduced interocular distance without affecting the body length 4. Reduced locomotor activity in the dark 5. Impairment of mitochondrial membrane integrity 6. No significant change in LPO levels and total antioxidant capacity	Bashirova et al. (2023); De Souza Toedoro et al. (2024)
Zebrafish (*Danio rerio*)	PMMA	Embryos	1. Concentration-dependent mortality, delayed hatching, and pericardial edema 2. No significant effects on swimming behavior 3. Nonlinear increase in GPx activity, inconsistent effects on LPO content and CAT activity, and no effect on GST activity 4. AChE activity did not show any significant changes	Manuel et al. (2022)
Zebrafish (*Danio rerio*)	PPP	Embryos	1. Failed to develop normal morphology 2. Delayed hatching and curved spine and reduced larval body length were observed	Monikh et al. (2022)
Zebrafish (*Danio rerio*)	PS, PS-NH_2,_ and PS-COOH	Embryos	1. Depending on the exposure routes, inconsistent effects on survivability, malformation rates (pericardial edema, inhibition of myocardial diastolic functions, curved spine, scoliosis, and uninflated swim bladder), and hatching rates were observed; however, heart rates and larval body length tended to reduce 2. The development of neurons and motor neurons in the brain of zebrafish (72 hpf) was interrupted, and there was a significant reduction in the touch response 3. Positively charged PS (PS-NH_2_) induced stronger developmental toxicity than negatively charged PS (PS-COOH) 4. Uninflated swim bladder (concentration-dependent) 5. Decline in the HR (H^+^-ATPase) and NaK (Na^+^ K^+^-ATPase) cell (ionocytes) densities and active ionocytes in the skin cells 6. The total length of microridges on the skin keratinocytes significantly reduced, and the distance between myosepta was found to be smaller 7. Disruptions observed in the sprouting of intersegmental vessels and small vessels (nasal vessels, dorsal vessels, and ventral vessels) and promotes vasculogenesis (increasing the number and length of extrinsic branches of the sub-intestinal venous plexus) 8. Induced overgrowth of the common cardinal vein (CCV) and endothelial cells in CCV. 9. Elicited complex effects on locomotor behavior 10. The whole-body contents of Na^+^, K^+^, and Ca^2+^ of the embryos and H^+^ and NH_4_^+^ secretion of the skin declined 11. No effect on GPx and CAT activity (on a few occasions increased), SOD activity inconsistent, and GR activity decreased; GSH content decreased/unaltered; and MDA content remained unaltered. [The oxidative stress index (based on CAT, peroxidase, and SOD activities and GSH and MDA contents) significantly increased] 12. ROS content was enhanced, and apoptosis and ferroptosis (cell death due to iron accumulation) were induced 13. Significantly increased NO content and decreased the activities of soluble guanylate cyclase (sGC) and protein kinase G (PKG) enzymes 14. Induced disorders in amino acid metabolism including valine, leucine, and isoleucine biosynthesis and β-alanine, aspartate, and glutamate metabolism 15. Neutrophil population increased and macrophage population decreased on the abdominal area of the larvae 16. Significant decrease in neutral lipid storage and increase in oxygen concentration rates were observed 17. Cortisol and glycogen concentrations increased 18. AChE activity mostly decreased 19. Decrease in the mitochondrial coupling efficiency and inconsistencies in the NADH level were observed 20. There was no change in metallothionine (MT) (mt2) expression 21. Induced DNA damage in the brain	Chen et al. (2017a); Van Pomeren et al. (2017); Pitt et al. (2018a); Brun et al. (2019); Lee et al. (2019); Trevisan et al. (2019), Trevisan et al. (2020); Pedersen et al. (2020); Sokmen et al. (2020), Zhang et al. (2020), Barreto et al. (2021), Liu et al. (2021); Bhagat et al. (2022); Chackal et al. (2022); Cheng et al. (2022); Feng et al. (2022); Geum and Yeo, (2022); Kantha et al. (2022); Liu et al. (2022b); Manuel et al. (2022); Martinez-Alvarez et al. (2022); Santos et al. (2022), Teng et al. (2022a); Wang et al. (2022); Barreto et al. (2023); Chen et al. (2023b); Chen et al. (2023c); Dai et al. (2023); Duan et al. (2023); Gao et al. (2023b); Martin et al. (2023); Martin-Folgar et al. (2023); Tamayo-Belda et al. (2023); Varshney et al. (2023); Wang et al. (2023d), Wang et al. (2023e); Zhou et al. (2023c); Chen et al. (2024); Santos et al. (2024)
Zebrafish (*Danio rerio*)	PS	Larvae	1. No mortality was observed 2. The number of neutrophils and macrophages increased in the gut and caudal fin 3. ROS content (stomach and gut) was increased	Sendra et al. (2021)
Zebrafish (*Danio rerio*)	PS	Adult	1. Inhibited AChE activity (inconsistent) 2. No effect on fecundity (total number of eggs laid), reduced fecundity, spawning events, fertilization, and hatchability of the embryos 3. Induced oxidative stress a) GR activity was lower in the brain and muscle of females and muscle and testis of males b) GPx activity was elevated only in the brain of females, while CAT activity remained unaltered (reduced) c) In the liver, no effects on the ROS (increased in the brain) and MDA (increased in the intestine and liver) contents and the GST and CAT (reduced in liver) activities were observed. GSH activity decreased in the liver 4. The oxygen consumption rate (OCR) in the heart and testis remained unaltered, while it was enhanced in the ovary 5. Significant expansion of the villi structure of the intestinal tissue; increased mucus secretion, and decreased LZM activity 6. Disruption of gut microbial community 7. Liver and intestine: a) inconsistent effects on HSI b) No significant effects on VTG contents of male or female (reduced) fish c) Creating a large number of vacuoles and lipid droplets in the liver cells d) Changed the lipid molecular contents related to cell membrane function and lipid biosynthesis e) MAO (the catalytic enzyme of 5-HT) and the mRNA level of MAO in the intestine tended to decrease 8. Brain, a) the histology indicated that fish exposed to PSNAP showed damage in the neuronal layers as well as reduction in the neuronal cell number b) a small amount of micro thrombosis consisting of aggregated and dissolved red blood cells and the mitochondria with a damaged membrane and loss of cristae c) the mitochondrial DNA copy number was significantly reduced d) MAO activity decreased; AChE activity and dopamine, melatonin, GABA, serotonin (5-HT), vasopressin, kisspeptin, oxytocin, glutamine synthase (GS), and αKGPD activity/contents were significantly decreased e) no effect was observed in the acetylcholine level f) changes in the brain metabolites, including 3,4-dihydroxyphenylacetic acid and l-glutamine, occurred g) the glutamate dehydrogenase (GDH) activity was enhanced h) the β-galactosidase and lipofuscin levels (aging markers) are significantly higher in the brain of zebrafish (both males and female) exposed to PSNAP. i) temperature-dependent degenerative necrotic changes in the medulla oblongata, medial longitudinal fascicle, lateral valvula nucleus, and thalamus regions were observed j) the γ-H2AX levels, 8-hydroxydeoxyguanosine (8-OHdG), and MDA contents were significantly higher in the brain of male and female fish exposed to PSNAP. k) ATP and cyclin-dependent kinase levels were significantly lower and p53 levels were significantly higher in the brains of male and female zebrafish 9. Gonads (a) inconsistent effect on GSI (no change/decreased) (b) decrease in sperm content, and lacunae and interstitial cells were observed in the testis (c) no effect on ovaries (d) did not affect E2 or T contents in female and male fish (decreased E2 and T) (e) increased the number of spermatogonia and spermatocytes in the testis; moreover, deformation of seminiferous tubules was observed (f) showed more preovulatory oocytes and smaller mature oocytes. Unable to alter the amount of PO, LVO, CAO, and EVO in the ovary (g) no significant effects on the amount of spermatogonia, spermatocytes, spermatocytes, spermatocytes, spermatids, and spermatozoa (percent) were observed (h) did not exhibit any effects on the LH contents in the ovary; serum E2 and testis E2 levels and GnRH and FSH contents remained unaltered; the LH levels in the testis were significantly reduced by PSNAP exposure; significant effects on the T3 and T4 levels were observed in both male and female fish 10. Behavioral alteration in the locomotor activity is temperature-dependent 11. No effect on fertilization rates and hatching rates 12. Intergenerational: a) due to parental exposure, accumulation of PSNAP was observed in different organs of F1 (yolk sac, GI tract, liver, pancreas, and gall bladder); however, no developmental defect in F1 larvae was observed b) parental exposure, did not alter T3 and T4 levels in F0 fish as well as in F1 larvae; however, T4 levels were reduced in eggs c) bradycardia in heart, with reduced GR activity (F1) d) spontaneous movements of the embryos, the heart beats, hatching rates, and the length of the F1 larvae were affected (spinal curvature, pericardial cyst, and growth retardation).	Chen et al. (2017b); Pitt et al. (2018b); Sarasamma et al. (2020); He et al. (2021); Wu et al. (2021); Xie et al. (2021); Zhao et al. (2021); Zuo et al. (2021); Ling et al. (2022); Sulukan et al. (2022a); Teng et al. (2022b); Yu et al. (2022a); Yu et al. (2022b); Aliakbarzadeh et al. (2023); Deng et al. (2023); Habumugisha et al. (2023); Li et al. (2023a); Lin et al. (2023); Yang et al. (2023); Zhang et al. (2023); Zhang et al. (2024c); Zhou et al. (2023d); Du et al. (2024); Ye et al. (2024)

**TABLE 7:** Genotoxic effects of NAPs on fish.

Polymer (name, method of application, and duration)	Fish (name and developmental stage)	Organ and gene types	Upregulated	Downregulated	Unchanged	References
PS (80 nm) (10, 100, and 1,000 μg/L; waterborne, 8 days)	Grass carp (juveniles)	Gut/intestine	In intestine *IL-6, IL-8, IL-10, IL-1β, TNF-α,* and *INF-γ2*			Li et al. (2024a)
PS (50 nm) (IP injected) (0.1 mL of 5 μg/L injected volume) exposed for 48 h	Fathead minnows (adult male)	Liver and head kidney		1. In the liver, macrophage-stimulating 1 (*mst1*) and complement component 3 (*c3*) genes 2. In the head kidney, *ncf* and *mst1* genes	1. Cytosolic factor 2 (*ncf2*) and NADPH oxidase 2 (*nox2*) genes in the liver 2. In head kidney, *nox2* and *c3* genes	Elizalde-Velazquez et al. (2020)
PS (50 nm) (trophic transfer) (48 h)	Fathead minnows (adult male)	Liver and kidney	1. Upregulation of *mst1* and *c3* genes in the liver	1. In the liver, neutrophil cytosolic factor 2 (*ncf2*) expression was downregulated 2. In the head kidney, *ncf* and *c3* genes	1. In the liver, no effects on the *nox2* gene were observed 2. In head kidney, nox*2* and *mst1* genes	Elizalde-Velazquez et al. (2020)
PS (100 nm) [(5 mg/g); dietary, 30 days]	Marine medaka (adults)	Gut			1. In male and female fish*, sod*, *cat,* and *gpx* genes	Zhang et al. (2021)
PS (100 nm) (3.5 mg/g; dietary for 30 days) Parental exposure (F0); F1 was not exposed (observed after 60 dpf)	Marine medaka (adults)	Liver	1. In the liver of male fish (F1), *sod* expression was upregulated	1. In F1 males, the expression of *igf1* in the liver was reduced	1. In F1 female, *igf1* in the liver remained unaltered 2. Expression of *gpx* in the liver (F1 females) remained unaltered 3. In the liver of (F1) male fish, the expressions of *cat* and *gpx* remained unaltered	He et al. (2022)
PS (70 nm) (20, 200, and 2000 μg/L trophic transfer, 90 days)	Marine medaka (adults)	Intestine, liver, muscle, and F1 offspring	1. The expressions of *il6, il8, il1b, il10,* and *tnf* genes in the intestine 2. The expression of inflammatory factor-related genes (*il6, il8, il1b,* and *tnf*) in liver 3. The expression of lipid synthesis-related genes (*fasn, srebf1*, and *pparg*) in the liver 4. The expression of lipid transport-related genes (*cetp*, and *ldlr*) in the liver 5. Genes of the Toll-like receptor 4 (TLR4) pathways (*irf3, irak4, traf6,* and *tbk1*) in the liver	1. The expression of lipid degradation-related genes (*atg1, ppara*, and *aco)* in the liver 2. Muscle development-related gene (*myog, myod, mstn, myf5,* and *fgf6b*) expressions were downregulated 3. Cardiac development-related genes (*bmp4, nkx2.5, cox, epo,* and *smyd1*) genes during embryo–larval development		Li et al. (2024b)
PS (100 nm) (20 mg/L, waterborne); exposed for 7 days and depurated for 7 days	Mozambique tilapia (larvae) (4 weeks old) (0.57 ± 0.13 g body weight)	Whole fish	1. *ptgdsl2, pla2g7, cad*, and *odc1* maintained their expression during exposure and had upregulated expression during recovery	1. Cell adhesion molecules (*cam, ncam2, cntn2,* and *nlg1*) 2. Neuroactive ligand–receptor activation (*grin2a, grin2b, gabrb2,* and *gabra2*) 3. Decreased during exposure and recovered to normal levels during the depuration period (*ncam2, p2rx3, gad1,* and *gad2*) 4. *col1a1* and *col1a2* maintained their expression during exposure and had downregulated expression during the depuration period		Pang et al. (2021)
PS (86 nm) (1 mg/L, (waterborne, exposed for 21 days and depurated for 7 days)	Nile tilapia (juveniles) (10.9 ± 3.9 g body weight)	Gut/intestine	1. *tnfα, il1β*, and *il8* (intestine)	1. *il10* (intestine)		Hao et al. (2023)
PS (100 nm) (waterborne, 1, 10, and 100 μg/L for 7 days)	Nile tilapia (juvenile body weight 15 ± 5 g)	Liver	1. *tnfα* and *il1b* 2. eukaryotic translation initiation factor 2a (*eif2a*), activating transcription factor 4a (*atf4a*), and C/EBP homologous protein (*chop*) 3. Nuclear factor erythroid 2-related factor (*nrf2*) and kelch-like ECH-associated protein 1 (*keap1*)	1. *cyp1a* and *cyp3a* 2. Calreticulin (*calr)* and glucose-regulated protein (*hspa5)*		Wang et al. (2023b)
PS (47 nm) (1 mg/L, 120 h waterborne)	Zebrafish (embryos)	Whole larvae	1. *gfap* and *α1-tubulin* mRNAs (related to the nervous system)		1. Visual system (rhodopsin, *zfrho;* blue opsin, *zfblue*)	Chen et al. (2017a)
PS (70 ± 9.21 nm) (injected 0.52 nL volume of 1,000, 3,000, and 5,000 mg/L and also exposed to 0.5 and 5 mg/L PSNAP waterborne until hatching), depurated until 4 weeks)	Zebrafish (embryos)	Whole larvae		1. *sod2* (in waterborne exposure) 2. *mbp* (responsible for myelination of axons) and *syn2α* (a neuronal phosphoprotein, induced synaptogenesis) (in injected fish) 3. *gfap* (an intermediate filament protein, expressed in astrocytes) (in waterborne fish) 4. Visual system cone genes (*opn1sw2, opn1lw2,* and *opn1mw*1) (injected fish) 5. Visual system cone genes *(opn1w2* and *opn1mw1)* (waterborne)	1. *sod*1 and *sod2* (in injected fish)	Zhang et al. (2020)
PS (100 nm) (exposed to 10 μg/L; waterborne until 12 hpf) and depurated until 120 hpf)	Zebrafish (embryos 2 hpf)	Whole larvae		1. *dnmt3bb1* and dnmt*3bb2* 2. *cyp19a1a* and *cyp19a1b*		Liu et al. (2021)
PS (50 nm) (exposed to 1 mg/L; waterborne until 96 hpf)	Zebrafish (embryos)	Whole larvae	1. *abcc2* and *P-gp* (efflux transporter genes)	1. *abcc1, abcc4*, and *abcb4* (efflux transporter genes)	1. Metallothionein *(mt2)* 2. *gadd45a, p53, xrcc2, rad51*, and *trl3*	Bhagat et al. (2022)
PS (50 and 100 nm) (0.1, 0.5, 2, and 10 mg/L; waterborne exposure 120 hpf	Zebrafish (embryos)	Liver	1. Liver-specific fatty acid-binding protein 10a (*fabb10a*)			Cheng et al. (2022)
PS (100 nm) (100, 200, and 400 mg/L; 24 h waterborne)	Zebrafish (embryos)	Whole embryo	1. Base excision pathways (*lig1, lig3, polb, parp1, pold, fen1, nthl1, apex,* and *xrcc1*) 2. *lig3, polb*, and *ogg1* (lower concentration)	1. Flap endonuclease 1 *(fen1)*		Feng et al. (2022)
PS (100 nm) (10 μg/L, waterborne) exposed for 144 hpf and depurated for 3 days	Zebrafish (embryos, 2 hpf)	Whole embryo	1. *pax2, pax6,* and *six3* (retinal system development)	1. The f*oxg1* related to stem cell expression 2. *lhx9* (retinal system development)		Liu et al. (2022b)
PS (80 nm) (50 μg/L, 100 μg/L, 1 mg/L, 5 mg/L, and 10 mg/L; waterborne, 120 hpf)	Zebrafish (embryos)	Whole larvae	1. *tshβ* (HPT axis) 2. *tg* (thyroglobulin) 3. Sodium (Na)–iodide symporter (NIS) 4. *trβ* 5. *esr2* 6. *vtg*	1. Thyroxine-transport protein gene (*ttr*) and *dio2*	1. *trα*	Wang et al. (2022)
PS (50 nm) (0.1, 1, 5, 10, 20, 30, and 50 mg/L) (waterborne exposure for 5 days and depurated until 12 days)	Zebrafish (embryos)	Whole larvae	1. *Slc7a11, Acs14a, Keap1b*, and *Ncoa4* (ferroptosis) 2. *Adma, Nos,* and *Pde6d* (NO-sGC-cGMP pathway) 3. *tnfα, tgfβ, il-4, il-6 (*inflammatory cytokines) 4. *bik, bad, bax, bim, bid*, and *bok* (mitochondrial-dependent apoptosis pathways)	1. *prkg*		Chen et al. (2023c)
PS (20 nm) (2, 5, and 8 mg/L) (waterborne, exposed for 22, 46, and 70 h)	Zebrafish (embryos, 2 hpf)	Whole embryo	1. *vegfa, nrp1,* and *klf6a* increased after 22 hpf (VEGFA/VEGFR pathways) 2. *fik1, cldn5a* (VEGFA/VEGFR pathways) 3. *rspo3* (VEGFA/VEGFR pathways)	1. *vegfa, nrp1,* and *klf6a* (VEGFA/VEGFR pathways) decreased after 46 hpf 2. *flt1* and *fih1* (VEGFA/VEGFR pathways)		Dai et al. (2023)
PS (80 nm) (5, 10, 25, 50, and 100 μg/L) (waterborne, exposed until 96 hpf)	Zebrafish (embryos 2 hpf)	Whole larvae		1. *runx2a, runx2b, sp7, bmp2b,* and *shh* (related to osteogenesis)		Gao et al. (2023b)
PS (30 nm and 100 nm) (0.1, 1, and 10 mg/L, exposed for 96 h)	Zebrafish (embryos 5 hpf)	Whole larvae	1. *il6* and *il1β (*pro-inflammatory cytokine genes) 2. *cyp1a* and *cyp51 (cytochrome* P450 genes*)*			Martin et al. (2023)
PS (30 nm) (0.1, 0.5, and 3 mg/L, waterborne, exposed for 120 hpf)	Zebrafish (embryos, 1 hpf)	Whole larvae	1. *hsp70* (heat shock protein) 2. *sod1* and *sod2* (oxidative stress) 3. *cas1* and *cas8* (apoptosis) 4. *bcl2a* (antiapoptotic) 5. *il1β* (inflammation)	1. *bcl2a* (antiapoptotic) 2. *AChE* (neurotransmitter)	1. *hsp27* and *hsp90* (heat shock proteins) 2. *cat* (oxidative stress) 3. *gadd45α* and *rad51* (DNA damage) 4. *cas3a* (apoptosis) 5. *cox1* (mitochondrial metabolism)	Martin-Folgar et al. (2023)
PS (80 nm) (0.05, 0.1, 1, 5, and 10 mg/L, waterborne, exposed for 120 hpf)	Zebrafish (fertilized eggs)	Whole larvae		1. *gpx1a* (antioxidant gene)	1. *cyp1a1* (cytochrome P450)	Wang et al. (2023c)
PS (100 nm) (10 mg/L, waterborne, exposed for 5 days)	Zebrafish embryos (2 hpf)	Whole larvae	1. *caspase 3a* and *Baxa* (apoptosis)	1. *Gap43, C-fos, Bdnf, Shha, Neurog1,* and *Flavl3* (central nervous system development)	1. *Gfap, Syn2a, Mbpa*, and *a1b-tubulin* (central nervous system development) 2. *Bcl2a* (apoptosis)	Zhou et al. (2023c)
PS (80 nm) (0.1, 0.5, 1, 5, 10, 25, and 50 mg/L) (waterborne, exposed for 120 hpf)	Zebrafish embryos (8 hpf))	Whole larvae	1. *gfap* and *rab33a* (neural genes) 2. *rho, opn1sw1,* and *opn1* (optical genes)			Chen et al. (2024)
PS (80 nm) (1 mg/L) waterborne, exposed for 21 days	Zebrafish (adults)	Gut	1. *il8, il10, il1β*, and *tnf α* (inflammation)		1. *il6* and *ifnphi 1*	Xie et al. (2021)
PS (54.5 ± 2.8 nm) (10 mg/L), waterborne, exposed for 120 days. Both P1 and F1	Zebrafish (adults)	Brain and liver			1. *tshβ* (female brain, F0) 2. *trβ* (male liver, F0*)*	Zhao et al. (2021)
PS (70 nm) (100 μg/L, waterborne, exposed for 3 months	Zebrafish (adult male and female fish)	Liver			1. *p38a, p38b, ERK2, ERK3, Nrf2, H O -1, cat1, sod1, gax, JINK1*, and *gstr1* (antioxidant)	Ling et al. (2022)
PS (100 nm) (25 mg/L; exposed at 28-, 29-, and 30°C for 96 h)	Zebrafish (adults, 3 months old)	Brain	1. *Gfap*, (indicator of CNS injuries) 2. *8-OHdG* (indicator of oxidative stress)			Sulukan et al. (2022a)
PS (44 nm) (1, 10, and 100 μg/L, waterborne, exposed for 30 and 60 days)	Zebrafish (juveniles and adults)	Intestine	1. *tnf, il1b, il6, il10, cxcl8a* (exposed to 100 μg/L), *caspase B*, and *tight junction protein 2a* (exposed to 100 μg/L; 30 days of exposure)	1. *tnfα*, *interferon*, *il1β, il10*, and *chemokine 8a* [ exposed to 1 and 10 μg/L: 30 days of exposure) 2. *ahr* (30 days of exposure)		Teng et al. (2022b)
PS (100 nm) (500 ng/mL) waterborne, exposed for 28 days	Zebrafish (adults)	Liver (hepatocytes)	1. *Ldlra, plin2, zbtb16a, foxo1a, angpt14, txnipa, klf6a, c7b, si: dkey-22f5.9*, and *hsd11b2* (male hepatocytes) 2. *vtg6, crp2.1, crp2, igfbp1b, slc38a4, bzw1b, si: dkeyp-73d8.9, pck1, angptl4,* and *chac1* (female hepatocytes) 3. *ccl33.3*, *adh8a*, *fabp10a, fetub*, *si: dkey-7f3.14*, *apoa1b, si:ch211-222121.1, si: dkeyp-73d8.9, apoa2*, and *agxtb*. (macrophages) 4. *BX901920.1, CU914776.1, ins, NC-002333.4, FQ323156.1, hbba1.1, CR753876.1nfkbiaa, ccl20a.3,* and *egr3* (lymphocytes) 5. *Ins, pik3r1, deptor, ulk2,* and *hmgb1a (non*-parenchyma cells in liver)	1. *h1fx, rpf26, BX908782.2, si:ch 1973-110a20.7, cbln11, hamp, vtg1, sgk1, ldhba,* and *ccl39.2* (male hepatocytes) 2. *rpl26, cbla11, mycb, si:ch1073-110a20.7, mt2, CR318588.1, si:ch211-270n8.1, rnasel2, bhmt*, and *npm1a* (female hepatocytes) 3. *lygl1, si: dkey-30j10.5, anxa3b, MFAP4, lgals2a, si:dkey-5n18.1, c1qb, gnr1, c1qc,* and *ccl34a.4* (macrophage) 4. *si: dkey21e2.12.1, vtg1, si: dkeyp-75b4.10, icn, BX908782.2, si:ch211-14a17.10, mmp13a.1, lect2l, lyz,* and *grn2* (lymphocytes)		Deng et al. (2023)
PS (80 nm) (15 and 150 mg/L, waterborne, exposed for 21 days	Zebrafish (adults)	Liver	1. Hydroxy-3-methylglutaryl coenzyme A (*hmgcra*), sterol regulatory element-binding protein (*srebp1*), diaceylglycerol aceyltransferase 1b (*dgat1b*), acetyl coenzyme A carboxylase (*acc*) and carbohydrate response element-binding protein (*cvhrebp*)	1. carnitine palmitoyl transferase 1 (*cpt1)*		Li et al. (2023a)
PS (100 nm) (1 mg/L, waterborne, exposed for 30 days)	Zebrafish (adults)	Brain	1. Mitochondrial division-related genes (*drp1, mff, fis 1, mid49,* and *mid51*) 2. Related to mitophagy (*ulk1a* and *parl*) 3. *htr1aa, htr1ab*, and *htr2*c (5-HT receptor RNA in the brain)	1. Mitochondrial fusion-related genes (*mfn1a, mf1b*, and *opa1*) 2. The neurotransmitter catabolic gene (*mao)* 3. *tp1a, tp1b,* and *tph2* (tryptophan hydroxylase) 4. *htr1b* and *htr4*	1. Mitochondrial synthesis (*pgc1-a* and *pgc1-b*) in the brain 2. Neurotransmitter synthase gene (*th*) and *chat*	Zhang et al. (2023)
PS (50 nm) (1.0 mg/L, waterborne, exposed for 21 days)	Zebrafish (adults)	Gonad (ovary) and liver			1. *Sgk1* and *stc* (ovary) 2. *cyp17a2* and *hsdβ1* (ovary) 3. *esr2b, vtg1*, or *vtg2* (female liver) 4. *esr2b* or *vtg2* (male liver)	Ye et al. (2024)

**TABLE 8:** Effects of NAPs and various environmental contaminants used in coexposure studies on the toxicological endpoints of fish.

Additives (name/concentration)	Type/nature	Fish	Developmental stages	Nanoplastics (name/size/concentrations)	Mode of exposure and duration	Results	References
Acetaminophen (APAP) (2 and 8 mM)	Drug	Zebrafish (*Danio rerio*)	Embryos (3 hpf)	PS (80 nm) (100 μg/L)	Waterborne (96 hpf)	1. PS was unable to induce developmental disorders (pericardial edema, spinal curvature, pigment deficiency, and melanocyte abnormalities), which were more pronounced with coexposure with APAP 2. Body length tended to reduce with coexposure with APAP 3. PS induced hyperactivity in swimming behavior of the larvae. Coexposure with APAP caused depressed swimming activities (total distance, swimming speed, and the maximum acceleration)	Gao et al. (2023b)
*Aeromonas hydrophilia* (2 × 10^7^ CFU/mL)	Bacteria	Grass carp (*Ctenopharyngodon idella*)	Juveniles	PS (80 nm) (10, 100, and 1,000 μg/L)	PS = waterborne (5 days) bacteria = injection; depurated for 3 days	1. Pronounced the intestinal damage induced by the PS alone 2. Increased the CAT, GST, SOD, and MPO activities and MDA content in the intestine induced by the PS alone 3. Induced modifications in the microbial composition	Li et al. (2024a)
nAL_2_O_3_ (1 mg/L)	Metal	Zebrafish (*Danio rerio*)	Embryos	PS (50 nm) (1 mg/L)	Waterborne (96 hpf)	1. PS enhanced the accumulation of Al_2_O_3_ 2. PSNAP alone or in combination enhanced ROS. 3. Coexposure significantly decreased the GPx activity 4. Coexposure enhanced GSH content, which remained by exposure to either PS or Al_2_O_3_	Bhagat et al. (2022)
Arsenic (As; 200 μg/L)	Metalloid	Zebrafish (*Danio rerio*)	Adults	PS (100 nm) (1 mg/L)	Waterborne (30 days)	1. PSNAP enhanced the accumulation of As in the brain 2. Compared with controls, the level of ROS significantly increased in the brain of zebrafish exposed to PSNAP and As, either alone or in coexposed conditions 3. The SOD activity significantly increased and the GSH content significantly decreased in the brain of fish coexposed to As + PSNAP 4. The MDA content in the brain of zebrafish, compared with controls, significantly increased in fish exposed to As alone or in combination with PSNAP. 5. Compared with controls, a small amount of micro thrombosis consisting of aggregated and dissolved red blood cells and the mitochondria with a damaged membrane and loss of cristae were observed in the brain of the fish exposed to PSNAP and As either alone or in combinations 6. The mitochondrial DNA copy number was significantly reduced in fish exposed to PSNAP, As, and also in combinations when compared with the controls	Zhang et al. (2023)
As (1 mg/L)	Metalloid	Zebrafish (*Danio rerio*)	Adult	PS (100 nm) (1 mg/L)	Waterborne (30 days)	1. Compared with controls, there was no significant difference in the mortality of the fish exposed to PSNAP, As, and PSNAP + As groups 2. The swimming speed significantly decreased in fish exposed to PSNAP and As alone or in combinations compared with controls 3. The anxiety-like behavior (evaluated by the open-field test) showed the coexposure group and those exposed to PSNAP alone spent more time in the lower layer than in the upper layer, while controls and As groups spent uniform time in both upper and lower layers 4. The learning memory ability (evaluated by T-maze test) showed control and PSNAP groups swam quickly in the feeding zone (F zone) and stayed there for a long time, while the fish exposed to As and in combinations stayed both in the F zone and stimulating zone (S zone) 5. Compared with controls, the level of the 5-hydroxytryptamine (5-HT) level in the brain was significantly reduced in fish exposed to PSNAP and As; moreover, coexposure further promoted the reduction 6. The 5-HT levels in the serum remained unaltered in fish exposed to PSNAP and As and were significantly reduced in coexposure groups when compared with controls 7. In intestines, the 5-HT level tended to decrease in fish exposed to PSNAP and As alone or in fish exposed to a combination 8. The activity of MAO (the catalytic enzyme of 5-HT) and the mRNA level of *mao* in the intestine tended to decrease in fish exposed to PSNAP and As either alone or in combination when compared with controls	Zhang et al. (2024c)
Avobenzone (AVO) or butyl methoxydibenzoylmethane (1, 10, and 100 μg/L)	PCP/sunscreen	Zebrafish (*Danio rerio*)	Embryos	PS (100 nm) (10 μg/L)	Waterborne (2–12 hpf). Depurated until 120 hpf	1. PS decreased the adsorption of AVO on embryos 2. Combined exposure caused lower levels of oxidative stress than individual exposures	Liu et al. (2021)
Avobenzone (AVO; 10 μg/L)	PCP/sunscreen	Zebrafish (*Danio rerio*)	Embryos (2 hpf)	PS (100 nm) (10 μg/L)	Waterborne (144 hpf). Depurated for 3 days	1. PS promoted the accumulation of AVO in zebrafish embryos	Liu et al. (2022b)
Benzo [a] pyrene (BAP) (0.1, 0.5, 1, 5, and 10 mg/L)	PAH	Zebrafish (*Danio rerio*)	Embryos	PS (50 nm) (0.069, 0.69, 69, 687, and 6,870 μg/L) (120 hpf)	Waterborne (120 hpf)	1. PS function as a vector for BAP 2. Accumulation of PS was observed in the chorion, eye, tail, and yolk sac of the embryos at different time points of development	Martinez-Alvarez et al. (2022)
BDE-47 (10 ng/L)	Flame retardant	Zebrafish (*Danio rerio*)	Embryos	PS (100 nm) (2.5 and 25 μg/L)	Waterborne (7 days)	1. Coexposure increased feeding and oxygen consumption rates 2. BDE-47-induced gene expression was abolished by coexposure with PS.	Chackal et al. (2022)
BDE-47 (0.1 mg/L)	Flame retardant	Zebrafish (*Danio rerio*)	Embryos	PS (80 nm) 0.05, 0.1, 1, 5, and 10 mg/L	Waterborne (120 hpf)	1. Coexposure enhanced mortality in a time- and concentration-dependent manner 2. Decreased heart rates by BDE-47 and PS coexposure 3. Spontaneous movement of the embryos during 12 hpf, which was stimulated by BDE-47 and reduced by PS during coexposure 4. Coexposure to PSNAP and BDE-47 induced greater damage to the retinal structures in the eyes, muscle fiber, and cartilage tissue	Wang et al. (2022)
BDE-47 (0.1 and 10 μg/L)	Flame retardant	Zebrafish (*Danio rerio*)	Embryos	PS (80 nm) (0.05, 0.1, 1, 5, and 10 mg/L)	Waterborne (120 hpf)	1. No significant effect on mortality was observed in embryos exposed to PS; however, concentration-dependent effect was observed in coexposure groups (120 hpf) 2. Hatching stimulated by PS was modulated by BDE-47 coexposure 3. BDE-47 decreased heart rates of the 96 hpf embryos, while PS in the coexposure group is unable to modulate the effects 4. Liver size was markedly reduced in coexposure groups than the fish exposed either to PS or BDE-47 5. Coexposure exacerbated ROS production compared with single-exposure groups	Wang et al. (2023c)
Butylmethoxydibenzoylmethane (BMDBM) or avobenzone (1,10, and 100 μg/L)	PCP/sunscreen	Zebrafish	Embryos (2 hpf)	PS (100 nm) (10 μg/L)	Waterborne (2–12 hpf)	1. The brain development, head development, and notch signaling pathways were altered by both pollutants 2. Among the 7 cell types identified in zebrafish embryos (neural anterior cells, neural crest cells, neural mid cells, neural posterior cells, endoderm cells, mesoderm cells, and epidermal cells), the neuronal mid cells are the targets of both PS and BMDBM 3. Significant inhibition in the locomotor activity	Liu et al. (2021)
Bisphenol A (BPA) (100 μg/L)	Plastic additive	Marine medaka (*Oryzias melastigma*)	Embryos (6 hpf)	PS (50 nm) (55 μg/L)	Waterborne (21 days)	1. Accumulation of PS decreased in the presence of BPA 2. Presence of BPA reduced the developmental abnormalities induced by PS 3. The presence of BPA reduced the histopathological changes induced by PS in the liver (vacuolation, apoptosis, and necrosis) and heart	Yu et al. (2023)
BPA (0.78 μg/L)	Plastic additive	Zebrafish (*Danio rerio*)	Adults (6 months old)	PS (47 nm) (1 mg/L)	Waterborne (3 days)	1. Coexposure increased BPA uptake 2. No inhibition of AChE activity in coexposure groups 3. Coexposure upregulated the expression of myelin, tubulin protein/gene expression, dopamine content, and the mRNA expression of mesencephalic astrocyte-derived neurotrophic factor (MANF)	Chen et al. (2017b)
nCeO_s_ (1 mg/L)	Metal	Zebrafish (*Danio rerio*)	Embryos	PS (50 nm) (1 mg/L)	Waterborne (96 hpf)	1. PS enhanced the accumulation of Ce 2. The hatching rate declined in embryos co-exposed with nCeO_2_ 3. PS alone or in combination enhanced ROS. 4. CAT activity remained unaltered in fish exposed to CeO_2_ alone or in combinations, which was increased by PS exposure 5. GPx was induced in fish exposed to CeO_2_ alone; however, it was significantly reduced in fish coexposed with PSNAP.	Bhagat et al. (2022)
Chloroauric acid (1 μg/mL)	Inorganic compound	Zebrafish (*Danio rerio*)	Embryos	PS (50 nm) (0.1 mg/L)	Waterborne (6, 24, and 96 hpf)	1. Chloroauric acid (Au ions) synergistically exacerbated the effects of PS (hatching rates, developmental abnormalities, and cell death) in a concentration- and size-dependent manner	Lee et al. (2019)
*p, p’*-DDE (100 μg/L)	Insecticide	Zebrafish (*Danio rerio*)	Embryos	PS (15 nm) (50 mg/L)	Waterborne (96 hpf)	1. No significant difference was observed in the oxygen consumption rate of the larvae exposed to PS only; however, in DDE and PS + DDE groups, oxygen consumption rates increased significantly compared to those in controls 2. DDE alone or in combination with PSNAP induced pericardial edema, lordosis, and uninflated swim bladder 3. Locomotor behavior of the larvae (movement, distance moved, velocity, angular velocity, and rotations) did not change after PSNAP exposure, while significant alterations (reductions) were noticed in larvae exposed to DDE alone or DDE + PSNAP	Varshney et al. (2023)
Diethylstilbesterol (DES) (1,10, and 100 ng/L)	Synthetic hormone (estradiol)	Zebrafish (*Danio rerio*)	Adults (male and female) 5 months old	PS (70 nm) (2 mg/L)	Waterborne (21 days)	1. PSNPS and DES alone or in coexposure induced lacunae in the testis and increased the number of spermatogonium and spermatocytes in the testis; moreover, deformation of seminiferous tubules was observed 2. PSNAP and DES alone or coexposure groups showed more preovulatory oocytes and smaller mature oocytes than controls 3. Both PSNAP and DES (concentration-dependent) alone and in coexposure decreased the levels of E2 and T in both male and female zebrafish 4. The VTG content of male fish remained unaltered after PSNAP exposure; however, DES alone or coexposed with PSNAP enhanced the VTG content in a concentration-dependent manner in male fish; however, in female fish, NPS alone or in combination with DES reduced the VTG content in a concentration-dependent manner 5. PSNAP exposure has no significant effects on the T3 and T4 levels of both male and female fish; however, DES alone or in combination with PSNAP decreased both T3 and T4 contents in male and female fish in a concentration-dependent manner 6. Compared to controls, PSNAP and DES alone or in combination reduced fecundity, spawning events, fertilization, and hatchability of the embryos 7. PSNAP and DES either alone or in combination induced abnormal development (teratogenic effects) of the larvae observed at 96 hpf (spinal curvature, pericardial cyst, and growth retardation)	Lin et al. (2023)
Diphenhydramine (DPH) (0.01 and 10 mg/L)	Antihistamine	Zebrafish (*Danio rerio*)	Embryos	PS (44 nm) (0.015 and 1.5 mg/L)	Waterborne (96–120 f)	1. After 96 h, coexposure induced mortality, malformation, decreased heart rates, and hatching 2. After 120 h, coexposure decreased the swimming activity 3. After 96 h, glutathione S-transferase and cholinesterase activities increased in coexposure groups, while catalase activity remained unaltered	Barreto et al. (2023)
17α-ethinylestradiol (EE2) (2 and 20 μg/L)	Hormone (synthetic estrogen)	Zebrafish (*Danio rerio*)	Embryos	PS (47 nm) (1 mg/L	Waterborne (120 h)	1. PS can reduce the accumulation of EE2 in larvae 2. EE2 can change the swimming behavior of the larvae (hypoactivity) induced by PS	Chen et al. (2017a)
F-53B (500 μg/L)	Polyfluoroalkyl substance	Hainan medaka (*Oryzias curvinotus*)	Adults (length 2.85 ± 0.17 cm; weight 440 ± 90 mg)	PS (80 nm) (200 μg/L)	Waterborne (7 days)	1. F-53B interferes with the accumulation of PSNAPs in the gills and intestine 2. Attenuation of hepatic damage (appearance of eosinophilic vesicles and vacuolization) by PS induced by F-53B	Gao et al. (2023a)
Glucose (40 mM)	Carbohydrate	Zebrafish (*Danio rerio*)	Larvae (72 hpf)	PS (25 nm) (20 mg/L)	Waterborne (exposed 72–120 hpf)	1. The presence of glucose had no effect on the cortisol concentrations induced by PS Hyperactivity (movement) of larvae induced by PS was reduced by glucose	Brun et al. (2019)
Homosolate (0.0262–262 μg/L)	Organic compound/UV filter	Zebrafish (*Danio rerio*)	Adults	PS (50 nm) (1 mg/L)	Waterborne days)	1. PS enhanced (not significant) the accumulation of homosolate in the testis, ovary, liver, and brain of male and female fish 2. Exposure to PSNAP alone was unable to alter the amount (percentage) of PO, LVO, CAO, and EVO in the ovary; however, coexposure with homosolate decreased the number of PO and increased the number of LVO and CAO and EVO remained unaltered 3. PS alone has no significant effect on the amount of spermatogonium, spermatocytes, spermatids, and spermatozoa (percent); however, coexposure with homosolate showed testicular damage (lacunae in the seminiferous tubules) with a decreased amount of spermatozoa and no effect on spermatogonia, spermatocytes, or spermatids 4. Egg production and hatching rates remained unaffected by PSNAP exposure alone; however, hatching rates reduced in coexposure with homosolate in a concentration-dependent manner 5. PSNAP alone has no significant effect on F1 embryo mortality; however, coexposure with homosolate enhanced F1 embryo mortality 6. No significant effect of PSNAP alone in the malformation of F1 larvae (spinal curvature, swim bladder deformities, mandibular malformation, body edema, yolk sac edema, pericardial edema, and tail deformity) was observed; however, coexposure with homosolate enhanced the malformation rates of the F1 embryos 7. No effect of PSNAP was observed in the expressions of *sgk1* and *stc* mRNAs in the ovary of adult zebrafish; however, coexposure with homosolate enhanced the expressions of both *sgk1* and *stc* mRNAs in the ovary 8. No effect was observed in the E2 level in the ovary and serum of the fish exposed to PSNAP alone; however, co exposure with homosolate enhanced the E2 content in the ovary as well as in the serum 9. T content in the ovary did not alter in zebrafish after exposure with PSNAP alone or in combination with homosolate 10. PSNAP alone was unable to alter the GnRH and FSH levels in the ovary; however, PSNAP attenuated the effects induced by homosolate alone (increased GnRH and FSH) in the ovary of zebrafish 11. PSNAP did not exhibit any effect on the LH content in the ovary when exposed alone; however, coexposure with homosolate enhanced the LH content in the ovary 12. In male fish, serum E2 and testis E2 levels and GnRH and FSH contents remained unaltered in fish exposed to PSNAP alone; however, PSNAP attenuated the effects induced by homosolate alone (increased serum T and testis T and GnRH and FSH in the testis) in the zebrafish 13. The LH levels in the testis significantly reduced with exposure to PSNAP alone, and coexposure with homosolate aggravated the effect	Ye et al. (2024)
Lead (50 μg/L)	Metal	Zebrafish (*Danio rerio*)	Adults	PS (100 nm) (20 and 200 μg/L)	Waterborne (exposed for 3 weeks)	1. Lead increased the accumulation of PS in the intestine 2. There are seven types of cell populations identified in the intestine: enterocytes, macrophages, neutrophils, B cells, T cells, enteroendocrine cells, and goblet cells 3. Lead with PS enhanced the MDA content in the intestine compared to the fish exposed to PS alone 4. The 8-hydroxy-2′-deoxygluconate (8-OHdG) level was enhanced in the intestine by lead, and presence of PS in the medium significantly increased 8-OHdG level induced by exposure to lead alone 5. TNF-α level was increased by PS in a concentration-dependent manner, and presence of lead in the medium enhanced the TNF-α level compared to the fish exposed to PS or lead alone 6. In macrophages, immune system-related DEGs (*ctsba, nfkbiab*, and *pycard*) were significantly altered in PSNAP fish than PSNAP + lead groups, and the genes related to MAPK signaling pathways (*hsp70.1, hsp70.2*, and *hsp70l*) were altered in fish exposed only to lead 7. In enterocytes, genes related to glutathione metabolism and cytochrome P450 (*gsta2, gsto 1, gsto2, gpx1a,* and *mgst1.2*) were significantly altered in fish exposed to lead and lead + PSNAP. 8. In B and T cells, upregulation of *hsp70.1, hsp70.*2, and *hsp70.3* occurred in fish exposed to PSNAP, lead, and also in combinations 9. Gene ontology (GO) analysis found several other DEGs altered in macrophages after PSNAP exposure, such as *gadd45ba, jun, ccl35.2,* and *ccl35.2*. and in PSNAP + lead groups were *ccr9a, cxcr4b*, and b*cl2l10*; however, lead exposure altered *mt2* and *pycard* 10. In enterocytes, GO analysis showed alterations in the expressions of *apoa4a, apoa1a*, and *apoea* in fish exposed to PSNAP and lead either alone or in combinations. Moreover, expressions of *npc2* and *prdx1* were altered in fish exposed to lead and lead + PSNAP	Yu et al. (2022a)
Microcystin LR (MCLR) (0.9, 4.5, and 22.5 μg/L)	Antibiotics	Zebrafish (*Danio rerio*)	Adults	PS (70 nm) (100 μg/L)	Waterborne (96 h) 21 days parental exposure (F0) and F1 larvae (120 hpf) were evaluated without exposure	1. Due to parental exposure, accumulation of PS was observed in the testis and ovary of the F1 larvae, and PS increased the accumulation of MCL in F1 larvae 2. Parental exposure of MCL and PSNAP + MCL affects the hatchability (decreased), malformation (decreased), mortality (increased), body length (decreased), and heart rates (decreased) of the F1 larvae 3. Parental exposure of MCL either alone or in coexposure with PS reduced T4 and T3 levels of the F1 larvae	Zuo et al. (2021)
Microcystin LR (MCL) (0.9, 4.5, and 22.5 μg/L)	Antibiotics	Zebrafish (*Danio rerio*)	Adults (male and female)	PS (70 nm) (100 μg/L)	Waterborne (96 h) 3 months	1. PS enhanced the accumulation of MCL in the liver of fish 2. In the liver, cellular swelling, fat vacuolation, and cytoarchitectural damage were induced by MCL, and PS exacerbated these adverse effects 3. MCLR alone enhanced ROS and MDA contents of the liver in a concentration-dependent manner, and the presence of PS exacerbated the effects 4. The GST and CAT activities reduced in a concentration-dependent manner by MCLR, and the presence of PSNAP further reduced the enzymatic activities	Ling et al. (2022)
Microcystin-LR (MSL) (1 μg/L)	Antibiotics	Silver carp (*Hypophthalmichthys molitrix*)	Adults (9.33 ± 1.01 cm length, 10.43 ± 3.41 g weight)	PS (80 nm) (10 and 1,000 μg/L)	Waterborne (96 h)	1. The length of intestinal villi is significantly shorter 2. Imbalance in glycerophospholipid metabolism 3. Increase in hepatocyte space 4. The diversity and richness in gut microbiota increase by PS exposure was further enhanced by MSL	Zhang et al. (2024a)
4-Nonylphenol (1 μg/L)	Nonionic surfactant	Zebrafish (*Danio rerio*)	Adults	PS (20–80 nm) average size 57.5 nm (0.1, 1, 10, and 100 μg/L)	Waterborne (45 days)	1. Inhibition of AChE activity in the brain induced by PS and 4-NP exposure was inhibited by coexposure with 4-NP and PS. 2. The activity of brain glutamine synthase (GS) decreased by PSNAP or 4-nonylphenol exposure alone was increased by coexposure 3. 4-NP alone or in combinations showed severe damage in neuronal cell layers as well as reduced the number of neurons	Aliakbarzadeh et al. (2023)
Oxytetracycline (100 μg/L)	Antibiotics	Zebrafish (*Danio rerio*)	Adults (6 months old)	PS (40–54 nm) (60–338 μg/L)	Waterborne (30 days)	1. The intestinal damages induced by OTC (rapture and lysis of the epithelial layers and vacuolation of the intestinal cells) was reduced by coexposure with PSNAP 2. The gut microbial diversity was significantly affected by PSNAP and OTC exposure either alone or in combinations	Yu et al. (2022b)
Penicillin (1 and 10 μg/L)	Antibiotics	Zebrafish (*Danio rerio*)	Embryos (8 hpf)	PS (80 nm) (0.5 and 5 mg/L)	Waterborne (120 hpf)	1. Accumulation of PS in the yolk sac, eye, head, and nerve tubes was interrupted by penicillin 2. Penicillin interrupted motor behaviors (spontaneous movements, touch response, and swimming) and heart beats during development	Chen et al. (2023b)
Phenanthrene (PHE) (0.1, 0.5, and 1.0 mg/L) and jellyfish mucin (50 μg/mL)	PHE (polycyclic aromatic hydrocarbon); mucin (biological substance)	Zebrafish (*Danio rerio*)	Embryos	PS (50 nm) (5 mg/L)	Waterborne (4, 8, 12, 24, 32, 48, and 72 hpf)	1. Mucin obstructed the absorption of PS and PHE into the embryos	Geum and Yeo, (2022)
Phenmedipham (PHN) (0.02, 0.2, and 20 mg/L)	Herbicide	Zebrafish (*Danio rerio*)	Embryos	PS (44 nm) (0.015 and 1.5 mg/L)	Waterborne (96–120 hpf)	1. The effects induced by PS in locomotion and oxidative stress were reduced by PHN coexposure	Santos et al. (2022)
Polycyclic aromatic hydrocarbons (PAH) (5.07–25.36 μg/L)	Organic substance	Zebrafish (*Danio rerio*)	Embryos	PS (44 nm) (0.1, 1, and 10 mg/L)	Waterborne (96 hpf)	1. PS decreased the absorption of the PAH 2. PS impaired vascular development caused by PAH	Trevisan et al. (2019)
Polycyclic aromatic hydrocarbons (PAH) (1 mg/L)	Organic substance	Zebrafish (*Danio rerio*)	Embryos	PS (44 nm) (1 mg/L)	Waterborne (96 hpf) (7 days)	1. PAH accumulation did not interrupt the accumulation of PS in the brain 2. PS either alone or in coexposure increased NADH production	Trevisan et al. (2020)
Simvastatin (SIM) (0.015–150 μg/L)	Statin	Zebrafish (*Danio rerio*)	Embryos	PS (60 nm) (0.05 or 1.5 mg/L)	Waterborne (96 h)	1. Hatching delay and decreased heart beats induced by SIM were interrupted by PS exposure	Barreto et al. (2021)
Sodium nitroprusside (8 µM)	Inorganic compound/	Zebrafish (*Danio rerio*)	Embryos	PS (50 nm) (20 mg/L)	Waterborne (12 days)	1. SNP reduced the accumulation of PS in larvae 2. SNP alleviated the toxic effects of PS 3. PS increased the NO content, while co-exposure with SNP did not potentiate the effect 4. PS decreased the activities of soluble guanylate cyclase (sGC) and protein kinase G (PKG) enzymes; however, coexposure with SNP diminished the effects of PS on enzymatic activities 5. PS exposure enhanced ROS levels in the larvae, and coexposure with SNP did not aggravate the ROS content 6. The metabolic level of the liver was significantly increased in larvae by PS, and SNP coexposure alleviated the process 7. The oxidative stress index (based on CAT, peroxidase, and SOD activities and GSH and MDA contents) was significantly increased by PS, while SNP coexposure alleviated the process 8. PS exposure caused significant apoptosis in larvae, while SNP coexposure significantly alleviated the process 9. PS exposure caused significant mitochondrial depolarization, which was alleviated by SNP coexposure 10. The activity of the caspase-3 was significantly increased by PS, while coexposure with SNP alleviated the process 11. PS exposure induced ferroptosis (cell death due to iron accumulation), while coexposure with SNP alleviated the process 12. PS exposure significantly increased the proliferation of macrophages and neutrophils; coexposure with SNP alleviated the process	Chen et al. (2023c)
Sulfamethazine (SMZ) (0.5 and 5 mg/g)	Antimicrobial agent	Marine medaka (*Oryzias melastigma*)	Adults	PS (100 nm) (5 mg/g)	Dietary (30 days)	1. The intestinal toxicity induced by SMZ (gut microbiota and oxidative stress) was alleviated by PS exposure	Zhang et al. (2021)
Sulfamethazine (SMZ) (4.62 mg/g)	Antimicrobial agent	Marine medaka (*Oryzias melastigma*)	Adults (580.2 ± 189.5 mg body weight)	PS (100 nm) (3.45 mg/g)	Dietary (30 days) parental (F0) exposure; F1 evaluated after 60 days	1. The growth of the (body weight) F1 fish, reduced by PS exposure, was further increased by SMZ coexposure 2. Sex-specific alterations in the expression of several genes (*sod* and *cat* in the intestine of female fish enhanced, while that of *sod* in the intestine of male fish remained enhanced by PS and remained at the same level in coexposure)	He et al. (2022)
Sulfamethazine (SMZ) (0.5 and 5 mg/g)	Antimicrobial agent	Marine medaka (*Oryzias melastigma*)	Adults (4 months old)	PS (100 nm) (5 mg/g)	Dietary (30 days) depurated 21 days	1. Sex-specific alterations in gut microbial community 2. During the depurating phase, higher occurrence of pathogenic bacteria was found in fish belonging to the combined exposure group than that exposed to single pollutant	Wang et al. (2023a)
Sulfamethoxazole (SMX) (100 μg/L	Antibiotics	Marine medaka (*Oryzias melastigma*)	Juveniles (2 months old)	PS (100 nm) (1 mg/L)	Waterborne (30 days)	1. Intestinal mucus volume increased and goblet cell number decreased 2. Gut microbiota altered 3. SMX enhanced the intestinal toxicity (decreased intestinal microbiota diversity and composition and induced intestinal epithelial damage) induced by PS exposure	Li et al. (2023b)
Tetracycline (TC) (5,000 μg/L)	Antibiotics	Grass carp (*Ctenopharyngodon idella*)	Juveniles	PS (80 nm) (20, 200, and 2000 μg/L) -	Waterborne (7 days)	1. Enhanced the total antioxidant capacity and the activities of CAT and SOD in the liver and intestine 2. Induced lesions in the gills and intestine	Liu et al. (2022a)
Triclosan (TCS) (0.01, 0.1, and 1 mg/kg)	Biocide	Tooth carp (*Aphaniops hormuzensis*)	Adults	PS (100 nm) (0.5 mg/L)	Dietary (3, 14, and 28 days)	1. TCS did not significantly affect the uptake of PS into the tissues	Saemi-Komsari et al. (2023)
Triphenyl phosphate (TPhP) (0.08, 0.5, 0.7, 1, 1.2, and 1.5 mg/L)	Flame-retardant and plasticizer	Zebrafish (*Danio rerio*)	Adults (male and female)	PSNAP (46 nm) (2 mg/L)	Waterborne (21 days)	1. Significant increase in the HSI by TPhP was aggravated by coexposure with PS 2. TPhP alone decreased the GSI in male fish and increased in female fish, when coexposed with PS, and the GSI was increased in both male and female fish 3. TPhP alone inhibited spermatogenesis by enhancing the amount of immature spermatocytes (spermatogonium and spermatocytes) and reducing the amount of mature spermatocytes (spermatids and spermatozoa). With coexposure with PS, the amount of mature spermatogenetic cells decreased further, and lacunae and interstitial tissue were observed in seminiferous tubules 4. TPhP inhibited ovarian development by inhibiting the maturation processes of the oocytes having more perinuclear and cortical alveolar oocytes in the female fish exposed to TPhP alone By coexposure with PSNAP, more perinuclear and cortical alveolar oocytes were observed, and some of the mature follicles were atretic 5. Fish exposed to PSNAP or TPhP alone did not affect the E2 and T contents of both male and female fish. Combined exposure of PS and TPhP enhanced the E2 level in male fish but not in female fish 6. PS and TPhP alone has no effect on the vitellogenin (VTG) content in male fish; however, coexposure significantly increased the VTG concentration in male fish 7. In female fish, PS alone had no effect on the VTG content, while TPhP alone significantly inhibited VTG content; coexposure mitigated the effect of TPhP on VTG content in zebrafish 8. Significant inhibition in the fecundity (total eggs produced) of fish exposed to PS or TPhP alone. However, coexposure with PS reduced the fecundity further 9. TPhP alone or in combination with PS reduced spawning events, fertilization, and hatching rates of the embryos	He et al. (2021)
Tris (1,3-dichloro-2-propyl) phosphate (TDCIPP) (0.47, 2.64, or 12.78 μg/L)	Flame-retardant	Zebrafish (*Danio rerio*)	Adults	PS (54.5 ± 2.8 nm) (10 mg/L)	Waterborne (120 days) evaluated F0 and F1 larvae (without exposure)	1. PS enhanced the accumulation of TDCIP fish 2. Total T3 and T4 levels in F0 fish and F1 larvae were not altered significantly when exposed to PS alone; however, fish exposed to TDCIPP alone or in combinations with PS had decreased T3 and T4 levels in F0 female fish and T4 level in F0 male fish 3. In eggs, the T4 level was reduced significantly when the fish were exposed to PS alone and in combination with TDCIPP. 4. In F1 larvae, PS exposure did not induce any significant changes in T3 and T4 contents, while TDCPP exposure decreased T4 levels alone or in combination with PS in a concentration-dependent manner. A concentration-dependent reduction in the T3 level was observed when the parents were exposed to a combination of TDCPP and PS.	Zhao et al. (2021)
Vitamin D (280 and 2,800 IU/kg body weight)	Vitamin	Zebrafish (*Danio rerio*)	Adults	PS (80 nm) (15 and 150 mg/L)	Dietary (for 21 days)	1. High vitamin D diet partially reversed the increases in triglyceride and total cholesterol contents induced by PSNAP exposure 2. Lipidomic analysis showed that in the liver, PSNAP exposure changed the lipid molecular contents related to cell membrane function and lipid biosynthesis; high vit D diet reduced the contents of lipid molecules related to lipid biosynthesis and thus alleviated cell membrane damage and lipid droplet accumulation induced by PSNAP exposure.	Li et al. (2023a)
ZnO (760 μg/L)	Metal oxide	Grass carp (*Ctenopharyngodon idella)*	Juveniles	PS (23.03 ± 0.266 nm) (760 μg/L)	Waterborne (72 h)	1. Affected the response on mirror tests (longer immobility time and shorter interaction with their images) 2. Stimulated the antioxidant activity of the brain 3. Increased AChE activity in the brain 4. Induced DNA damage in erythrocytes	Estrela et al. (2021)

**TABLE 9:** Genotoxic effects of NAPs with various environmental contaminants used in coexposure studies.

Additives (name/concentration)	Type/nature	Fish	Developmental stages	Nanoplastics (name/size/concentrations)	Mode of exposure and duration	Gene expressions	References
Acetaminophen (APAP) (2 and 8 mM)	Drug	Zebrafish (*Danio rerio*)	Embryos (3 hpf)	PS (80 nm) (100 μg/L)	Waterborne (96 hpf)	1. Downregulation of the expression of genes (*runx2a, runx2b, sp7, bmp2b,* and *shh*) related to osteogenesis in PS alone and coexposure groups	Gao et al. (2023b)
*Aeromonas hydrophilia* (2 × 10^7^ CFU/mL)	Bacteria	Grass carp (*Ctenopharyngodon idella*)	Juveniles	PS (80 nm) (10, 100, and 1,000 μg/L)	PS = waterborne (5 days); bacteria = injection; depurated for 3 days	1. *IL-6, IL-8, IL-10, IL-1β, TNF-α,* and *INF-γ2* (immune genes) expressions were upregulated in the intestine exposed to PS alone and infection with *A*. *hydrophilia* in PS-exposed fish enhanced the gene expression induced by PS alone	Li et al. (2024a)
nAL_2_O_3_ (1 mg/L)	Metal	Zebrafish (*Danio rerio*)	Embryos	PS (50 nm) (1 mg/L)	Waterborne (96 hpf)	1. There was no change in the metallothionine (MT) (*mt2*) expression induced by PSNAP exposure alone. Exposure with Al_2_O_3_ alone enhanced *mt2* expression; however, coexposure with PSNAP significantly decreased the expression of *mt2* compared to the expression made by exposure to AL_2_O_3_ alone 2. The expressions of *abcc2* and *P-gp* mRNAs were upregulated, and those of *abcc1, abcc4*, and *abcb4* mRNAs were downregulated (efflux transporter genes) with PSNAP exposure. Coexposure with Al_2_O_3_ modulated the expression patterns of efflux transporter genes (increased expression in *abcc4*) induced by PSNAP exposure.	Bhagat et al. (2022)
Arsenic (As; 200 μg/L)	Metalloid	Zebrafish (*Danio rerio*)	Adults	PS (100 nm) (1 mg/L)	Waterborne (30 days)	1. Expressions of genes related to mitochondrial synthesis (*pgc1-a* and *pgc1-b*) in the brain were significantly downregulated in fish exposed to As alone and in combination with As + PSNAP; however, no significant effect was observed in fish exposed to PSNAP alone 2. Compared with controls, the mitochondrial fusion-related gene (*mfn1a, mf1b*, and *opa1*) expressions were downregulated in the brain of fish exposed to PSNAP, As, and in combinations 3. The expression of mitochondrial division-related genes (*drp1, mff, fis 1, mid49,* and *mid51*) tended to be upregulated by PSNAP exposure, As, and in combinations 4. The expression of genes related to mitophagy (*ulk1a* and *parl*) were upregulated by PSNAP and As exposure either alone or in combinations. Moreover, other mitophagy gene (*parkin, pink 1,* and *fundc1*) expressions were upregulated in combined exposure groups. In addition, the expression of *parkin* was upregulated in fish exposed to As alone 5. The neurotransmitter synthase gene (*th*) expression was significantly downregulated, and that of the *chat* gene was significantly upregulated in the brain of fish exposed to As + PSNAP groups. The other two groups (PSNAP and As) did not induce any significant change 6. The expression of the neurotransmitter catabolic gene *mao* was significantly downregulated in the brain of fish exposed to PSNAP and As, either alone or in combinations	Zhang et al. (2023)
As (1 mg/L)	Metalloid	Zebrafish (*Danio rerio*)	Adult	PS (100 nm) (1 mg/L)	Waterborne (30 days)	1. The mRNAs of tryptophan hydroxylase (TPH), the rate-limiting enzyme for 5-HT synthesis, (*tp1a, tp1b,* and *tph2),* tended to be downregulated in fish exposed to PSNAP and As, either alone or in combinations 2. Among the 5-HT receptor mRNAs, *htr1aa, htr1ab*, and *htr2*c expressions were upregulated in the brain of fish exposed to PSNAP and As, either alone or in coexposure; while the expressions of *htr1b* and *htr4* showed downregulation in fish exposed to PSNAP and As, either alone or in coexposure	Zhang et al. (2024c)
Avobenzone (AVO) or butyl methoxydibenzoylmethane (BMDZM) (1, 10, and 100 μg/L)	POP	Zebrafish (*Danio rerio*)	Embryos	PS (100 nm) (10 μg/L)	Waterborne (2–12 hpf) Depurated until 120 hpf	1. Expressions of *α1- tubulin, elav13, gap43, gfap, mbp,* and *syn2a* were upregulated, and *lfing* expression was downregulated at 12 hpf by AVO alone or coexposure. However, at 144 hpf*, α1-tubulin, elavl3, gap43*, and *mbp* did not show any significant alterations, and after recovery, no alteration was seen in the expressions of all these genes 2. The f*oxg1* (stem cell expression) was upregulated in AVO fish and downregulated in fish exposed to PSNAP alone or in combinations. Other stem cell-related genes (*her5, her6, shha*, and *sox2*) were altered significantly in all three exposure groups. However, after recovery, no significant difference was observed in the expressions of *foxg1, her6, shha,* and *sox 2* 3. The genes related to retinal system development were affected by PSNAP alone or in coexposure. The expressions of *pax2, pax6*, and *six3* were upregulated, while that of *lax9* was downregulated	Liu et al. (2021)
BDE-47 (0.1 mg/L)	Flame-retardant	Zebrafish (*Danio rerio*)	Embryos	PS (80 nm) 0.05, 0.1, 1, 5, and 10 mg/L	Waterborne (120 hpf)	1. The expression of the HPT axis gene *tshβ* was upregulated by PSNAP exposure alone in a concentration-dependent manner; however, it significantly reduced in coexposure groups compared with PSNAP alone (10 mg/L) 2. The expression of the sodium (Na)-iodide symporter (NIS) gene was significantly upregulated by PSNAP alone in a concentration-dependent manner; coexposure showed a reducing tendency (not significantly different) 3. Thyroglobulin (TG) gene expression was significantly upregulated in PSNAP and BDE-47, either alone or in coexposure in a concentration-dependent manner 4. The expression of the thyroxine transport protein gene (TTR) showed a decreasing tendency in larvae exposed to PSNAP and BDE-47, either alone or in combination 5. The expression of *dio2* showed a decreasing tendency in larvae exposed to PSNAP (not significant) compared with controls. BDE-47 alone was able to upregulate *dio2* expression (not significant). Coexposure reduced the expression of *dio2* 6. The expression of *trα* remained unaltered in all treatment groups; however, the expression of *trβ* was upregulated by BDE-47 and PSNAP exposure alone, and coexposure showed a tendency to reduce the expression compared with BDE-47 alone 7. The expression of *esr2* tended to increase with PSNAP exposure alone (not significant); however, coexposure with BDE-47 tended to decrease the expression of *esr2* (not significant) 8. Compared with controls, the *vtg* expression was upregulated in larvae exposed to PSNAP in a concentration-dependent manner. Coexposure reduced the expression of VTG compared with larvae exposed to PSNAP alone	Wang et al. (2022)
BDE-47 (0.1 and 10 μg/L)	Flame retardant	Zebrafish (*Danio rerio*)	Embryos	PS (80 nm) (0.05, 0.1, 1, 5, and 10 mg/L)	Waterborne (120 hpf)	1. Expression of *gpx1a* (an antioxidant gene) was downregulated by PSNAP and BDE-47 either alone or in combination 2. The expression of *cyp1a1* remained unaltered in larvae exposed to PSNAP and BDE-47 alone; however, coexposure upregulated *cyp1a1* expression in a concentration-dependent manner	Wang et al. (2023e)
BMDBM or avobenzone (1, 10, and 100 μg/L)	PCP/sunscreen	Zebrafish (*Danio rerio*)	Embryos	PSNAP (100 nm) (10 μg/L)	Waterborne (120 hpf)	1. BMDBM exposure alone significantly downregulated the expressions of *dnmt1* and *dnmt3aa*, while PSNAP exposure alone significantly decreased the expressions of *dnmt3bb1* and dnmt*3bb2* 2. Coexposure of BMDBM and PSNAP downregulated the expression of *dnmt1* and *dnmt3aa*, while downregulation of *dnmt3bb2* was interrupted as well as no effect was observed in the expression of *dnmt3bb1* 3. BMDBM exposure alone significantly downregulated the expressions of *cyp19a1a* and *cyp19a1b* in a concentration-dependent manner, while PSNAP exposure alone or in combination did not affect the expressions of these genes (*cyp19a1a* and *cyp19a1b*) 4. BMDBM affected the differentiation and fate of neurons in the central nervous system through the regulation of *her5, her6, her11, ifng, pax2a*, and *fgfr4* 5. PSNAP regulated the expressions of *olig2, foxg1a, fzd8b, six3a, rx1, lhx2b, nkx2.1a,* and *sfr5* to alter nervous system development, retinal development, and stem cell differentiation	Liu et al. (2021)
nCeO_2_ (1 mg/L)	Metal	Zebrafish (*Danio rerio*)	Embryos	PS (50 nm) (1 mg/L)	Waterborne (9 hpf)	1. There was no change in metallothionine (MT) (*mt2*) expression by PSNAP exposure alone. Exposure with CeO_2_ alone enhanced *mt2* expression; however, coexposure with PSNAP significantly decreased the expression of *mt2* compared to the expression induced by CeO_2_ alone 2. The expressions of *abcc2* and *P-gp* mRNAs were upregulated, and those of *abcc1, abcc4*, and *abcb4* mRNAs were downregulated (efflux transporter genes) by PSNAP exposure 3. CeO_2_ alone downregulated the expressions of *abcc1, abcc4, abcb4,* and *p-gp* 4. Coexposure with PS reduced the expressions of *abcc1* and *p-gp* by CeO2 5. The expressions of *gadd45a, p53, xrcc2, rad51*, and *trl3* remained unaltered in fish exposed to PSNAP alone 6. Coexposure with CeO_2 and_ PS downregulated *tlr3* and *mt2* gene expressions	Bhagat et al. (2022)
17α-ethinylestradiol (EE2) (2 and 20 μg/L)	Hormone	Zebrafish (*Danio rerio*)	Embryos	PS (47 nm) (1 mg/L	Waterborne (120	1. Upregulation of *gfap* and *α1-tubulin* mRNA expressions (related to the nervous system) by PSNAP exposure alone or coexposed with E2 occurred 2. Genes related to the visual system (rhodopsin, *zfrho;* blue opsin, *zfblue*) were not significantly changed with PSNAP exposure	Chen et al. (2017a)
Homosolate (0.0262–262 μg/L)	Organic compound/UV filter	Zebrafish (*Danio rerio*)	Adults	PS (50 nm) (1 mg/L)	Waterborne (21 days)	1. No effect of PSNAP was observed in the expressions of *sgk1* and *stc* mRNAs in the ovary of adult zebrafish; however, coexposure with homosolate enhanced the expressions of both *sgk1* and *stc* mRNAs in the ovary 2. The expressions of *cyp17a2* and *hsdβ1* mRNAs in the ovary remained unaffected in fish exposed to PSNAP alone; coexposure with homosolate enhanced the expression 3. In the testis, homosolate-induced enhancement in the levels of *hsdβ1, cyp19a1*, and *cyp11a2* mRNAs were attenuated by PSNAP during coexposure 4. In the liver of female fish, PSNAP has no effect on the expressions of *esr2b, vtg1*, or *vtg2* mRNAs, but coexposure with homosolate upregulated the expressions of these mRNAs in a concentration-dependent manner 5. In the liver of male fish, PSNAP exposure alone has no effect on the expressions of *esr2b* or *vtg2* mRNAs; however, coexposure with homosolate upregulated the expressions of these mRNAs	Ye et al. (2024)
Lead (50 μg/L)	Metal	Zebrafish (*Danio rerio*)	Adults	PS (100 nm) (20 and 200 μg/L)	Waterborne (exposed for 3 weeks)	1. In macrophages, immune system-related DEGs (*ctsba, nfkbiab*, and *pycard*) were significantly altered in PSNAP fish than PSNAP + lead groups, and the genes related to MAPK signaling pathways (*hsp70.1, hsp70.2*, and *hsp70l*) were altered in fish exposed only to lead 2. In enterocytes, genes related to glutathione metabolism and cytochrome P450 (*gsta2, gsto 1, gsto2, gpx1a,* and *mgst1.2*) were significantly changed in fish exposed to lead and lead + PSNAP. 3. In B and T cells, upregulation of *hsp70.1, hsp70.*2, and *hsp70.3* expressions occurred in fish exposed to PSNAP, lead, and also in combinations 4. Gene ontology (GO) analysis found several other DEGs such as *gadd45ba, jun, ccl35.2* and *ccl35.2* were altered in macrophages after PSNAP exposure. And in PSNAP + lead groups, *ccr9a*, *cxcr4b*, and *bcl2l10* were altered; however, lead exposure altered *mt2* and *pycard* 5. In enterocytes, GO analysis showed alterations in the expressions of *apoa4a*, *apoa1a,* and *apoea* in fish exposed to PSNAP and lead either alone or in combinations. Moreover, expressions of *npc2* and *prdx1* were altered in fish exposed to lead and lead + PSNAP	Yu et al. (2022a)
Microcystin LR (MCLR) (0.9, 4.5, and 22.5 μg/L)	Antibiotics	Zebrafish (*Danio rerio*)	Adults	PS (70 nm) (100 μg/L)	Waterborne (96 h) 21 days parental exposure (F0) and F1 larvae (120 hpf) were evaluated without exposure	1. The HPT axis and GH/IGF axis genes in the F1 larvae remained unaltered when the parents were exposed to PSNAP alone; however, the expression of the HPT axis genes (*trα, trβ, dio2, dio1, ttr, tg, tshr, nis, crh, pax8,* and *nkx2.1*), except *ugt1ab* and *tpo,* were altered in F1 larvae after parental exposure either to MCLR alone or coexposed with PSNAP. 2. Among the GH/IGF axis genes (*igf2α, igf1, gh, ghrh, ghrα, igf1ra, igf1rβ, igf2β,* and *igf2r*), only *igf1, igf2α,* and *ghrβ* were altered in F1 larvae when the parents were exposed to MCL + PSNAP.	Zuo et al. (2021)
Microcystin LR (MCL) (0.9, 4.5, and 22.5 μg/L)	Antibiotics	Zebrafish (*Danio rerio*)	Adults (male and female)	PS (70 nm) (100 μg/L)	Waterborne (96 h) 3 months	1. The genes related to antioxidant responses (*p38a, p38b, ERK2, ERK3, Nrf2, H O -1, cat1, sod1, gax, JINK1*, and *gstr1*) indicated that PSNAP exposure was unable to produce any significant effect on the expression of these genes 2. MCLR alone enhanced the expressions of *ERK2, ERK3, p38a, Nrf2, gpx1a, gstr1, cat1,* and *sod1* genes in a concentration-dependent manner 3. Coexposure with PSNAP further aggravated the expression of only *Nrf2* gene induced by MCLR	Ling et al. (2022)
Sodium nitroprusside (8 µM)	Inorganic compound/	Zebrafish (*Danio rerio*)	Embryos	PS (50 nm) (20 mg/L)	Waterborne (12 days)	1. The expressions of *Adma, Nos,* and *Pde6d* were significantly higher in PSNAP groups than control or larvae coexposed with SNP; however, the expression of *prkg* was significantly reduced in PSNAP groups than control and SNP coexposed groups 2. The activity of the caspase-3 and the expressions of *bik, bad, bax, bim, bid*, and *bok* were significantly increased by PSNAP exposure, while coexposure with SNP alleviated the process 3. The expression of GPX4, the key protein for ferroptosis, and those of the genes *Slc7a11, Acs14a, Keap1b*, and *Ncoa4* were higher in larvae exposed to PSNAP, while coexposure with SNP alleviated the process 4. The expressions of *tnfα, tgfβ, il-4,* and *il-6* were upregulated by PSNAP, while coexposure with SNP alleviated the process	Chen et al. (2023c)
Sulfamethazine (SMZ) (0.5 and 5 mg/g)	Antimicrobial agent	Marine medaka (*Oryzias melastigma*)	Adults	PS (100 nm) (5 mg/g)	Dietary (30 days)	1. In male fish, histological and biochemical investigations indicate that PSNAP either alone or in combinations with SMZ were unable to alter *sod*, *cat,* and *gpx* transcription in the intestine 2. In female fish, PSNAP alone did not alter *cat* transcription; however, significant reductions in *cat, sod*, and *cat* transcription were observed when coexposed with SMZ	Zhang et al. (2021)
Sulfamethazine (SMZ) (4.62 mg/g)	Antimicrobial agent	Marine medaka (*Oryzias melastigma*)	Adults (580.2 ± 189.5 mg body weight)	PS (100 nm) (3.45 mg/g)	Dietary (30 days) parental (F0) exposure; F1 evaluated after 60 days	1. No significant difference was observed in the expression of the *igf1* gene in the liver of F1 female fish among all four groups 2. In F1 male fish (F0 fed with PS), the expression of *igf1* in liver showed a significant reduction compared to the controls 3. Compared to the PS groups, the expression of the *igf1* gene in the liver of combined exposure (PS + SNZ) group showed a significantly higher level of expression 4. The expressions of *sod* and *cat* genes in female fish (F1) of the SMZ + PS group were significantly higher than those of controls, SMZ, and PS groups; and the expression of *gpx* remained unaltered 5. In male fish, *cat* and *gpx* expressions remained at the same level among the four groups; while that of *sod* was elevated in PS groups than control and SMZ + PS groups	He et al. (2022)
Tetracycline (TC) (5,000 μg/L)	Antibiotics	Grass carp (*Ctenopharyngodon idella*)	Juveniles	PS (80 nm) (20, 200, and 2000 μg/L)	Waterborne (7 days)	1. Lesions in gills and intestine 2. Enhanced the oxidative-related changes in the liver and intestine 3. Upregulation of *MMP2, MMP9* and *IL8* expressions in the liver and intestine of the co-exposed fish in a concentration-dependent manner	Liu et al. (2022a)
Tris (1,3-dichloro-2-propyl) phosphate (TDCIPP) (0.47, 2.64, or 12.78 μg/L)	Flame-retardant	Zebrafish (*Danio rerio*)	Adults	PS (54.5 ± 2.8 nm) (10 mg/L)	Waterborne (120 days); evaluated F0 and F1 larvae (without exposure)	1. In the brain of female adult fish (F0), the transcription of corticotropin-releasing hormone (*crh*) was upregulated in a nonlinear fashion in fish exposed to TDCPP either alone or in combinations of PSNAP. However, the transcription of *tshβ* remained unaltered in all treatment groups when compared with that in controls 2. In the liver of female fish (F0), the expressions of thyroglobulin (*tg*) and uridine diphosphate glucuronosyltransferase (*ugt1ab*) were upregulated in fish exposed to TDCPP alone or in combination with PSNAP when compared with controls. Moreover, the expressions of deiodinase 1 (*dio1*) and transthyretin (*ttr*) were downregulated, and the expression of the deiodinase 2 (*dio2*) gene was upregulated in fish exposed to TDCPP either alone or in combination with PSNAP in a nonlinear fashion when compared with controls 3. In the brain of male F0 fish, the transcription of *crh* and *tshβ* increased only in the fish exposed to TDCPP and PSNAP when compared with controls 4. In the liver of male fish, the transcription of *tg* and *ugt1ab* genes was upregulated in fish exposed to TDCPP alone or in combinations with PSNAP when compared with the controls in a nonlinear fashion. Moreover, the expression of *trβ* remained unaltered in all the experimental groups, while *trα* expression in the liver of male fish (F0) was upregulated when exposed to TDCIPP alone or in combinations with PSNAP in a nonlinear fashion when compared with controls. In addition, a significant downregulation of the *ttr* expression was observed in the liver of male fish when exposed to TDCIPP either alone or in combinations in a nonlinear fashion when compared with controls 5. In F1 larvae, relative to control, the expressions of *crh, tg*, *trα, tshβ,* and *ugt1ab* were enhanced in coexposure groups in a concentration-dependent manner; moreover, the expression of *dio2* was upregulated in TIDCIPP-exposed larvae, and coexposure further enhanced the expression when compared with controls	Zhao et al. (2021)
Vitamin D (280 and 2,800 IU/kg body weight)	Vitamin	Zebrafish (*Denio rerio*)	Adults	PS (80 nm) (15 and 150 mg/L)	Dietary (for 21 days)	1. Nonlinear increase in the gene hydroxy-3-methylglutaryl-coenzyme A (*hmgcra*), sterol regulatory element-binding protein (*srebp1*), diaceylglycerol aceyltransferase 1b (*dgat1b*), acetyl coenzyme A carboxylase (*acc*), and carbohydrate response element-binding protein (*cvhrebp*) by PSNPs in the liver; however, the expression of carnitine palmitoyl transferase 1 (*cpt1*) decreased significantly with PSNAP exposure	Li et al. (2023a)
Vitamin D (280 and 2,800 IU/kg body weight)	Vitamin	Zebrafish (*Denio rerio*)	Adults	PS (80 nm) (15 and 150 mg/L)	Waterborne (21 days)	1. Vit D reduced the accumulation of PSNAP in the intestine 2. The blood–brain barrier basement membrane damage by PSNAP was less when coexposed with vit D 3. PSNAP exposure induced anxiety-like behavior, while vit D alleviated the process 4. Vit D coexposure increased 5-HT content in the brain 5. PSNAP exposure induced vacuolization in intestinal goblet cells and mitochondria and disorder in the arrangement of intestinal villi, while coexposure with vit D alleviated the process 6. The SOD activity in the intestine increased by PSNAP exposure in a concentration-dependent manner; coexposure with vit D alleviated the process 7. The MDA content increased in fish exposed only to 15 μg/L PSNAP; vit D alleviated the process	Teng et al. (2023)
ZnO (760 μg/L)	Metal oxide	Grass carp (*Ctenopharyngodon idella)*	Juveniles	PS (23.03 ± 0.266 nm) (760 μg/L)	Waterborne (72 h)	1. Affected the response on mirror tests (longer immobility time and shorter interaction with their images) 2. Stimulated the antioxidant activity of the brain 3. Increased AChE activity in the brain 4. Induced DNA damage in erythrocytes	Estrela et al. (2021)

Among the 114 selected articles, we further screened by focusing only on studies on NAPs that are ≤100 nm in diameter/size; therefore, studies made focusing on plastic sizes >100 nm (15 articles) were excluded during evaluation (Table 3). Among these 15 articles, two articles, Monikh et al., 2022 (PE, PPP, PS, and PVC), and Tamayo-Belda et al., 2023 (LDPE, PLA, PPP, and PS), focused on more than one plastic type and included together in one article. Moreover, their studies examined various sizes of plastics, belonging to both NAPs and MIPs. Therefore, these two articles were included in both inclusion (Tables 2, 4) exclusion (Table 3) tables. Wang L. et al. (2023) did not mention the plastic types used for zebrafish embryos, although the size of the NAP was 100 nm. Therefore, we did not consider Wang L. et al. (2023) for review (Table 3). In addition, 26 articles included both MIP (>100 nm) and NAP (≤100 nm) in their investigations (Table 4). During the review process, we considered these 26 articles and focused only on the studies carried out on NAPs and excluded the studies carried out on MIPs (Table 4). Moreover, Tamayo-Belda et al. (2023) measured the diameter of the plastics (LDPP, PLA, PPP, and PS) every day during embryo development (4–96 hpf), and the diameter of the plastic particle was widely variable (>100 nm) within the days of exposure. However, in case of PS, the diameter of the plastic particle during the exposure (4 hpf) was 91 nm, which was below the exclusion limit of the MIPs (≤100 nm) followed in this study. In addition, for LDPE, the diameter of the plastic particle is 91 nm only on 4 dpf (96 hpf) of development (Table 4). We, therefore, consider PS and LDPE as NAPs during evaluation. Furthermore, three articles, namely, Manuel et al. (2022) (studies on PMMA and PS on zebrafish embryos); Monikh et al. (2022) (studies on PPP, PE, PS, and PVC on zebrafish embryos); and Tamayo-Belda et al. (2023) (studies on LDPE, PLA, PPP, and PS on zebrafish embryos), studied multiple plastic particles and described the results together in one article. Elizalde-Velazquez et al. (2020) studied the effects of PS on fathead minnows using two methods of exposure (IP and trophic transfer) and described the results together in one article. Moreover, we confined our search to *in vivo* studies and excluded *in vitro* studies (Greven et al., 2016). However, Greven et al. (2016), used two different sizes of PS (158.7 nm and 41 nm sizes) on fathead minnows and described the results together in one article. Therefore, 15 (13 + 2) articles, including studies by Monikh et al. (2022) and Tamayo-Belda et al. (2023), were excluded (Table 3), 26 articles were partly excluded from the review, and finally, 101 (99 + 2) articles were selected for NAP evaluation (Tables 5–9).

## 3 Results

In laboratory studies, fish at different developmental stages (embryos, larvae, juveniles, and adults) were used for the assessment of NAP toxicity (Table 2). In embryos, NAPs were accumulated/agglomerated on the chorion after exposure (waterborne) and depending on the size of the NAPs and the pore diameter of the chorion (in zebrafish, the size of the chorion was 200–700 nm in diameter, Chen et al., 2020), NAP particles crossed the barrier and entered into the body of the developing embryos and gradually accumulated on different organs over time. In some experiments, NAPs were directly injected inside the eggs (Sokmen et al., 2020; Zhang et al., 2020). However, in larvae, juveniles, and adults, the fish when exposed to NAPs through waterborne mode, trophic transfer, or through diet entered inside the body through the mouth, gills, and skin. In a few cases, NAPs were directly administered through injections (Elizalde-Velazquez et al., 2020).

### 3.1 Effects of NAPs on fish

#### 3.1.1 Polyethylene

Polyethylene (PE) is also known as polythene, is a synthetic resin and the most commonly used plastic in the world. It can only generate nonspecific van der Walls interactions (Geum and Yeo, 2022). Our literature search found only two fish species; common carp (one article) and zebrafish (four articles on PE and one article on LDPE; three on embryos and two on adults) were used to evaluate the toxic potential of PE/LDPE as NAPs. Moreover, two more studies were conducted on PE where the particle size was >100 nm (Sun et al., 2021; Khan and Ali, 2023), and were therefore excluded from evaluation. The 96 hpf no observed adverse effect level (NOAEL) found on the toxicity of PE in zebrafish embryos was 0.05 mg/L (hydrodynamic size 191.10 ± 3.13 nm) (Sun et al., 2021). Zebrafish adults exposed to pristine polyethylene (76,740 ± 14,070 nm) were able to excrete small PE (5,920 ± 4,960 nm) within 24 h of exposure (Supplementary Table S1), which indicates that PEMIP enters the gut, metabolizes to smaller fragments, and is excreted in the fecal material (Khan and Ali, 2023).

##### 3.1.1.1 Common carp

In juvenile common carp (*Cyprinus carpio*), PE significantly decreased the enzyme activities (AChE and MAO) and NO content in the brain (Hamed et al., 2022) and caused histological damages, indicating varying degrees of necrosis, fibrosis, changes in blood capillaries, tissue detachment, edema, degenerated connective tissues, and necrosis of large cerebellar neurons and ganglion cells (Tables 2, 5, 6, Supplementary Table S1). In eyes, necrosis, degeneration, vacuolation, and curvature in the inner layer were observed after PE exposure.

##### 3.1.1.2 Zebrafish

Both embryos and adults of zebrafish were used for the evaluation of PE toxicity (Tables 2, 5, 6; Supplementary Table S1). Zebrafish embryos within 6 hpf were exposed to PE (50 nm; 3 × 10^−10^/L) for 24 h or to LDPE (91–342 nm) for 96 h, and mortality and development were evaluated until 4–5 dpf (Tables 2, 5, 6; Supplementary Table S1). It was observed that PE did not induce mortality; however, delayed hatching was observed, and the hatched embryos were normal, although the larval body length was reduced when compared with that of controls (Monikh et al., 2022). The zebrafish larvae (120 hpf) exposed to LDPE during development showed slight locomotor activity during the light phase (Tamayo-Belda et al., 2023). Zebrafish adults were exposed to PE (70 nm) at a concentration of 20 μg/mL for 21 days (Tables 2, 5, 6; Supplementary Table S1), and the oxidative stress and AChE enzyme activity in the gill, gut, and liver of fish on 7, 14, and 21 days of exposure (Li R. et al., 2023) were investigated. Moreover, gut dysbiosis was also analyzed. Organ-dependent oxidative damage induced by PE was observed after chronic exposure. Insignificant differences in the neurotoxicity (inhibition of AChE activity) and dysbiosis of gut microbiota were also observed in fish exposed to PE (Li R. et al., 2023). The effects on GST, GSH, CAT, LPO, and SOD showed that PE induced organ-specific oxidative damage in the gill, gut, and liver (Li R. et al., 2023).

Taken together, it was observed that PE (50 nm) was able to reduce the length of zebrafish larvae when the embryos were exposed only for 24 h (Monikh et al., 2022).Juvenile common carp exposed to PE (<100 nm; 15 mg/L) for 15 days had disrupted brain structure (histology) and function (AChE and MAO activities and NO contents), while in adult zebrafish, PE (70 nm; 20 μg/L for 21 days) induced organ-specific oxidative stress (gill/gut/liver), inhibited AChE activity, and induced dysbiosis in gut bacterial communities (Li R. et al., 2023). Therefore, although the study is limited only to two fish models and studies on gene expression are lacking, PE was found to induce toxicity in fish, depending on the developmental stages, concentration, sizes, and the duration of exposure, as well as in different organs of the fish (Table 6).

### 3.2 Polyethylene terephthalate

Polyethylene terephthalate (PET) is one of the most used plastic polymers, particularly for containers (container for food, drinks, and plastic bags), owing to its transparency, flexibility, and innocuity (Dhaka et al., 2022). It is also used in textiles and as parts of automotives and electronics (Gwada et al., 2019; Dhaka et al., 2022). PET particles have been found in ground water, drinking water, soils, and sediments in the air (Dhaka et al., 2022; Jiang et al., 2022; Lin et al., 2022; Zhang H. et al., 2022). The hazardous effects of PET in the form of nanoparticles (PETNAPs) in marine organisms such as amphipods, copepods, and fish have been studied (Heinder et al., 2017; Ji et al., 2020). PETNAPs have raised severe concerns regarding potential danger and risks for nature and human wellbeing (Dhaka et al., 2022; Zhang H. et al., 2022). Studies on human cell culture showed that PETNAPs at a higher concentration have inhibitory effects on the cell viability (Margi et al., 2021; Zhang H. et al., 2022; Villacorta et al., 2022), and the interaction of PETNAPs with different contaminants (Hg^2+^, glyphosate, and levofloxacin) can significantly change the cell physiology (Margi et al. (2021)). Using human lung carcinoma cell culture, Zhang H. et al (2022) have shown that PETNAP increased levels of reactive oxygen species (ROS), which may affect mitochondrial potential. A comprehensive system-level tracking of the toxicity pathways affected by PETNAPs is necessary to understand the toxicity mechanisms of PETNAPs. Our literature search found that only zebrafish embryos were used (two articles) to evaluate the toxic potential of PETNAPs in fish (Bashirova et al., 2023; de Souza Toedoro et al., 2024).

#### 3.2.1 Zebrafish

Zebrafish embryos (6 hpf and 72 hpf) were exposed to PET (70 ± 5 nm and 68.06+ nm) until 96–120 hpf (Bashirova et al. (2023) or 6 days (de Souza Toedoro et al. (2024) at concentrations ranging from 0.5 to 200 mg/L (Tables 2, 5, 6; Supplementary Table S1). PET was accumulated in liver, kidney, and intestine of the larvae (Table 5), and its exposure reduced the survivability and hatching of the embryos in a concentration-dependent manner. The heart rates remained unaltered. The locomotor activity of the larvae in the dark phase was reduced in a concentration-dependent manner. Quantitative analysis of the metabolites indicated a significant decrease in acetate, glucose, alanine, leucine, isoleucine, valine, glutamate, cystine, glycine, and GSH levels; however, a significant increase was noticed in lactate, choline, glycerophosphorylcholine and ethanolamine, tryptophan, phenylalanine, tyrosine, free fatty acids, and cholesterol levels (Bashirova et al., 2023). Higher levels of ROS were generated in the intestine, liver, and kidney region of the larvae (Bashirova et al., 2023). In contrast to the study, de Souza Toedoro et al. (2024) observed that PET accumulated on the surface of the chorion in a concentration-dependent manner, and no effect on the mortality and hatching of the embryos was observed. The heart rates of the treated embryos at 48 hpf increased significantly in a concentration-dependent manner, and the length of the hatched larvae did not change significantly; also, no effect on locomotor activity was observed. The interocular distance reduced significantly in embryos exposed to PET. Moreover, spontaneous tail coiling was diminished by PET exposure. No significant effect was observed in lipid peroxidation or total antioxidant capacity during embryo–larval development (de Souza Toedoro et al., 2024). Therefore, despite the differences between the two studies, PET was able to modulate the embryonic development as well as the behavior of the zebrafish larvae; however, there are few studies on the genotoxicity.

### 3.3 Polymethylmethacrylate

Polymethylmethacrylate (PMMA), is often used in electronic equipment and prosthetics, and 0.26 million tons were used in Europe in 2019 (Plastic Europe, 2022). However, the effect of PMMA on aquatic animals is poorly understood (Manuel et al., 2022). A recent study showed that 40-nm PMMA nanoparticles, at higher concentrations, impaired survival and growth in tadpoles and induced deformities (Venancio et al., 2022). In the marine fish, *Sparus aurata*, 40-nm PMMA nanoparticles demonstrated the ability to alter the antioxidant status and lipid metabolism pathways and induced genotoxic effects on red blood cells (Brandts et al., 2021). In our literature search, only zebrafish embryos (one article) were used to evaluate the toxic potential of PMMA in fish (Manuel et al., 2022).

Zebrafish embryos (2 hpf) were exposed to PMMA (32 nm; 0.001–100 mg/L) until 96 hpf (Tables 2, 5, 6; Supplementary Table S1), and the larvae (96 hpf) were used for evaluation of mortality, hatching, and pericardial edema (Manuel et al., 2022). The swimming behavior of the larvae was assessed after 120 hpf. It was observed that PMMA at the highest concentration induced mortality and delayed hatching of the embryos. No significant effect on the swimming behavior of the larvae was observed. AChE activity did not show any significant alterations, except for the larvae exposed to a concentration of 0.01 mg/L, in whom the activity significantly decreased when compared with controls. Among the antioxidant enzymes, GST did not show any significant alterations; however, GPX activity was enhanced only in larvae exposed to 10 mg/L PMMA. CAT activity, though nonlinear, was found to be enhanced in larvae exposed to concentrations of 0.001, 0.1, and 10 mg/L. Concerning energy reserves, no significant effect in terms of glycogen was observed (Manuel et al., 2022). Although the concentrations limited the toxic potential of PMMA in zebrafish, the effects were mediated through ROS and oxidative stress.

### 3.4 Polypropylene

Polypropylene (PPP) is one of the most widely used plastics, with the application ranging from food packaging to use as automotive parts, and it is also one among the most significant components of personal protective equipment such as masks, the use of which has increased since the COVID-19 pandemic (Aragaw, 2020; Patricio Silva et al., 2021; Vanapalli et al., 2021). A considerable amount of PPP waste has accumulated in the environment and is continuously converted to PPPMIPs by action of external factors such as UV radiation, oxidation, and biofilms (Min et al., 2020). PPPMIPs have been detected in the gastrointestinal tracts of sea turtles of the Atlantic Coastlines of Florida (White et al., 2018). In zebrafish embryos, PPP are internalized by ingestion and distributed in the intestine and eventually excreted (Lee et al., 2022). Adult zebrafish were exposed to the micro/nanoplastics extracted from food-grade PPP nonwoven bags for 2 and 14 days, and the activities/contents of several oxidative-stress related biomarkers (ROS, GSH, SOD, CAT, and MDA) were modulated in the gill and liver of the exposed fish (Li J. et al., 2023). Additionally, a recent study reported that PPPMIPs were released from infant feeding bottles during formula preparations (Li et al., 2020). Moreover, in a study on human-derived cell and animal models (zebrafish and nematodes), PPPMIPs induced cytotoxicity, proinflammatory cytokine activity, oxidative stress, and intestinal damage (Lei et al., 2018b; Hwang et al., 2019). Therefore, it was suggested that the preparation and labeling techniques for PPPNAPs as model plastic nanomaterials are important for enhancing toxicological and biodistribution studies (Cassano et al., 2021). Our literature search found that two fish species tilapia juveniles (one article) and zebrafish embryos (three articles) were used to study the toxic potential of PPPNAP; however, two of the articles (Lee et al., 2022 and Tomayo-Belda et al., 2023) used PPPMIPs.

#### 3.4.1 Tilapia

Tilapia (body weight 10 ± 1 g; length 13 ± 1 cm) were exposed to PPPNAP (100 nm) in water for 21 days at three different concentrations (1, 10, and 100 mg/L), and the liver was used for metabolomics analysis (Tables 2, 5, 6; Supplementary Table S1). It was observed that the body weight and the hepatosomatic index (HSI) of the fish did not change after 21 days of exposure to PPP (Wu et al., 2023). However, the plastics induced significant effects on glycerophospholipid, arginine, and proline metabolism and on aminoacyl-tRNA biosynthesis (Wu et al., 2023).

#### 3.4.2 Zebrafish

Embryos of zebrafish within 6 hpf were exposed to 3 × 10^10^ particles/L of PPP (50 nm) for 24 h (Tables 2, 5, 6; Supplementary Table S1). It was observed that although there was no induction in the mortality among the embryos, the hatching was delayed, and the larval length was reduced significantly. Moreover, 18% of the larvae exposed to PPP showed a curved spine (Monikh et al., 2022).

### 3.5 Polystyrene

Polystyrene (PS) plastic used in producing Styrofoam, which is used in food containers and packaging products (Kik et al., 2020). It is one of the most produced plastic polymers in the world; in 2019, there was a demand of 1.58 million tons alone in Europe (Manuel et al., 2022). Due to its significant use, often in single-use products associated with food packing, PS is the most detected plastic in the environment (Fahrenfeld et al., 2019) and the most studied plastic on aquatic organisms (Lu et al., 2016; de Sa et al., 2018; Peng et al., 2024). In addition, it is one of the most abundantly found plastics in the marine environment (Pitt et al., 2018b). Among the plastic polymers, PS has an intermediate density (1.05 g/cm^3^), with a value close to density of water (1–1.03 g/cm^3^); this makes PS plastics behave differently in waters of different salinity and thus become bioavailable for aquatic organisms, from surface waters to bottom waters or in sediments (Earni-Cassola et al., 2019). PS has a relatively higher adsorption capacity than PE (Geum and Yeo, 2022). The 96-h LC_50_ as determined in tooth carp (*Aphaniops hormuzenis*) was 19.3 mg/L (Saemi-Komsari et al., 2023). PSNAP produces ROS, which results in oxidative stress-mediated toxicity (Schirinzi et al., 2017; Lei et al., 2018a; Eom et al., 2020; Kim and Rhee, 2021). Our literature search showed that PS is the only plastic for which almost all the selected fish species were studied and the highest number (104) of articles (∼89%) were considered for review (Figure 1).

#### 3.5.1 Carp

The search terms nanoplastics, PS, and carp identified articles on carp (one article), grass carp (five articles), silver carp (one article), and tooth carp (one article). Our search indicated that among all these carps, the toxic effects of PS were evaluated on embryos, larvae, and juveniles of grass carp and on adults of carp, silver carp, and tooth carp. Moreover, the size (20–8,000 nm), concentrations (5 μg–200 mg/L), duration (2 hpf–20 days), and the modes of exposure (waterborne and dietary) were widely variable (Tables 2, 5, 6). It was observed that in embryos (grass carp), the accumulation of NAPs was mostly on the chorion; in larvae (grass carp) in the intestine and nose area; in juveniles (grass carp) in the gut, intestine, blood, liver, and brain; while in adults (carp, silver carp, and tooth carp), PS was accumulated in the gill, gut, intestine, liver, heart, muscle, and skin (Table 5). The studied effects were mostly focused on toxicological endpoints (Table 6), while genotoxic effects were also investigated (Table 7).

PS (80 nm) was unable to induce any disorder in heart rates or mortality in grass carp embryos (Zhang C. et al., 2022), while in juveniles, PS increased liver weight (HSI), induced DNA damage in erythrocytes, lesion in the gills and intestine, and histological damages in the gut and brain (Table 5). Moreover, the overall antioxidant activities and LPO contents in the brain (CAT, GST, GPx, and SOD activities and GSH and MDA contents) increased, while NO contents remained unaltered. The enhancement of AChE activity in the brain did not affect locomotory movements (Table 6). Moreover, in the intestine of juveniles (grass carp), the expressions of several immunomodulatory genes (*IL-6, IL-8, IL-10, IL-1β, TNF-α, and INF-γ2*) were upregulated (Li Z. et al., 2024). In adults, PS induced apoptosis, inhibited antioxidant capacity, and increased the protein contents of TL4 and NOX2, which resulted in induction of apoptosis and myocardial injury (Wu et al., 2022). Moreover, the diversity and richness of gut microbiota increased after PS exposure (Zhang et al., 2024a). Taken together, despite the variations in the dose, duration, mode of exposure, and developmental stages, PS was found to be toxic to carps, and PS accumulation in the brain and induction of oxidative stress resulted in immunomodulatory effects in the intestine that disrupted the gut microbial communities.

#### 3.5.2 Fathead minnows

The effects of PS were studied in fathead minnows both *in vitro* and *in vivo*. For *in vitro* effects, neutrophils were collected from adult fish and exposed to PS (41.0 nm diameter) either for 1 h (100 μg/L) or for 2 h at four different concentrations (0.025, 0.05, 0.1, and 0.2 μg/L) (Supplementary Table S1). PS induced degranulation of primary granules, and neutrophil extracellular traps were released in a concentration-dependent manner (Greven et al., 2016), even though nonlinear. However, oxidative burst was less affected.

Adult male fish were exposed to PS (50 nm) either by IP injection (0.1 mL of 5 μg/L) or by trophic transfer [fed PS-exposed (5 mg/L) daphnia to the experimental fish] and sacrificed after 48 h (Elizalde-Velazquez et al., 2020). PS was accumulated in the liver and head kidney of the exposed fish and regulated the expressions of four immune-related genes (*ncf2, nox2, mst1*, and *c3*) (Table 7; Supplementary Table S1). The expressions of *mst1* and *c3* were upregulated in fed animals and downregulated in injected fish (Elizalde-Velazquez et al., 2020). Moreover, the expression of *ncf2* was downregulated and that of *nox2* remained unaltered in both the liver and head kidney of fish exposed to PS either by injection or by feeding (Elizalde-Velazquez et al., 2020). In the head kidney, significant downregulation was observed in *ncf2* expression in both methods of exposure, while *mst1* expression was downregulated in injected fish and remained unaltered in fed ones. *C3* in the head kidney was downregulated in fed fish and remained unaltered in PSNAP-injected fish (Tables 5, 6; Supplementary Table S1). Therefore, modes of exposure of PS to the fish played a significant role in the expression of immunomodulatory genes in fathead minnows.

#### 3.5.3 Medaka

The search terms, nanoplastics, PS, and medaka identified 14 articles belonging to Chinese rice fish (one article), Hainan medaka (one article), Japanese medaka (two articles), and marine medaka (10 articles). Among these fish (medaka), embryos of marine medaka (Chen et al., 2022; Chen et al., 2023 Y.; Yu et al., 2023), larvae (9 dph) of Japanese medaka (Zhou et al., 2023b) and marine medaka (Kang et al., 2021; Li X. et al., 2024), juveniles of marine medaka (Li Y. et al., 2023; Li X. et al., 2023) and adults of Hainan medaka (Gao D. et al., 2023), Japanese medaka (Zhou et al., 2023a; Zhou et al., 2023b), and marine medaka (Zhang et al., 2021; He et al., 2022; Wang F. et al., 2023) were used for evaluation of PS toxicity. Accordingly, in these studies, the sizes (50 nm–45 µm or 50–45,000 nm), concentrations/doses (5.5 × 10^−12^ mg/L −5 mg/L), modes of exposure (waterborne, trophic transfer, and dietary), and duration (24 h–120 dph) of exposure with PSNAP were widely variable (Tables 2, 5). Moreover, the accumulation and the effects of PSNAPs in embryos (yolk sac, GI tract, intestinal villi, liver, and heart), larvae (gut, intestine, liver, muscle, and gonads), juveniles (intestine), and adults (gills, intestine, gut, liver, ovary, and testis) were dependent on the developmental stages of the fish (Tables 2, 5). Although the studies were focused on toxicological endpoints (Table 6), investigations on genotoxic effects (Table 7) as well as intergenerational effects have also been done. Moreover, because the diameter of the exposed PS particle was >100 nm, we have excluded the studies carried out by Zhang YT. et al. (2024) on adults of marine medaka in this review (Table 3).

The embryos of marine medaka with PSNAP (50 nm; 55 μg/L) exposure exhibited reduced heart rates (6 dpf), induced mortality, and reduced larval body length (21 dpf); also, deformities in craniofacial structures and abnormalities were also observed in the histology of the liver and heart of the larvae (21 dpf) (Table 6). Moreover, embryos of marine medaka were exposed to PS-NH_2_ (80 nm) and PS-COOH (80 nm) at 10 μg/L concentration in regular sea water (pH 8.2) or in acidified sea water (pH 7.4) for 10 days and allowed hatching under a PS-free environment in sea water (Chen Y. et al., 2023). It was observed that both PS-NH_2_ and PS-COOH accumulated in the gut and intestinal villi of the larvae and induced toxic effects (mortality, hatching, heart rates, morphological abnormalities, malformations, and swimming speed and distance) during embryo–larval development (Chen Q. et al., 2023). PS-NH_2_ showed greater toxicity than PS-COOH; however, in acidified conditions (pH 7.4), the toxicity of PS-COOH was greater than that of PS-NH_2_ (Chen J. et al., 2023).

PSNAP has no effect on the length, weight, and eye diameter of the fish larvae exposed to PSNAP. Moreover, the oxidative stress (ROS content and the activities of CAT, SOD, and GST) induced by PSNAP exposure exhibited stronger effects and disruption of gut microbiota (Kang et al., 2021). In juveniles (2-month-old marine medaka), PSNAP (100 nm; 5 mg/L, 30 days) was unable to induce histopathological changes in the intestine; however, the mucus content was slightly increased, and the number of intestinal goblet cells significantly decreased with alteration in the gut microbial community (Li X. et al., 2023).

Exposure to PSNAP (80 nm; 250 μg/L) for 7 days in fasting conditions in Hainan medaka adults damaged the gills (fusion of the gill lamellae), liver (appearance of eosinophilic vesicles and vacuolization), and intestine (erosion of intestinal villi) (Table 6). Moreover, the oxidative enzymes (CAT and SOD) and the LPO content (MDA) were altered in these organs (Gao X. et al., 2023). The gut microbiota was also affected by PSNAP exposure. In adults of Japanese medaka, PSNAP induced concentration-dependent mortality and intestinal damage by enhancing the activities of trypsin and chymotrypsin and reducing the amylase activity (Table 6). Moreover, intestinal lipase contents tended to increase, and alkaline phosphatase contents decreased in a concentration-dependent manner (Zhou et al., 2023a; Zhou et al., 2023b). The gut permeability was also disrupted by PSNAP exposure, with enhancement in the diamine oxidase activity and decrease in the d-lactate contents. The oxidative stress-related enzyme (CAT) and MDA contents in the intestine were enhanced, and that of SOD was suppressed after PSNAP exposure. In contrast, the antioxidant enzymatic activities (SOD, CAT, GPx, and LZM) and the MDA content in the gonads were altered in a nonlinear fashion (Zhao et al., 2021). Gut microbial community diversity exhibited a decrease, and changes were observed in the composition (Zhou et al., 2023b). In gonads, a concentration-dependent inhibition in spermatogenesis and oogenesis was observed in Japanese medaka exposed to PSNAPs for 3 months (Zhou et al., 2023a).

Adults of marine medaka were exposed to PSNAPs (70 nm) by trophic transfer (fed with rotifers exposed to PSNAPs), which indicated accumulation in the gut of the fish. Moreover, long-term exposure (90 days) through trophic transfer not only damaged the tissues, including the intestine, liver (induced inflammation), muscle (decreased nutrient contents), and gonads (disrupted spermatogenesis and oogenesis) but also disrupted the gut microbial community. Moreover, reduction in fertility, inhibition of hatching, and disruption in the growth of the offspring were also observed (Li X. et al., 2024). Gene expression analysis indicated that the expressions of *il6, il8, il1b, il10,* and *tnf*, in the liver and intestine of the PSNAP (70 nm)-fed fish were upregulated, and in the liver, the expressions of lipid synthesis-related genes (*fasn, srebf1*, and *pparg*) and lipid transport-related genes (*cetp*, and *ldlr*) were upregulated and those of the lipid degradation-related genes (*atg1, ppara*, and *aco)* were downregulated (Li X. et al., 2024). The gene expressions of the Toll-like receptor 4 (TLR4) pathway (*irf3, irak4, traf6,* and *tbk1*) in the liver showed a trend of upregulation, while those in muscle development-related genes (*myog, myod, mstn, myf5,* and *fgf6b*) were downregulated after PSNAP exposure by trophic transfer (Table 6).

Marine medaka adults fed 5 mg/g (actual concentration was 3.45 mg/g) PSNAPs (100 nm) for 30 days and depurated for 21 days showed sex-specific dysbiosis in the gut microbial community (male fish were more effective than female fish), and during depuration, male fish recovered quickly than female fish (He et al., 2022). Moreover, the eggs produced at the 30th day of exposure by the parents (F0) were reared for 60 days without any additional treatments (F1), and the intergenerational effects on growth, gut microbial content, and the hepatic gene expressions related to oxidative stress (*cat, sod*, and *gpx*) and *igf1* were evaluated (He et al., 2022). It was observed that parental exposure to PSNAP significantly reduced the body weight of F1 male fish and decreased the hepatic *igf1* and decreased *sod* mRNA content than controls (F1); in female fish, no alteration in the hepatic *igf1* mRNA level was observed (Tables 7). The composition of the gut microbiota of the F1 fish was altered when the parents (F0) were fed with PSNAP. The mRNA expression pattern of *sod, cat*, and *gpx* remained unaltered in female F1 fish (He et al., 2022). Adults of Chinese rice fish were exposed to PSNAPs (57.29–60.39 nm) either directly (5 mg/L) for 7 days or through trophic transfer by feeding daphnia (*Daphnia magna*), which consumed algae (*Chlamydomonas reinhardtii*) exposed to PSNAPs (Chae et al., 2018). Moreover, fertilized eggs laid by the parents during direct exposure periods were further exposed to PSNAPs (5 mg/L) for 24 h, and the unhatched embryos (144 hpf) and larvae (0 dph) were evaluated for accumulation of the PSNAPs (Supplementary Table S1). It was observed that both in trophic transfer and direct exposure, accumulation of PSNAPs was observed in the gut of the parents; in larvae (0 dph) and embryos (144 hpf), the PSNAPs were accumulated on the yolk sac. The locomotor activity of the larvae was also affected by PSNAP exposure. It was observed that the total distance covered during swimming tended to increase; however, the area traveled tended to decrease by the larvae (Chae et al., 2018).

#### 3.5.4 Rainbow trout

The search terms nanoplastics, PS, and rainbow trout identified two articles focused only on juvenile fish (Supplementary Table S1). Moreover, among these studies, in one study (Clark et al., 2023a), the diameter of the exposed PS particle was >100 nm, which was excluded from this review (Table 3). Juvenile rainbow trout (5–10 g bodyweight) were exposed to PSNAP (35 ± 8 nm) through diet (5.9 μg/kg food; fed 2% of body weight) for 3, 7, and 14 days (Table 5), and it was observed that PSNAPs were accumulated in the hind intestine after 3 days and transferred to the liver after 7 days of exposure (Clark et al., 2023b).

#### 3.5.5 Tilapia

The search terms nanoplastics, PS, and tilapia identified seven articles focusing on of two species, *Oreochromis mossambicus* (Mozambique tilapia, one article) and *Oreochromis niloticus* (Nile tilapia, six articles*).* Moreover, our literature search did not find any study on embryos or on adult tilapia; only larvae (Pang et al., 2021 Zheng and Wang, 2024; Zheng et al., 2024) and juveniles (Ding et al., 2018; 2020; Hao et al., 2023; Wang W. et al., 2023) were used in the studies. Although the mode of exposure of PSNAP was waterborne, the sizes (80 nm–90 µm or 80–90,000 nm), concentrations/doses (1 µg–100 mg/L), and duration (7–28 days) of exposure were highly variable (Tables 2, 5). Moreover, the whole larvae of Mozambique tilapia and gill, stomach, liver, intestine, muscle, and brain of Nile tilapia were considered targets of PSNAP toxicity. Although the studies were focused on toxicological endpoints (Table 6), investigations on genotoxic effects (Table 7) have also been done. Because the diameter of the exposed PS particle was >100 nm, we have excluded the studies conducted by Ding et al. (2020) on juvenile tilapia in this review (Table 3).

The gill of Nile Tilapia larvae consisted of twelve types of cells (Zheng and Wang, 2024; Zheng et al., 2024). After PSNAP exposure (80 nm, 100 μg/L, 28 days) differential damage in the gill tissue was induced, with a 22% decrease in cell types including endothelial cells, fibroblasts, macrophages, natural killer cells, and B-cells; only H^+^-ATPase-rich cells exhibited significantly higher cell counts (Zheng and Wang, 2024). The oxygen consumption, gill histopathology, and transcriptomic and metabolomics analyses of the genes in gills indicate that PSNAP exposure induced severe respiratory distress in tilapia (Table 6).

The larvae of Mozambique tilapia were exposed to PSNAP (100 nm, 20 mg/L) for 7 days and depurated for a week (Table 5). Transcriptomic and metabolomic analyses identified a total of 203 significantly changed metabolites and 2,152 differentially expressed unigenes after PSNAP treatment and recovery (Pang et al., 2021). Moreover, the study indicated that short-term exposure to PSNAPs induced abnormal metabolism of glycolipids, energy, and amino acids (Pang et al., 2021). Transcriptomic results suggested that PSNAP exposure caused signaling disorders, particularly the pathways associated with cell adhesion molecules (CAMs), neuroactive ligand–receptor interaction, and extracellular matrix (ECM)–receptor interactions. A series of differentially expressed genes related to CAMs revealed that PSNAP exposure might have caused early inflammatory responses (Pang et al., 2021). Moreover, the biological processes of “detection of chemical stimulus involved in sensory perception of smell” are affected by PSNAP exposure (Pang et al., 2021).

Juvenile Nile tilapia were exposed to PSNAPs (86–100 nm, 1–1,000 μg/L for 7–21 days), and some of them were under depuration for a week (Hao et al., 2023, Wang et al., 2023b). It was observed that PSNAP exposure did not induce any mortality or mechanical injury in the body and produced insignificant effects on feeding or swimming behavior. Moreover, PSNAP was internalized and accumulated in the gill, gut, intestine, liver, brain, and muscle tissues of the fish (Ding et al., 2018; Hao et al., 2023; Wang W. et al., 2023). The intestine exhibits severe damage in the mucosal layers, which leads to an impact on the microbial community. The intestinal injury was related to the induction of inflammation (upregulation of *tnfα, il1β,* and *il8* and downregulation of *il10*) and oxidative stress (enhanced activities of SOD and GPx and MDA content) (Hao et al., 2023). In the liver, PSNAP induced hepatic steatosis, modulated the inflammatory response, and disrupted liver functions (Wang W. et al., 2023). The oxidative stress induced in the liver showed enhanced SOD activity with no alterations in the MDA content (Ding et al., 2018). The CYP enzymes, EROD (cyp1a) and BFCOD (cyp3a), showed inconsistent effects. Mechanistically, PSNAP perturbed protein homeostasis in the endoplasmic reticulum by inhibiting the expression of chaperon proteins and genes involved in endoplasmic reticulum-related degradation (Wang W. et al., 2023). The dysfunction of lipid metabolism in the liver was due to the activation of PERK-eIF2α and Nrf2/Keap1 pathways by PSNAP. Moreover, induction of oxidative stress (inhibition of SOD activity and enhanced level of MDA) is also involved in hepatic lipid accumulation (Wang W. et al., 2023). However, in the brain, the AChE enzymatic activity was significantly reduced by PSNAP exposure (Ding et al., 2018).

#### 3.5.6 Zebrafish

The search terms nanoplastics, PS, and zebrafish identified 69 articles belonging to embryo larval development (45 articles) and adults (26 articles). Five articles (4 on embryos and 1 on adults) were excluded from the review because the diameter of the studied PSNAP was >100 nm (Table 3). In these studies, the structure of PS (pristine/acidic/alkaline/aged/non-aged), sizes (15 nm–234 µm or 15–234,000 nm), concentrations/doses (0.04 ng–400 mg/L), modes of exposure (waterborne, injection, trophic transfer, and dietary) exposure conditions (temperature, pH, and depuration), and duration of exposure (4 h–120 dph; with or without depuration) were highly variable (Tables 2, 5). The accumulation and the effects of PSNAPs in embryos (chorion, yolk sac, mouth, trunk, eye, tail, caudal fin, muscle, somite, gill, GI tract, gall bladder, liver, intestine, pancreas, pericardium, heart, brain, nerve tubes, neuromast, and swim bladder) and in adults (gills, blood, GI tract, intestine, liver, gall bladder, pancreas, testis, ovary, brain, muscle) were dependent on the developmental stages (embryos) and the age (larvae, juveniles, and adults) of the fish (Tables 2, 5). Moreover, the studies indicated that PSNAP accumulated in different tissues of zebrafish larvae and adults have altered transcriptomes affecting the physiology and behavior of the fish (Pedersen et al., 2020).

##### 3.5.6.1 Embryo–larval development

Zebrafish embryos at different stages of development and transgenic zebrafish embryos were exposed to PSNAPs, and their effects on development (mortality, hatching rates, and morphology), cardiovasculature (heart rates, circulation, vessel formation, and endothelial cells), neurobehavior (spontaneous contraction in the early period of development, neurotransmitters, brain, eye, and movements), inflammation, oxidative stress, apoptosis, and gene expression were evaluated (Tables 6, 7). Although the 96-h LC_50_ of PSNAP (100 nm) on the 24-hpf zebrafish embryos was 431.1 mg/L (Feng et al., 2022), depending on the exposure routes and the concentration and duration of PSNAP exposure, inconsistent effects on survivability, malformation rates (pericardial edema, yolk sac edema, short tail, malformed head, jaw abnormalities, spontaneous movements of the embryos, sprouting of the transverse blood vessels, inhibition of myocardial diastolic functions, curved spine, scoliosis, and uninflated swim bladder), and hatching rates were observed; however, heart beats (bradycardia) and larval body length tended to reduce (Table 6). Moreover, concentration-dependent decline in ion contents (Na+, K+, and Ca ^2+^) and acid/ammonia excretion by skin cells of the embryos was observed after PSNAP exposure (Kantha et al., 2022). The number of active mitochondria in the ionocytes of the skin cells was also decreased by PSNAP accumulation in embryos in a concentration-dependent manner. Vascular malformations, including the ectopic sprouting of intersegmental vessels (ISVs), malformations of superficial ocular vessels (SOVs), and overgrowth of common cardinal veins (CCVs), as well as disorganized vasculature of the sub-intestinal venous plexus (SIVPs), were also observed in zebrafish embryos after PSNAP exposure (Dai et al., 2023). The gene expression analysis of the VEGFA/VEGFR pathways including *vegfa, nrp1, klf6a, flt1, fih1, flk1, cldn5a*, and *rspa3* were altered in a time- and concentration-dependent manner (Dai et al., 2023). These studies indicated that PSNAP interferes with the VEGFA/VEGFR pathways during embryogenesis and induced malformed vasculature in zebrafish.

The metabolic levels of the liver were significantly increased in larvae owing to PSNAP exposure (Chen J. et al., 2023). Particles with smaller sizes and longer duration of exposure (PSNAP; 50 and 100 nm diameter, for 24–120 hpf.) induced higher aggregations of neutrophils and apoptosis of macrophages in the abdominal region of the larvae (Cheng et al., 2022). The glycogen concentrations showed a concentration-dependent increase and isocitrate dehydrogenase concentrations inconsistently decreased during larval development when exposed to PSNAPs (Manuel et al., 2022). Cortisol concentration in the whole larvae (72–120 hpf exposure) was increased significantly by PSNAP exposure in a concentration-dependent manner (Brun et al., 2019). The glucocorticoid receptor mutant zebrafish larvae (*gr−/−*) have high cortisol levels, and no significant difference was observed in these larvae (*gr−/−*) exposed to PSNAPs (Brun et al., 2019). The expression of fabp10a (liver-specific fatty acid binding protein) was enhanced in the larval liver by PSNAP exposure (Cheng et al., 2022) and upregulation of the expression of *tg, trβ,* and *esr2* genes and enhanced expression of *tshβ*, thyroglobulin (*tg*), *nis, dio2,* and *trβ* and no effect on *cyp1a1* expression by PSNAP were also observed (Wang et al., 2022).

Decrease in the frequency of the spontaneous contraction of the embryos during development (Santos et al., 2024) indicated that PSNAP modulated nervous system development in zebrafish embryos. Administration by microinjection also showed bioaccumulation of PSNAP in the brain, which induced DNA damage and resulted in excessive ROS and apoptosis (Sokmen et al., 2020). PSNAP exposure decreased the number of larval neurons, axonal abnormalities in motor neurons, and induced neuronal apoptosis (Zhou W. et al., 2023). Compared with controls, there was a decrease in the GAD1 activity and GABA and 5-HT contents of larvae and no effect on the activities of AChE, tyrosine hydroxylase (THY), TPH and acetylcholine (ACh), and dopamine (DA) contents in larvae exposed to PSNAP (Zhou W. et al., 2023). Cholinesterase activity remained unaltered in larvae exposed to PSNAP. However, the activity of AChE significantly decreased in lower concentrations (0.01 and 0.1 mg/L) of PSNAP and increased in the higher concentration (1 mg/L; 22 nm) group (Manuel et al., 2022). The AChE activity was significantly enhanced after 144 hpf, while during recovery (maintained in PSNAP-free media), there was no significant difference between control and the exposure groups (Liu Y. et al., 2022). Compared with the controls, PSNAP (50 nm) with concentrations 1, 5, and 10 mg/L for 144 hpf (6 days) enhanced AChE activity and dopamine content of the larvae (Wang Y. et al., 2023). Moreover, exposure to PSNAP (80 nm) increased neural and optical-specific mRNAs (Chen et al., 2024). Transcriptomic analysis indicated that neurodegeneration and motor dysfunction were induced during larval development when exposed to PSNAPs. Expressions of *mbp* (responsible for myelination of axons) and *syn2α* (a neuronal phosphoprotein which induced synaptogenesis) were downregulated only in injected groups, and that of *gfap* (an intermediate filament protein, expressed in astrocytes) was downregulated only in waterborne exposure groups (Zhang et al., 2020). In transgenic zebrafish larvae [*Tg (atoh1a: dTomato*)], PSNAP (50 nm; 1, 5, and 10 mg/L for 144 hpf) inhibited the expression of *atoha1* mRNA in the cerebellum, thereby indicating damage to the central nervous system (Wang Y. et al., 2023). Single-cell RNA sequencing indicated PSNAP (12 h with 100 nm size PSNAP, 10 μg/L) regulated the expressions of *olig2, foxg1a*, *fzd8b, sis3a, rx1, lhx2b*, *nkx2.1a*, and *sfrp5* to alter nervous system development, retinal development, and stem cell differentiation (Liu et al., 2021). Upregulation of *gfap* and *α1-tubulin* mRNAs (related to nervous system) by PSNAP was also observed (Chen et al., 2017a).

PSNAP induced morphological changes in the eyes (decreased eye area with reduced interocular distance) and head (increased head area and reduction in head width and depth) (Santos et al., 2024). Expressions of visual system cone genes (*opn1sw2, opn1lw2* and *opn1mw1*) were downregulated by injection of PSNAP to the embryos; however waterborne exposure downregulated the expressions of *opn1w2* and *opn1mw1* only (Zhang et al., 2020). The gene expression analysis indicated PSNAP dominated the regulation of retinal system development genes (*pax1, pax2*, *six3*, *lax9,* and *six6*). However, increased cell density and disintegration of the retinal pigment epithelium occurred (Wang et al., 2022; Wang et al., 2023 L.). Genes related to visual system (rhodopsin, *zfrho*; blue opsin, *zfblue*) did not undergo significant alterations with PSNAP exposure (Chen et al., 2017a).

Metabolomic analysis revealed that the metabolic pathways of catabolic processes, amino acids, and purines were highly promoted by PSNAP exposure (Supplementary Table S1). Moreover, PSNAP induced the upregulation of several stress and immune-responsive genes (*il6* and *il1b*), cytochrome P450s (*cyp1a* and *cyp51*), and initiation of ROS removal protein-encoding genes (*sod* and *cat*). Moreover, PSNAP was also accumulated in macrophages during early development of zebrafish (Martin et al., 2023). ROS generation was induced by PSNAPs during embryo–larval development (Cheng et al., 2022). The activities of GST, GPx, and CAT decreased, although inconsistent (Santos et al., 2022). Moreover, the LPO levels showed inconsistent effects (Manuel et al., 2022). No effect was observed on CAT and GPx activity on larvae (96 hpf) with PSNAP exposure; however, GSH content decreased significantly (Chen et al., 2017a). The integrated biomarker response/index based on the seven oxidative stress-related biomarkers (SOD, CAT, GPx, GSH, GR, MDA, and ROS) showed an increase after PSNAP exposure (Bhagat et al., 2022; Chen J. et al., 2023). Enhanced ROS content induced apoptosis and ferroptosis (cell death due to iron accumulation). Significantly increase in NO content and decrease in the activities of soluble guanylate cyclase (sGC) and protein kinase G (PKG) enzymes were observed. Gene expression analysis indicated that PSNAP exposure significantly upregulated gene expressions related to antioxidant enzymes (SOD, GPx, and GST) and downregulated the expression of aromatase (*cyp19a1a* and *cyp19a1b*) and DNA methyl transferases (*dnmt3bb1*) (Liu et al., 2021). The expression of GPX4, the key protein for ferroptosis, and of the genes *Slc7a11, Acs14a, Keap1b*, and *Ncoa4* were higher in larvae exposed to PSNAP (Chen J. et al., 2023). The mRNA expressions of *cat, gpx1a, sod1*, and *sod2* were downregulated in embryos exposed to PSNAP, however, the expression of *casp3a* (apoptotic marker) mRNA was upregulated and that of *bcl2* mRNA (non-apoptotic marker) was downregulated in embryos exposed to PSNAP (Kantha et al., 2022). The activity of the caspase-3 and the expressions of *bik, bad, bax, bim, bid*, and *bok* were significantly increased by PSNAP exposure (Chen J. et al., 2023). Moreover, the expressions of several base excision pathway genes (*lig1, lig3, polb, parp1, pold, fen1, nthl1, apex, xrcc1, and ogg1*) were altered by PSNAP exposure (Feng et al., 2022).

The locomotor activity of the PSNAP-exposed larvae showed increased activity in the dark phase (Brun et al., 2019); however, the swimming behavior of the larvae exposed to PSNAPs (50 nm) did not show any significant change (Pedersen et al., 2020) but reduced counterclockwise and anticlockwise rotations (Zhang et al., 2020). Other behaviors (meander, angular velocity, and moving distance) remained unaltered (Zhang et al., 2020). In contrast, swimming behavior significantly decreased in the larvae (120 hpf) when the embryos were exposed to PSNAP (Barreto et al., 2023), or the effects observed in swimming behavior were found to be very insignificant (Parenti et al., 2019; Manuel et al., 2022; Tamayo-Belda et al., 2023). PSNAP exposure increased (50 nm; 1, 5, and 10 mg/L for 144 hpf) the swimming distance significantly by decreasing the swimming speed (Santos et al., 2024). However, PSNAP exposure suppressed the locomotor activity (total distance traveled) during the dark phase (Chen et al., 2017a). PSNAP exposure elicited complex effects on locomotor behavior with increased long distance and decreased short distance movements (Supplementary Table S1). When fish were allowed to recover (72 h), the locomotor behavior (swimming speed), compared with that in controls, significantly reduced during 144 hpf of development (Liu Y. et al., 2022). Behavioral analysis indicated that PSNAP exposure induced hyperactivity compared to control larvae (Santos et al., 2022; Gao X. et al., 2023). All these data suggested that PSNAPs have the potential to induce movement disorders in zebrafish.

Positively charged PSNAPs (PS-NH_2_) induced stronger developmental toxicity (decreased spontaneous movements of the embryos, heart beats, hatching rates, and larval length) and cellular apoptosis in the brain and greater impairment of neurobehavioral disorders (locomotor activity and behavior) than negatively charged PSNAPs (PS-COOH) (Teng et al., 2022a). A study compared the effects of pristine PS (80 nm, 0.5 and 5 mg/L), aged UV-PS (0.5 and 5 mg/L), and non-aged O3-PS (0.5 and 5 mg/L) on zebrafish embryos exposed for 8-120 hpf, indicated that these PSNAPs did not induce developmental toxicity (hatching, malformation, and mortality) (Chen J. et al., 2023). Cellular apoptosis was induced in 24 hpf embryos and 120 hpf larvae in all experimental groups (apoptosis mostly seen in embryonic tail and larval head region), except those exposed to O3-PS (Chen J. et al., 2023). Moreover, PS-NH_2_ interacted with neurotransmitter receptor N-methyl-D-aspartate receptor 2b (NMDA2B), whereas PS-COOH impacted on the G-protein coupled receptor (GPR1). The differences in the binding ability and affinity between neurotransmitter receptors (NMDA2B, and GPR1) as a function of positive or negative charge revealed the mechanism of different toxicity (Teng et al., 2022a).

The influence of temperature on the toxic effects of PSNAP on zebrafish embryos were studied after exposing the 4 hpf embryos to PSNAP (0.1, 0.5, and 1.0 mg/L) and then maintained at three different temperatures (24°C, 27°C, and 30°C) (Supplementary Table S1). The evaluation was made from 24 to 72 hpf (Duan et al., 2023). The elevated temperature promoted the accumulation of PSNAP during zebrafish development and resulted in an increase in the mortality of zebrafish larvae (Duan et al., 2023).

##### 3.5.6.2 Juveniles and adult zebrafish

Juveniles and adults of zebrafish were exposed to PSNAPs, and the effects on mortality, morphology, cardiovasculature (heart rates, circulation, vessel formation, and endothelial cells), neurobehavior (swimming activity, aggressiveness, predator avoidance, and shoal formation), inflammation, oxidative stress and apoptosis, gut microbiota, and gene expressions (Tables 6, 7) were evaluated. Depending on the exposure routes and the concentration and duration of PSNAP exposure, inconsistent effects on survivability and malformation rates were observed; however, heart beats (bradycardia) and body length tended to reduce (Table 6).

In zebrafish larvae (72 hpf), PSNAPs (20 mg/L) were accumulated in the intestine, exocrine pancreas, and gall bladder (Table 5; Supplementary Table S1), while the swim bladder failed to inflate (Brun et al., 2019). No effect was observed on growth, although the length of the larvae tended to reduce after PSNAP exposure. Cortisol concentration in the whole larvae (72–120 hpf exposure) was increased significantly by PSNAP exposure in a concentration-dependent manner (Supplementary Table S1).

Zebrafish juveniles were exposed to 1,000 μg/L PSNAP (50 nm diameter) through diet (Tables 2, 5, 6; Supplementary Table S1). The feeding with regular diet was done for 3 weeks, while for PSNAP exposure, it was only for 1 week. It was observed that PSNAPs perturb lipid metabolism and gut microbiota stability in zebrafish (Du et al., 2024) despite no effects on the body weight. The CAT activity increased, and MDA content decreased, while SOD activities remained unaltered in the liver. The mRNA expression of *cpt1ab* was upregulated, that of *fasn* was downregulated, and that of *hmgcra* remained unaltered after PSNAP exposure (Du et al., 2024).

Juvenile/adult zebrafish were exposed to PSNAPs (44 nm) for 30 days (1, 10, and 100 μg/L), and growth and the brain–intestine–microbe axis were evaluated. It was observed that the growth of the fish (body length) was significantly inhibited in a concentration-dependent manner (Table 2; Supplementary Table S1). Moreover, metabolomic analysis revealed alterations in 42 metabolites involved in neurotransmission (Teng et al., 2022b). Moreover, changes in fourteen metabolites correlated to changes in three microbial groups, including *Proteobacteria, Firmicutes*, and *Bacteroidetes*, in fish exposed to PSNAPs. These findings suggest that PSNAPs cause intestinal inflammation, growth inhibition, and restricted development of zebrafish, which are strongly linked to the disrupted regulation within the brain–intestine–microbiota axis (Teng et al., 2022b).

In zebrafish adults, PSNAP exposure (either fluorescently labeled or regular) did not significantly affect the survivability, body length, BMI, or the observable health of the fish. The bioaccumulation of the PSNAP was dependent on the concentrations, duration of exposure, and tissue types (intestine, liver, gill, muscle, brain, and gonads) (Chen et al., 2017b; Sarasamma et al., 2020; He et al., 2021; Habumugisha et al., 2023; Lin et al., 2023; Yang et al., 2023; Ye et al., 2024; Zhang et al., 2024c). During depuration, PSNAP was eliminated from the gut within 2–3 days in a concentration-dependent manner (Yang et al., 2023).

In the intestine, the damage of the epithelium including a cilia defect and enhanced mucus secretion induced by PSNAP exposure depended on the size of the plastic; as the size decreased, the damage of the intestinal epithelium increased (Yu J. et al., 2022; Yu et al., 2022 Z.). The histophysiology indicated vacuolization of the intestinal goblet cells and mitochondria (Teng et al., 2023), and the intestinal villi were swollen and disorganized in the fish exposed to PSNAP, even though the height of the villi significantly decreased. Moreover, the ratio of the villus height/crypt depth or the ratio of the villus height/villus width was also significantly decreased by PSNAP exposure when compared with controls (Teng et al., 2023; Zhang et al., 2024c). The level of ROS in the intestine markedly increased and GSH content significantly decreased; however, SOD activity and MDA content remained unaltered (Zhang et al., 2024c). In contrast to these studies, Teng et al. (2023) observed a significant concentration-dependent increase of SOD activity and an inconsistent increase in MDA content in the intestine of zebrafish adults exposed to PSNAP (80 nm, 15–150 μg/L, 21 days). The mitochondrial DNA content was significantly reduced and that of TNF-α and immunoglobulin IgM was increased by PSNAP exposure in the intestine in a concentration-dependent manner. Moreover, in the intestine, 5-HT level tended to decrease in fish exposed to PSNAP (Zhang et al., 2024c). Compared with controls, the activity of MAO (the catalytic enzyme of 5-HT) and the mRNA level of *mao* in the intestine tended to decrease in fish exposed to PSNAP. The mRNAs (*tph1a, tph1b*, and *tph2*) of tryptophan hydroxylase (TPH), the rate-limiting enzyme for 5-HT synthesis, showed a tendency to downregulate in fish exposed to PSNAP (Zhang et al., 2024c). Concentration-dependent dysregulation of the gene expression of several genes in the intestine was observed in adult zebrafish exposed to PSNAP (downregulation of *tnfα, il1β, il10*, and *chemokine 8a* in fish exposed to 1 and 10 μg/L; upregulation of *tnf, il1b, il6, il10, cxcl8a*, inflammatory *caspase B*, and tight junction protein 2a in fish exposed to 100 μg/L), while the expression of *ahr* was downregulated by all concentrations of PSNAP used in the experiments (Teng et al., 2022b). PSNAP exposure decreased the expression of *IL-6* and increased the expression of nuclear factor kappa-B (*nf-κb*) in the intestine. The expression of *IL-1β* in the intestine was upregulated by PSNAP exposure (15 μg/L) while downregulated by a higher concentration (150 μg/L). The expressions of tight junction proteins 2a (*tjp2a*) and *tjp2b, cyp1a1,* and *cyp1b1* increased significantly in the intestine of fish when exposed to a lower concentration of PSNAP (15 μg/L) (Teng et al., 2023).

There are seven types of cells identified in zebrafish intestine: enterocytes, macrophages, neutrophils, B cells, T cells, enteroendocrine cells, and goblet cells (Yu J. et al., 2022), and the effects of PSNAP were found to be cell-specific. In macrophages, immune system-related DEGs (*ctsba, nfkbiab*, and *pycard*) were significantly altered by PSNAP exposure, and the genes related to MAPK signaling pathways (*hsp70.1, hsp70.2*, and *hsp70l*) remained unaltered. In enterocytes, genes related to GSH metabolism (*gsta2, gsto1, gsto2, gpx1a,* and *mgst1.2*) and cytochrome P450 remained unaltered. In B and T cells, upregulation of *hsp70.1, hsp70.*2, and *hsp70.3* occurred in fish exposed to PSNAP. Gene ontology (GO) analysis found several other DEGs such as *gadd45ba, jun, ccl35.2,* and *ccl35.2* remained altered in macrophages after PSNAP exposure. In enterocytes, GO analysis showed alterations in the expression of *apoa4a, apoa1a*, and *apoea* in fish exposed to PSNAP. Moreover, PSNAP (1 mg/L) induced dysbiosis in gut microbiota and significantly increased the abundance of Proteobacteria and decreased that of Fusobacteria, Firmicutes, and Verrucomicrobiota at the phylum level; at the genus level, *Aeromonas* abundance was increased by PSNAP exposure ((Xie et al., 2021; Yu Z. et al., 2022; Yang et al., 2023; Zhang et al., 2024c). Therefore, the diversity and abundance of the gut virome were also disrupted by PSNAP exposure (Teng et al., 2023).

In adult fish, PSNAP exposure increased HSI and also vacuoles and lipid droplets in the liver cell matrices (Li Y. et al., 2023). Moreover, the triglycerides and total cholesterol content also increased in the liver (Tables 5; Supplementary Table S1). A significant increase in MDA content and decrease in CAT activities and GSH levels suggests significant oxidative damage induced by PSNAP in zebrafish liver (Deng et al., 2023). Like the intestine, zebrafish liver also consists of nine different types of cells, of which 85% cells were hepatocytes belonged to male (52.39%) and female (33.63%) fish (Deng et al., 2023). The single-cell transcriptomic analysis (scRNA-seq) observed the heterogeneous response patterns of hepatocytes belonging to male and female fish (Supplementary Table S1; Deng et al., 2023). The peroxisome proliferator receptor activator (PPAR) signaling pathway was upregulated in hepatocytes of both male and female zebrafish (Deng et al., 2023). Lipid-metabolism-related functions were altered more notably in male-derived hepatocytes, while female-derived hepatocytes were more sensitive to estrogen stimulus. In macrophages, oxidation–reduction process and immune responses were significantly altered, while in lymphocytes, oxidation–reduction process, ATP synthesis, and DNA binding were mostly altered (Deng et al., 2023). Moreover, a nonlinear increase in the gene hydroxy-3-methylglutaryl-coenzyme A (*hmgcra*), sterol regulatory element-binding protein (*srebp1*), diacylglycerol aceyltransferase 1b (*dgat1b*), acetyl coenzyme A carboxylase (*acc*), and carbohydrate response element-binding protein (*cvhrebp*) by PSNAP exposure in the liver was observed; however, the expression of carnitine palmitoyl transferase 1 (*cpt1)* was decreased significantly by PSNAP exposure (Sarasamma et al., 2020). In the liver, biochemical biomarkers (*tnfα*, cortisol, vitellogenin, *cyp1a1*, *cyp11a1*, and *cyp19a1*) were altered after 30 days of exposure to PSNAPs; however, no alteration was observed in MDA content and EROD activities (Sarasamma et al., 2020). In addition, PSNAP exposure did not show any induction of *esr2b, vtg1,* or *vtg2* mRNAs in the liver of both males and female fish (Ye et al., 2024). In contrast to the studies mentioned above, the studies carried out by Ling et al. (2022) indicated that the histology of the liver remained unaltered in the fish exposed to PSNAP (70 nm, 100 μg/L for 3 months) (Ling et al., 2022). HSI either remained unchanged (He et al., 2021) or a significant decrease was observed in both male and female fish (70 nm, 2 mg/L, 3 weeks) with exposure to PSNAP (Lin et al., 2023). The biochemical analysis of the oxidative stress-related mechanisms also showed that PSNAP was unable to induce any significant effects on the ROS, GSH, and MDA contents and the CAT activity (Ling et al., 2022). Consequently, gene expression analysis related to antioxidant mechanisms (*p38a, p38b, ERK2, ERK3, Nrf2, H O -1, cat1, sod1, gax, JINK1*, and *gstr1*), remained unaffected after PSNAP exposure (Ling et al., 2022).

In the muscle, PSNAP exposure enhanced ROS content and reduced GR activity in female fish, while ATP content was decreased, and no alteration was observed in creatine kinase and *hif1α* contents (Pitt et al., 2018b; Sarasamma et al., 2020).

PSNAP, when accumulated in the brain of adult zebrafish, slightly increased (not significant) the craniosomatic index (CSI), resulted in damage to the brain histology, and reduced the number of neurons in a concentration-dependent manner (Aliakbarzadeh et al., 2023; Teng et al., 2023). Moreover, the basement membrane of the blood–brain barrier (BBB) was damaged, and a small amount of microthrombosis consisting of aggregated and dissolved red blood cells was observed; also, the mitochondria with a damaged membrane and loss of cristae were observed. Consequently, mitochondrial DNA copy number was significantly reduced, and the genes related to mitochondrial synthesis (*pgc1-a* and *pgc1-b*) in the zebrafish brain did not show any significant effects. However, the mitochondrial fusion-related gene (*mfn1a, mf1b*, and *opa1*) expressions were downregulated and those of mitochondrial division-related genes (*drp1, mff, fis 1, mid49,* and *mid51*) showed a tendency to upregulate (Zhang et al., 2023). The expression of genes related to mitophagy (*ulk1a*, and *parl*) were also upregulated by PSNAP exposure. The enzymatic activities of CAT, SOD, AChE, GR (females), glutamine synthase, and GSH contents in the brain were reduced by PSNAP exposure (Pitt et al., 2018b); moreover, GPx (only females) and glutamate dehydrogenase activity in the brain was increased in fish exposed to PSNAP, and upregulation of myelin/basic protein gene expressions occurred in the central nervous system of adult zebrafish (Chen et al., 2017b; Pitt et al., 2018b). Several neurotransmitter biomarkers (AChE, dopamine, melatonin, GABA, serotonin, vasopressin, kisspeptin, and oxytocin) were significantly altered in a concentration-dependent manner in fish exposed to PSNAPs, even though the acetylcholine, prolactin, and vasotocin levels remained unaltered (Chen et al., 2017b; Sarasamma et al., 2020).

The 5-HT level in the brain was significantly reduced in fish exposed to PSNAP, while the serum 5-HT levels remained unaltered. Among the 5-HT receptor mRNAs, expressions of *htr1aa, htr1ab,* and *htr2c* were significantly upregulated, while the expressions of *htr1b* and *htr4* showed downregulation in the brain of fish. In addition to 5-HT, PSNAP exposure decreased GABA, dopamine, and oxytocin levels and enhanced cortisol content in the brain (Teng et al., 2023). The activity of MAO tended to decrease, while AChE activity remained unaltered (Zhang et al., 2023). The neurotransmitter catabolic gene *mao* was significantly downregulated, while the expression of *ache* tended to increase in the brain of fish exposed to PSNAP (Zhang et al., 2023). Compared with controls, the γ-H2AX levels (marker for DNA damage), 8-hydroxydeoxyguanosine (8-OHdG), and MDA contents were significantly higher in the brain of male and female fish exposed to PSNAP (Zhang et al., 2023). Moreover, the ATP and cyclin-dependent kinase levels were significantly lower and p53 levels were significantly higher in the brains of male and female zebrafish exposed to PSNAP, and the β-galactosidase and lipofuscin levels (aging markers) are significantly higher in the brain of zebrafish (both males and females) exposed to PSNAP, with higher levels of H_2_O_2_ and O_2_^−^ in the brain (Zhou W. et al., 2023).

The impacts of PSNAP exposure (50 nm; 1.0 mg/L, 21 days) on the adult zebrafish were also focused on reproductive endpoints (Tables 6,7). It was observed that PSNAP was unable to alter the GSI in both males and female fish, cause histological alterations in the ovary and testis, egg production (fecundity) and hatching of the embryos, and the expressions of *sgk1* (glucocorticoid-regulated kinase 1) and *stc* mRNAs in the ovary; moreover, the E2 level of the ovary and serum, T, GnRH, FSH, and LH contents in the ovary also remained unaltered after PSNAP exposure (Ye et al., 2024). In male fish, E2 levels in the serum and testis and the GnRH, FSH, and LH levels in the testis remained unaltered (Ye et al., 2024). The expressions of *cyp17a2* and *hsdβ1* mRNAs in the ovary and testis remained unaffected after PSNAP exposure.

Adult male and female zebrafish exposed to 2 mg/L PSNAP (46 nm) for 21 days (Table 2; Supplementary Table S1) showed no significant effects on HSI, GSI, histological alterations in the testis and ovary, spermatogenesis and oogenesis, VTG content, and E2 and T levels in male and female fish (He et al., 2021). However, the amount of mature sperm in the testis and the fecundity (total eggs produced during the experimental period) of the fish decreased in fish exposed to PSNAP (He et al., 2021). The spawning events, fertilization, and hatching rates of the eggs remained unaltered in fish exposed to PSNAP (He et al., 2021).

The studies conducted by Lin et al. (2023) indicated that PSNAP (70 nm, 2 mg/L, 21 days) exposure can decrease HSI and GSI in both male and female fish. Moreover, in male fish, the seminiferous tubules were deformed, and lacunae appeared in the testis; the spermatogonium and spermatocytes were increased (Lin et al., 2023). In female fish, PSNAP exposures showed more preovulatory oocytes and smaller mature oocytes than controls. The levels of E2 and T in PSNAP-exposed fish decreased in both male and female zebrafish (Lin et al., 2023). However, no effect of PSNAP on the E2/T ratio of male and female fish was observed. The VTG content of male fish remained unaltered, while in female fish, VTG content was induced by PSNAP exposure in a concentration-dependent manner. Moreover, no significant effects on the T3 and T4 levels of both male and female fish were observed after PSNAP exposure (Lin et al., 2023). Compared to controls, PSNAP exposure reduced fecundity, spawning events, fertilization, and hatchability of the embryos. In addition, PSNAP exposure induced abnormal development (teratogenic effects) of the larvae observed at 96 hpf (spinal curvature, pericardial cyst, and growth retardation) (Lin et al., 2023).

Behavioral alterations in locomotor activities (aggressiveness, shoal formation, and predator avoidance behavior) in adult zebrafish were affected by PSNAP exposure in a concentration-dependent manner, while the circadian rhythm of locomotor activity was dysregulated (Sarasamma et al., 2020). PSNAP exposure induced anxiety-like behavior; however, the average velocity and acceleration were unaffected by the treatment (Teng et al., 2023). Adult male and female zebrafish were exposed to 1 mg/L PSNAP (50 ± 3 nm) for 28 days, and the learning and memory (the primary cognitive functions of the brain) were assessed with classic T-maze exploration tasks. It was observed that PSNAP-exposed zebrafish (both males and female) took significantly longer time for their first entry and spent significantly less time in the reward zone in the T-maze task, indicating deficit in the learning and memory (Zhou W. et al., 2023). Adult male and female zebrafish were exposed to PSNAP (100 nm sizes) at a concentration of 1 mg/L for 30 days (Table 2; Supplementary Table S1). The anxiety-like behavior (evaluated by the open field test) showed those exposed to PSNAP alone spent more time in the lower layer than the upper layer, while controls spent uniform time in both upper and lower layers. Furthermore, in the T-maze test, control and PSNAP groups swam quickly in the feeding zone (F zone) and stayed there for long time (Zhang et al., 2024c), indicating effective learning and memory ability of the fish.

Zebrafish adults (3 months old, AB strain) were exposed to 25 mg/L PSNAP (134 ± 2.9 nm) at 28°C, 29°C, and 30°C for 96 h (Table 2; Supplementary Table S1). It was observed that PSNAP exposure with increased temperature induced DNA damage, degeneration, necrosis, and hyperemia in the liver, while in gills, adhesion of lamellae, desquamation, and inflammation in the lamellar epithelium and in muscle alteration in oxidative stress were observed (Senol et al., 2023). Moreover, the locomotor activity (total distance traveled, average speed, and average angular velocity) was decreased in PSNAP-exposed fish, and these effects were modulated by temperature (Sulukan et al., 2022b). The PSNAP was accumulated in the brain and induced degenerative necrosis changes in the medulla oblongata, medial longitudinal fascicle, lateral valvula nucleus, and thalamus, and the effect was increased with the increased in temperature (Sulukan et al., 2022b). Moreover, two proteins, Gfap (indicator of brain injuries) and 8-OHdG (indicator of oxidative DNA damage), were found to be increased in the damaged region of the brain, which is also temperature-sensitive (Sulukan et al., 2022b). Moreover, the temperature and PSNAP exposure caused a synergistic effect on the brain metabolomic alteration (Sulukan et al., 2022b)

##### 3.5.6.3 Intergenerational effects

The intergenerational effects were evaluated in F1 embryos or adults exposing zebrafish embryos (1 article) or adults (3 articles) to PSNAPs in the F0/P1 generation for a reasonable period of time, and the effects on offspring (F1) without exposing them to the plastics were evaluated. In a study on zebrafish, fertilized eggs (4 hpf) were injected with PSNAPs (20 nm, ∼270 mg/L; 3 nL injected volume/egg) and grown in plastic-free media for 6 months (P1) and were allowed to breed, and the offspring (F1) were evaluated for morphological, molecular, and metabolomic disorders (Table 5; Supplementary Table S1). It was observed that compared with controls, parental PSNAP exposure (P1) induced significant malformations, decreased survival rates, increased heart rates, as well as decreased eye size and locomotor activity in the F1 offspring (Sulukan et al., 2022a). In addition, cell death and ROS were increased significantly; however, lipid accumulation was decreased in the F1 generation (Sulukan et al., 2022a).

AB strain zebrafish adults (90 dpf) were exposed to PSNAP (54.5 ± 2.8 nm; 10 mg/L, 90 days), waterborne and F1 larvae (without exposure to PSNAP) were evaluated for disruptions induced in the HPT axis (Table 2; Supplementary Table S1). Parental exposure (F0) to PSNAP reduced survival rates, hatching rates, and body length (7 dpf) and significantly enhanced the malformation rates during the embryo–larval development of F1 larvae (Zhao et al., 2021). Compared with controls, total T3 and T4 levels in F1 larvae remained unaltered; in F1 eggs, T4 level reduced significantly, while T3 level remained unaltered (Zhao et al., 2021). However, in F1 larvae, no significant changes in T3 and T4 contents were observed. In another experiment, adult zebrafish were exposed to 100 μg/L PSNAP (70 nm) for 21 days (P1), and the F1 larvae (120 hpf) were evaluated for intergenerational effects (Table 6; Supplementary Table S1). It was observed that due to parental exposure (F0), accumulation of PSNAP was detected in the testis and ovary of the F1 larvae (Zuo et al., 2021). PSNAP exposure to parents had no effect on the induction of developmental disorders and no alterations in the T4 and T3 levels. Gene expressions in the HPT axis and GH/IGF axis remained unaltered. In a study by Wu et al. (2021) in which parents (P1) were exposed to PSNAP (70 nm, 100 μg/L) for 45 days (Table 2; Supplementary Table S1), the F1 embryos/larvae were evaluated for intergenerational effects. It was observed that PSNAP was accumulated in the F1 embryos (Wu et al., 2021); however, compared with controls, no significant effect was observed on hatching rates (72 hpf), hatching enzymatic activities, and spontaneous tail movements (wagging). Moreover, no significant effect was observed on the AChE activity of the F1 embryos exposed to PSNAP, parentally; gene expression analysis related to hatching enzymes (*tox 16, foxp1, ctslb, xpb1, klf4, cap1, bmp4, cd63, He1.2, zhe1,* and *prl*), cholinergic system (*ache* and *chrnα7*), and muscle development (*Wnt, MyoD, Myf5, Myogenin, and MRF4*) indicated alterations in the F1 larvae exposed parentally to PSNAP (Wu et al., 2021). In another study, juvenile/adult zebrafish were exposed to PSNAPs (44 nm) for 60 days (1, 10, and 100 μg/L), and the intergenerational effects during embryo–larval development (F1) were evaluated (Teng et al., 2022b). Accumulation of PSNAPs in the GI tract after 60 days of exposure to the fish impaired the development of the F1 embryos, including reduced spontaneous movement, hatching rates, and larval length (Teng et al., 2022b). Moreover, accumulation of PSNAPs was observed in the intestine, liver, and pancreas of the F1 fish (Teng et al., 2022b).

Taken together, it was observed that PSNAP as a chemical is transferred to the next generation and is accumulated in the whole embryos, intestine, liver, pancreas, and gonads (testis and ovary) of the F1 offspring. Moreover, several of the toxic potentials observed in the P1 fish were also observed in F1 fish, which indicate that intergenerational effects of PSNAP were independent of the dose, duration, mode of exposure, and developmental stage of zebrafish.

### 3.6 Coexposure

NAPs with small particle sizes and high surface area/volume ratios easily absorb environmental pollutants and affect their bioavailability (Liu et al., 2021). Due to high adsorption activity, the toxic effects of NAPs could be modified by exposure to other toxic chemicals found in the environment. Moreover, NAPs can absorb contaminants and potentially decrease their uptake due to particle agglomeration or function as a vector to accumulate the hazardous chemicals inside the cell, which were unable to enter by themselves. Our literature search found several chemicals including hormones, pesticides, antibiotics, metals, organic chemicals, biological materials, and bacteria disposed/found in the environments used as additional contaminants along with NAPs during experiments (Tables 8, 9). In coexposure studies, the diameter of the PVC particles is 200 nm (Monikh et al., 2022). We therefore excluded this article from the review. Among thirteen fish species, only six species, grass carps (juveniles), silver carp (adults), tooth carp (adults), marine medaka (embryos, juveniles, and adults), Hainan medaka (adults), and zebrafish (embryo–larvae–juveniles–adults), were used in coexposure experiments (Tables 8, 9).

#### 3.6.1 Carps

Juveniles of grass carp were coexposed with tetracycline (TC), ZnO, and also infected with pathogenic bacteria (*Aeromonas hydrophilia*) during PSNAP exposure (Table 8). TC coexposure showed pathogenic lesions in the gills and intestine and enhanced the oxidative stress-related changes (total antioxidant capacity and the activities of CAT and SOD) in the liver and intestine (Liu S. et al., 2022). The expressions of *MMP2, MMP9,* and *IL-8* in the liver and intestine of the coexposed fish were also upregulated (Table 9; Supplementary Table S1; Liu S. et al., 2022). Coexposure with ZnO (750 μg/L) did not induce alterations in the locomotor activity, biochemical concentrations of the liver and brain (carbohydrates, proteins, and triglycerides in the liver and carbohydrate and protein contents in the brain), while it increased the oxidative stress-related activities and AChE activity in the brain (Estrela et al., 2021). Moreover, DNA damage in the erythrocytes was also observed. Injection of the pathogenic bacteria to grass carp, pre-exposed to PSNAP (80 nm diameter, 10–1,000 μg/L), showed enhancement in the enzymatic activities of CAT, SOD, and GST, and MPO and MDA contents were enhanced in the oxidative stress-related mechanisms in the grass carp gut after bacterial infection (Li Z. et al., 2024). Moreover, the microbial communities in the gut were also modified after injection of *A. hydrophilia* (Li Z. et al., 2024). In silver carp adults (*Hypopthalmichthys molitrics*), MCLR (1 μg/L) coexposure caused pathological damages in the gill, liver, and intestine of the fish (Zhang et al., 2024a) and aggravated the changes in the microbial community in the intestine and the metabolic patterns in the liver (Table 7). In tooth carp, coexposure with triclosan (TCS) did not significantly affect the uptake of PSNAPs in the organs of tooth carp and reduced the toxic effects induced by PSNAP in this fish (Saemi-Komsari et al., 2023).

#### 3.6.2 Medaka

Embryos, juveniles, and adults of marine medaka were used in coexposure studies. Embryos were coexposed with BPA, juveniles with SMX, and adults with SMZ (Table 8). BPA reduced the accumulation of PSNAP in the embryos and thus mitigated the toxic effects of PSNAP on embryo mortality, heart rates, and larval body length during embryo larval development (Yu et al., 2023). In juveniles, SMX coexposure was unable to modulate the toxic effects (mucus content in the intestine, goblet cell number, and gut microbial community) induced by PSNAP exposure alone (Li X. et al., 2023). Coexposure of SMZ in adults (through diet) modulated the gut microbial community (Wang F. et al., 2023) and the intergenerational effects of PSNAP on growth, gut microbial content, and the hepatic gene expressions (*cat, sod*, *gpx,* and *igf1*) in F1 generation (He et al., 2022). Hainan medaka adults were coexposed with F-53B, which can interact with the effects induced by PSNAPs and modulated the effects on the accumulation, histology, antioxidant activity, and gut microbiota induced in fish after PSNAP exposure (Gao X. et al., 2023).

#### 3.6.3 Zebrafish

In zebrafish, embryos along with PSNAP were coexposed with varieties of chemicals including acetaminophen (APAPM), Al_2_O_3,_ Au, avobenzone (AVO), B(a)P, BDE-47, CeO2, diphenhydramine (DPH), DDE, EE2, glucose, PAHs, penicillin, mucin (jelly fish), phenmedipham, simvastatin (SIM), and sodium nitroprusside (SNP), and the toxic effects of PSNAP with interaction of these compounds were evaluated (Tables 8, 9).

It was observed that APAPM, a non-opioid and antipyretic agent used for treating pain and fever, potentiated the toxic effects of PSNAP in inducing edema, spinal curvature, pigment deficiency, melanocyte abnormalities, and reducing larval body length, and in the swimming behavior of zebrafish (Gao X. et al., 2023). Moreover, the downregulation of genes related to osteogenesis (*runx2a, runx2b, sp7, bmp2b,* and *shh*) by PSNAP was also observed with APAMP coexposure (Gao X. et al., 2023). AVO is an organic molecule used in sunscreens (cosmetics), and exposure to PSNAP alone enhanced the accumulation of AVO in zebrafish embryos in a time-dependent manner and did not produce any lethal effects and morphological disorders (Table 8); however, the heart rates increased and the locomotor behavior (swimming speed) significantly reduced (Liu et al., 2021; Liu Y. et al., 2022). In addition, oxidative stress, which was enhanced by exposure with PSNAP and AVO alone, was reduced in coexposed embryos (Liu et al., 2021). The AChE activity significantly enhanced during coexposure, while during recovery (maintained in treatment-free medium), there was no significant difference with the controls (Liu Y. et al., 2022). Gene expression analysis indicates that exposure to AVO and PSNAP alone significantly upregulated gene expressions related to antioxidant enzymes (CAT, SOD, GPx, and GST by AVO and SOD, GPx, and GST by PSNAP) and downregulated the expressions of aromatase (*cyp19a1a* and cyp*19a1b*) and DNA methyl transferases (*dnmt1* and *dnmt3aa* by AVO and *dnmt3bb1* by PSNAP); however, the coexposure reduced the adverse effects induced by PSNAP and AVO alone during the expression of all these genes (Liu et al., 2021). Moreover, genes in stem cells (*foxg1*, *her5, her6, shha,* and *sox2*) were responsive to exposure of both AVO and PSNAP (Liu Y. et al., 2022). During the early life stages of zebrafish, AVO dominated the regulation of nervous system-related genes (*α1-tubulin*, *elav13, gap43, gfap, mbp, syn2a, lfing, her5, her6, her11, lfng, pax2a*, and *fgfr4*), while PSNAP alters gene expression related to nervous system development, retinal development, and stem cell differentiation (*pax1, pax2*, *six3*, *lax9, six6, olig2, foxg1a*, *fzd8b, sis3a, rx1, lhx2b*, *nkx2.1a*, and *sfrp5*) (Liu et al., 2021; Liu Y. et al., 2022).

Zebrafish embryos were coexposed with BDE-47 (2,2′,4′-tetrabromodiphenyl ether; 10 ng/L), a flame-retardant, and the effects on accumulation, morphological deformities (pericardial edema, yolk sac edema, tail curvature, jaw malformation, and fin and heart malformation), spontaneous movement during embryonic development, survival and hatching, growth, feeding, oxygen consumption, larval movement, histopathology of the eye, muscle, and cartilage, and gene expressions in the HPT-, HPI-, and HPG-axis,VTG, and other genes (*apoa1a, apoba, insa, insb, pck, pomca,* and *pomcb*) were evaluated. It was observed that PSNAPs alone were quickly aggregated on the surface of the embryonic chorions and accumulated in the brain, mouth, trunk, gills, heart, liver, and GI tract of the larvae (Chackal et al., 2022; Wang et al., 2022; Wang Q. et al., 2023) and served as a vector for accumulation of B(a)P in the embryos (Martinez-Alvarez et al., 2022). Moreover, coexposure with BDE-47 exacerbates the morphological deformities induced by PSNAP with regard to hemorrhage, small head and eyes, yolk edema, pericardial edema, spine curvature, swim bladder deficiency, and curved tail (Wang et al., 2022; Wang et al., 2023 L.). In addition, coexposure caused lower survival rates and shorter body lengths and accelerated spontaneous movements of the embryos. Histopathological observations revealed that coexposure caused damage to retinal structures, muscle fiber, liver morphology (color), and cartilage tissues. Gene expression analysis further indicated that exposure to PSNAP alone upregulated the expressions of *tshβ*, *tg*, *nis, dio2,* and *trβ* and had no effect on *cyp1a1* (Wang et al., 2022; Wang et al., 2023 L.); however, coexposure with BDE-47 upregulated the expressions of *cyp1a1* and *tg*, while downregulating the expressions of *tshβ, nis, ttr, doi2*, *trβ,* and *gpx1a* in larvae (Wang et al., 2022; Wang et al., 2023 L.), which indicates the negative interaction with the gene expression made by BDE-47 was abolished by PSNAP (Chackal et al., 2022).

Zebrafish embryos (6hpf) were exposed to PSNAP either alone or with a mixture of river sediment extracts that contain PAHs for 96 hpf (Tables 8; Supplementary Table S1). It was observed that in coexposure, the incidence of disorders induced by PAH alone was reduced (Trevisan et al., 2019). Moreover, PSNAP, either alone or in coexposure increased NADH production. PSNAP alone accumulated in the yolk sac and brain; however, accumulation of PAH was observed only in the yolk sac when exposed to PAH alone; during coexposure, PAH accumulation was observed in the brain (Trevisan et al., 2020). This study indicates that PSNAPs can absorb contaminants and potentially decrease their uptake due to particle agglomeration or function as a vector to accumulate the hazardous chemicals inside the cell, which were unable to enter by themselves. Zebrafish embryos coexposed with PHE (an aromatic hydrocarbon; PSNAP + PHE) and jellyfish mucin (PSNAP + PHE + mucin) (Table 8) showed that PSNAP and PHE alone induced pericardial edema, yolk sac edema, and decreased hatching rates (Geum and Yeo, 2022), and PSNAP was agglomerated on the surface of the chorion of the embryos in PSNAP + PHE groups, while in coexposure with mucin (jellyfish), a clean chorion was observed (Table 8).

PSNAP enhanced the accumulation of aluminum and cerium in zebrafish embryos by inhibiting the ATP-binding cassette (ABC) transporter inhibitor activity, while no effect was observed on embryo mortality or malformation rates (pericardial edema, yolk sac edema, curved tail, and spinal curvature). The hatching rate declined in embryos co-exposed with CeO_2_. Coexposure with chloroauric acid (Au) synergistically exacerbated the marginal effects induced by PSNAP on the survival, hatching rate, developmental abnormalities, and cell death of zebrafish embryos, which was dependent on the production of ROS and the proinflammatory responses synergized by the combined toxicity of PSNAP and metal ions (Lee et al., 2019; Bhagat et al., 2022). Enhanced ROS production and oxidative stress lead to the activation of genes (*gadd45a*, *p53*, *xrcc2*, *rad51*, and *trl3)* associated with DNA damage and repair. Al_2_O_3_ alone upregulated the expression of *gadd45a* and *xrcc2,* and coexposure with PSNAP enhanced the expression of *rad51* and *p53*; moreover, coexposure with CeO_2_ downregulated *tlr3* and *mt2* gene expressions (Bhagat et al., 2022). There was no change in metallothionine (*mt2*) expression by PSNAP alone, while both Al_2_O_3_ and CeO_2_ alone enhanced *mt2* expression; surprisingly, coexposure with PSNAP significantly decreased the expression of *mt2* compared to the expression induced by AL_2_O_3_ and CeO_2_ alone (Table 9). The expressions of *abcc2* and *P-gp* mRNAs were upregulated, and those of *abcc1, abcc4*, and *abcb4* mRNAs were downregulated (efflux transporter genes) by PSNAP exposure. Al_2_O_3_ alone, except *abbcc2,* downregulated the expression of the efflux transporter genes studied, while CeO_2_ alone downregulated the expressions of *abcc1, abcc4, abcb4*, and *p-gp*. Coexposure with Al_2_O_3_ (increased *abcc4*) and CeO_2_ (reduced *abcc1* and *p-gp*) modulated the expression patterns of efflux transporter genes regulated by PSNAP (Table 9). The synergistic effects of PS on toxicity appeared to relate to the mitochondrial damage. Taken together, the effects of PSNAPs were marginal but could be a trigger for exacerbating the toxicity induced by metal ions (Lee et al., 2019; Bhagat et al., 2022).

Coexposure with antihistamine diphenhydramine (DPH) for 96 h induced embryo mortality, malformations, and decreased heart beats and hatching rates; moreover, the activities of GST and AChE increased, while that of CAT remained unaltered (Barreto et al., 2023). The movement disorders were also induced in larvae with PSNAP and DPH coexposure (Barreto et al., 2023). Moreover, coexposure of zebrafish embryos with phenmedipham (PHN), an herbicide, did not induce any significant change in embryo mortality or deformities; however, at 96 hpf, the PSNAP increased CAT activity, while coexposure increased both CAT and GST enzymatic activities (Santos et al., 2022). Behavioral analysis indicates that during 120 hpf (larvae), PS alone or coexposed with PHN induced hyperactivity (Santos et al., 2022). Moreover, cholinesterase activity was found to be decreased only in coexposed larvae and not in larvae exposed to PSNAP or PHN alone. In coexposure with DDE, due to its large surface area, PSNAP served as a carrier of the pesticide and enhanced toxicity (morphological, cardiac, and respiratory) in zebrafish embryos (Varshney et al., 2023). DDE alone or in combination with PSNAP induced pericardial edema, lordosis, and uninflated swim bladder (Table 8). No significant difference was observed in the oxygen consumption rate of the larvae exposed to PSNAP only; however, in DDE and PSNAP + DDE, oxygen consumption rates increased significantly. The locomotor behavior of the larvae (movement, distance moved, velocity, angular velocity, and rotations) did not change after PSNAP exposure, while significant alterations (reductions) were noticed in larvae exposed to DDE alone or DDE + PSNAP (Varshney et al., 2023). The uptake of EE2, a synthetic estrogen, by zebrafish embryos was reduced by PSNAP in coexposure; however, the body length of the larvae was reduced and locomotor activity (total distance travelled) during the dark phase was suppressed (Table 8). Upregulation of *gfap* and *α1-tubulin* mRNAs (related to nervous system) by PSNAP alone or coexposed with EE2 occurred in zebrafish larvae (Chen et al., 2017a).

Zebrafish embryos were exposed to pristine PS, aged UV-PS, non-aged O3-PS, and penicillin either alone or coexposed with antibiotics (Table 8). Penicillin alone did not induce developmental toxicity (hatching, malformation, and mortality); however, accumulation of PSNAP in the yolk sac, eye, head, and nerve tubes was interrupted by penicillin coexposure (Chen J. et al., 2023). It was observed that pristine PS and penicillin coexposure synergistically suppressed heart rates and spontaneous movements of the embryos and swimming behavior and touch responses of the larvae (Chen J. et al., 2023). Except those exposed to O3-PS, ROS levels were significantly increased in PS + penicillin and UV-PS + penicillin groups resulted in induction of cellular apoptosis (apoptosis mostly seen in the embryonic tail and larval head region) (Chen J. et al., 2023). Coexposure with penicillin affected the motor behaviors (spontaneous movements, touch response, and swimming) and heart beats of the embryos during development. Upon exposure with PS, aged PS, or penicillin co-exposed with PS, neurotransmitter metabolite expressions in zebrafish larvae were significantly dysregulated (Chen J. et al., 2023).

Coexposure with simvastatin (SIM) (an anticholesterolemic drug) increased hatching rates and heart beats, while SIM alone can delay hatching, reduce heart beats, induce edema, and cause mortality after 96 h of exposure (Barreto et al., 2021). Coexposure of zebrafish embryos with sodium nitroprusside (SNP) significantly reduced the accumulation of PSNAP in the larvae and antagonized the effects induced by PSNAP (20 mg/L) during embryo–larval development (spinal curvature, organ edema, and survival rates) (Table 8; Chen Q. et al., 2023). Moreover, the activities of several enzymes including soluble guanylate cyclase (sGC), protein kinase G (PKG), caspase 3, which were regulated by PSNAP exposure, were also antagonized by SNP coexposure. The oxidative stress and ROS levels, apoptosis and ferroptosis, GPX4 (the key protein for ferroptosis) content, and the expression of several PSNAP-responsive genes including *Adma, Nos*, *Pde6d, prkg, bik, bad, bax, bim, bid*, *bok, Slc7a11, Acs14a, Keap1b,* and *Ncoa4* were also modulated by SNP exposure during embryo–larval development of zebrafish (Table 9; Chen Q. et al., 2023). Moreover, the increased proliferation of macrophages and neutrophils and the upregulation of *tnfα, tgfβ, il-4,* and *il-6* mRNAs by PSNAP were alleviated by SNP exposure in coexposed embryos (Tables 8, 9; Chen Q. et al., 2023).

In larval zebrafish, PSNAP accumulated in the intestine, pancreas, and gall bladder and disrupted glucose homeostasis with increased cortisol secretion (Table 8). Moreover, coexposure with glucose did not show any significant response (Brun et al., 2019). The locomotor activity of the PSNAP-exposed larvae showed increased activity in the dark phase; coexposure with glucose diminished the hyperactivity. It was suggested that the adverse effects of PSNAPs are at least in part are mediated by glucocorticoid receptor activation, leading to aberrant locomotor activity (Brun et al., 2019).

Zebrafish juveniles were fed with regular diet, high-fat diet, and exposed to 1,000 μg/L PSNAP (50–1,000 nm diameter) either to fish fed with normal diet or fed with high-fat diet (Supplementary Table S1). The feeding with regular diet and high-fat diet has been done for 3 weeks, while for PSNAP exposure, it was only for 1 week. Despite no effects on the body weight, it was observed that PSNAP exposure perturbs lipid metabolism and gut microbiota stability in zebrafish (Du et al., 2024). Combined exposure of PSNAP with high-fat diet resulted in gastrointestinal injury and reduced the number of goblet cells in the intestinal layer (Du et al., 2024). The CAT activity increased, and MDA content decreased, while SOD activities remained unaltered in the liver of zebrafish after PSNAP exposure (Du et al., 2024). Moreover, the mRNA expression of *cpt1ab* was upregulated, that of *fasn* was downregulated, and that of *hmgcra* remained unaltered after PSNAP exposure (Du et al., 2024).

In adult zebrafish, the toxic potentials of PSNAP were also evaluated in the presence of other environmental pollutants, including arsenic, BPA, diethylstilbestrol (DES), homosolate, lead, MCLR, 4-nonylphenol (4-NP); oxytetracycline, triphenyl phosphate (TPhP), tris (1,3-dichloro-2-propyl) phosphate (TDCIPP), and vit D (Tables 8). Moreover, the expressions of several genes related to metabolism, immunity, oxidative stress, apoptosis, neurobehavior, reproduction, and growth were also evaluated (Table 9). Furthermore, the intergenerational effects of PSNAP exposure were also evaluated in some of these experiments in coexposure (Wu et al., 2021; Zhu et al., 2021).

During coexposure, the accumulation of PSNAP in different organs of adult zebrafish was interrupted by the presence of coexposed chemicals. For example, PSNAP nonlinearly enhanced the accumulation of TDCIPP in the whole fish (body burden) as well as in the eggs (ovary), and the order of accumulation was gut > gills > gonad > liver. The accumulation of TDCIPP in female fish tended to be higher than that in male fish (sex-specific) (Zhao et al., 2021). Moreover, the accumulation of PSNAP in the liver of zebrafish was independent of MCLR, while accumulation of MCLR in the liver of zebrafish was enhanced by PSNAP exposure (Ling et al., 2022). In addition, PSNAP exposure enhanced the accumulation of BPA in viscera, gills, head, and muscle of zebrafish (Chen et al., 2017b) and As in the intestine and brain. Accumulation of homosolate in the testis, ovary, liver, and brain of male and female fish was enhanced by PSNAP exposure (not significant). Coexposure with As or OTC has no effect on mortality (Zhang et al., 2023); however, exposure to TPhP alone was highly toxic to zebrafish (LC_50_ was 976 μg/L). It was also observed that Pb enhanced the accumulation of PSNAP in the intestine, while excessive Pb reduced the accumulation (Yu J. et al., 2022).

The effect of PSNAP in coexposure with Pb, As, and OTC was evaluated in intestines of adult zebrafish (Yu J. et al., 2022; Zhang et al., 2024c). The intestinal villi were swollen, and the ratio of the villus height/crypt depth or the ratio of the villus height/villus width were decreased in fish exposed to As either alone or in combinations (Zhang et al., 2024c). Moreover, exposure of the fish to OTC alone caused damage of the lining epithelium of intestinal villi and vacuolation of intestinal epithelial cells, while coexposure with PSNAP alleviated the processes (Ye et al., 2024). There are seven types of cells found in the intestine (enterocytes, macrophages, neutrophils, B cells, T cells, enteroendocrine cells, and goblet cells) of adult zebrafish, and PSNAP and Pb exposure influenced enterocytes, macrophages, B cells, T cells, and goblet cells during coexposure (Yu J. et al., 2022). The PSNAP exposure induced the effects on macrophages by affecting the expressions of genes related to immunologic (*ctsba, nfkbiab*, and *pycard)* and apoptotic processes, while Pb exposure influenced the enterocytes by altering genes related to oxidative stress (*gsta2, gsto 1, gsto2, gpx1a,* and *mgst1.2)* and lipid metabolism. Consequently, in coexposure, the effects induced by PSNAP on macrophages were decreased by Pb, while in enterocytes, the Pb-induced effects were decreased by PSNAP exposure (Yu J. et al., 2022). In B and T cells, upregulation of *hsp70.1, hsp70.*2, and *hsp70.3* occurred in fish exposed to PSNAP and Pb alone, and also in coexposure (Table 9; Yu et al.). The 8-hydroxy-2′-deoxygluconate (8-OHdG) and TNF-α levels were enhanced in the intestine by Pb exposure, and PSNAP synergized the effects. As, either alone or in combinations, markedly increased ROS and decreased GSH content in the intestine, while SOD activity and MDA content remained unaltered. The mitochondrial DNA copy number significantly reduced in fish exposed to PSNAP or As, either alone or in combinations. Moreover, 5-HT level in the intestine was decreased by As in coexposure, while in serum, it (5-HT) remained unaltered (Zhang et al., 2024c). The mRNA (*tph1a, tph1b*, and *tph2*) expressions of tryptophan hydroxylase (TPH), the rate-limiting enzyme for 5-HT synthesis, tended to downregulate in fish exposed to PSNAP and As either alone or in combinations (Zhang et al., 2024c). The intestinal microbiota was also altered by Pb, As, and OTC, either alone or in coexposed conditions (Yu Z. et al., 2022; Zhang et al., 2024c).

The effect of PSNAP in coexposure with TDCIPP, BPA, MCLR, and vit-D (dietary) was evaluated in the liver of adult zebrafish (Zhao et al., 2021; Ling et al., 2022; Li Y. et al., 2023). The HSI was increased by PSNAP and remained unaltered when fed with vit D (Li Y. et al., 2023), while MCLR induced cellular swelling, fat vacuolization, and cytoarchitecture of the organ, and coexposure with PSNAP exacerbated the effects (Ling et al., 2022). The biochemical analysis showed that MCLR alone enhanced ROS and MDA contents and reduced GSH and CAT activities in a concentration-dependent manner, while coexposure with PSNAP aggravated the effects (Ling et al., 2022). Consequently, gene expressions related to antioxidant mechanisms (*p38a, p38b, ERK2, ERK3, Nrf2, HO-1, cat1, sod1, gax, JINK1*, and *gstr1*) remained unaffected after PSNAP exposure, while MCLR enhanced the expression of several genes (*ERK2, ERK3, p38a, Nrf2, gpx1a, gstr1, at1,* and *sod1*) in a concentration-dependent manner, and coexposure with PSNAP exacerbated the expression of *Nfr2* (Ling et al., 2022). TDCIPP alone or in combination with PSNAP upregulated the expressions of thyroglobulin (*tg*) and uridine diphosphate glucuronosyltransferase (*ugt1ab*) genes in the liver of female zebrafish. Moreover, the expressions of deiodinase 1 (*dio1*) and transthyretin (*ttr*) were downregulated, and the expression of deiodinase 2 (*dio2*) gene was upregulated in female fish exposed to TDCIPP either alone or in combination with PSNAP (Zhao et al., 2021). In the liver of male fish, the transcription of *tg* and *ugt1ab* genes was upregulated in fish exposed with TDCIPP alone or in combinations. Moreover, the expression of *trβ* remained unaltered in all the experimental groups, while *trα* expression in the liver of male fish was upregulated when exposed to TDCIPP alone or in combinations with PSNAP. In addition, a significant downregulation of *ttr* expression was observed in the liver of male fish exposed to TDCIPP either alone or in combinations (Zhao et al., 2021). Vit D altered the number of lipid droplets as well as the triglyceride and total cholesterol contents in the liver (Li Y. et al., 2023). Moreover, inconsistent effects were observed in CAT and SOD enzymatic levels and MDA contents in the liver. A nonlinear increase in the gene hydroxy-3-methylglutaryl-coenzyme A (*hmgcra*), sterol regulatory element binding protein (*srebp1*), diacylglycerol acetyltransferase 1b (*dgat1b*), acetyl coenzyme A carboxylase (*acc*), and carbohydrate response element binding protein (*cvhrebp*) by PSNAPs in the liver was ameliorated by high vit D diet (2800 IU/kg); in contrast, the expression of carnitine palmitoyl transferase 1 (*cpt1)* was decreased significantly by PSNAPs and was increased by vit D.

The effects of PSNAP in coexposure with BPA, TDCIPP, NP-4, and As were evaluated in the brain of adult zebrafish (Chen et al., 2017b; Zhao et al., 2021; Aliakbarzadeh et al., 2023; Zhang et al., 2023). It was observed that in the brain, similar to PSNAP, BPA alone can inhibit AChE activity and upregulate myelin basic protein (MBP) gene expression, while coexposure upregulated the expressions of myelin and tubulin protein/gene, dopamine content, and the mRNA expression of mesencephalic astrocyte-derived neurotrophic factor (MANF). However, AChE activity in the brain remained unaltered by coexposure (Chen et al., 2017b). Therefore, PSNAP by increasing the BPA concentration in the brain induced neurotoxic effects through a mechanism other than AChE inhibition (Chen et al., 2017b). TDCIPP alone can interrupt the thyroid hormone-dependent mechanisms in the brain of adult zebrafish. In female fish, the transcription of corticotropin-releasing hormone (*crh*) was upregulated in a nonlinear fashion in fish exposed to TDCIPP either alone or in combinations. However, the transcription of *tshβ* remained unaltered in fish exposed to PSNAP and TDCIPP either alone or in combinations. In the brain of male fish, transcription of *crh* and *tshβ* increased only in coexposed fish (TDCPP + PSNAP). The enzymatic activities of CAT, AChE, glutamine synthase, and GSH contents in the brain were reduced by 4-nolnynphenol (4-NP), either alone or in coexposure. However, the glutamate dehydrogenase activity in the brain was found to increase in fish exposed to PSNAP either alone or in combination with 4-NP (Aliakbarzadeh et al., 2023). The metalloid As was able to cross the blood–brain barrier and accumulated in the brain and enhanced ROS production by increasing the SOD activity and MDA content and decreasing the GSH levels. As a result, microthrombi were observed in the brain, and the mitochondrial DNA significantly reduced; the expressions of genes related to mitochondrial synthesis (*pgc1-a* and *pgc1-b*) and fusion (*mfn1a, mf1b*, and *opa1*) were downregulated, while those of the genes related to mitochondrial division (*drp1, mff, fis 1, mid49,* and *mid51*) were upregulated (Zhang et al., 2023). Moreover, the expressions of genes related to mitophagy (*ulk1a*, *parl, parkin*, *pink 1* and *fundc1*) were upregulated. The neurotransmitter dopamine (DA) activity significantly decreased, and ACh activity increased. The activity of neurotransmitter catabolic gene *mao* was significantly downregulated, and the activity of MAO was significantly decreased, and the activity of AChE significantly increased in the brain of fish exposed to As. The expression of *ache* mRNA in the brain was upregulated, while 5-HT level in the brain was significantly reduced. PSNAP was able to promote the accumulation of As in the brain of adult zebrafish and potentiated most of the effects induced by As alone (Zhang et al., 2023). Moreover, PSNAP when coexposed with As decreased the swimming speed and induced anxiety-like behavior and affected learning and memory of the adult zebrafish (Zhang et al., 2024c).

The effect of PSNAP in coexposure with TPhP, TDCIPP, DES, and homosolate was evaluated in the gonads and hormone levels of adult zebrafish (He et al., 2021; Zhao et al., 2021; Lin et al., 2023; Ye et al., 2024). TPhP alone enhanced liver weight (HSI) and ovarian weight and disrupted spermatogenesis and oogenesis as well as the histological structure of the testis and ovary (He et al., 2021). Moreover, TPhP alone did not significantly disrupt the sex steroid levels (E2 and T), and thus the VTG content in male fish, even though VTG decreased in female fish (He et al., 2021). The fecundity (total eggs produced during the experimental period) of the fish decreased in fish exposed to TPhP alone (He et al., 2021). Coexposure of PSNAP along with TPhP (PSNAP + TPhP) increased HSI and GSI and reduced VTG content in both male and female fish. Moreover, coexposure also inhibited spermatogenesis with structural derangements (formation of lacunae and interstitial tissue) in the testis and induced follicular atresia (atretic follicles) in the ovary (He et al., 2021). The E2 level in male fish enhanced, while T level remained unaltered in both male and female fish in coexposure (He et al., 2021). The fecundity significantly reduced, and the number of spawning events, fertilization, and hatching rates of the embryos were also reduced (He et al., 2021). The synthetic estrogen, DES, like TPhP, decreased HSI and GSI in both male and female fish. Moreover, in the testis, DES alone or in coexposure induced lacunae and increased the number of spermatogonium and spermatocytes and induced the deformation of seminiferous tubules (Lin et al., 2023). In female fish, PSNAP and DES exposure showed more preovulatory oocytes and smaller mature oocytes. The levels of E2 and T in PSNAP- and DES-exposed fish either alone or in coexposure decreased in both male and female zebrafish (Lin et al., 2023). However, DES alone or in combination with PSNAP increased the E2/T ratio in a concentration-dependent manner in male fish. In female fish, a concentration-dependent reduction in the E2/T ratio was observed in fish coexposed with PSNAP and DES (Lin et al., 2023). DES alone or coexposed with PSNAP enhanced the VTG content in a concentration-dependent manner in both males and female fish. PSNAP exposure has no significant effects on the T3 and T4 levels of both male and female fish; however, DES alone or in combination with PSNAP decreased both T3 and T4 contents in male and female fish in a concentration-dependent manner (Lin et al., 2023). Moreover, PSNAP and DES alone or in combination reduced fecundity, spawning events, fertilization, and hatchability of the embryos. In addition, PSNAP and DES either alone or in combination induced abnormal development (teratogenic effects) of the larvae (spinal curvature, pericardial cyst, and growth retardation) (Lin et al., 2023). Adult zebrafish exposed to TDCIPP alone or in combinations with PSNAP decreased T3 and T4 levels in female and T4 level in male fish (Zhao et al., 2021). In eggs, only T4 level (no T3) was reduced significantly when the fish were exposed to PSNAP alone and in combination with TDCIPP (concentration-dependent). A concentration-dependent reduction in the T3 level was observed when the fish was exposed in a combination of TDCIPP and PSNAP. Coexposure with homosolate, an emerging POP, did not induce any alteration in the GSI of both male and female fish; however, it resulted in higher expression of *sgk1* and promoted ovary development, while inhibiting spermatogenesis (Ye et al., 2024). Coexposure also modulated steroid hormone synthesis genes (*cyp17a2* and *hsd 17β1)* and *esr2b, vtg1,* and *vtg2* and resulted in higher E2 release in female fish. Conversely, male fish showed lower T and E2 levels and altered the expressions of *cyp11a1, cyp11a2, cyp17a1, cyp17a2*, and *hsdβ1* (Ye et al., 2024).

The intergenerational effect of PSNAP in coexposure with MCLR was evaluated in F1 embryos/larvae, which were obtained from the parents exposed to PSNAP and MCLR either alone or in combinations for 45 days (Wu et al., 2021; Zhu et al., 2021). It was observed that PSNAP was accumulated also in the F1 embryos and influenced the accumulation of MCLR (Wu et al., 2021). A concentration-dependent reduction in hatching rates, hatching enzymatic activities, and tail wagging of the F1 embryos exposed to MCLR alone or in combination with PSNAP was observed (Wu et al., 2021). Pathological alterations in somite muscles (irregular somite boundaries) were observed in F1 larvae exposed parentally to MCLR alone or coexposed with PSNAP, while no significant effect was observed on the AChE activity; however, a concentration-dependent increase in the AChE activity was observed in F1 larvae coexposed to MCLR and PSNAP. Gene expression analysis related to hatching enzymes (*tox 16, foxp1, ctslb, xpb1, klf4, cap1, bmp4, cd63, He1.2, zhe1,* and *prl*), cholinergic system (*ache* and *chrnα7*), and muscle development (*Wnt, MyoD, Myf5, myogenin, and MRF4*) indicated alterations in the F1 larvae exposed parentally to PSNAP and MCLR either alone or in combinations (Wu et al., 2021). It was also observed that, due to parental exposure (F0) to PSNAP and PSNAP + MCLR, accumulation of PSNAP was detected in the testis and ovary of the F1 larvae, and the presence of PSNAP in the environment increased the accumulation of MCLR in F1 larvae (Zuo et al., 2021). Moreover, parental exposure of MCLR and PSNAP + MCLR affects the hatchability (decreased), malformation (decreased), mortality (increased), body length (decreased), and heart rates (decreased) of the F1 larvae; even though parents with PSNAP exposure alone had no effects on the induction of developmental defects in F1 larvae. Parental exposure to PSNAP alone did not alter the T4 and T3 levels in the F1 larvae. However, MCLR either alone or in coexposure reduced T4 and T3 levels of the F1 larvae. Gene expression in the F1 larvae of the HPT axis and GH/IGF axis remained unaltered when the parents were exposed to PSNAP alone; however, the expressions of HPT axis genes (*trα, trβ, dio2, dio1, ttr, tg, tshr, nis, crh, pax8*, and *nkx2.1*), except *ugt1ab* and *tpo*, were altered in F1 larvae after parental exposure either to MCLR alone or coexposed with PSNAP. Among GH/IGF axis genes (*igf2α, igf1, gh, ghrh, ghrα, igf1ra, igf1rβ, igf2β*, and *igf2r*), only *igf1, igf2α,* and *ghrβ* altered in F1 larvae when the parents were exposed to MCLR + PSNAP (Zuo et al., 2021).

## 4 Discussion

In the systematic review, our search strategy collected literature on eight plastic polymers (PA, PC, PE, PET, PMMA, PPP, PS, and PVC) (Table 1) studied on 13 fish species, consisting of 114 articles (Figure 1; Table 2). The effects of the plastics on fish were evaluated either alone or when coexposed with other environmental pollutants, including heavy metals, POP, drugs, and bacteria. The accumulation of NAPs by fish was also influenced by the surface charge of the plastics and environmental conditions (temperature, pH, and diet). The information collected on plastic toxicity summarized from all these literatures was assembled in Supplementary Table S1 and deposited at Figshare (www.figshare.com) for reference and future upgradation, if needed.

Our strategies found a wide variation in the diameters of the plastic polymers used in these studies. Although the size of the MIPs is usually considered to be < 5,000,000 nm (5,000 µm), the size of NAPs has not yet achieved a consensus, with some considering it to be < 1,000 nm and others <100 nm (Torres-Ruiz et al., 2021). During the review, we considered the size/diameter of the NAPs as ≤100 nm and excluded 15 (13 + 2) articles, where the sizes of the studied plastic particles were >100 nm (Table 3). In addition, the diameter of the studied plastics (PE, PPP, PET, and PS) in 26 articles was ≤100 nm as well as > 100 nm (Table 4). In these studies, we have considered the effects observed on the plastic sizes ≤100, and the effects found on diameters >100 nm were excluded (Table 4). Moreover, our review focused mostly on whole/intact animals and embryos; therefore, the studies performed *in vitro* were also excluded from this review (Greven et al., 2016). In addition, in 48 articles, NAPs were coexposed with various environmental pollutants (Table 8). Moreover, in some studies, modifications in diet (high-fat diet) and environmental conditions (temperature and pH) were made. Considering all these variations, we have finally selected 101 (99 + 2) articles for review (Figure 1; Table 5).

Our findings revealed that among the five plastic polymers (PE, PET, PMMA, PPP, and PS), the studies were limited either to plastic types or the developmental stages (embryos, larvae, juveniles, and adults) of the fish (Table 5). For example, effects of PE/LDPE were studied on embryos and adults of zebrafish and juveniles of common carp; PET and PMMA were found on embryos of zebrafish, PPP in juveniles of tilapia and zebrafish, and PS on grass carp (embryos, larvae, and juveniles), silver carp (adults), tooth carp (adult), fathead minnows (adult male), Chinese rice fish (adults), Japanese medaka (larvae and adults), marine medaka (embryos, larvae, juveniles, and adults), rainbow trout (juveniles), Nile/red tilapia (larvae and juveniles), Mozambique tilapia (larvae), and zebrafish (embryos, larvae, and adults). Moreover, most of the studies on fish were focused on the effects of PS (∼89%), probably because of their wide availability and a well-characterized research material that can be manufactured with a large range of particle sizes, fluorescence labeling, as well as various surface modifications (Torres-Ruiz et al., 2021; Xu et al., 2022). In addition, among thirteen fish species, our search strategies found that zebrafish was the most studied fish (78 articles out of 114; ∼69%) than any other fish species included in this review. However, despite wide arrays of variability in the mode of exposures (waterborne, trophic transfer, dietary, injections, or coexposure with other environmental pollutants) and durations and concentrations, the study showed bioaccumulation of NAPs on chorion and embryos during embryo–larval development as well as in the gill, gut/intestine, liver, kidney, gonads (testis and ovary), muscle, and brain of larvae, juveniles, and adult fish. Moreover, accumulation of NAPs in the tissues/organs of fish induced multiple biological effects including body and bone morphology, teratogenic, cardiac, oxidative stress, inflammatory, genotoxic, hepatotoxic, neurotoxic, behavioral, reproductive, endocrine disruptions, and an intergenerational impact (Tables 5–9). In coexposure experiments, the combined effects of NAP and other environmental pollutants on fish can be observed as synergistic or antagonistic, while no influence of some of the chemicals was also noticed (Table 8). Our studies agree with the concept that in fish, NAPs due to their small size are able to penetrate tissues by crossing the biological barriers (chorions in the embryos and gill, skin, and gut in larvae, juveniles, and adults), as observed in humans (lung, skin, and gastrointestinal barriers in humans) and can induce toxicogenomic effects at the cellular level (Lehner et al., 2019; Mantovani et al., 2019). Although the bioaccumulation of NAPs in fish was evident from our literature survey, the data on LC_50_, NOEC, or LOEC are very limited. The 96 hpf NOAEL as determined on PE (hydrodynamic size 191.10 ± 3.13 nm; Sun et al., 2021) in zebrafish embryos was 50 μg/L, the 96 h LC50 for PS (diameter 100 nm) on zebrafish embryos (24 hpf) was 431.1 mg/L (Feng et al., 2022), while in tooth carp adults (PS, average diameter was 185 nm), it was 19.3 mg/L (Saemi-Komsari et al., 2023), which are significantly higher than the plastic concentrations found in the aquatic environments (Mojiri et al., 2024).

Oxidative stress and inflammation are the two major pathways commonly affected by exposure to NAPs in fish (Brun et al., 2019). Engineered nanoparticles are known as potent inducers of immune and inflammatory responses as well as for the generation of reactive oxygen species (Khanna et al., 2015). Although we have limited the diameter of NAPs to ≤ 100 nm (minimum is 15 nm), our literature survey showed that small NAPs can reach internal organs (brain, eyes, liver, pancreas, and heart), and comparatively larger particles accumulated in the gut, gill, and skin of fish (Table 5). In embryos, NAPs after crossing the chorion (probably through chorionic pores) were initially accumulated in the yolk sac and later transported to various organs, including the GI tract, liver, pancreas, gall bladder, kidney, heart, and brain (Table 6; Supplementary Table S1); while, in larvae, juveniles, and adults, the accumulation was initially observed on the gill, skin, and gut and then gradually transferred to the liver, pancreas, kidney, gonads, and brain. Consequently, as a part of the detoxification process (mediated by cytochrome P450-dependent mechanisms), the Oxidative stress induced, resulted in cellular apoptosis, histological damage in the accumulated organs, and activated immunomodulatory mechanisms. Accordingly, the genes belonging to these pathways were functional and controlled the processes as well (Aschner et al., 2025).

Oxidative stress is a key putative mechanism of NAPs causing imbalance of ROS (Sharpton, 2018), which is an intracellular chemical species that contain oxygen (O_2_) and are reactive toward lipids, proteins, and DNA (Glasauer and Chandel, 2013). Excessive ROS is a major cause of oxidative damage and weakens the immunity of fish (Ding et al., 2018; Sun et al., 2019). Enzymatic antioxidants such as SOD and CAT participate in protecting organisms from excesses of ROS, which was induced by exposure to xenobiotics (Mates, 2000). SOD encompasses mitochondrial Mn-SOD and cytosolic SOD (Cu and Zn-SOD) enzymes that convert the superoxide anion into H_2_O_2,_ which was then converted by CAT into water and oxygen (Abele et al., 2011). The impairment of these oxidative enzymes damaged the cell membrane and DNA, resulting in a loss of defense capability (Matos et al., 2019). Both in embryos and adult fish, the major oxidative enzymes are CAT, SOD, GPx, GST, and the GSH and MDA, which were used as important biomarkers for NAP toxicity. The oxidative stress index (based on CAT, peroxidase, and SOD activities and GSH and MDA contents) was found to be increased in fish after NAP exposure (Bhagat et al., 2022; Chen J. et al., 2023). Our review indicated that the plastic particles we surveyed (PE, PET, PMMA, PPP, and PS) have the potential to regulate oxidative stress and ROS in the fish. Therefore, oxidative stress, calculated as the oxidative stress index, should be considered a potential indicator of NAP toxicity.

Our literature search also indicated that the effects of NAPs on gene expression analysis were observed in 33 articles (∼29%) and restricted only to PS (Tables 7, 9). No other plastic types were used for gene expression analysis. Moreover, in larvae (Mozambique tilapia and zebrafish), juveniles (grass carp and Nile tilapia), and adults (FHM, marine medaka, and zebrafish), the gene analyses were also restricted to PS, and the studied organs were gut/intestine (grass carp, marine medaka, Nile tilapia, and zebrafish), liver (FHM, marine medaka, Nile Tilapia, and zebrafish), kidney (FHM), ovary (zebrafish), brain (zebrafish), and muscle (marine medaka) of the fish (Table 7).

Our studies indicate that in zebrafish embryos, PSNAP either alone or in coexposure upregulated several genes which belonged to membrane transport, detoxification, oxidative stress, apoptosis and ferroptosis, inflammation, base excision pathways, VEGFA/VEGFR pathways, and also related to the liver, vasculature, nervous system, visual system, and HPT and HPG axis (Tables 7, 9), while downregulation of several genes was related to membrane transport, apoptosis*,* steroidogenesis, neurodegeneration and motor dysfunction, visual system*,* epigenome*,* VEGFA/VEGFR pathways, osteogenesis*,* thyroxin transport, and synthesis*.* Moreover, several of the studied genes belonged to detoxification, visual system, oxidative stress, metallothionein, DNA damage, and mitochondrial metabolism, and the central nervous system development remained unaltered (Tables 7, 9). In larvae, juveniles and adults, gene regulations were organ-specific and mostly related to the functions of the organs. Moreover, as in embryo–larval development, in coexposure with environmental pollutants, synergistic/antagonistic or no significant effects in gene expressions were observed (Table 9). In the gut/intestine, the gut microbiota played a significant role in gene regulations, which could be synergistic/antagonistic to the effects induced by PSNAP in other organs. The expressions of several genes related to oxidative stress and immunomodulation (*IL-6, IL-8, IL-10, IL-1β, TNF-α,* and *INF-γ2*) were upregulated by PSNAP (Li Z. et al., 2024). Moreover, in macrophages of the intestine, immune system-related DEGs (*ctsba, nfkbiab*, and *pycard*) were significantly altered by PSNAP exposure, and the genes related to MAPK signaling pathways (*hsp70.1, hsp70.2*, and *hsp70l*) remained unaltered. In intestinal enterocytes, genes related to GSH metabolism (*gsta2, gsto1, gsto2, gpx1a,* and *mgst1.2*) and cytochrome P450 remained unaltered. In intestinal B and T cells, upregulation of *hsp70.1, hsp70.*2, and *hsp70.3* was observed in fish exposed to PSNAP.

In the liver, in addition to immunomodulation, lipid synthesis-related genes (*fasn, srebf1*, and *pparg*), and lipid transport-related genes (*cetp* and *ldlr*) were upregulated, and the lipid degradation-related genes (*atg1, ppara*, and *aco)* were downregulated (Li X. et al., 2024). The genes of the Toll-like receptor 4 (TLR4) pathway (*irf3, irak4, traf6,* and *tbk1*) in the liver showed a trend of upregulation, while muscle development-related gene (*myog, myod, mstn, myf5,* and *fgf6b*) expressions were downregulated, and no alteration was observed in creatine kinase and *hif1α* contents after PSNAP exposure (Pitt et al., 2018b; Sarasamma et al., 2020).

In the brain, the development of microthrombi in the basement membrane of the blood–brain barrier, a well-known toxicogenomic index, was associated with the downregulation of mitochondrial fusion-related genes (*mfn1a, mf1b*, and *opa1*), while the mitochondrial division-related genes (*drp1, mff, fis 1, mid49,* and *mid51*) showed a tendency of upregulation (Zhang et al., 2023). The expressions of genes related to mitophagy (*ulk1a*, and *parl*) were also upregulated by PSNAP exposure. Moreover, among the 5-HT receptor mRNAs, *htr1aa, htr1ab,* and *htr2c* were significantly upregulated, while the expressions of *htr1b* and *htr4* showed downregulation in the brain of fish.

In zebrafish, PSNAP have the potential to accumulate in the gonads (testis and ovary), disrupted endocrine functions, impaired gametogenesis, interfere with intergenerational inheritance and thus embryonic development, and modulated the gene expressions related to hatching enzymes (*tox 16, foxp1, ctslb, xpb1, klf4, cap1, bmp4, cd63, He1.2,zhe1,*and *prl*), cholinergic system (*ache* and *chrnα7*), and muscle development in F1 offspring (*Wnt, MyoD, Myf5, myogenin, and MRF4*) (Wu et al., 2021). The molecular mechanisms underlying these effects, including oxidative stress, inflammation, and epigenetic modifications, highlighted the complex and multifaceted nature of NAP toxicity.

Taken together, even though much work remains to be done, our systematic review analysis on the effects of NAP on fish embryos and adults together with genetic analysis *in vivo* revealed a toxicity pathway starting with the particles entering the cell and inducing oxidative stress and immune responses that generated inflammation. Further intrusion of NAPs on the organelles such as mitochondria induced alterations in energy (carbohydrate) metabolism. The accumulation of NAPs in different organs was dependent on size, concentrations, and durations, influenced on specific neurobehavioral, cardiac, lipid metabolism, reproduction, and intergenerational inheritance.

Plastic pollution is a global problem and poses a significant threat to ecosystems, wildlife, and human health, with plastics taking hundreds of years to decompose in the environment. Several countries have recently introduced regulations and legislations focused on plastic. These are primarily aimed to reduce the consumption and improve waste management; however, attention should be given to plastic production. More than 60 countries have implemented bans and levies on plastic packaging and single-use waste. In 2018, the European Commission published its strategy to reduce usage of single-use plastics, followed by legislation in the form of the Single-Use Plastics Directive. In 2021, the EU has levied a “plastic tax” on all unrecycled plastic waste generated within the region. The EPA’s “National Strategy to Prevent Plastic Pollution” aims to eliminate the release of plastic waste into the environment by 2040. However, despite all these regulations and rules, we may all be aware of the problem and cooperate to implement the government policies to reduce plastic pollution in the environment.

## 5 Conclusion

Our systematic review has synthesized current knowledge on the toxicogenomic effects of NAPs in fish, using them as a model to assess the potential health risk to humans. Although methodological challenges and the limited scope of studies in plastics beyond PS remain, our findings indicate that the toxicity of NAPs can be influenced by several factors, including particle size, exposure duration, exposure route, tissue accumulation, and the chemical composition of plastics. Furthermore, NAPs pose risks to various organs through mechanisms such as oxidative stress, immune system modulation, and specific organ effects, including neurotoxicity, cardiotoxicity, genotoxicity, teratogenesis, endocrine disruption, energy metabolism alterations, and intergenerational inheritance. Despite the variability in fish species, sizes and types of the plastics, surface charge, environmental conditions, exposure routes, duration of exposure, and developmental stages of the experimental fish, our review highlights that NAPs can cross the biological barriers and gradually accumulate in the various parts/organs of the body in a non-specific manner. This accumulation occurs over time, further emphasizing the complex and potentially widespread impact of NAP exposure on aquatic organisms, with implications for human health. In summary, NAPs possess significant adsorptive properties and serve as vectors for other environmental contaminants, potentially exerting synergistic, antagonistic, or neutral effects on the tissues and organs of fish. The biotransformation process activates oxidative stress-dependent mechanisms, which in turn induce specific gene regulatory responses. In the gut/intestine, the toxicogenomic responses to NAPs exhibited either synergistic or antagonistic interactions with the gut microbiota. Intergenerational transfer of NAPs has been shown to disrupt embryo–larval development in the F1 generation. Although significant knowledge gaps remain, our systematic review addresses several critical scientific questions regarding the toxicological effects of NAPs, paving the way for future research into their environmental and health impacts.

## Data Availability

The original contributions presented in the study are included in the article/Supplementary Material; further inquiries can be directed to the corresponding author.

## Glossary

Ach: Acetylcholine
AChE: Acetyl cholinesterase
αKGPD: alpha keto glutarate dehydrogenase
APAP: Acetaminophen
AVO: Avobenzone
FHM: Fathead minnows
BAP: Benzo [a] pyrene
BDE-47: Polybrominated diphenyl ethers
BFCOD: 7-benzyloxy-4-trifluoromethyl-coumarin *O*-dibenzyloxylase
BMDMB: butyl methoxydibenzoylmethane
BPA: Bisphenol A
CAT: Catalase
CCV: Common cardinal vein
CO: Cardiac output
*c3*: Complement component 3
DES: Diethylstilbestrol
DA: Dopamine
DPH: Diphenhydramine
dph: Day post hatch
EROD: 7-Etoxyresorufin *O*-deethylase
EE2: 17 α-Ethynyl estradiol
FHM: Fathead minnows
GABA: Gamma-aminobutyric acid
GAD: Glutamic acid decarboxylase
GDH: Glutamate dehydrogenase
GI-tract: Gastrointestinal tract
GR: Glutatione reductase
GS: Glutammine synthetase
GSH: Glutathione
GSSG: Oxidized glutathione
GST: Glutathione-S-transferase
HSI: Hepatosomatic index
HPT: Hypothalamus–pituitary–thyroid
5-HT: Serotonin
ISV: Intersegmental vessel
LDPE: Low-density polyethylene
LOEL: Lowest observed effect level
LZM: Lysozyme
*mst1*: Macrophage-stimulating factor 1
MAO: Monoamine oxidase
MAPs: Macroplastics
MCL: Microcystin-LR
MIPs: Microplastics
NAPs: Nanoplastics
*ncf2*: Neutrophil cytosolic factor 2
No: Nitric oxide
NOAEL: No observed adverse effect level
Noel: No observed effect level
*nox2*: NADPH oxidase 2
PC: Polycarbonate
PCP: Personal care products
PE: Polyethylene
PLA: Polylactic acid
PMME: Polymethylmethacrylate
PEMIP: Polyethylene microplastics
PENAPs: Polyethylene nanoplastics
PET: Polyethylene terephthalate
PETNAPs: Polyethylene terephthalate nanoplastics
PHE: Phenanthrene
PHN: Phenmediphamµ
POP: Persistent organic pollutants
PP: Polypropylene
PPAR: Peroxisome proliferator activator receptor
PPPMIP: Polypropylene microplastics
PS: Polystyrene
PSMIPs: Polystyrene microplastics
PSNAPs: Polystyrene nanoplastics.
PU: Polyurethane
PVC: Polyvinyl chloride
SIM: Simvastatin
SIVP: Sub-intestinal venous plexus
SMX: Sulfamethoxazole
SMZ: Sulfamethazine
SNP: Sodium nitroprusside
SOV: Superficial ocular vessels
T-AOC: Total antioxidant content
TC: Tetracycline
TCS: Triclosan
TDCIPP: Tris 1,3-dichloro-2-propyl phosphate
TG: Thyroglobulin
TGL: Triglyceride
TLR: Toll-like-receptor
TPH: Tryptophan hydroxylase
TPhP: Triphenyl phosphate
VEGFA: Vascular endothelial growth factor
VEGFR: Vascular endothelial growth factor receptor
vit D: Vitamin D
VTG: Vitellogenin
wph: Weeks post hatch
