# Supplementary material for: A systematic review of the effects of nanoplastics on fish
Source: Front Toxicol. 2025 May 30;7:1530209. doi: 10.3389/ftox.2025.1530209 (PMC12163001; doi:10.3389/ftox.2025.1530209)
Supplement: Supplementary file 1 [file DataSheet1.pdf]

Table ST 1: Supplementary data

Fish	Plastic (Polymers)	Developmental stage	Treatment conditions	Effects	References
Common carp (*Cyprinus carpio*)	Polyethylene (PE): Macro plastic (>5 mm); microplastic (100 nm- 5 mm); and nanoplastics (<100 nm)	Juvenile (length 5.5±1 cm; weight 4±1 g)	Fish were exposed to 100 mg/L of macro- micro- and nanoplastic exposed for 15 days).	i) The AChE and MAO activities and the NO concentration decreased significantly in all three (macro-, micro-, and nano)- plastic concentrations<br>ii) In the tectum (brain), varying degrees of necrosis, fibrosis, changes in blood capillaries, tissue detachment, edema, degenerated connective tissue, necrosis of large cerebellar neurons, and ganglion cells were observed.<br>i) In retina, plastic exposure (macro-, micro-, and nano-) induced necrosis, degeneration, vacuolation, and curvature in the inner layer.	Hamed et al., (2022)
Carp	Polystyrene (PS) (50, 100, 400 nm)	Adult	1000 µg/L exposed waterborne, 28 days.	ii) Induced myocardial injury (massive blood cells, and broken tissue fragments seen); the gap between cardiomyocytes increased and the structure and texture of	Wu et al., (2022)

				myocardial tissue were unclear.<br>iii) The smaller the particle size of PSNAP, the damage to the myocardial tissues are more severe.<br>iv) PS exposure increased the apoptosis in the cardiac myocytes<br>v) Increase in the protein content of TLR4 and NOX2 after PS exposure<br>vi) Promoted the levels of H_2O_2 and MDA in myocardial tissue and inhibited the antioxidant capacity (CAT, SOD, GPx enzyme activity and GSH and T-AOC content) in myocardial tissue<br>vii) Induced imbalance in Th1/Th2 levels (shift to Th1)<br>viii) expression of the proinflammatory cytokines (TNF-α, INF-γ, IL-6, IL1β, and iNOS) were enhanced, while reduction in the expression of anti-inflammatory cytokines (IL-4, and IL-10) by PSNAP.	

				ix) Disruption of IGFB3/p53/AChE signaling pathways by PSNAP exposure in myocardial tissues of the carp<br>x) AChE activity increased by PSNAP in the myocardial tissue<br>xi) Disrupted the apoptosis related pathways by PSNAP (BCL2, caspase 3, caspase 9).	
Grass carp (*Ctenopharyngodon Idella*)	Polystyrene (PS), diameter 23.03±0.266 nm (20-26 nm)	Juveniles	i) 0.04 ng/L, 34 ng/L, and 34 µg/L for 20 days	i) Concentration-dependent reduction in visceral somatic index and enhancement in hepatosomatic index.<br>ii) No effect was observed in the total protein, carbohydrate, and lipid content of the brain after PS exposure,<br>iii) Did not affect overall locomotor activity<br>iv) Increased AChE activity and cerebral lipid peroxidation in brain, however, no change in nitrate production was observed.<br>i) Accumulation of PS in the brain was found in a concentration-dependent increase after PS exposure	Guimaraes et al., (2021)

Grass carp (*Ctenopharyngodon idella)*	PS (23.03±0.266 nm) Yellow-green, fluorescent	Juveniles	PS= 760 µg/L;<br>ii) ZnO=760 µg/L; alone and coexposure; for 72 h	i) No effects on behavioral tests (swimming speed, anxiety-like behavior, anxiogenic-like behavior)<br>ii) PS, ZnO, alone or with coexposure have affected the response on mirror tests (longer immobility time and shorter interaction with their images)<br>iii) Any of the treatments did not change the biochemical parameters (total carbohydrate, proteins, and triglycerides in the liver, and total carbohydrate, and protein contents in the brain).<br>iv) PSNAP either alone or in combination with ZnO stimulated the antioxidant activity of the brain (increase in GSH content, SOD activity, diphenyl-1-pycrilhydrazil [DPPH] radical scavenging activity<br>v) Increased (PSNAP and ZnO) thiobarbituric acid reactive substances and H_2O_2 production in the	Estrela et al., (2021)

				brain; however, no effect on NO production.<br>vi) PSNAP either alone or in combination with ZnO increased AChE activity in the brain<br>vii) PSNAP alone or in combination with ZnO induced DNA damage in erythrocytes.	
Grass carp (*Ctenopharyngodon idella*)	PS (80 nm) +TC	Juveniles	PS=20, 200, 2000 µg/L; TC=5000 µg/L).<br>iii) Single exposure and coexposure (7 days)	i) Coexposure significantly enhanced the total antioxidant capacity (T-AOC) and the activities of CAT and SOD in the liver and intestine of the grass carp in a concentration-dependent manner.<br>ii) Induced lesion in the gills and intestine in all treatment groups<br>iii) Upregulation of *MMP2, MMP9* and *IL8* in the liver and intestine of the co-exposed fish in a concentration-dependent manner.	Liu et al., (2022a)
Grass carp (*Ctenopharyngodon idella*)	PS (0.08 and 8 µm) GFPPS (0.05 and 5 µm) and RFPPS (1 and 5µm)	Embryos (12 hpf)	i) Embryos (12 hpf) were exposed to 5, 15 and 45	i) Due to larger sizes (80 nm and 8 µm) plastics can aggregated on the chorion and unable to cross the chorion<br>ii) No embryo mortality	Zhang et al., (2022b)

			µg/L) exposed for 2, 4, and 8 h<br>ii) Larvae (24 hpf) were exposed to 10 mg/L GFPPS and RFPPS for 4 days	iii) No difference in embryonic heart rates Accumulated in the intestine of the larvae and around the nose area.	
Grass carp (*Ctenopharyngodon idella)*	PS (80 nm)	Juveniles (6.64±0.22 cm in length; 3.95±0.35 g weight)	PS=10, 100, and 1000 µg/L; exposed alone for 8 days. Coexposed with 2X10^{7}CFU/mL *Aeromonas hydrophilia* to the fish which were preexposed with PS for 5 days, and harvested 24, 48, and 72 h after infection; total exposure 6-8 days (5+3 days).	i) The LD50 of *Aeromonas hydrophilia infection* of grass carp is 7.5X10^{7}CFU/mL.<br>ii) A concentration-dependent histological damage (increase in vacuoles) of the gut of grass carp was observed by PS alone; moreover, coexposure with *Aeromonas hydrophilia* pronounced the intestinal damage induced by PS alone.<br>iii) The PS alone increased the activities of CAT, GST, SOD, LPO, and MDA concentration in the	Li et al., (2024a)

				intestinal tissues in a concentration-dependent manner, infection with *A.hydrophilia* inconsistently increased the CAT, GST, SOD, MPO activities and MDA content in the intestine of grass carp induced by the PS alone.<br>iv) Gene expression analysis of immune genes (*IL-6, IL-8, IL-10, IL-1β, TNF-α, INF-γ2* were upregulated in the intestine of the grass carp exposed to PS alone and infection with *A. hydrophilia* in PS exposed fish enhanced the gene expression observed by PS alone.<br>v) Exposure to PS and *A. hydrophilia* induced modifications in the microbial composition of the gut of the fish.	
Silver carp (*Hypophthalmichthys molitrix*)	PS (80 nm)	Adults (9.33±1.01 cm in length and	PS=10 and 1000 µg/L. Microcystin-LR= 1µg/L; alone and coexposure (8	i) The length of intestinal villi significantly shorter in co-exposure groups in a concentration-dependent manner	Zhang et al., (2024a)

		10.43±3.41 g in weight)	groups); exposed for 96 h	ii) The histopathology of the liver showed increase in hepatocyte space in a concentration-dependent manner<br>iii) The diversity and richness in gut microbiota increased after PSNAP exposure and also enhanced in coexposure experiments.<br>vi) Imbalance induced in glycerophospholipid metabolism by PSNAP alone as well as with coexposure with microcystin-LR	
Tooth Carp (*Aphaniops hormuzensis*)	Polystyrene (PS). Minimum, maximum, and average diameter is 100, 300 and 185 nm, respectively	Adults	**Experiment 1**= 1,5,10,25,50, 100, 200 mg/L of PS for 96 h waterborne or fed with 200 mg/L PS or 500 mg/kg of triclosan (TCS) for 96 h<br>**Experiment 2=** PS=.01,0.1,1, 5 mg/kg (exposed through feeding); TCS=0.01, 0.1, and 0.5 mg/kg (exposed through feeding); PS+TCS= 0.5+0.01,	i) The 96h LC50 for TCS is 0.924 mg/L<br>ii) The 96 h LC50 for PS is 19.3 mg/L<br>iii) PS (100-300 nm size) are accumulated in gut, gill, liver, muscle, and skin after 28 days dietary exposure.<br>iv) Presence of TCS did not significantly affect the uptake of PS into the tissues.	Saemi-Komsari et al., (2023)

			0.5+0.1; 0.5+0.5 mg/L (exposed through feeding and harvested on 3, 14, and 28 days after exposure)		
Fathead minnows (*Pimephales promelas*)	Polycarbonate (PC) (158.7 nm)	Adults (average weight 4.5 g) used for isolation of neutrophils for *in vitro* assay	Concentration determination interrupted by fathead minnow plasma (0.025, 0.05,0.1 and 0.2 and 100 µg/L, added during in vitro study, incubated at room temperature for 2h)	i) Significant degranulation of neutrophils by PC *in vitro* (concentration-dependent).<br>ii) Significant increase in respiratory burst (detection of ROS)<br>iii) Initiated maximum functional response (myeloperoxidase, oxidative burst, and neutrophil extracellular trap release) in a concentration-dependent manner.	Greven et al., (2016)
Fathead minnows (*Pimephales promelas*)	Polystyrene (PS) (41 nm)	Adults (average weight 4.5 g) used for isolation of neutrophils for in vitro assay	0.025, 0.05,0.1 and 0.2 and 100 µg/mL of fathead minnows (in vitro assay); incubated for 1-2h.	i) Neutrophils phagocytize larger PSNAPs and probably fragmented into smaller particles<br>ii) Significant degranulation of neutrophils by PSNAPs in vitro.<br>iii) Concentration-dependent enhancement in respiratory burst (Detection of ROS).	Greven et al., (2016)

				iv) Significant increase in degranulation of neutrophil's primary granules (concentration-dependent)<br>v) Induced significant increase in neutrophil's extracellular trap release.	
Fathead minnows (*Pimephales promelas*)	PS (50 nm) IP injected	Adult males	0.1 ml (5 µg/L) injected volume; exposed for 48 h	i) PSNAPs were observed in liver and head kidney<br>ii) In liver, significant downregulation of macrophage stimulating 1 (*mst1*) and complement component 3 (*c3*) genes.<br>iii) No effects were observed in the expression of cytosolic factor 2 (*ncf2*) and NADPH oxidase 2 (*nox2*) genes by PCNAP-exposed fish<br>iv) In the head kidney, significant downregulation of *ncf* and *mst1* genes by PSNAP was observed.<br>v) No effect of PSNAPs was observed in the expression of *nox2* and *c3* genes in the head kidney	Elizalde-Velazquez et al., (2020)
Fathead minnows	PS (50 nm) ingestion exposure (trophic transfer)	Adult males	Daphnia were exposed to PS (5µg/L)	i) PSNAPs were observed in liver and head kidney	Elizalde-Velazquez

(*Pimephales promelas*)				through green algae (*Raphidocelis subcapitata*) and the experimental fish were fed with exposed Daphnia and sacrificed after 48h exposure	ii) In liver, PSNAP exposure significantly downregulated neutrophil cytosolic factor 2 (*ncf2*) genes compared to controls<br>iii) Upregulation of *mst1* and *c3* genes and no effect on *nox2* gene in liver after PSNAP exposure were observed<br>iv) In the head kidney, significant downregulation of *ncf* and *c3* genes by PSNAP was observed<br>v) No effect of PSNAPs were observed in the expression of *nox2* and *mst1* genes in the head kidney.	et al., (2020)
Chinese rice fish (*Oryzias sinensis*)	PS (60.39, 57.45, 57.29 nm in distilled water, moderately hard water, and tris-	Adults and F1 larvae		Adults were exposed (5 mg/L) for 7 days and adults that	i) In embryos (144 hpf) and hatched larvae (0 dph) the PS-NAP is deposited in the yolk sac.	Chae et al., (2018)

	acetate phosphate medium)		that laid eggs after during exposure were exposed (5 mg/L) for 24 h after hatching.	ii) Locomotive activities were affected by PSNAP (total distance travelled by the exposed fish tended to increase, however, the total area traveled, tended to decrease)	
Hainan medaka (*Oryzias curvinotus*)	PS (80 nm) and PS + 6:2 chlorinated polyfluorinated ether sulfonate (Cl-PFAES trade name F-53B), and F-53B	Adults (length 2.85±0.17 cm; weight 440±90 mg)	i) Exposed to 200 µg/L PSNAP for 7 days without food<br>ii) Exposed to F-53B (500 µg/L) for 7 days without food<br>iii) Exposed to 200 µg/L PSNAP+ 500µg/L F-53B for 7 days	i) PSNAPS were accumulated in the gills and intestine and the presence of F-53B interferes with the accumulation of PSNAPs.<br>ii) Exposure to PSNAP, F-53B or combination of PSNAP +F-53B caused different extent of damage to the gills (fusion of the gill lamellae), liver (appearance of eosinophilic vesicles and vacuolization), and intestine (erosion of intestinal villi), while for the liver, the combined exposure group (PSNAPs+F-53B) appeared to attenuate the hepatic damage induced by F-53B alone.	Gao et al., (2023a)

				iii) The MDA content in the gills and muscle remained unaltered by PS, F-53B and combined group; in liver, significant increase only in F-53B group; in intestine, MDA content significantly increased in PS, F-53B, and combined group (equal with single exposure groups)<br>iv) The SOD activity in gill and muscle remained unaltered in all three groups: in liver, SOD remained unaltered in PS alone group, however, enhanced in F-53B and combined groups<br>v) The CAT activity in gills decreased in PS, F-53B, and combined groups, however, remained unaltered in intestine; in liver, CAT activity significantly increased only in F-53B group; in muscle, CAT activity increased in all groups, compared to controls.<br>vi) Disrupted the gut microbial community	

Japanese medaka (*Oryzias latipes*)	PS (100 nm)	Adult	i) 10, 10^4, 10^6 particles/L ($1.79589 X10^{13}$ particles/10 mg concentration) for 3 months	i) Survivability is concentration-dependent (higher concentrations are more toxic than lower concentrations)<br>ii) The enzyme activities of CAT, GPx, LZM and MDA content in testis were decreased in a concentration-dependent manner, however SOD activities showed significant enhancement only in highest concentration used in this study (decreased in 10^4 and increased in 10^6 particles/L)<br>iii) In ovaries, the activities of CAT, GPx, LZM and MDA content is almost identical with the testis, however, SOD activity was also showed significant reduction.<br>iv) Concentration-dependent inhibition in spermatogenesis (mature sperms were slightly decreased) and oogenesis (increase in primary oocytes and decrease in	Zhou et al., (2023a)

				mature spawning follicles)	
Japanese medaka (*Oryzias latipes*)	PS (100 nm)	i) Larvae (9dph)<br>ii) Adults (60 days)	i) Larvae were exposed to PSNAPs (10^{14} items/L) or 48 h<br>ii) Adults were exposed to PSNAPs (10 items/L= $5.5X10^{-12}$ mg/L; 10^{4} items /L= $5.5X10^{-9}$ mg/L; 10^{6} items/L= $5.5X10^{-7}$ mg/L for 3 months (90 days)	i) Although the mortality significantly increased in a concentration-dependent manner, the body length, body mass, and eye diameter of the survived fish did not show any significant difference after PSNAP exposure (10-10^{6} particles/L) for 90 days.<br>ii) PSNAP (100 nm) was accumulated in the gut of larvae after 48 h exposure.<br>iii) Significant concentration-dependent alterations in the gut of the adult fish (widening of the lamina propria, shortening, and swelling of villi, edema, fusion, and cracking of villi).<br>iv) The lipase, and chymotrypsin activities were significantly higher in gut of adult fish exposed to PSNAPs in a concentration-dependent manner; trypsin activities were higher in lower two doses (10, 10^{4} items/L),	Zhou et al., (2023b)

				but reduced in higher dose (10^6 items/L)<br>v) The SOD activities in gut significantly reduced, while the CAT activities and MDA content increased by PSNAP exposure<br>vi) The lysozyme activity in the gut showed an increasing tendency after PSNAP exposure, while alkaline phosphatase activity decreased in a concentration-dependent manner<br>vii) Diamine oxidase significantly increased, while d-lactate content significantly decreased in the gut of medaka after PSNAP exposure.<br>viii) The gut microbial community was altered after PSNAP exposure.	
Marine medaka (*Oryzias melastigma*)	PS<br>NAP (50 nm)<br>MIP (45 µm)	Larvae<br>(7 dph)	i) 10 µg/mL for 24 h (NAPs and MIPs)<br>ii) 2.5 µg/mL (NAPs	i) PS (NAP and MIP) were detected in the gut of the larvae (24 h exposure; 10 µg/L); gradually increase over the duration of exposure	Kang et al., (2021)

			and MIPs) for 1,7, 14 days and 4 months.	ii)<br>iii)<br>iv)<br>v)<br>vi)<br>vii)<br>viii)	Neither MIP nor NAP after 14 days of exposure had significant effect on body length, weight, or eye diameter.<br>MIP exposure showed significant increase in the volume of intestinal mucus<br>The level of diamine oxidase and d-lactate (a metabolic product of intestinal bacteria) was increased in gut after MIP exposure<br>NAP exposure increased only diamine oxidase activity in the gut<br>Apoptosis was induced in NAP rather than MIP exposure<br>NAP exposure increased the SOD, CAT, and GST activities in the gut and liver tissues, while the ROS decreased in gut and increased in liver<br>MIP exposure increased ROS decreased SOD and CAT activities, and unaltered GST activities in gut; while in liver, ROS content and the activities	

				of CAT and GST decreased, and SOD remained unaltered.<br>ix) MIP disrupt gut bacteria population more than NAP exposure.	
Marine medaka (*Oryzias melastigma*)	Plain PS (100 nm), sulfamethazine (SMZ) either alone or in combinations.	Adults were exposed to PS (5 mg/g), SMZ (0.5 and 5 mg/g) either alone or in combination via food for 30 days	The measured concentrations of SMZ in food was 0.28±0.003 and 4.62±0.491 mg/g and the measured PSNAP concentration was 3.45±0.574 mg/g.	i) No significant difference was observed with regard to mortality, deformities, weight, and condition factors of the treated fish with controls.<br>ii) PSNAP alone slightly altered the composition of gut microbiota<br>iii) PSNAP alone alter only one metabolic pathway in males (metabolism of cofactors and vitamins)<br>iv) In males, histological and biochemical investigations indicate that PSNAP either alone or in combinations with SMZ were unable to alter *sod*, *cat* and *gpx* transcripts in intestine<br>v) In females, PSNAP alone did not alter *cat* transcript; however, significant reduction in *cat, sod*, and *cat* transcripts were observed	Zhang et al., (2021)

				when coexposed with SMZ	
Marine medaka (*Oryzias melastigma*)	Plain PS (100 nm) and Sulfamethazine (SMZ)	Adults (580.2±189.5 mg body weight) exposed as parents (F0) and evaluated in F1 generation.	Dietary exposure of adults (parents) with PS (3.45 mg/g) and SMZ (4.62 mg/g) for 30 days, either alone or in combination and the offsprings were evaluated after two months of hatching (F1) without any further exposure.	i) Significant decrease in body weights of F1 males and females generated from the parents fed with PS alone (compared to controls) were observed<br>ii) The F1 males and females generated from PS+SMZ fed parents showed significantly higher body weight than the F1 fish generated from the parents (F0) fed with PS alone<br>iii) The F1 offsprings of SMZ, PS, SMZ+PS groups showed significant reduction in body weights than controls (F1).<br>iv) No significant difference was observed in the condition factor [(W/L^3) X100] among four groups (controls, PS, SMZ, SMZ+PS) in F1 females<br>v) In males the condition factor in PS, SMZ, and PS+SMZ groups decreased significantly than controls.	He et al., (2022)

				vi) The gut microbiota in F1 offsprings altered in the SMZ, PS, and PS+SMZ groups<br>vii) No significant difference was observed in the expression of *igf1* gene in the liver of F1 females among all four groups<br>viii) In F1 males (F0 fed with PS), the expression of *igf1* in liver showed significant reduction than controls.<br>ix) Compared to the PS groups, the expression of *igf1* gene in liver of binary exposure (PS+SNZ) showed significantly higher level of expression.<br>x) The expression of *sod* and *cat* genes in intestine of females (F1) of the SMZ+PS group was significantly higher than controls, SMZ and PS groups; expression of *gpx* remained unaltered<br>xi) In males, *cat* and *gpx* expression in intestine remained at the same level among four groups;	

				while *sod* was elevated in PS groups than control and SMZ+PS groups.	
Marine medaka (*Oryzias melastigma*)	PS-NH_2 (80 nm), PS-COOH (80 nm)	Embryos	Embryos were exposed to either PS-NH_2 (10 µg/L) or PS-COOH (10 µg/L) in regular sea water (pH 8.2) or acidified sea water (pH 7.4) for 10 days and depurated until additional 10 days in regular sea water until hatching.	i) No significant difference was observed in embryo mortalities between the embryos exposed to regular sea water (no PS; pH 8.2) and acidified sea water (no PS, pH 7.4)<br>ii) Under normal conditions (regular sea water, pH 8.2), the mortalities and hatching of the embryos exposed to PS-NH_2 or PS-COOH did not show any significant difference with controls.<br>iii) In acidified conditions (pH 7.4), the mortalities of the embryos exposed to PS-NH_2 or PS-COOH increased significantly, and the hatching rate was significantly lower than the embryos exposed as controls.<br>iv) Embryos exposed to PS-NH_2 or PS-COOH required longer hatching time that the embryos exposed as controls (no PS).	Chen et al., (2023a)

				v) The hatching time of the embryos decreased significantly in embryos exposed to PS-COOH than the embryos exposed to PS-NH_2 in acidified sea water.<br>vi) The heart rates were significantly higher in the embryos exposed to PS-NH_2 or PS-COOH either in regular sea water or acidified sea water than the corresponding controls (only in 6-7 days of development)<br>vii) Morphological abnormalities (craniofacial deformities, yolk sac edema, fin rot, spinal deformity, pericardial edema, and cardiac stretch) were significantly higher in PS-NH_2 and PS-COOH exposed groups (both regular and acidified sea water) than the control embryos (no PS).<br>viii) Teratogenic effects of PS-COOH was significantly higher than the PS-NH_2 in acidified sea water.	

				ix) The malformations (cardiac stretch, spinal deformities, pericardial edema) in the embryos exposed to PS-COOH in acidified sea water, was significantly higher than all other treatment groups including controls.<br>x) The swimming velocity and distance in larvae exposed to PS-NH_2, PS-COOH, and acidified sea water (no PS) was significantly lower than those in the controls (regular sea water, no PS).<br>xi) The Ca^{2+} in embryos exposed to PS-NH_2 or PS-COOH in regular sea water or acidified sea water was significantly higher than the embryos exposed to regular sea water or acidified sea water (no PS).<br>xii) The integrated biomarker response (mortality, hatching period, swimming velocity and distance, and malformation) analysis (IBR) indicated that	

				embryos exposed to PS-NH_2 and PS-COOH in acidified sea water showed significantly higher index than the embryos exposed as controls or in regular sea water with PS-NH_2 or PS-COOH.<br>xiii) The quantity of membrane bound PS-NH_2 on the surface of the embryos (chorion consisting of pores with 5µm diameter) was significantly higher (due to high affinity and positive charge) than the membrane bound PS-COOH over time in both regular and acidified conditions. Moreover, in acidified conditions, more PS-NH_2 and PS-COOH are bound to the surface and internalized than the embryos in regular sea water.<br>xiv) Internalization of PS-NH_2 was higher than PS-COOH in regular conditions; however,	

				opposite in acidified conditions.<br>xv) $PS-NH_2$ and PS-COOH was distributed on the digestive tract and intestinal villi of the larvae under normal and acidified conditions.	
Marine medaka (*Oryzias melastigma*)	PSNAP (100 nm) and sulfamethoxazole (SMX)	Juveniles (2 months old) were exposed to PSNAP (1 mg/L), SMX (100µg/L) or both for 30 days.	Juveniles (2 months old) were exposed to PSNAP (1 mg/L), SMX (100µg/L) or both for 30 days.	i) No obvious change in the histological structure of intestine of the three exposed groups compared to control.<br>ii) Volume of intestinal mucus tended to increase in PSNAP groups compared with controls.<br>iii) Goblet cell numbers declined in all three treatment groups.<br>iv) Modulation of intestinal microbial community in all three exposed groups	Li et al., (2023b)
Marine medaka (*Oryzias melastigma*)	PSNAP (100 nm) and SMZ	Adults (four months old) fed with PSNAP and SMZ for 30 days; depurated for 21 days)	i) 0.5 mg SMZ/g food (low SMZ, L-SMZ)<br>ii) 5 mg SMZ/g food (high	i) No significant effects were observed on the growth of the fish.<br>ii) After 30 days of exposure with PS, compared to controls, the gut microbial community in male fish significantly decreased (Shannon index) and no	Wang et al., (2023a)

			SMZ, H-SMZ)<br>iii) 5 mg PS/g food (PS)<br>iv) 5 mg PS+ 5 mg SMZ/g food (PS+HSMZ)<br>v) Control (fed with normal diet)	alterations in females; However, after 21 days depuration the gut microbial community in females showed significant reduction and in males remained at the same levels as in controls.<br>iii) Fish exposed to H-SMZ reduced microbial community significantly than controls in both sexes, however, depuration for 21 days recovered microbial community significantly in females, and in males.<br>iv) Coexposure of PS+HSMZ for 30 days did not significantly alter gut microbial community in both sexes; however, significant recovery of the gut microbial community was observed in females, not in males.	
Marine medaka (*Oryzias melastigma*)	PSNAP (50 nm) BPA	6 hpf embryos Exposed for 21 days	55 µg/L PSNAP and 100 µg/L BPA; exposed either alone or in combination	i) Accumulation of PSNAPs were observed mainly in the abdominal area of the larvae; accumulation of PSNAPs decreased in presence of BPA.	Yu et al., (2023)

				ii) PSNAPs (55 µg/L) reduced heart rates (6 dpf), increased embryonic mortality, and reduced the body length of larvae (21 dpf); however, no effect was observed on hatching rate or hatching time.<br>iii) BPA (100 µg/L) exposure reduced heart rates (6 dpf) and hatching time compared to controls.<br>iv) Upon coexposure, no significant difference was observed in heart rates, embryo mortality, hatching time and rates, and body length of the larvae (21 dpf).<br>v) Developmental deformities (hemorrhaging, craniofacial abnormalities, stretched heart, spinal curvature, and fin deformities) were observed in larvae (21 dpf) after exposure to PSNAP; however, BPA alone or coexposed with PSNAP did not induce any morphological	

				deformities in terms of deformity index.<br>vi) Liver histopathology indicate inflammatory responses (vacuolation, apoptosis, and necrosis) after single exposure to PSNAPs, however, BPA alone induced only vacuolation; coexposure did not induce significant alteration in the histopathological condition index in liver<br>vii) Exposure to PSNAPs induced thinner myocardial wall, reduced myocardial fiber and irregularity in cardiac morphology; BPA alone induced severe degree of irregularity in heart morphology, however, coexposure (PASNAP+BPA) did not significantly alter the histopathological condition index of the heart.	
Marine medaka (*Oryzias melastigma*)	PSNAP (70 nm, 500 nm), PSMIP (2µm)	3 dph larvae fed with PS-exposed	i) 20, 200, and 2000 µg/L (70nm,	i) Accumulation of NAPs are higher than MIPs in the intestine	Li et al., (2024b)

		rotifers for 90 days	500 nm PSNAP and 2 µm PSMIP) were fed to rotifers and used for trophic transfer through rotifer feeding for 90 days (3 dph-93 dph) to marine medaka	ii) Length, weight, and condition factor did not change after trophic exposure of NAPs or MIPs for 90 days.<br>iii) HSI in male and female fish significantly increased in fish fed with 70 nm NAPs by trophic exposure<br>iv) Concentration-dependent decrease in the GSI of both male and female fish<br>v) Structural damage, including hepatocyte vacuolation and hyaline degeneration, and lipid accumulation occurs in marine medaka fish liver exposed to PSNPAs by tropic transfer<br>vi) Hepatic protein, sugar, glycogen, and lactate content were reduced, and triglycerides (TG) content were increased in a concentration-dependent manner in fish exposed to PSNAPs by trophic transfer<br>vii) The fiber density and diameter in muscle were significantly decreased by	

				PSNAP in a concentration-dependent manner; however, TG and lactate content in muscle significantly increased and the total sugar and glycogen content reduced significantly in fish exposed to PSNAP by trophic transfer.<br>viii) PSNAP exposure by trophic transfer disrupted the intestinal histology and microbial community in fish<br>ix) The expressions of *il6, il8, il1b, il10* and *tnf* genes were upregulated by PSNAPs (trophic transfer) in the intestine in a nonlinear fashion.<br>x) The expression of inflammatory factor-related genes (*il6, il8, il1b,* and *tnf*), lipid synthesis-related genes (*fasn, srebf1*, and *pparg*), and lipid transport-related genes (*cetp*,and *ldlr*), were upregulated and the expression of lipid degradation-related genes (*atg1, ppara*, and *aco*)	

				were downregulated in the liver of fish exposed to PSNAPs in a nonlinear fashion.<br>xi) Genes of the Toll-like receptor 4 (TLR4) pathways (*irf3, irak4, traf6,* and *tbk1*) in liver showed a trend of upregulation, while in muscle development-related genes (*myog, myod, mstn, myf5,* and *fgf6b*) were downregulated after PSNAP exposure by trophic transfer.<br>xii) Trophic exposure to PSNAPs (200 and 2000 µg/L) induced structural damage of the testis and ovary and inhibited the maturation processes (increased spermatogonium and reduced sperms; increased perinuclear oocytes and reduced mature follicles).<br>xiii) Fecundity was reduced significantly by trophic exposure of PSNAPs, however there was no	

				alteration in fertilization rate and hatching.<br>xiv) Hatching delay of the embryos were concentration-dependent to the PSNAP exposure.<br>xv) Larval growth was significantly reduced on PSNAP exposed fish on 7dph of development; heart rates and the expression of cardiac development-related genes (*bmp4, nkx2.5, cox, epo,* and *smyd1*) genes were significantly reduced during embryo-larval development.	
Marine medaka (*Oryzias melastigma*)	Plain PS (z-average 244±11.6 nm), PS-COOH (z-average 294.7±8.6 nm), PS-NH2 (z-average 277±15.9 nm); mixed with sulfamethazine (SMZ); SMZ+PS, SMZ+PS-COOH, SMZ+NH_2.	Adults (10-12 months old)	3.65 mg/g NAPs were mixed with SMZ (4.62 mg/g) and fed the fish for 30 days (F0-E); depurated for 21 days and sacrificed (F0-D); some of the embryos were collected at the end of the experiment and cultured for 60 days (F1)	i) The gut microbial community did not differ among three experimental groups (PS+SMZ, PS-COOH+SMZ, PS-NH_2+SMZ) during F0-E and F1 fish.<br>ii) During depuration, a recovery of the bacterial community was observed only in PS+SMZ groups	Zhang et al., (2024b)

Rainbow trout (*Oncorhynchus mykiss*)	Palladium doped polystyrene nanoplastics (Ps-Pd NP) (~200 nm)	Juvenile rainbow trout (body mass 5-10 g)	10 mg PS-Pd NP/kg food; fish fed 2 % of body weight for a period of 7 days; harvested day 3 and 7 exposure period; rest depurated for additional 7 days.	i) Pd (NPs) was detected in the intestine, liver, kidney, gills, and carcass (bone, muscle, and sinew) not in the gall bladder after 3 and 7 days feeding [intestine> kidney >gills >carcass> liver]<br>ii) After depuration for 7 days, no Pd was observed in any of the organs of the exposed fish.	Clark et al., (2023a)
Rainbow trout (*Oncorhynchus mykiss*)	PS (35 ±8 nm);	Juvenile rainbow trout (body weight 5-10 g)	fed the fish (5.9 µg/g food); feeding amount 2% of body weight; exposed for 3, 7 and 14 days	i) Accumulation of PSNAPs were observed in hind intestine after 3 days exposure and transported to liver on day 7.	Clark et al., (2023b)
Tilapia (*Oreochromis niloticus*)	Polypropylene (PPP) PPPMIP (100 µm) PPPNAP (100 nm)	Juveniles (Body weight 10 ±1 g; length 13±1 cm)	Exposed to PPMIP (1, 10, and 100 mg/L) or PPMAP (1, 10, 100 mg/L) for 21 days	i) No effect on body weight and HSI of the fish<br>ii) Significant effects on glycerophospholipid metabolism, arginine, and proline metabolism, and aminoacyl-tRNA biosynthesis	Wu et al., (2023)
Tilapia (*Oreochromis niloticus*)	PSMIP (20 µm); PSNAPs (80 nm)	Larvae (3.5-4 cm total length)	Exposed with 100 µg/L MIP (22,727 particles/L) or NAP ($3.55X10^{11}$ particles/L) for 28 days.	i) No effect on the total length and weight of the fish.<br>ii) Accumulation of both MIPs and NAPs occurred in gills; the accumulation of MIPs was ~ 2.6 X	Zheng and Wang (2024)

				higher than NAPs, even though the number of NAP particles were higher than MIPs.<br>iii) Differential damage was observed in gill tissue after PSMIP (mitochondrial swelling and cristae fragmentation) and PSNAP (chromatin marginalization and apoptosis) exposure.<br>iv) Significant aneurysms were observed in PSMIP-exposed fish, not in PSNAP-exposed fish<br>v) 12 cell populations were identified in gills; endothelial cells (ENDCs), fibroblasts (FIBs), ionocytes (H^{+}ATPase -rich cells and Na^{+}/K^{+}-ATPase rich cells), immune cells (T-cells); macrophages (MAPs), B cells, natural killer cells (NKC); pavement cells (PVCs), neurons, neuroepithelial cells, and mucus cells; PSMIPs increased the total cell numbers of 12 types of gill cells (8%	

				increase), but decreased by 22.8% after exposure to PSNAPs.<br>vi) For PSMIP-treated groups, six cell types (ENDCs, PVCs, Na^+/K^+-ATPase cells, T-cells, neurons, and neuroepithelial cells) exhibited a significant increase in quantity, whereas five cell types (FIBs, H^+ATPase rich cells, macrophages, NKCs, and B-cells) were inhibited significantly by PSNAPs.<br>vii) For PSNAP exposed fish, significant reduction in cell number (EDCs, FIBs, macrophages, NKCs and B cells); only H^+ATPase rich cells showed significant increase.<br>viii) The MIP responsive DIGs in FIBs are *colla1, colla2, col6a1, coll3a1, eif 2a, dcn, pdgfra, dlx3, copx2*, and *deptor.*<br>ix) MIP exposure primarily downregulated the activity of proton transmembrane	

				transporter in FIB, whereas NAPs suppressed their vacuolar transport and carbohydrate derivative metabolic process<br>ii) Cell-cell communication between fibroblasts, and H^+-ATPase rich cells, neurons, macrophages, neuroepithelial cells and Na^+/K^+-ATPase rich cells in gills were significantly inhibited by MIP exposure; however, NAP exposure did not show any significant change in cell-cell communication in gills.	
Tilapia (*Oreochromis niloticus*)	PSMIP (2 and 20 µm); PSNAPs (80 nm)	Larvae (3.5-4 cm total length)	100 µg/L for 28 days	i) The accumulation of PSMIPs in gills were significantly higher than the PSNAPs (size-dependent accumulation)<br>ii) The oxygen consumption rates (OCR) was significantly higher in PSMIP exposed fish than PSNAP fish.<br>iii) Epithelial lifting, cell swelling, and increased mucus production were found in PSMIPs	Zheng et al., (2024)

				(more sever in 20 µm)<br>iv) Fusion of gill lamellae, development of aneurysm was also observed in fish exposed to 20 µm PSMIPs.<br>v) Apoptosis and cell necrosis were also detected in gills exposed to 20 µm PSMIPs.<br>vi) The number of up- and downregulated genes were much higher in PSMIPs (20 µm: up 2110 and down1989; 2 µm: up 3080, down 3040) than PSNAPs (up226, down 379).<br>vii) Upregulation of *egln3* (egl-9 family hypoxia-inducible factor 3) and *nadk* (nicotinamide adenine dinucleotide kinase a) genes were upregulated by PSMIPs, while *cftr* (cystic fibrosis transmembrane conductance regulator) gene is downregulated in gills of fish exposed to 20 µm PSMIP.<br>viii) Activation of inflammatory response in	

				gills after exposure to PSMIPs, however, NOD-like receptor signaling pathways were highly enriched by PSNAP, indicating differential inflammatory responses induced by MIPs and NAPs.<br>ix) Metabolomics analysis indicated significant downregulation of ADP (adenosine diphosphate) by both PSMIPs and PSNAP, isocitrate and oxidative stress (GSH/GSSG) by PSMIPs, and upregulation of phenyl pyruvic acid (PPA) by PSMIPs only.	
Tilapia (*Oreochromis mossambicus*)	PS (100 nm)	4 weeks old larvae (0.57 ±0.13 g body weight)	Exposed to 20 mg/L ($1.3X10^5$ particles/mL) for 7 days with or without 7 days depuration	i) 203 metabolites were significantly altered<br>ii) The genes downregulated after 7 days of exposure belonged to cell adhesion molecules (*cam, ncam2, cntn2,* and *nlg1*), and neuroactive ligand receptor activation (*grin2a, grin2b, gabrb2* and *gabra2*).	Pang et al., (2021)

				iii) The genes affected during recovery are belonged to ECM-receptor interaction (*cd36, lamc2, itgb4, lama1, itga10, col1a1, and col1a2*) and the metabolic processes of carbohydrate (*pck1, pmm1, gldc,pfk1*), energy (*soux,papss1, ca14, sqor, ca6*), lipid (*srd5a2,ptgdsl2, pla2g7, hsd17b10*) and amino acid (*cad, odc1, smox, ahcyl2*)<br>iv) 4 genes decreased during exposure and recovered to normal levels during depuration period (*ncam2, p2rx3, gad1* and *gad2*)<br>v) 2 genes (*col1a1* and *col1a2*) maintained their expression during exposure and downregulated during depuration period<br>4 genes (*ptgdsl2, pla2g7, cad*, and *odc1*) maintained their expression during exposure and upregulated during recovery.	

Nile Tilapia (*Oreochromis niloticus*)	PS (86 and 185 nm).	Juveniles (10.9 ±3.9 g body weight; 8.8±1.0 cm body length)	Exposed to PSNAPs (1 mg/L, waterborne) for 21 days and depurated for 7 days (total duration was 28 days).	i) No significant difference between the body length and weight of the fish during the experiment<br>ii) Both respiration and ingestions are the main pathways for PSNAPs accumulation<br>iii) PSNAPs were accumulated in the gill, stomach, intestine, liver, and muscle.<br>iv) Accumulation of PSNAPs in the gills and liver were associated with the NAP mass concentration in the aqueous phase rather than size, while accumulation in the intestine, liver, and muscle were size-dependent (smaller sizes have greater accumulation than larger sizes).<br>v) Maximum accumulation was reached on day 14 of exposure.<br>vi) Elimination of PSNAPs from the tissues was also size- and organ-dependent; smaller particles eliminate faster than the larger particles	Hao et al., (2023)

				vii) Complete elimination of the 86 nm particles in the intestine, stomach, and gills, however, retained in liver (17.3%) and muscle (7.79%) after 7 days depuration.<br>viii) Complete elimination of 186 nm particles was not observed in all five tissues during depuration.<br>ix) PSNAPs passed through intestinal wall and delivered to other tissues.<br>x) Mechanical damage was observed in the intestinal wall by PSNAPs (thinner mucosal layer, disordered epithelial cells, submucosal cell edema and eosinophilic infiltration) which is size-dependent (smaller the particle more sever the damage).<br>xi) The diamine oxidase activity and d-lactate content of the intestinal wall increased after PSNAP exposure.<br>xii) Upregulation of *tnfα, il1β*, and *il8* and downregulation of *il10*	

				genes by PSNAPs occurred in the intestine.<br>xiii) The SOD, GPx activities and the MDA content in the gut increased by PSNAPs.<br>xiv) Intestinal microbiota disrupted by PSNAP exposure.	
Nile Tilapia (*Oreochromis niloticus*)	PS<br>PSMIP (500 and 5000 nm sizes)<br>PSNAP (100 nm size)	Juvenile<br>Body weight 15±5 g	Exposed to 1, 10, and 100 µg/L for 7 days	i) Little effect on feeding and swimming behavior.<br>ii) PS was accumulated in gill, liver, intestine, and muscle tissues; accumulation of PSNAP was higher than PSMIPs in gill and liver.<br>iii) PSNAPs not the PSMIPs induced hepatic steatosis in a concentration-dependent manner.<br>iv) PSMIP (500 nm) resulted in mild local inflammatory infiltration in the hepatic lobule and increased the expression of proinflammatory cytokines.<br>v) Significant upregulation of *tnfα* and *il1b* was observed in fish exposed to PSNAP not in PSMIPs; *cyp1a* and *cyp3a* were	Wang et al., (2023b)

				downregulated by PSNAP and PSMIP (only in 500 nm particle size)<br>vi) PSNAP upregulated 113 genes and downregulated 128 genes (total 241 genes) in the liver of tilapia<br>vii) Downregulation of calreticulin (*calr)*, and glucose-regulated protein (*hspa5)* genes by PSNAP was observed in the liver of tilapia<br>viii) Concentration-dependent upregulation of eukaryotic translation initiation factor 2a (*eif2a*), and activating transcription factor 4a (*atf4a*), and C/EBP homologous protein (*chop*) genes occurred by PSNAP exposure<br>ix) Concentration-dependent upregulations of nuclear factor erythroid 2-related factor (*nrf2*) and klech-like ECH-associated protein 1 (*keap1*) were observed in the liver of tilapia exposed to PSNAPs.	

				x) Hepatic GSH content remained unaltered in both PSMIP and PSNAPs in tilapia; however, concentration-dependent decrease in the activities of SOD with concomitant increase in MDA content was observed in fish exposed to PSNAP	
Red Tilapia (*Oreochromis niloticus*)	PSNAP (100 nm)	Juvenile; Body weight 21±3.9 g; length 9.5±1.7 cm	Exposed to 1, 10 and 100 µg/L for 14 days waterborne	i) No mortality or abnormality (deformity and ulceration)<br>ii) Accumulated in the gut, gills, liver, and brain in a concentration-dependent manner<br>iii) Accumulation was tissue specific; gut and gills accumulated more PSNAPs than liver and brain<br>iv) AChE activities in brain reduced by PSNAP<br>v) In liver the EROD (cyp1a) and BFCOD (cyp3a) were altered in a nonlinear fashion<br>vi) SOD activity induced, while MDA content remained unaltered.	Ding et al., (2018)

Red Tilapia (*Oreochromis niloticus*)	PSMIP (300, 5000, and 70000-90000 nm sizes)	Juveniles; body weight 27.7±4.2 g and length 11.4±1.1 cm	Exposed to 100 µg/L for 6 and 14 days	i) Accumulated in gut, gills, liver, and brain tissues with highest accumulation was in the gut<br>ii) SOD activity in liver increased significantly in fish exposed to PSMIPs 14 days<br>iii) The MDA content showed size and concentration-dependent change in the liver of fish after 14 days exposure (decreased in fish exposed to 300 nm size, however, increased in 5000 and 70,000 - 90,000 nm sizes)<br>iv) The EROD (cyp1a) and BECOD (cyp3a) activities altered inconsistently between early (6 days) and late (14 days) exposure periods of PSMIP.<br>v) The brain AChE activity after 14 days exposure decreased significantly than controls in all PSMIP exposed fish<br>vi) A size-dependent change in metabolome profile of liver exposed to different PSMIPs.	Ding et al., (2020)

				vii) Influenced the pathways of tyrosine metabolism by PSMIPS and PSNPL in tilapia liver.	
Zebrafish (embryos)	Polyamide (~ 32.50 µm)	Embryos (2 hpf)	1, 10, and 20 mg/L (10 dpf waterborne)	i) No significant effects on hatching<br>ii) No malformation and mortality of the zebrafish larvae<br>iii) The body weight of the larvae decreased by 12.8% of the controls<br>iv) Ingested polyamide is mainly distributed in the intestinal tract of the larvae.<br>v) The level of TNF-α was significantly lower<br>vi) Damaged intestinal enterocytes with vacuolar appearance in the intestinal mucosa<br>vii) The level of ROS is significantly higher (146.7%) than controls with altered GSH content and SOD activity.<br>viii) Disorders in lipid metabolism<br>ix) Downregulated the pathways related to digestion and absorption,	Zhang et al., (2022c)

				pancreatic secretion, cholesterol metabolism, and steroid biosynthesis.<br>x) Downregulated the expression of *cel.1* and *cel2*genes	
Zebrafish (*Danio rerio*)	LDPE (164, 106, 342, 122, 91 nm)	Embryos (4 hpf)	0.001, 0.01, 0.1, 1, 10 mg/L (96 h waterborne)	i) No effect on hatching or malformation of the embryos<br>ii) No significant effects was observed on heart rates<br>iii) Locomotor activity insignificantly modified in dark and light phases.	Tamayo-Belda et al., (2023)
Zebrafish (*Danio rerio*)	PE (50 nm)	Embryos (6hpf)	3X10^{10} particles/L (24 h waterborne)	i) Delayed hatching of the embryos<br>ii) Reduced larval body length<br>iii) No effect on larval morphology (cardiac edema, axial curvature, head deformities)	Monikh et al., (2022)
Zebrafish	PE (hydrodynamic size 191.10 ±3.13 nm)	Embryos (6 hpf)	25, 50, 100, 200, 400, 600, 800, 1000 µg/mL for 48-96 h	i) Pericardial edema and yolk sac degeneration observed in PE exposed larvae in a concentration-dependent manner<br>ii) NOAEL is 50 µg/L<br>iii) No effect on heart rates<br>iv) Inhibit angiogenesis in a concentration-dependent manner (100-200 µg/L)<br>v) Inhibits cardiac output and blood in a	Sun et al., (2021)

				concentration-dependent manner<br>vi) Induce ROS in a concentration-dependent manner<br>vii) Concentration-dependent induction of systematic inflammation (accumulation of erythrocytes in tail veins).	
Zebrafish	Polyethylene (76.74±14.07 μm) (polythene) (pristine)	Adults (8–10-month-old; length 3.5±2 cm)	Waterborne 24 h exposure	i) No mortality occurred<br>ii) The accumulated pristine PE was broken down into microplastic particles (5.92±4.96 μm) as detected in the fecal matter (approximately 70% size reduction)	Khan and Ali (2023)
Zebrafish	Polyethylene MIP (13.5 μm) and NAP (70 nm)	Adults (AB strain and 3 months old) 3-5 cm body length and 0.4-0.6 g weight	Exposed to PEMIP (20 mg/L) and PENAP (20 mg/L) and a combination of PEMIP+PENAP for 21 days.	i) GST activity in gills decreased by PENAP, not by PEMIP or in combinations of PENAP and PEMIP<br>ii) GSH content and SOD activity in gills remained unaltered<br>iii) CAT activity was increased in gills exposed to both PEMIP and PENAP<br>iv) LPO levels increased in gills by PEMIP and PENAP after 14 days	Li et al., (2023c)

				exposure not after 21 days exposure<br>v) GST activity in intestine was significantly low after 7 days, while enhanced by PENAP after 14- and 21-days exposures<br>vi) GSH content was enhanced in gut by PEMIP after 7- and 14-days exposure<br>vii) CAT activity in gut remained unaltered.<br>viii) LPO levels in gut increased in PEMIP and PEMIP+PENAP fish on 14 days exposure; however, remained unaltered in PRNAP fish; in 21 days, LPO levels significantly decreased in all exposed fish (PEMIP, PENAP, PEMIP+PENAP) compared with controls.<br>ix) Alteration in the SOD activity in gut of PEMIP, PENAP and PEMIP+ PENAP exposed fish remained inconsistent.<br>x) In liver, GST activity increased in all exposed groups, however, GSH	

				remained unaltered in PEMIP+PENAP fish.<br>xi) CAT activity in liver significantly increased in PEMIP exposed fish in 7 and 14 days; SOD activity and LPO level responded inconsistently in plastic-exposed fish compared to controls.<br>xii) The AChE activity in gill significantly decreased in fish exposed to PEMIP on 7- and 14-days and inconsistently altered in PENAP and PEMIP+PENAP fish.<br>xiii) In gut, the AChE activity is altered inconsistently, while in liver significantly reduce in fish exposed to PENAP and PEMIP+PENAP only on 7 day.<br>xiv) The protobacteria population (intestinal dysbiosis) increased and tenericutes decreased in the gut of fish by PEMIP, PENAP, and PEMIP+PENAP groups.	

Zebrafish	Polyethylene terephthalate (PET) (hydrodynamic diameter 70±5 nm)	Embryos	6 hpf and 72 hpf embryo were exposed to 5, 10, 50, 100, 200 mg/L until 96- 120 hpf of development	i) No effects on heart rates, however, survivability and the hatching of the embryos reduced in a concentration-dependent manner<br>ii) Reduced locomotor activity in the dark phase (embryos exposed to higher concentration of PETNPs)<br>iii) Significant alteration of metabolites related to targeting the liver and pathways associated with detoxification and oxidative stress<br>iv) Impairment of mitochondrial membrane integrity as reflected by elevated levels of polar head group phospholipids<br>i) Cellular bioenergetics as evidenced by changes in numerous metabolites associated with interrelated pathways of energy metabolism.	Bashirova et al., (2023)
Zebrafish	Polyethylene terephthalate (PET) nanoplastic (68.06-955 nm); PET	Embryos	0.5, 1, 5, 10, and 20 mg/L; embryos exposed for 6 days	i) No effects on mortality and hatching<br>ii) Diminish spontaneous tail coiling,	De Souza Teodoro et al., (2024)

	microplastic (1305-2032 µm)			iii) Elevated heart rates in a concentration-dependent manner<br>iv) Accumulated on the chorion surface in a concentration-dependent manner<br>v) Reduced interocular distance without affecting the body length.<br>vi) No significant effect on locomotor activity<br>vii) No significant change was observed in lipid peroxidation levels and total antioxidant capacity.	
Zebrafish	Poly lactic acid (PLA) (122, 255, 615, 615, 712 nm)	Embryos (4hpf)	0.001, 0.01,0.1, 1, 10 mg/L (96h)	i) No effects on mortality, malformation, and hatching<br>ii) Heart rates significantly decreased in a nonmonotonic manner.<br>iii) Locomotor activity strongly modified in light phase than dark phase in a concentration-dependent manner	Tamayo-Belda et al., (2023)
Zebrafish	Polymethylmethacrylate (PMMA) (32 nm)	embryos	0.001, 0.01,0.1, 1, 10, 100 mg/L 96 hpf waterborne	i) concentration- dependent mortality<br>ii) delayed hatching<br>iii) Pericardial edema (concentration-dependent)<br>iv) No significant effects on swimming behavior; however, total distance	Manuel et al., (2022)

				swam during light and dark phases increased significantly than controls<br>v) AChE activity did not show any significant change except in larvae exposed 0.01 mg/l where the activity tended to decrease<br>vi) Nonlinear increase of GPx activity<br>vii) No effect of GST<br>viii) CAT activity tended to increase in lower concentrations.<br>viii) LPO content increased in lower concentrations (0.001-0.1 mg/L)	
Zebrafish	PPP (562.15±118.47 nm)	Embryos (24 hpf and 72 hpf)	50 mg/L for 24 h	i) Uptake by ingestion and Accumulated in the intestine<br>ii) No significant difference was observed in the mortality and deformities of the embryos (pericardial edema, yolk edema, yolk necrosis, curved tail, fin deformities, and head malformation)	Lee et al., (2022)
Zebrafish	PPP (50 nm)	Embryos (6hpf)	$3X10^{10}$ particles/L (24 h waterborne)	i) Delayed hatching of the embryos	Monikh et al., (2022)

				ii) Reduced larval body length<br>iii) Curved spine	
Zebrafish	PPP (164, 255, 459, 531, 220 nm)	Embryos (4 hpf	0.001, 0.01, 0.1, 1 and 10 mg/L	i) No effects on mortality, malformation, and hatching<br>ii) Heart rates decreased in a concentration-dependent manner<br>iii) Locomotor activity nonmonotonically elicited in light phase while decreased in dark phase	Tamayo-Belda et al., (2023)
Zebrafish (*Danio rerio*)	PSNAP (47 nm) and PSMIP (41µm); coexposure 17α-ethinylestradiol (EE2) (2 and 20 µg/L)	Embryos;	1 mg/L (120 h waterborne)	i) Both PSMIP and PSNAP reduced the accumulation of EE2 in the embryos<br>ii) No effect of PSMIP and PSNAP was observed on the survivability and malformation rate of the embryos<br>iii) PSNAP alone and coexposure (EE2) suppressed locomotor activity (total distance travelled) during dark phase, while PSMIP did not<br>iv) PSNAP alone or coexposure with E2, reduced body length<br>i) Upregulation of *gfap* and *α1-tubulin* mRNAs	Chen et al., (2017a)

					(related to nervous system) by PSNAP alone or coexposed with E2 occurred (PSMIP has no effect)<br>ii) Genes related to visual system (rhodopsin, *zfrho;* blue opsin, *zfblue*) were not significantly changed by PSNAP exposure; however, PSMIP upregulated *zfrho* only<br>iii) No effect was observed on CAT and GPx activity by both PSNAP and PSMIP; however, GSH content decreased significantly in both PSNAP and PSMIP exposures.<br>iv) Decreased AChE activity by PSNAP alone not by PSMIP	
Zebrafish (*Danio rerio*)	PS (25, 50, 250, 700 nm)	Embryos; three time points (0-48 hpf; 24-72 hpf; 72-120 hpf	5-50 mg/L (48 h waterborne). Three time points (0-48 hpf;25 mg/L) (24-72 hpf; 50 mg/L) 72-	i) PS adsorbed in the chorion (fertilized eggs, immediately after fertilization)<br>ii) PS adsorbed on the epidermis after 24 hpf<br>iii) After 72 hpf, accumulation occur on		Van Pomeren et al., (2017)

			120 hpf (5 mg/L)	eyes (25-50 nm), GI tract and gills (250-700 nm)	
Zebrafish (*Danio rerio*)	PS (51 nm)	Embryos (6hpf)	0.1, 1, 10 mg/L (120h); depuration 120 hpf-168 hpf	i) PS accumulated in the yolk sac and migrated into other organs (GI tract, gall bladder, liver, pancreas, heart, and brain).<br>ii) accumulation decreased during depuration in all organs<br><br>iii) Did not induce mortality, deformities, or mitochondrial bioenergetics<br>iv) Heartbeats decreased<br>v) Behavior altered (hypoactive swimming)	Pitt et al., (2018a)
Zebrafish (*Danio rerio*)	PS (25 nm). coexposure with glucose (40 mM)	larvae (72 hpf)	20 mg/L (72 hpf were exposed until 120hpf.)	i) Absorption was dependent on PS size and time of exposure<br>ii) PS was accumulated in intestine, exocrine pancreas, and gall bladder.<br>iii) Cortisol concentration of the whole larvae increased after PS exposure, while no effect was seen when co-exposed with glucose.<br>iv) Locomotor activity was enhanced by PS in dark	Brun et al., (2019)
Zebrafish (*Danio rerio*)	PS (50, 200, and 500 nm);	Embryos;	0.1 mg/L (6, 24 and 96 h immersion)	v) Smaller PS readily penetrated the chorion	Lee et al., (2019)

	coexposure chloroauric acid (Au ions) (1 µg/mL)			and accumulated throughout the whole body<br>vi) PS induced only marginal effects on hatching rates, developmental abnormalities, and cell death<br>vii) Chloroauric acid (Au ions) synergistically exacerbated the effects in a concentration and size-dependent manner	
Zebrafish (embryos)	PS (500 nm)	Embryos	Embryos (72 hpf) were exposed to 1 mg/L (for 48h; 72-120 hpf)	i) PSNAP accumulated in the gut and gill and also in the neuromast (a mechanosensory organ belongs to lateral line sense organ)<br>ii) The activity of P-glycoprotein (a membrane protein) remained unaltered after PSNAP exposure<br>iii) No significant effect on ROS level CAT, GPx, and GST activity, while a significant induction of SOD activity of the larvae exposed to PSNAP was observed.<br>iv) Significant decrease in COX activity was	Parenti et al., (2019)

				observed in larvae exposed to PSNAP during development<br>v) No effect was observed in the locomotor activity (total distance travelled), however, compared with the controls, a significant increase in the absolute turn angle of the treated larvae was observed throughout the first light period.	
Zebrafish (*Danio rerio*)	PS (44 nm) Coexposure: polycyclic aromatic hydrocarbons (PAH)	Embryos	0.1, 1, 10 mg/L PS (96 h waterborne) alone and coexposed with river sediment extract which contain PAH (5.07-25.36 µg/L)	i) PS did not exhibit developmental defects<br>ii) PS decreased the developmental abnormalities and impaired vascular development caused by PAH<br>iii) PS decreased the mitochondrial coupling efficiency and increased NADH production<br>iv) PS decreased the sorbing of the PAH	Trevisan et al., (2019)
Zebrafish (*Danio rerio*)	PS (50 and 200 nm)	Embryos (6-120 hpf)	10, 100, 1000 and 10,000 µg/L	i) Developmental abnormalities induced by PS (50 and 200 nm) is not significantly different from the controls. However, the rate of	Pedersen et al., (2020)

				swim bladder uninflation was concentration dependent (7% increase only in 200 nm groups, exposed to 100 and 10,000 μg/L), but not significant.<br>ii) No effects on mortality and hatching rates.<br>iii) 50 nm PS has no effect on swimming behavior; however, 200 nm PS induced hyperactivity during dark cycle (total distance travelled) in a concentration-dependent manner (1000 and 10000 μg/L)<br>iv) Concentration and size-dependent accumulation of PS observed in GI tract, eye, liver, and cranial region<br>v) Transcriptomic analysis suggests neurodegeneration and motor dysfunction induced by PS exposure.	
Zebrafish (embryos)	PS (20 nm)	embryos	Microinjected PSNAP (~270 mg/L; injected volume 3 nL)	i) The survival rate significantly decreased after PSNAP injection.<br>ii) Hatching rates were slightly reduced in	Sokmen et al., (2020)

			to the zebrafish embryos and evaluated after 120 hpf of injection	PSNAP-exposed embryos at 72 hpf not at 48 hpf.<br>iii) Malformation of the embryos (pericardial edema, yolk sac edema, short tail, and malformed head) induced by PSNAP administrations.<br>iv) ROS induction enhanced in the head region by PSNAP<br>v) Apoptosis was induced after cellular intake of PSNAP<br>vi) PSNAP induced DNA damage in the brain of zebrafish.	
Zebrafish (*Danio rerio*)	PS (44 nm) Coexposure: polycyclic aromatic hydrocarbons (PAH) (1mg/L)	Embryos	1 mg/L for 7 days	i) Unable to exhibit developmental disorders (PSNAPs alone or in coexposure)<br>ii) PSNAPs accumulated in the yolk sac and brain; PAH alone was accumulated in yolk sac, however, coexposure showed accumulation in the brain too.<br>PSNAP decreased NADH production.	Trevisan et al., (2020)
Zebrafish	PS (70 ± 9.21 nm)	Embryos	i) Embryos (<1 hpf) were	i) Accumulation of PSNAP occurred by both exposure roots and was	Zhang et al., (2020)

			injected 0.52 nL volume of 1000, 3000, and 5000 mg/L PSNAP and after hatching reared in PSNAP-free water until 4 weeks.<br>ii) Embryos (1hpf) were exposed to 0.5 and 5 mg/L PSNAP waterborne until hatching and then reared until 4 weeks in PSNAP-free solution.	maximum in the yolk sac and also found in other organs like brain, eyes, gut, and swim bladder.<br>ii) Mortality of the embryos tended to increase in embryos exposed to PSNAP waterborne in a concentration-dependent manner (not significant), however, no effect was observed in embryos exposed to PSNAP by injections.<br>iii) No change in hatching rates<br>iv) Larval length significantly reduced in fish exposed to PSNAP waterborne or injection in a nonlinear fashion (observed on week 4 of exposure)<br>v) Developmental abnormalities (tail flexure, jaw abnormalities, and pericardial edema) was observed in larvae (96 hpf) exposed to PSNAP waterborne in a concentration-dependent manner.	

				vi) Locomotor activity was not affected in larvae exposed to PSNAP by injection, while in waterborne exposure significantly reduced counterclockwise and anticlockwise rotations only. Other behavior (meander, angular velocity, and moving distance) remained unaltered.<br>vii) The expression of *sod*1 and *sod2* did not change in injected fish, while *sod2* was significantly downregulated in fish exposed to PSNAP waterborne.<br>viii) Expression of *mbp* (responsible for myelination of axons) and *syn2α* (a neuronal phosphoprotein, induced synaptogenesis) was downregulated only in injected groups and *gfap* (an intermediate filament protein, expressed in astrocytes) was downregulated in only in	

				waterborne exposed groups<br>ix) Expression of visual system cone genes (*opn1sw2, opn1lw2* and *opn1mw*1) were downregulated by injection of PSNAP to the embryos, however waterborne exposure downregulated *opn1w2* and *opn1mw1* only.	
Zebrafish (*Danio rerio*)	Polystyrene (PS) (60 nm); coexposure simvastatin (SIM)	Embryos;	1. PS (0.015, 1.5 and150 mg/L)<br>2. SIM (0.015-150 µg/L)<br>3. PS (0.05 or 1.5 mg/L) +SIM (12.5 or 15 µg/L) (96 h immersio n)	i) PS did not exert any significant effects<br>ii) SIM (12.5µg/L) delayed hatching, decreased heartbeats, induced edema, and mortality<br>iii) Coexposure of PS (0.015 mg/L) and SIM (12.5 or 15 µg/L) showed increase in hatching and heartbeats	Barreto et al., (2021)
Zebrafish (embryos)	PS (100 nm) coexposed with Butyl Methoxy dibenzoyl methane (BMDBM)	Embryos	iii) Embryos were exposed for 2-12 h with PSNAP (10 µg/L) and BMDMB (1, 10,	i) PSNAP decreased the adsorption of BMBBM on zebrafish embryos.<br>ii) BMDBM exposure alone increased the expression of CAT, SOD, GPx, and GST- related genes.<br>iii) PSNAP alone upregulated SOD, GPx, and GST genes.	Liu et al., (2021)

			100 µg/L) either alone or in combination. Locomotor activity and development was evaluated at 120 hpf	iv) Combined exposure caused lower levels of oxidative stress than individual exposures.<br>v) BMDBM exposure alone significantly downregulated the expression of *dnmt1* and *dnmt3aa*, while PSNAP exposure alone significantly decreased the expressions of *dnmt3bb1* and dnmt*3bb2*<br>vi) Coexposure of BMDBM and PSNAP downregulated the expression of *dnmt1* and *dnmt3aa*, while downregulation of *dnmt3bb2* was interrupted as well as no effect was observed in the expression of *dnmt3bb1*.<br>vii) BMDBM exposure alone significantly downregulated the expression of *cyp19a1a* and *cyp19a1b* in a concentration-dependent manner while PSNAP exposure alone or in combination did not affect the expression of these	

				genes (*cyp19a1a* and *cyp19a1b*)<br>viii) There are 7-types of cells were found in zebrafish embryos (neural anterior cells, neural crest cells, neural mid cells, neural posterior cells, endoderm cells, epidermal cells, and mesoderm cells)<br>ix) Among the 7 cell types, mesoderm cell populations were found to be highest (35-47%) and the DEG was also highest in these cells. After exposure, all three treatment types reduced DEG in these cells. Moreover, the neural mid cells were also affected by the exposures.<br>x) BMDBM mainly affected the differentiation and fate of neurons in the CNS through the regulation of *her5, her6, her11, ifng, pax2a*, and *fgfr4*.<br>xi) PSNAP regulated the expression of *olig2, foxg1a, fzd8b, six3a, rx1, lhx2b, nkx2.1a,* and *sfr5*	

				to alter nervous system development, retinal development, and stem cell differentiation.<br>x) At 120 hpf of development it was observed that BMDBM and PSNAP either alone or in combination has no effect on survivability. However, the heart rates increased, and the larval swimming was significantly decreased.	
Zebrafish (*Danio rerio*)	PS (50 nm) Co-exposed with nAL_2O_3 and $nCeO_3$	Embryos	PSNAP=1 mg/L nAL_2O_3=1 mg/L $nCeO_3$=1 mg/L exposed for 96 hpf	i) PSNAP enhanced the accumulation of Al and Ce<br>ii) No effects on embryo mortality or malformation rates (pericardial edema, yolk sac edema, tail, and spinal curvature)<br>iii) Hatching rate was declined in embryos co-exposed with $nCeO_2$.<br>iv) PSNAP interfere with the efflux transporter activity resulting increased accumulation of metal ions (Al or Ce)<br>v) PSNAP alone or in combination enhanced ROS.	Bhagat et al., (2022)

				vi) SOD activity significantly decreased in fish exposed to PSNAPs, Al_2O_3, CeO_2 alone or in combination<br>vii) CAT activity significantly increased in fish exposed to PSNAP but decreased in fish exposed to Al_2O_3 alone. Combined exposure showed enhancement in CAT activity compared to controls. CAT activity remained unaltered in fish exposed to CeO_2 alone or in combinations.<br>viii) GPx activity remained unaltered in fish exposed either to PSNAP or Al_2O_3 alone; coexposure significantly decreased GPx activity. GPx was induced in fish exposed to CeO2 alone, however, significantly reduced in fish coexposed with PSNAP.<br>ix) GSH content remained unaltered in fish exposed to PSNAP, Al_2O_3, CeO_2 alone. Coexposure with Al_2O_3 enhanced GSH	

				content, however, with CeO_2 remained unaltered.<br>x) GR content showed reduction in fish exposed to all treatment groups, while MDA remained unaltered in fish exposed to PSNAP, AL_2O_3, CeO_2 alone. While combination with Al_2O_3 or CeO_2 showed significant reduction.<br>xi) The integrated biomarker response (IBR) was calculated based on seven oxidative stress-associated biochemical markers (SOD, CAT, GPx, GSH, GR, MDA, and ROS). It was observed that IBRv2 values showed an increase after PSNAP exposure. In combined exposures, AL_2O_3 showed increase, while CeO_2 showed decline<br>xii) There was no change in metallothionine (MT) (*mt2*) expression by PSNAP alone. Exposure with Al_2O_3 and CeO_2 alone enhanced *mt2*	

					expression, however, coexposure with PSNAP significantly decreased the expression of *mt2* compared to the expression made by AL_2O_3 and CeO_2 alone.<br>xiii) The expression of *abcc2* and *P-gp* mRNAs were upregulated and *abcc1, abcc4*, and *abcb4* mRNAs were downregulated (efflux transporter genes) by PSNAP exposure.<br>xiv) Al2O3 alone downregulated the expression of all efflux transporter genes except abbcc2, while CeO2 alone downregulated the expression of *abcc1, abcc4, abcb4,* and *p-gp.*<br>xv) Coexposure with Al_2O_3 (increase in *abcc4*) and CeO_2 (reduced *abcc1* and *p-gp*) modulated the expression patterns of efflux transporter genes regulated by PSNAP<br>xvi) The expression of *gadd45a, p53, xrcc2, rad51*, and *trl3* expression	

				remained unaltered in fish exposed to PSNAP alone<br>xvii) Al_2O_3 upregulated the expression of *gadd45a* and *xrcc2* and coexposure with PSNAP enhanced the expression of *rad51* and *p53*; coexposure with CeO_2 downregulated *tlr3* and *mt2* genes.	
Zebrafish (*Danio rerio*)	PS (100 nm); Co-exposure BDE-47 (10 ng/L)	Embryo-larval	2.5 and 25 µg/L) (0-7 dpf) Waterborne	i) Accumulated in the anterior part containing the yolk sac and digestive tract<br>ii) No effect on body length<br>iii) Food consumption increased in both PS and PS+BDE-47 groups<br>iv) Significant decrease in neutral lipid storage in a concentration-dependent manner both in PS and PS+ BDE-47 groups<br>v) Increase in oxygen concentration rates in both PS and PS+BDE-47 groups.<br>vi) PS exposure elicited complex effects on locomotor behavior with increased long distance and decreased short distance movement	Chackal et al., (2022)

				xii) Gene expression analysis pointed to a negative interaction while the BDE-47- induced gene expression was abolished by coexposure with PS.	
Zebrafish (*Danio rerio*)	PS (50 and 100 nm) and micro-PS	Embryo larval development [transgenic larvae were also used]	0.1, 0.5, 2 and 10 mg/L; waterborne exposure 120 hpf	i) PS detected in the intestine and areas of excretion (when they hatched)<br>ii) Neutrophil population increased in the abdomen of the larvae<br>iii) Macrophage population decreased in the abdomen of the larvae.<br>iv) Increased expression of liver-specific fatty acid binding protein 10a (*fabb10a*)<br>v) ROS generation was induced by PS exposure<br>vi) 51-59 differentially expressed metabolites were identified in the larvae of which 80-90% were upregulated. Among them, metabolites of citric acid cycle and amino acid biosynthesis cycle proteins were upregulated	Cheng et al., (2022)

Zebrafish (*Danio rerio*)	PS (100 nm)	Embryos	100, 200, and 400 mg/L (96 h waterborne)	i) Decreased hatching and survival rates<br>ii) 96 h LC_{50} of the 24 hpf embryos was 431.1 mg/L<br>iii) Inhibits heart rate and reduced body length and suppressed behavioral activity<br>iv) Induced activation of oxidative stress including reactive oxygen species<br>v) Increased SOD and CAT activities<br>vii) mRNA analysis indicate that the mRNAs related to base excision pathways (*lig1, lig3, polb, parp1, pold, fen1, nthl1, apex, xrcc1,* and *ogg1*) were altered	Feng et al., (2022)

Zebrafish (*Danio rerio*)	PS (50 nm) Coexposure with phenanthrene (PHE) and mucin (jelly fish)	Embryos	PSNAP=5 mg/L; PHE=0.1, 0.5 and 1.0 mg/L; mucin=50 µg/mL [4,8,12,24,32,48, 72 hpf]	i) Hatching rates significantly reduced by PSNP and PHE (concentration-dependent) alone<br>ii) Pericardial edema and yolk sac edema were observed in larvae exposed to PSNAP and PHE alone.<br>iii) PSNAP was agglomerated on the surface of the chorion of the embryos exposed to PSNAP, and PSNP+PHE however, clean chorion was observed in embryos exposed to PSNP+ mucin; PSNP+PHE+mucin<br>iv) 246, 104, and 550 DEGs were observed in embryos exposed to PSNP, PHE, and PSNP+PHE groups.<br>v) PSNP (5 mg/L) increased the expression of CAT and p53, while decreased the expression of bcl2.	Geum and Yeo, (2022)
Zebrafish	PS (25 nm)	embryos	Embryos were exposed to PSNAP (10, 25, and 50 mg/L until 96 hpf.	i) Concentration-dependent decrease in embryo survivability by PSNAP<br>ii) Concentration-dependent increase in hatching of the	Kantha et al., (2022)

				embryos (48 hpf) by PSNAP<br>iii) Concentration-dependent decline in the whole-body contents of Na^+, K^+, and Ca^{2+} of the embryos exposed to PSNAP.<br>iv) Concentration-dependent decline in H^+ and NH_4^+ secretion of the skin of the embryos exposed to PSNAP<br>v) The total length of microridges on the skin keratinocytes of the embryos significantly reduced by PSNAP exposure<br>vi) Concentration-dependent decline in the HR (H^+ -ATPase) and NaR (Na^+ K^+ -ATPase) cell (ionocytes) densities in the yolk sac skin of the embryos exposed to PSNAP<br>vii) Concentration-dependent decline in the active ionocytes of the embryos exposed to PSNAP<br>viii) Concentration-dependent increase in ROS in both ionocytes and non-	

				ionocytes in embryos exposed to PSNAP<br>ix) Concentration-dependent downregulation of *CAT*, *GPx1a*, *sod1* and *sod2* mRNAs occurred in embryos exposed to PSNAP.<br>x) The mRNA expression of *casp3a* (apoptosis marker) was upregulated, while *bcl2* (anti-apoptosis marker) was downregulated in embryos exposed to PSNAP.	
Zebrafish (embryos)	PS (100 nm)	Embryos (2 hpf)	Embryos (2hpf) exposed to PSNAP (10 µg/L) and avobenzone (AVO; 10 µg/L) either alone or in combinations for 144 hpf and recovered for 3 days (without any treatment).	i) PSNAP promoted the accumulation of AVO in zebrafish embryos.<br>ii) AVO alone or in coexposure with PSNAP did not affect the survivability or induced any morphological abnormalities of the larvae.<br>iii) The expressions of *α1-tubulin, elav13, gap43, gfap, mbp* and *syn2a* were upregulated and *lfing* expression was downregulated at 12 hpf by AVO alone or	Liu et al., (2022b)

				coexposure. However, at 144 hpf*, α1-tubulin, elavl3, gap43*, and *mbp* did not show any significant alteration and after recovery no alteration was seen in the expression of all these genes which suggests that these genes are suspectable to AVO during early phase of development.<br>iv) The f*oxg1* related to stem cell expression was upregulated in AVO fish while downregulated in fish exposed to PSNAP alone or in combinations. Other stem cell -related genes like *her5, her6, shha*, and *sox2* were altered significantly in all three exposure groups. However, after recovery, no significant difference was observed in the expression of *foxg1, her6, shha* and *sox 2* between control and the exposure groups (AVO, PSNAP, and AVO+PSNAP).	

				v) The genes related to retinal system development were affected by PSNAP alone or in coexposure. The expressions of *pax2, pax6*, and *six3* were upregulated, while *lax9* was downregulated.<br>vi) The antioxidant enzyme activities (CAT and SOD) were enhanced significantly after 144 hpf in fish exposed to AVO, PSNAP and in combined exposure groups. After recovery (72 h without treatment) the CAT activities in all three treatments returned to normal level, while SOD activity in all three exposure groups still remained higher<br>vii) The AChE activity was significantly increased in all three exposure groups than controls at 144 hpf. After recovery, the enzyme activity went back to control levels.<br>viii) The locomotor behavior (swimming speed) of the	

				larvae (144 hpf) significantly reduced in all three exposed groups and the reduction was more pronounced in fish exposed to PSNAP and in combination. After recovery, the swimming speed tended to went back to the control level, however, remained significantly lower in treated groups	
Zebrafish (*Danio rerio*)	PS (22 nm)	embryos	0.001, 0.01, 0.1, 1, 10, 100 mg/L (until 96 hpf; waterborne)	i) Mortality was concentration-dependent<br>ii) Hatching delayed in a concentration-dependent manner<br>iii) Did not induce any morphological abnormalities (pericardial edema, tail deformities)<br>iv) No significant alteration occurred in swimming behavior; however, total distance travelled during light phase, showed an increasing tendency.<br>v) The activity of AChE decreased in lower concentrations (0.01-0.1 mg/L), however,	Manuel et al., (2022)

				increased in higher concentration (1 mg/L)<br>vi) The enzyme activities related to oxidative stress (GST, GPx, CAT), showed a decreasing tendency, although nonlinear.<br>vii) LPO levels decreased in 0.1 mg/L and increased in 1 mg/L.<br>viii) Glycogen concentrations increased in a concentration-dependent manner	
Zebrafish (*Danio rerio*)	PS (micro- and nano) (micro=4.5 µm; nano=50 and 500 nm);<br>Also, co-exposed with B(a)P and B(a)P alone.	embryos	50nm (0.000069, 0.00069, 0.069,0.687, 6.87 mg/L).<br>500 nm (0.00034, 0.00069, 0.069, 0.687, 6.87mg/L).<br>4.5 µm (0.0251, 0.0501, 0.501, 5.01, 50.1 mg/L)<br>B(a)P (0.1, 0.5, 1, 5, 10 mg/L)<br>for 120 hpf	i) Survivability of the embryos remained unaffected either by PSNAP or PSMIP alone.<br>ii) Embryos exposed to 50.1 mg/L and 4.5 µm MIP-B(a)P caused a significant increase in malformed embryos (120 hpf)<br>iii) B(a)P alone induced concentration-dependent malformation in embryos at 120 hpf<br>iv) The EC50 values estimated for 4.5 µm PSMIPs-B(a)P were 45.57 ±9.12 mg/l and for	Martinez-Alvarez et al., (2022)

				B(a)P alone was 3.55±0.68 mg/L<br>v) PSNAP and PSMIP are distributed in the chorion, eye, tail, and yolk sac of the embryos in a size-, concentration-, and developmental stage-dependent manner.	
Zebrafish (*Danio rerio*)	PS (200 nm and 600 nm). Coexposure with PS 200 nm PS+ B(a)P (10 µg/L)	Embryos (6hpf)	$3X10^{10}$ particles/L (waterborne 24 h exposure)	i) No embryo mortality<br>ii) Hatching delayed in embryos exposed to 200 nm and 600 nm PSMIPs alone.<br>iii) Coexposure with PSMIP (200 nm) ameliorated the hatching delay induced by B(a)P.<br>iv) No morphological disorders observed in larvae, however, the length of the larvae reduced.	Monikh et al., (2022)
Zebrafish	PSMIP (1000 nm) and PSNAP (400 nm)	Embryos	Embryos (1 dpf) were exposed to MIP1 ($1.09X\ 10^9$ particles/L=60 mg/L), MIP2 ($8.19X10^8$ particles/L= 45 mg/L), MIP3 ($5.46X10^8$ particles/L= 30	i) Concentration-dependent increase in embryo mortality was observed in both PSMIPs and PSNAPs treatment; PSNAP was more toxic than PSMIP.<br>ii) Exposure of the embryos to PSMIP (30 mg/L) and PSNAP (30 mg/L)	Park and Kim (2022)

			mg/L) MIP4 ($2.73X10^8$ particles/L= 15 mg/L), and NAP1 ($8.53X10^9$ particles/L=30 mg/L) NAP2 ($6.39X10^9$ particles/L= 22.5 mg/L), NAP3 ($4.26X10^9$ particles/L=15 mg/L), NAP4 ($2.13X10^9$ particles/L=7.5 mg/L) in suspension for 1-4 days.	induced tail malformation (reduced tail length) and vasculatures.<br>iii) Blood flow resistance of the caudal artery increased as the embryos exposed to PSMIP (30 mg/L) and PSNAP (30 mg/L); however, remained unaltered in dorsal artery	
Zebrafish (*Danio rerio*)	PS (44 nm) Co exposure with phenmedipham (PHN)	Embryos	PS (0.015, 0.15, 1.5, 15, and 150 mg/L). PHN (0.02, 0.2, and 20 mg/L) Coexposure [0.015 mg/L PS+ 2 mg/L PHN; 0.015 mg/L PS+ 20 mg/L PHE; 1.5 mg/L PS+ 2 mg/L PHN; 1.5 mg/L PS+ 20 mg/L PHN); exposed for 96-120 hpf	i) During 96 hpf, PS and PHN either exposed alone or combined did not affect embryo development<br>ii) At 120 hpf, PS induced hyperactivity and PHN induced hypoactivity; in combination (0.015 mg/L PS+ 20 mg/L PHN) hyperactivity seen with inhibition of cholinesterase activity.<br>iii) At 96 hpf, PS increased CAT, while PHN increased GST, and combination (1.5 mg PS+	Santos et al., (2022)

				20 mg/L PHN) increased both CAT and GST	
Zebrafish (embryos)	PS (20 nm)	embryos	4 hpf embryos were injected with PSNAP (~270 mg/L; 3 nL injected volume/egg) and grown in plastic-free media for six months. Then they breed and the offspring were evaluated for toxicity	i) The malformation rate observed in offspring (F1) is lower than the rate observed in PSNAP-injected larvae (P1).<br>ii) The mortality rate in the parent larvae was higher than the mortality rate observed in F1 offspring<br>iii) The survival rate in the F1 offspring was higher than the PSNAP-injected parents<br>iv) No difference was observed in hatching rates between injected P1 and F1 offspring<br>v) Compared with F1 controls, significant reduction in eye size, body length, and swimming behavior (total distance covered) was observed in F1 offspring exposed to PSNAP in P1 generation.<br>vi) Compared with F1 controls, heart rates of PSNAP offspring (F1) was found to be significantly higher	Sulukan et al., (2022a)

				vii) Compared with F1 controls, cellular apoptosis, and ROS content was increased and lipid accumulation was decreased in F1 offspring exposed to PSNAP during parental generation.<br>viii) Pathway analysis indicate that tyrosine, unsaturated fatty acid metabolism, folate biosynthesis, arginine-proline metabolism were affected by PSNAP exposure	
Zebrafish	PS-NH_2 (50 nm fluorescent)<br>PS-COOH (30 nm fluorescent)<br>PS-NH_2 (51 nm, unlabeled) (+ve charge)<br>PS-COOH (50 nm unlabeled) (-ve charge)	Embryos	Exposed 30 and 50 mg/L to labelled or unlabeled PS-NH_2 or PS-COOH for120h.	i) Both positively charged PS (PS-NH_2) and negatively charged PS (PS-COOH) was accumulated in GI tract, pericardium, and brain<br>ii) Positively charged PSNAP (PS-NH_2) induced stronger developmental toxicity (decreased spontaneous movement, heart beats, hatching rates and larval length) than negatively charged PS (PS-COOH)<br>iii) Positively charged PSNAP (PS-NH_2) Induced	Teng et al., (2022a)

				stronger apoptosis in the brain cells and greater neurobehavioral impairment<br>iv) Positively charged (PS-NH_2) decreased levels of glycine, cystine, glutathione, and glutamic acids,<br>v) The negatively charged PS (PS-COOH) increased the levels of spermine, spermidine, and tyramine. Positively charged PS (PS-NH_2) interacted with neurotransmitter receptor N-methyl-D-aspartate receptor 2B (NMDA2B), whereas negatively charged PS-NP impacted the G-protein-coupled receptor 1 (GPR1), that led to the behavioral difference.	
Zebrafish (embryos)	PS (80 nm) Coexposure BDE-47	Embryos	PSNAP (50 µg/L, 100 µg/L, 1 mg/L, 5 mg/L, 10 mg/L) exposed either alone or in combination with BDE-47 (0.1 mg/L) until 120 hpf.	i) PSNAP accumulated in the surface of the chorion in a concentration-dependent manner, began 12 hpf.<br>ii) Concentration-dependent accumulation of the PSNAP occurred in the brain, gills, mouth, trunk,	Wang et al, (2022)

				heart, liver, and digestive tract of the larvae.<br>iii) Single exposure to PSNAP, BDE-47 and PSNAP+BDE-47 for 120 hpf resulted malformations (hemorrhage, small head and eyes, tail deformity, yolk edema, pericardial edema, spine curvature, swim bladder deficiency) during embryo-larval development of zebrafish (12-120 hpf) which is concentration-dependent, higher in combined exposure compared with single exposure groups.<br>iv) The survivability of the embryos showed no significant difference with controls in single exposure groups (either PSNAP alone or BDE-47 alone); however, in general, coexposure enhanced mortality in a time and concentration-dependent manner.<br>v) Length of the larvae (120 hpf) reduced in PSNAP (single exposure) and	

				PSNAP+BDE-47 groups in a nonlinear fashion.<br>vi) PSNAP has no effect on the heart rates of the embryos at 96 hpf, however, BDE-47 alone and in combination with PSNAP significantly decreased hear rates in a nonlinear fashion.<br>vii) The spontaneous movements of the embryos at 12 hpf was significantly reduced by PSNAP in a concentration-dependent manner; however, BDE-47 significantly enhanced spontaneous movements. In coexposure experiments, spontaneous movements were lower than the BDE-47 (single exposure) group, however, higher than the embryos exposed to PSNAP alone.<br>viii) No significant effect was observed in the hatching of the embryos (60 hpf) exposed to PSNAP, BDE-47 either alone or in combinations.	

				ix) Histopathological changes were observed in eyes, muscle, and cartilage tissues of larvae (120 hpf). Coexposure to PSNAP and BDE-47 induced greater damages to the retinal structures in the eyes, muscle fiber and cartilage tissue of the larvae than those with single exposure<br>x) 7 days recovery reduced PSNAP accumulation in GI tract, head, gall bladder, liver, and heart<br>xi) The transcription of adrenocorticotrophic releasing hormone (CRH) gene decreased in larvae exposed to BDE-47 and also in coexposure groups.<br>xii) Among the genes of the HPT-axis, *tshβ* expression was significantly upregulated by PSNAP alone in a concentration-dependent manner, however significantly reduced in coexposure groups compared with PSNAP alone (10 mg/L)	

				xiii) The expression of sodium (Na)-iodide symporter (NIS) gene was significantly upregulated by PSNAP alone in a concentration-dependent manner; coexposure showed a reducing tendency (not significantly different)<br>xiv) Thyroglobulin (TG) gene expression was significantly upregulated in PSNAP and BDE-47 either alone or in coexposure in a concentration-dependent manner.<br>xv) The expression of thyroxine-transport protein gene (TTR) showed a decreasing tendency in larvae exposed to PSNAP and BDE-47 either alone or in combination showed a decreasing tendency compared with controls<br>xvi) The expression *dio2* showed a decreasing tendency in larvae exposed to PSNAP (not significant) compared	

				with controls. BDE-47 alone was able to upregulate *dio2* expression (not significant). Coexposure reduced the expression of *dio2* in zebrafish larvae.<br>xvii) The expression of *trα* remained unaltered in all treatment groups compared with controls, however, expression of *trβ* upregulated by BDE-47 and PSNAP exposure alone, while in coexposure showed a tendency to reduce the expression compared with BDE-47 alone.<br>xviii) The expression of *esr2* tended to increase by PSNAP exposure alone (not significant); however, coexposure with BDE-47 tended to decrease the expression of *esr2* (not significant). Compared with controls, *vtg* expression was upregulated in larvae exposed to PSNAP in a concentration-dependent manner. Coexposure	

				reduced the expression of VTG compared with larvae exposed to PSNAP alone.	
Zebrafish (*Danio rerio*)	PS (44 nm). Coexposure: diphenhydramine (DPH) (0.01 and 10 mg/L)	Embryos	PS (0.015 and 1.5 mg/L) (96 -120 h waterborne)	i) After 96 h, coexposure induced mortality, malformation, and decreased heartrates, and hatching<br>ii) After 120 h, coexposure decreased swimming activity<br>iii) After 96 h, glutathione S-transferase and cholinesterase activity increased in coexposure, while catalase remained unaltered.	Barreto et al., (2023)
Zebrafish (*Danio rerio*)	PS pristine (80 nm); aged UV-PS; non- aged O3-PS; penicillin alone and co-exposure	Embryos	Pristine PS, UV-PS, and O3-PS (0.5 and 5 mg/L); penicillin (1 and 10 µg/L); zebrafish embryos (8 hpf-120 hpf) were exposed to PS and penicillin alone or in combinations	i) PS was accumulated in the yolk sac, eye, head, and nerve tubes; accumulation was interrupted in coexposure experiments<br>ii) PS and penicillin alone did not induce developmental toxicity (hatching, malformation, and mortality); however, coexposure affected the motor behaviors (spontaneous movements,	Chen et al., (2023b)

				touch response, swimming) and heart beats during development.<br>iii) The motor behaviors decreased in embryos exposed to UV-PS not with O3-PS groups; coexposure with penicillin only touch response decreased significantly compared to the larvae exposed only to penicillin.<br>iv) After one week growth in a treatment-free medium (12 dpf), the percent time in the light area was significantly decreased in PS, O3-PS, PS+ penicillin, UV-PS+ penicillin<br>v) After 13 dpf, the mirror attack was significantly increased in PS, PS+ penicillin, and O3-PS+penicillin groups<br>vi) Cellular apoptosis was induced in 24 hpf and 120 hpf during embryo-larval development of zebrafish exposed in all experimental groups (mostly in embryonic tail	

				and larval head region) except those exposed in O3-PS<br>vii) ROS levels were significantly increased in PS+ penicillin and UV-PS+ penicillin groups Upon PS, aged PS, or penicillin co-exposed PS neurotransmitter metabolites in zebrafish larvae were significantly dysregulated	
Zebrafish	PS (50 nm) and Sodium nitroprusside (SNP)	Embryos	PSNAP (0.1, 1, 5, 10, 20, 30, and 50 mg/L) exposed for 5 days, evaluated on, 5th, 7th, and 12 th day. Sodium nitroprusside (0.1,1, 10, 20, 30 and 40 µM); cultured alone with sodium nitroprusside and co-exposed with PSNAP; final concentration selected; PSNAP 20 mg/L, and SNP 8µM up to 12 days	i) Accumulation of PSNAP in zebrafish larvae was significantly reduced by coexposure with SNP.<br>ii) Larvae developed developmental abnormalities (deformities, spinal curvature, organ edema, survival rates) after PSNAP exposure in a concentration-dependent manner.<br>iii) Coexposure with sodium nitroprusside (SNP) alleviate the toxic effects of PSNAP in a concentration-dependent manner	Chen et al., (2023c)

				iv) PSNAP (20 mg/L) significantly increased NO content while co-exposed with SNP (8 μM) did not potentiate the effect.<br>v) PSNAP (20 mg/L) significantly decreased the activities of soluble guanylate cyclase (sGC) and protein kinase G (PKG) enzymes, however, coexposure with SNP diminished the effects of PSNAP on enzyme activities<br>vi) The expression of *Adma, Nos,* and *Pde6d* was significantly higher in PSNAP groups than control or larvae coexposed with SNP; however, the expression of *prkg* was significantly reduced in PSNAP groups than control and SNP coexposed groups.<br>vii) PSNAP exposure enhanced ROS levels in the larvae and coexposure with SNP did not aggravate the ROS content.	

				viii) The metabolic level of the liver was significantly increased in larvae by PSNAP and SNP coexposure alleviated the process<br>ix) The oxidative stress index (based on CAT, peroxidase, and SOD activities and GSH and MDA contents) significantly increased while SNP coexposure alleviated the process.<br>x) PSNAP exposure caused significant apoptosis in larvae, while SNP coexposure significantly alleviated the process.<br>xi) PSNAP exposure caused significant mitochondrial depolarization in zebrafish larvae, which was alleviated by SNP treatment.<br>xii) The activity of the caspase-3 and the expression of *bik, bad, bax, bim, bid*, and *bok* were significantly increased by PSNAP exposure, while	

				coexposure with SNP alleviated the process.<br>xiii) PSNAP exposure induced ferroptosis (cell death due to iron accumulation) while coexposure with SNP alleviated the process.<br>xiv) The expression of GPX4, the key protein for ferroptosis, and the genes *Slc7a11, Acs14a, Keap1b*, and *Ncoa4* were higher in larvae exposed to PSNAP, while coexposure with SNP alleviated the process.<br>xv) PSNAP exposure significantly increased the proliferation of macrophages and neutrophils; coexposure with SNP alleviated the process.<br>xvi) The expression of *tnfα, tgfβ, il-4, il-6* were upregulated by PSNAP while coexposure with SNP alleviated the process.	
Zebrafish	PS (20 nm)	Embryos (wild type and	2 hpf embryos exposed to PSNAP	i) Embryos developed pericardial edema, and curved spine after PSNAP	Dai et al., (2023)

		transgenic; *tg(flk1: eGFP)*	(2, 5, and 8 mg/L) for 22, 46, and 70 h	exposure in a concentration-dependent manner<br>ii) The survivability of the embryos and hatching was reduced in embryos exposed to PSNAP<br>iii) The body length of the larvae was also reduced by PSNAP exposure.<br>iv) The heart rates of the embryos after 46 h increased in a concentration-dependent manner<br>v) Malformations in sprouting of intersegmental vessels (ISV) occurred by PSNAPs in a concentration-dependent manner (24 hpf).<br>vi) Disruption in sprouting of small vessels (nasal vessels, dorsal vessels, and ventral vessels) induced by PSNAPs in a concentration-dependent manner (48 hpf)<br>vii) PSNAPs induced overgrowth of the common cardinal vein (CCV) and endothelial	

				cells in CCV in zebrafish embryos in a concentration-dependent manner (48 hpf)<br>viii) PSNAPs promotes vasculogenesis (increasing the number and length of extrinsic branches of the sub-intestinal venous plexus) in a concentration-dependent manner (72 hpf)<br>ix) The PSNAP exposure disrupted the expression of VEGFA/VEGFR pathway-related genes (*vegfa, nrp1, klf6a, flt1, fih1, fik1, cldn5a*, and *rspo3*) in a time and concentration-dependent manner.	
Zebrafish	PS (50 nm)	Embryos	0.1, 0.5, and 1 mg/L for 4-72 hpf; experimental temperatures are 24-, 27-, and 30 º C.	i) The accumulation of PSNAP at 24 hpf is temperature dependent; higher accumulation was observed in embryos exposed to 30 º C than exposed at 27 ^{0} C; however, at 24ºC accumulation was less than the embryos exposed at 27º C<br>ii) The heart beats of the embryos significantly increased in 24 hpf embryos exposed to PSNAP at 30 ^{0}C than at 27 ^{0}C. However, a	Duan et al., (2023)

				concentration-dependent decrease in heart rates was observed in embryos exposed to 30 ^{0}C at 24 hpf.<br>iii) The pericardial edema, and the mortality of the embryos exposed to PSNAP tended to increase at 72 hpf at 30 ^{0}C.<br>iv) PSNAP inhibited myocardial diastolic function<br>v) PSNAP (0.1 mg/L) at 27 ^{0}C induced 65 differential proteins, of which 31 were upregulated and 34 were downregulated; the differentially expressed proteins participated in metabolic and insulin signaling pathways.<br>vi) Among the differentially expressed proteins, PCK1 and CSTC were downregulated, and ABTA was upregulated<br>vii) PSNAP exposure also induced disorders in amino acid metabolism including valine, leucine, and isoleucine biosynthesis and β-alanine, aspartate, and glutamate metabolism.	

				viii) Downregulation of β-alanine, leucine and valine in the amino acid metabolism and upregulation of glucose-1, glucose-2, and glucose-6-phosphate 2 in the insulin signaling pathways were observed.<br>ix) When embryos were exposed to 0.1 mg/L PSNP and grown at 30 ^{0}C, 454 genes were differentially expressed of which 327 genes were upregulated and 127 genes were down regulated and the proteins belonged to the cardiac muscle contraction, oxidative phosphorylation, glutathione metabolism and metabolic regulation pathways.<br>x) Among the upregulated proteins, six of them (TRDN, TNT, TPM, MYOSIN, ATP, and CYTO) belonged to cardiac muscle contraction pathways; NADH dehydrogenase	

				(NDUFA2 and NDUFA8), cytochrome C reductase, (UOCR 10) cytochrome C oxidase 9COX6C) and ATP synthase (ATPSPD) participated in oxidative phosphorylation complex I, III, IV, and V.<br>xi) The proteins (GSTM2, GSTA1, and GSTT1A) involved in GSH metabolism were upregulated in PSNAP exposed zebrafish grown at 27 ^{0}C<br>xii) Compared to fish grown at 27 ^{0}C, among the differentially expressed proteins (beta-alanine, aspartate, and glutamate metabolism and linoleic acid metabolism) twelve differentially expressed proteins were upregulated (such as beta-alanine-1) and thirty were downregulated (such as linoleic acid) in zebrafish maintained at 30 ^{0}C.	
Zebrafish (*Danio rerio*)	PS (80 nm) Coexposure with Acetaminophen (APAP)	Embryos	5, 10, 25, 50, 100 µg/L PSNAP (waterborne three hpf-96 hpf)	i) PS alone has no significant effect on mortality or hatching and	Gao et al., (2023b)

	(2-8mM)		APAP 2mM, 8mM, PSNAP 100 µg/L+ APAP 2mM, APAP 8mM+PSNAP 100 µg/L (3 hpf-96 hpf)	morphological development is normal. ii) PS was unable to induce pericardial edema, spinal curvature, pigment deficiency, melanocyte abnormalities which are more pronounced with coexposure with APAP iii) Body length tended to reduce with coexposure with APAP iv) PS induced hyperactivity in swimming of the larvae. Coexposure with APAP caused a depression in the total distance, swimming speed, and the maximum acceleration. v) Downregulation of the genes (*runx2a, runx2b, sp7, bmp2b,* and *shh*) related to osteogenesis in PS alone and coexposure groups.	
Zebrafish	PS (30 nm and 100 nm)	Embryos	0.1, 1, and 10 mg/L for 96 h	i) PSNAPs were accumulated in the chorion, head, trunk and in the yolk. ii) The expression of pro-inflammatory cytokine genes, *il6* and *il1β* were	Martin et al., (2023)

				upregulated by 0.1 and 1 mg/L PSNAP (100 nm) in a time and concentration-dependent manner.<br>iii) The expression of ROS removing enzymes CAT and SOD was also elevated by PSNAP (100 nm) by time and concentration-dependent manner<br>iv) The expression of two cytochrome P450 genes (*cyp1a* and *cyp51*) was also upregulated by PSNAP (100 nm) in a time and concentration-dependent manner.<br>vi) In zebrafish embryos, macrophages were found around the eyes and uptake PSNAP.	
Zebrafish (embryos)	PS (30 nm)	Embryos	0.1, 0.5 and 3 mg/L for 120 hpf	i) PSNAP exposure did not affect mortality<br>ii) Down regulation of the expression of stress-response genes, such as heat shock protein 70 (*hsp70*) occurred in a concentration-dependent manner; however, the expression of *hsp27* and *hsp90* remained unaltered.<br>iii) The expression of oxidative stress-response genes *sod1*, and *sod2*	Martin-Folgar et al., (2023)

				upregulated in a concentration-dependent manner; however, *cat* expression remained unaltered.<br>iv) The genes responsible for DNA damage (*gadd45α* and *rad51*) did not alter after PSNAP exposure<br>v) The genes responsible for apoptosis, such as *cas1* and *cas8* were upregulated in a concentration-dependent manner, while *cas3a* remained unaltered in zebrafish embryos exposed to PSNAP.<br>vi) The antiapoptotic gene, *bcl2a*, was downregulated in a concentration-dependent manner by PSNAP<br>vii) The genes related to inflammation such as *il1β* was upregulated by PSNAP in a concentration-dependent manner, while *cox1* remained unresponsive.<br>viii) The expression of antiapoptotic gene *bcl2*	

				was inhibited by PSNAP in a nonlinear fashion.<br>ix) The expression of *AChE* gene was also downregulated in a concentration-dependent manner in embryos exposed to PSNAP during development.<br>The expression *cox1*, the gene responsible for mitochondrial response, remained unaltered in larvae (120 hpf) exposed to PSNAP.	
Zebrafish (embryos)	PS (500 nm)	Embryos	Embryos (3 hpf) exposed to PSMIP (0.1, 1, and 10 mg/L) for six dpf.	i) Accumulation occurred mostly in the intestinal region which is concentration-dependent<br>ii) No morphological changes as well mortality was induced in embryos exposed to PSMIP<br>iii) No effect on hatching and survival of the embryos as well as no significant effect on behavior (spontaneous movement).<br>iv) The swimming activity (swimming speed and total distance travelled) significantly reduced in a nonlinear fashion in	Suman et al., (2023)

				larvae exposed to PSMIP during development<br>v) A concentration-dependent increase in apoptosis was observed in embryos exposed to PSMIP during development<br>vi) SOD and CAT activities significantly reduced, and ROS content significantly increased in embryos exposed to PSMIP during development<br>vii) A significant nonlinear increase in nitrite/nitrate content and decrease in the AChE activity of the embryos exposed to PSMIP during development<br>viii) Compared with controls, the neurotransmitter serotonin and dopamine levels tended to decrease in embryos exposed to PSMIP during development compared with controls<br>ix) Compared with controls, gene expression analysis indicated upregulation of *p53, caspase-3* and	

				caspase-9 genes while downregulation of *bcl-2* and *bdnf* mRNAs in embryos exposed to PSMIP was observed.	
Zebrafish	PS (91, 122,220,712, 825nm)	Embryo (4 hpf)	0.001, 0.01, 0.1, 1, 10, 10 mg/L	i) No effect on mortality, malformation,<br>ii) heart rates showed nonmonotonic increase<br>iii) The locomotor activities was significantly increased during light phase ; however, in dark phase significant increase was observed in lower concentrations (0.001, 0.01, and 1 mg/L) while significant reduction was found at the highest concentrations of PS (10 mg/L)	Tamayo-Belda et al., (2023)
Zebrafish	PS (15 nm) alone and coexposure with *p,p'*-DDE	Embryos (<2 hpf)	50 mg/L PS, 100 µg/L DDE, and PS+DDE for 96 h	i) PS accumulated in GI-tract, pericardium, eye, and cranial regions<br>ii) No significant effect of PS was observed in larval mortality, body length, eye size, swim bladder inflation.<br>iii) DDE alone or in combination with PSNAP induced pericardial	Varshney et al., (2023)

				edema, lordosis, and uninflated swim bladder<br>iv) Heart rates remained identical with controls and PS exposed larvae.<br>v) No significant difference in the oxygen consumption rate of the larvae exposed to PS only, however, in DDE and PS+DDE groups, oxygen consumption rates increased significantly compared to controls<br>vi) Locomotor behavior of the larvae (movement, distance moved, velocity, angular velocity, rotations) did not change after PSNAP exposure, while significant alterations (reductions) were noticed in larvae exposed to DDE alone or DDE+PSNAP<br>vii) Downregulation of eight differentially expressed genes (DEG) in larvae exposed to PS was observed.	
Zebrafish (embryos)	PS (80 nm) (coexposed with BDE-47)	Embryos	Zebrafish embryos exposed to PSNAP (0.05, 0.1, 1, 5, and	i) PSNAP accumulated in gills, GI, liver, and heart of the larvae (120 hpf)	Wang et al., (2023c)

			10 mg/L) and BDE-47 (0.1 and 10 µg/L) alone or in combinations for 120 hpf	ii) No significant effect on mortality was observed in embryos exposed to PSNAP (120 hpf), however, concentration-dependent effect was observed in coexposure groups (120 hpf)<br>iii) The spontaneous movement of the embryos during twelve hpf was significantly decreased in a concentration-dependent manner in embryos exposed to PSNAP, while coexposure also showed a decreasing tendency of spontaneous movement in zebrafish embryos during 12 hpf.<br>iv) The hatching rates (48 hpf) was increased in embryos exposed to PSNAP in a concentration-dependent manner, while coexposure significantly increased hatching rates with lower PSNAP (0.05, 0.1 and 1 mg/L) and decreased hatching rates with higher PSNAP (5 and 10 mg/L)	

					when compared with controls.<br>v) No significant effects on heart rates of the embryos at 96 hpf exposed to PSNAP, while decreased in embryos exposed to BDE-47 alone or in combination with PSNAP.<br>vi) Compared with controls, the liver morphology (color) was altered in PSNAP and BDE-47 exposure either alone or in coexposure. Moreover, the size of the liver markedly reduced in coexposed larvae than the larvae exposed either to PSNAP or BDE-47.<br>vii) Compared with controls, ROS production occurred in eyes, yolk sac, GI tract and tail which was significantly higher in larvae exposed to PSNAP and BDE-47 either alone or in combination. Moreover, coexposure exacerbated ROS production compared with single exposure groups.	

				viii) Expression of *gpx1a* (an antioxidant gene) was downregulated by PSNAP, BDE-47 either alone or in combinations. The expression of *cyp1a1* remained unaltered in larvae exposed to PSNAP and BDE-47 alone, however coexposure upregulated *cyp1a1* expression in a concentration-dependent manner.	
Zebrafish (embryo-larval)	PS-COOH (50 nm)	Larvae	Embryos (4 hpf) were exposed at a concentration of 1, 5, and 10 mg/L until larval stage of development (144 hpf)	i) The distance travelled by the PSNAP-treated larvae was significantly higher than the controls, though the effect was not concentration-dependent<br>ii) Both AChE activity and dopamine content increased significantly in PSNAP-treated larvae and the enhancement of dopamine was concentration-dependent.<br>iii) With regard to alteration in the lysosomal proteins, it was concluded that PSNAP accumulated in the cellular lysosomes and induced oxidative stress.	Wang et al., (2023d)

				iv) Irreversible inhibition of *atoh1a* expression occurred in the cerebellum of zebrafish (transgenic) by PSNAP exposure<br>v) Several proteins related to Parkinson's disease (PARK7, PDX2, and MB) were upregulated and GAPDH-2 was downregulated in zebrafish larvae by PSNAP exposure N-acetyl-aspartic acid and arachidonic acid (neurotoxicity-related metabolites) were increased in larvae exposed to PSNAP	
Zebrafish	PS Fluorescent and nonfluorescent (100, 500, and 1000 nm)	Embryos	2 hpf embryos were exposed to 10 mg/L PS particles ($2.2X10^{12}$ particles /L; $1.76 X10^{10}$ particles/L, $2.2X10^{9}$ particles/L) for 24 hpf-120 hpf	i) Accumulation of PSNAP occurred on the surface of the chorion and the entry of the PSNAP through chorion was size-dependent.<br>ii) Accumulation of PSNAP in the brain of the embryos started from 48 hpf<br>iii) At 120 hpf, accumulation of PSNAP observed in brain, yolk sac, muscle,	Zhou et al., (2023c)

				GI tract, pancreas, gall bladder, liver, and swim bladder<br>iv) The tail retraction frequency (spontaneous movement) at 24hpf, mortality (24 and 48 hpf)72 hpf heartrates, body length at 120 hpf during embryo-larval development of the PSNAP groups did not differ significantly with the controls.<br>v) Size-dependent reduction of the hatching rates of the embryos exposed to PSNAP when compared with the control embryos was at 48 hpf.<br>vi) PSNAP exposure increased deformities (scoliosis, uninflated swim bladder, tail curvature, pericardial edema, and yolk sac edema) was enhanced when compared with the controls which is also dependent on the size of the PSNAP.<br>vii) Compared with controls, the locomotor activity	

				(the mean velocity and distance of movement) of the larvae (120 hpf) decreased/inhibited in larvae exposed to all PSNAP group when compared with controls<br>viii) The development of neurons and motor neurons in the brain of zebrafish (72 hpf) was interrupted by PSNAP when compared with controls (probably reduced the number of neurons); PSNAP exposure exhibited axonal deletion and loss of continuity (100 nm); reduced synaptic density and shorter length (500 nm) and disorganized ventral axons with no regularity (1000 nm).<br>ix) Compared with controls, embryos exposed to PSNAP induced apoptosis in the brain of zebrafish as observed at 72 hpf.<br>x) Among the genes related to the development of central nervous system (*Neurog1, Gfp43, Gfap,*	

					Syn2a, Mbpa, Elavl3,a1b-Tubulin, C-fos, Bdnf, and *Shha*), the expression of *Gfap, Syn2a,Mbpa*, and *a1b-Tubulin* remained unaltered when compared with controls; the expression of *Gap43,C-fos, Bdnf,* and *Shha* were significantly decreased in larvae exposed to PSNAP (100, 500, and 1000 nm); *Neurog1* and *Flavl3* expression was significantly downregulated in larvae exposed only to 100 and 500 nm PSNAP.<br>xi) Among the apoptosis-related genes (*Baxa, Bcl2a*, and *caspase 3a*) the expression of *caspase 3a* genes compared with controls, was significantly higher in larvae exposed to PSNAP (100, 500, and 1000 nm); the expression of *Baxa* level was upregulated in 100 and 500 nm PSNAP, and the expression of *Bcl2a* remained unaltered after PSNAP exposure.	

				xii) Compared with controls, there was a decrease in the GAD1 activity and GABA and 5-HT contents of larvae and no effect on the activities of AChE, tyrosine hydroxylase (THY), TPH and ACh and dopamine (DA) contents in larvae exposed only to PSNAP (100 nm). In 500 nm PSNAP fish THY activity and in 1000 nm group GABA and Ach contents were reduced significantly than the control larvae.	
Zebrafish	PS (80, 200, and 500 nm)	Embryos (normal and transgenic) and larvae	All three-sized PSNAP (0.1, 0.5, 1, 5, 10, 25, and 50 mg/L) were used and the embryos (8hpf) were exposed until 120 hpf; some of them were raised in PSNAP-free medium for 10 days (until 15 days) and used for behavioral assays (juveniles).	i) All three sized PSNAP crossed the chorion, absorbed by the yolk, and distributed into the intestinal tract, eye, brain, and dorsal trunk of zebrafish.<br>ii) PSNAP-80 was unable to induce malformation (pericardial edema, yolk sac edema, bent trunk, and malformed tail) and mortality of the embryos, while PSNAP-200 induced mortality and	Chen et al., (2024)

				malformation in a concentration-dependent manner.<br>iii) PSNAP (all three sized) decreased larval body length (96 hpf) in a concentration-dependent manner<br>iv) Spontaneous movements (24-48 hpf) of the embryos decreased in a concentration-dependent manner<br>v) A significant reduction in touch response was observed in 48 hpf embryos only by PSNPA 80 and PSNAP 200; no effect was observed in embryos exposed to PSNAP 500.<br>vi) Concentration-dependent decrease in heart rates were observed in embryos at 48 hpf exposed to all three-sized PSNAPs.<br>vii) Exposure to PSNAP 80 significantly decreased larval swimming distance in light and dark phases on both 5 and 10 dpf, while increased in larvae (5 and 10 dpf) in both	

				light and dark phases when exposed to PSNAP 200 and 500.<br>viii) During transition phase (light-dark changes) the movement of larvae increased in larvae exposed to PSNAP 80 and 200, while decreased in larvae exposed to PSNAP 500.<br>ix) Induced cellular death by all three-sized PSNAP in eye, brain, ventral trunks, and tail region in a time and concentration-dependent manner<br>x) PSNAPs increased neutrophil cell migration in mouth, eye, yolk, heart, and tail regions (24 hpf) in a concentration-dependent manner<br>xi) ROS was increased to 120 hpf larvae in all three sized PSNAP in a concentration-dependent manner.<br>xii) The neural genes *gfap* and *rab33a* was upregulated in PSNAP 80; *rab33a* and *tub1a* was downregulated in PSNAP 500.	

				The expressions of optical genes (*rho, opn1sw1* and *opn1* were upregulated in larvae exposed to PSNAP80 and downregulated in PSNAP 500, remained unaltered in PSNAP 200.	
Zebrafish	PS (23.03 ±0.266 nm)	Embryos	0.04 ng/l, 34 ng/L and 34 µg/L for 144 hpf	i) After 24 h of exposure, PSNAP aggregates/ agglomerates in the chorion, muscle, gills, and head of the fish<br>ii) In hatched larvae, PSNAP accumulation was found in digestive system, gills, and somite.<br>iii) No effect of PSNAP exposure was observed on survivability and hatching rates of the embryos.<br>iv) PSNAPs exposure have the potential to induce reduction in caudal fin twitching activity (neurotoxicity) in a concentration-dependent manner.<br>v) Heart rates reduced after 48 hpf in embryos exposed to PSNAP in a concentration-dependent manner.<br>vi) No significant morphological effects	Santos et al., (2024)

				(yolk sac edema, yolk sac and swim bladder areas, pericardial edema, liver area, and tail curvature) was observed in zebrafish embryos/larvae, exposed to PSNAP<br>vii) PSNAP induced significant morphometric alterations (decreased eye area with reduced interocular distance; reduction in minimum interocular distance; increased head area and reduction in head width and depth) in zebrafish larvae in a concentration-dependent manner.<br>viii) The angle between myosepta affected by PSNAP in a nonlinear fashion, while the distanced between the myosepta was found to be smaller compared with controls.<br>ix) PSNAP induced vasotoxicity in the yolk sac regions of the larvae (144 hpf) that impaired the formation of blood vessels.	

				x) Despite deposition of the PSNAP, no cytotoxicity was observed in the GI tract, however observed in the caudal vein of the larvae<br>xi) PSNAP induced ROS after 144 hpf exposure<br>xii) PSNAP reduced the average swimming speed of the larvae, however no effect on the anxious behavior of the larvae.	
Zebrafish	PS (50 nm, 1000 nm, and 50 µm)	larvae	Zebrafish larvae (120 hpf) were exposed to PSNAP (10 mg/L) for 24 h- 7 days	i) PSNAP was accumulated in the gut, skin, caudal fin and eyes and no mortality was observed in larvae<br>ii) The number of neutrophils and macrophages was increased in gut and caudal fin in the larvae exposed to PSNAP.<br>iii) ROS content (positive signals were mainly located in stomach and gut) was significantly increased, and downregulation of *cat* mRNA was observed in larvae exposed to PSNAP<br>iv) PSNAP enhanced the mortality of the larvae	Sendra et al., (2021)

				infected with *Aeromonas hydrophilia.*	
Zebrafish (*Danio rerio*)	PS (47 nm) coexposure with bisphenol A (BPA)	Adults (6 months old)	PSNAP (1 mg/L for 3 days); BPA (0.78 µg/L) alone and coexposed with PSNAP	i) PS accumulated in various tissues (viscera, gills, head, muscle)<br>ii) Inhibited AChE activity (not with coexposure)<br>iii) Upregulation of myelin/basic protein gene in the central nervous system<br>iv) Coexposure increased BPA uptake<br>v) Coexposure upregulated the expression of myeline and tubulin protein/gene expression, dopamine content, and the mRNA expression of mesencephalic astrocyte derived neurotrohic factor (MANF)	Chen et al., (2017b)
Zebrafish (*Danio rerio*)	PS (42 nm)	Adults	1 mg/g of fish (one week via food) and bred to produce F1 offspring	F0 fish:<br>i) The number of eggs produced by different fed groups (females exposed, not males; males exposed, not females, both males and females exposed, none of the parents exposed) and the percentage of fertilized eggs did not establish any	Pitt et al., (2018b)

				significant difference among different groups.<br>ii) GR activity was significantly lower in brain and muscle of exposed females, and muscle and testis of exposed males.<br>iii) GPx activity was elevated in the brain of exposed females, not in males<br>iv) CAT activity remained unaltered.<br>v) The oxygen consumption rate (OCR) in heart and gonad tissues did not show any significant difference; however, in ovary, OCR is slightly higher than controls.<br>F1 Fish:<br>i) Embryo mortality and deformities were not significantly different in the F1 offspring generated from F0 parents.<br>ii) Bradycardia observed in F1 embryos when both parents and mothers exposed to PSNAP<br>iii) Uninflated swim bladder was observed in F1 larvae (144 hpf) when both	

				parents (male and female) were fed with PS-containing diets.<br>iv) Accumulation of PSNAP was observed in yolk sac, GI tract, liver, pancreas, and gall bladder. More accumulation was observed in the liver and GI tract of the larvae when both parents were fed with PSNAPS.<br>v) No significant effect on larval locomotor activity<br>vi) GR activities were reduced significantly in F1 larvae (96 hpf) when both parents were fed with PSNAP<br>vii) GPx and CAT remained unaltered.<br>viii) OCR did not alter in any of the embryos (24 hpf) compared to controls	
Zebrafish (adults)	PS (~70 nm)	Adults (6 months old; 0.30 ±0.022 g body weight)	Exposed to 0.5, 1.5 and 5 mg/L for 7 days (acute exposure), 30 days, and 7 weeks (chronic exposure)	i) Accumulated in gonads, intestine, liver, and brain tissues (observed after 30 days exposure)<br>ii) Induced disturbances of lipid and energy metabolism, as well as oxidative stress.	Sarasamma et al., (2020);

				iii) In muscle, ROS level increased, and ATP level decreased in a concentration-dependent manner (0.5-1.5 mg/L); however, no change was observed in creatine kinase level, as well as the *hif-1α* content<br>iv) In liver, ssDNA and VTG contents were increased, *tnfα* and MDA content remained unaltered, EROD activity and cortisol level remained unaltered, however, *cyp1a1, cyp11a1* and *cyp19a1* were elevated in a concentration-dependent manner.<br>v) In the brain, concentration-dependent decrease in AChE, dopamine, melatonin, GABA, serotonin, vasopressin, kisspeptin, and oxytocin contents were observed, however, acetylcholine level remained unaltered.<br>vi) Behavioral alteration in locomotor activity, aggressiveness, shoal	

				formation, and predator avoidance behavior in a concentration-dependent manner (0.5-1.5 mg/L after 7 days exposure)<br>vii) The circadian rhythm in locomotor activity was dysregulated (exposed to 5mg/L for 7 weeks)	
Zebrafish (adults)	PSNAP (46 nm)<br>PSMIP (5.8 µm)	Male and female adult zebrafish	0.08, 0.5, 0.7, 1, 1.2, 1.5 mg/L Triphenyl phosphate (TPhP) and PSNAP (2 mg/L) and PSMIP (2 mg/L) exposed either alone (TPhP) or coexposed with PSNAP and PSMIP for 21 days	i) The 96 h LC_{50} for TPhP was 976 µg/L; presence of PSNAP or PSMIP (2 mg/L) did not have obvious effect on acute toxicity<br>ii) PSNAP and PSMIP alone had no effect on the HSI of the fish; however, TPhP alone increased HSI. Coexposure with PSMIP had no effect, while PSNAP aggravated the effects by further increasing HSI.<br>iii) PSNAP and PSMIP alone had no effect on the GSI of the fish, while TPhP decreased GSI in males and increased in females<br>iv) Coexposure of TPhP with PSMIP was unable to deregulate the effects of TPhP on GSI<br>v) Coexposure of TPhP with PSNAP significantly increased GSI in both male and female fish	He et al., (2021).

				vi) PSMIP alone did not alter the gonadal histology of both male and female fish, while PSNAP alone slightly decreased the amount of mature sperm in the testis and no effect on ovary.<br>vii) TPhP alone inhibited spermatogenesis by enhancing the amount of immature spermatocytes (spermatogonium and spermatocytes) and reducing the amount of mature spermatocytes (spermatids and spermatozoa).<br>viii) Coexposure with either PSMIP or PSNAP, the amount of mature spermatogenetic cells decreased further, and lacunae and interstitial tissue was observed in seminiferous tubules.<br>ix) PSNAP or PSMIP alone did not induce any alterations in ovarian histology.<br>x) TPhP inhibited ovarian development by inhibiting the maturation processes	

				of the oocytes having more perinuclear and cortical alveolar oocytes in the female fish exposed to TPhP alone.<br>xi) Coexposure with PSMIP, TPhP induced more mature follicles, mostly observed early vitellogenic than late vitellogenic oocytes<br>xii) Coexposure with PSNAP, more perinuclear and cortical alveolar oocytes were observed and some of the mature follicles were atretic.<br>xiii) Fish exposed to PSNAP, PSMIP, or TPhP alone did not affect the E2 and T contents of both male and female fish; however, combined exposure of PSNAP and TPhP enhanced E2 level in male fish but not in female fish. Moreover, T level enhanced in TPhP+ PSMIP exposed fish remained unaltered in TPhP+PSNAP group.<br>xiv) PSNAP, PSMIP, and TPhP alone has no effect	

				on the vitellogenin (VTG) content in male fish; however, coexposure of both PSNAP or PSMIP significantly increased the VTG concentration in male fish.<br>xv) In females, PSNAP or PSMIP alone had no effect on the VTG content, while TPhP alone significantly inhibited VTG content; coexposure with PSNAP and PSMIP, mitigated the effect of TPhP on VTG content in zebrafish.<br>xvi) Significant inhibition in the fecundity (total eggs produced) of fish exposed to PSNAP and TPhP alone only (not in PSMIP exposed fish). However, coexposure with PSNAP and PSMIP further reduced fecundity in fish.<br>xvii) No effect was observed in the spawning events, fertilization rates and the hatching rates of the embryos exposed to PSNAP and PSMIP alone, while TPhP alone or in	

				combination with PSNAP, or PSMIP reduced spawning events, fertilization, and hatching rates of the embryos.	
Zebrafish adults	PS (70 nm)	Adults were exposed, however, the F1 embryos were evaluated without any further exposure	Exposed to PSNAP (100µg/L), Microcystin LR (MCLR) (0.9, 4.5, and 22.5 µg/L) either alone or in combination for 45 days. The F1 embryos were collected and evaluated without any further treatment	i) In F1 larvae accumulation of PSNAP was observed due to parental exposure; although the accumulation of PSNAP did not affect MCLR concentrations, a concentration-dependent increase in MCLR content was observed in F1 embryos.<br>ii) Compared with controls, no significant effect was observed on hatching rates (72 hpf), hatching enzyme activities and spontaneous tail movements (wagging) of the F1 embryos exposed to PSNAP parentally; however, a concentration-dependent reduction in hatching rates, hatching enzyme activities and tail wagging of the F1 embryos exposed to MCLR alone or in combination with PSNAP.	Wu et al., (2021)

				iii) Pathological alterations in somite muscles (irregular somite boundaries) were observed in F1 larvae exposed parentally to MCLR alone or coexposed with PSNAP<br>iv) Compared with controls, no significant effect was observed on the AChE activity of the F1 embryos exposed to PSNAP or MCLR alone, parentally; however, a concentration-dependent increase in AChE activity was observed in F1 larvae coexposed to MCLR and PSNAP<br>v) Gene expression analysis related to hatching enzymes (*tox 16, foxp1, ctslb, xpb1, klf4, cap1, bmp4,cd63,He1.2,zhe1,an d prl*), cholinergic system (*ache* and *chrnα7*) and muscle development (*Wnt, MyoD, Myf5, Myogenin, and MRF4*) indicated alterations in the F1 larvae exposed parentally to PSNAP and	

				MCLR either alone or in combinations.	
Zebrafish (adults)	PS<br>PSMIP (8μm)<br>PSNAP (80 nm)	Adults	Adults were exposed to 10 μg/L and 1 mg/L PSMIP and PSNAP for 21 days.	i) Both PSMIP and PSNAP induced gut dysbiosis in adult zebrafish<br>ii) At the phylum level, both PSMIP and PSNAP at 1 mg/L concentration, increased the abundance of proteobacteria while the abundance of Fusobacteria, Firmicutes and Verrucomicrobiota decreased significantly<br>iii) The abundance of Actinobacteria decreased by PSMIP exposure while increased by PSNAP exposure.<br>iv) At the genus level, Aeromonas significantly increased in both PSMIP and PSNIP exposures<br>v) Only PSNAP, not the PSMIP, upregulated the mRNA levels of *il8, il10, il1β*, and *tnf α*; no effect was observed on mRNA levels of *il6* and *ifnphi 1*.	Xie et al., (2021)
Zebrafish (adults)	PS (54.5 ±2.8 nm)	Adults (both male and female 90 days old	Adults (90 days old) exposed to PSNAP (10 mg/L) and tris (1,3-dichloro-2-	i) Parents (F0) exposed to PSNAP and TDCIPP reduced survival rates, hatching rates, body	Zhao et al., (2021)

			propyl) phosphate (TDCIPP) (0.47, 2.64, or 12.78 µg/L) for 120 days (F0). Both F0 and F1 larvae (without exposure) were evaluated for thyroid endocrine disruptors.	length (7 dpf) and significantly enhanced the malformation rates during the embryo-larval development of F1 embryos compared with the embryos (F1) produced by the parents (F0) exposed to TDCIPP alone.<br>ii) PSNAP nonlinearly enhanced the accumulation of TDCIPP in the whole fish (body burden) as well as in the eggs (F0) and the order was gut> gills>gonad>liver. The accumulation in females tended to be higher than males.<br>iii) Compared with controls, the total T3 and T4 levels in F0 fish and F1 larvae did not altered significantly when exposed to PSNAP alone; however, fish exposed to TDCIPP alone or in combinations with PSNAP decreased the T3 and T4 levels in F0	

				females and T4 level in F0 males.<br>iv) In eggs T4 level reduced significantly when the fish were exposed to PSNAP alone and in combinations with TDCIPP (concentration-dependent). T3 levels in eggs were not altered in any treatment groups when compared with controls.<br>v) In F1 larvae, PSNAP exposure did not induce any significant change in T3 and T4 contents, while TDCIPP decreased T4 levels alone or in combination with PSNAP in a concentration-dependent manner. A concentration-dependent reduction in T3 level was observed when the fish was exposed in a combination of TDCIPP and PSNAP.<br>vi) In brain of female adult fish (F0), the transcription of corticotropin-releasing hormone (*crh*) was upregulated in a nonlinear	

				fashion in fish exposed to TDCIPP either alone or in combinations of PSNAP. However, the transcription of *tshβ* remained unaltered in all treatment groups when compared with controls.<br>vii) In the liver of female fish (F0), the expression of thyroglobulin (t*g*) and uridine diphosphate glucuronosyltransferase (*ugt1ab*) was upregulated in fish exposed to TDCIPP alone or in combination with PSNAP when compared with controls. Moreover, the expression of deiodinase 1 (*dio1*) and transthyretin (*ttr*) was downregulated, and the expression of deiodinase 2 (*dio2*) gene was upregulated in fish exposed to TDCIPP either alone or in combination with PSNAP in a nonlinear fashion when compared with control.<br>viii) In male F0 fish brain the transcription of *crh* and *tshβ* increased only in the	

				fish exposed to TDCIPP and PSNAP coexposure groups when compared with controls.<br>ix) In liver of male fish, the transcription of *tg* and *ugt1ab* genes was upregulated in fish exposed with TDCIPP alone or in combinations with PSNAP when compared with the controls in a nonlinear fashion. Moreover, the expression of *trβ* remained unaltered in all the experimental groups, while *trα* expression in the liver of males (F0) was upregulated in fish exposed to TDCIPP alone or in combinations with PSNAP in a nonlinear fashion when compared with controls. Also, a significant downregulation of the *ttr* expression was observed in male liver exposed to TDCIPP either alone or in combinations in a nonlinear fashion when compared with controls.	

				x) In F1 larvae, relative to control, the expression of *crh, tg*, *trα, tshβ* and *ugt1ab* was enhanced in coexposure groups in a concentration-dependent manner; moreover, the expression of *dio2* was upregulated by TDCIPP exposed larvae, and coexposure further enhanced the expression when compared with controls.<br>xi) The protein contents of TG was, compared with controls, enhanced in F1 larvae with the parental exposure to TDCIPP alone or in combination with PSNAP; however, the expression of TTR reduced significantly in F1 larvae with the parental exposure to TDCIPP alone or in combination with PSNAP.	
Zebrafish (adults)	PS (70 nm)	Adults were exposed and F1 larvae were evaluated	Exposed to PSNAP (100µg/L), Microcystin LR (MCL) (0.9, 4.5, and 22.5 µg/L) either alone or in	i) Due to parental exposure (F0) to PSNAP and PSNAP+ MCL, accumulation of PSNAP was observed in the testis and ovary of the F1 larvae	Zuo et al., (2021)

			combination with PSNAP (100 µg/L) for 21 days; the F1 larvae (120 hpf) were evaluated without further treatment.		and PSNAP increased the accumulation of MCL in F1 larvae<br>ii) Parental exposure of MCL and PSNAP+MCL affect the hatchability (decreased), malformation (decreased), mortality (increased), body length (decreased) and heart rates (decreased) of the F1 larvae. PSNAP exposure alone had no effect on the induction of developmental defects in F1 larvae.<br>iii) Parental exposure to PSNAP alone did not alter the T4 and T3 levels in the F1 larvae. However, MCL either alone or in coexposure reduced T4 and T3 levels of the F1 larvae<br>iv) The gene expression in the F1 larvae of HPT axis and GH/IGF axis remained unaltered when the parents were exposed to PSNAP alone; however, the expression of HPT axis genes (*trα, trβ, dio2, dio1, ttr, tg, tshr,*	

				nis, crh, pax8, and *nkx2.1*) except *ugt1ab* and *tpo,* were altered in F1 larvae after parental exposure either to MCLR alone or coexposed with PSNAP.<br>v) Among GH/IGF axis genes (*igf2α, igf1, gh, ghrh, ghrα, igf1ra, igf1rβ, igf2β,* and *igf2r*) only *igf1 igf2α* and *ghrβ* altered in F1 larvae when the parents were exposed to MCL+PSNAP.	
Zebrafish (adults)	PS (70 nm)	Adults (both males and females)	Exposed to PSNAP (100µg/L), Microcystin LR (MCL) (0.9, 4.5, and 22.5 µg/L) either alone or in combination for 3 months.	i) Accumulation of PSNAP in the liver is independent of the presence of MCLR in the media.<br>ii) Concentration-dependent increase in the accumulation of MCLR in the liver of fish was observed and presence of PSNAP enhanced the accumulation of MCL in a concentration-dependent manner.<br>iii) PSNAP alone has no effect on the histology of the liver, however, cellular swelling, fat vacuolation, and cytoarchitectural damage	Ling et al., (2022)

				was induced by MCL and PSNAP exacerbated these adverse effects.<br>iv) PSNAP alone has no effect on the ROS, MDA contents and the GST and CAT activities of the liver of the fish.<br>v) MCLR alone enhanced ROS and MDA contents of the liver in a concentration-dependent manner and the presence of PSNAP exacerbated the effects.<br>vi) The GST and CAT activities reduced in a concentration-dependent manner by MCLR and presence of PSNAP further reduced the enzyme activities<br>vii) The gene expression analysis related to antioxidant responses (*p38a, p38b, ERK2, ERK3, Nrf2, HO-1, cat1, sod1, gax, JINK1*, and *gstr1*) indicated that PSNAP was unable to produce any significant effect on the expression of these genes.	

				viii) MCLR alone enhanced the expression of *ERK2, ERK3, p38a, Nrf2, gpx1a, gstr1, cat1,* and *sod1* genes in a concentration-dependent manner.<br>ix) Coexposure with PSNAP further aggravated the expression of only *Nrf2* gene induced by MCLR.	
Zebrafish (adult)	PS (100 nm)	Adults	3 months old adult fish exposed to 25 mg/L PSNAP at 28-, 29-, and 30 ^{0} C for 96 h	i) The total distance, average speed, and average angular velocity taken by zebrafish in night phase (dark) decreased under PSNAP exposure, and the effect was modulated by temperature.<br>ii) Degenerative necrotic changes in the medulla oblongata, medial longitudinal fascicle, lateral valvula nucleus, and thalamus regions were observed in fish exposed to PSNAP with temperature effects<br>iii) Depending on the temperature, the protein, *Gfap*, which is an indicator of CNS injuries and *8-OHdG* (indicator of	Sulukan et al., (2022b)

				oxidative stress) was increased in the brain in fish exposed to PSNAP.<br>iv) Temperature and PSNAP exposure have a synergistic effect on metabolomic alterations.<br>v) PSNAP was accumulated in the brain.	
Zebrafish (Juveniles and adults)	PS (44 nm)	Juveniles and adults	1, 10, and 100 µg/L for 30 and 60 days.	A: 30 days exposure<br>i) Reduced body length (6%)<br>ii) Significant expansion of the villi structure of the intestinal tissue; increased mucus secretion and decreased LZM activity in a concentration-dependent manner<br>iii) Dysregulation of gene expression in intestine (downregulation of *tnfα*, *interferon*, *il1β, il10*, and *chemokine 8a* in fish exposed to 1 and 10 µg/L; however, upregulation of *tnf, il1b, il6, il10, cxcl8a,* inflammatory *caspase B*, and *tight junction protein 2a* by 100 µg/L group); the expression of *ahr* was downregulated in all	Teng et al., (2022b)

				groups of PSNAP exposure.<br>iv) In brain tissue, AChE significantly increased and LZM was decreased in a concentration-dependent manner in fish exposed to PSNAPs<br>v) Different concentrations of PSNAPs significantly disturbed the balance of the intestinal microbiome compared to the controls.<br>vi) Concentration-dependent changes in the brain metabolites, including 3,4- dihydroxyphenyl acetic acid, acetylcholine chloride, and l-glutamine.<br>B: 60 days exposure<br>i) Transgenerational transfer of PSNAP to F1 offspring from F0 parents exposed to PSNAPs for 60 days.<br>ii) In F1 the accumulation of particles PSNAPs were observed in liver, pancreas, and intestine.<br>iii) The spontaneous movements of the embryos, the heart beats, hatching rates, and the length of the F1 larvae	

				were affected by parental exposure of PSNAPs.	
Zebrafish (adults)	PS (100 nm) coexposed with lead	Adult	Adult fish were exposed to 20 and 200 µg/L PSNAP, 50 µg/L lead either alone or in combination for 3 weeks	i) PSNAP accumulated in the intestine in a concentration-dependent manner, and presence of lead in the medium can increase the accumulation of PSNAP in the intestine; however, higher concentration of PSNAP reduced the accumulation of lead in the intestine<br>ii) PSNAP with or without lead increased cilia defects and mucus secretion in the intestine in a concentration-dependent manner<br>iii) The MDA content in the intestine increased by PSNAP in a concentration-dependent manner; moreover, presence of lead in the medium enhanced the MDA content than those exposed to PSNAP alone.<br>iv) The 8-hydroxy-2´-deoxygluconate (8-OHdG) level was enhanced in the intestine	Yu et al., (2022a)

				by lead and presence of PSNAP in the medium, significantly increased 8-OHdG level induced by lead only fish.<br>v) TNF-α level was also increased by PSNAP in a concentration-dependent manner and presence of lead in the medium enhanced the TNF-α level than the exposed to PSNAP or lead alone.<br>vi) There are 7 types of cell populations were identified in intestine: enterocytes, macrophages, neutrophils, B cells, T cells, enteroendocrine cells, and goblet cells.<br>vii) In macrophages, immune system-related DEGs (*ctsba, nfkbiab*, and *pycard*) were significantly altered in PSNAP fish than PSNAP+ lead groups and the genes related to MAPK signaling pathways (*hsp70.1, hsp70.2*, and *hsp70l*) were altered in fish exposed only to lead	

				viii) In enterocytes, genes related to glutathione metabolism and cytochrome P450 (*gsta2, gsto 1, gsto2, gpx1a,* and *mgst1.2*) were significantly changed in fish exposed to lead and lead+PSNAP.<br>ix) In B and T cells, upregulation of *hsp70.1, hsp70.*2, and *hsp70.3* occurred in fish exposed to PSNAP, lead, and also in combinations,<br>x) Gene ontology (GO) analysis found several other DEG genes altered in macrophages after PSNAP exposure were *gadd45ba, jun, ccl35.2* and *ccl35.2*. and in PSNAP+lead groups were *ccr9a, cxcr4b*, and b*cl2l10*.; however, lead exposure altered *mt2* and *pycard*.<br>xi) In enterocytes, GO analysis showed alterations in the expression of *apoa4a, apoa1a*, and *apoea* in fish exposed to PSNAP, lead	

				either alone or in combinations. Moreover, expression of *npc2* and *prdx1* were altered in fish exposed to lead and lead +PSNAP	
Zebrafish (adults)	PSMIP (158-234 µm; 45-85 µm; 4-8µm)<br>PSNAP (394-407 nm; 40-54 nm)	Adults (Six months old)	Exposed to PSMIP (60-70 µg/L which is equivalent to 1770 items/L; 60-186 µg/L, which is equivalent to 1700-4900 items/L and 338 µg/L which is equivalent to 8902 items/L), PSNAP and oxytetracycline (100 µg/L) either alone or in combinations for 30 days.	i) No significant decrease in body length, body weight and BMI of the fish exposed to PSMIP, PSNAP, oxytetracycline (OTC) either alone or in combinations.<br>ii) No considerable damage was observed in thickness of intestinal layer in fish exposed to PSMIP (45-234 µm sizes), however, small sized PSMIP (4-8 µm), PSNAP, and OTC alone, or in combinations with micro or nanoplastics raptured and lysed the epithelium of intestinal villi and vacuolation of the intestinal epithelial cells.<br>iii) The gut microbial community were affected by OTC alone and combined exposure with PSMIP and PSNAP	Yu et al., (2022b)

Zebrafish (adults)	PS (20-80 nm) average size 57.5 nm	Adults	0.1, 1, 10 and 100 µg/L PS and 4-nonylphenol (1µg/L); exposed alone or in combination for 45 days	i) CAT activity in brain was decreased significantly in all treatment groups compared to controls.<br>ii) GSH content in the brain was also reduced in most treatment groups compared to controls.<br>iii) AChE activity in the brain is reduced by PSNAP alone in a concentration-dependent manner, though nonlinear. 4-nonylphenol alone was also significantly reduced brain AChE activity; however, coexposure did not inhibit brain AChE activity compared to controls.<br>iv) The activity of brain glutamine synthase (GS) was significantly decreased in fish exposed to different concentrations of PSNAP (nonlinear) and 4-nonylphenol; however, coexposure resulted higher GS activity than in fish exposed either alone to PSNAP or to 4-nonylphenol.<br>v) The glutamate dehydrogenase (GDH)	Aliakbarzadeh et al., (2023)

				activity enhanced by PSNAP exposure in a concentration-dependent manner; coexposure with 4-nonylphenol also potentiate (synergistic) the effect; Nonylphenol alone nonsignificantly enhanced GDH activity<br>vi) Both PSNAP and 4-nonylphenol and the combination of PSNAP+4-nonynphenol decreased αKGPD activity in the brain of fish compared with controls.<br>vii) Brain histology indicated that fish exposed to PSNAP showed damage in the neuronal layers as well as reduction in the neuronal cell number compared to controls, The fish exposed to 4-nonylphenol alone or coexposed with PSNAPs showed severe damage in neuronal cell layers as well as reduced the number of neurons.	
Zebrafish	PS (100 nm)	Adults	Exposed to 500 ng/mL PSNAP	i) No death was observed	Deng et al., (2023)

			waterborne for 28 days	ii) The CAT activity and GSH levels significantly decreased, and MDA content increased in liver of fish exposed to PSNAP; consequently, ROS production increased in liver<br>iii) There are nine types of cells isolated in zebrafish liver (hepatocytes male and female, endothelial cells, lymphocytes, cholangiocytes, epithelial cells, hepatic stellate cells, macrophages, and erythrocytes)<br>iv) 85% of the liver cells are hepatocytes male (52.39%) and hepatocytes female (33,63%)<br>v) The upregulated genes in hepatocytes male after PSNAP exposure were *ldlra, plin2, zbtb16a, foxo1a, angpt14, txnipa, klf6a, c7b,* *si: dkey-22f5.9*, and *hsd11b2* and downregulated genes were *h1fx, rpf26, BX908782.2, si:ch1973-110a20.7, cbln11, hamp,*	

				vtg1, sgk1, ldhba, and *ccl39.2*<br>vi) The upregulated genes in hepatocytes female after PSNAP exposure were *vtg6, crp2.1, crp2, igfbp1b, slc38a4, bzw1b, si: dkeyp-73d8.9, pck1, angptl4,* and *chac1* and downregulated genes *rpl26, cbla11, mycb, si:ch1073-110a20.7, mt2, CR318588.1, si:ch211-270n8.1, rnasel2, bhmt*, and *npm1a* genes<br>vii) In macrophages, the upregulated genes after PSNAP exposure were *ccl33.3*, *adh8a*, *fabp10a, fetub*, *si: dkey-7f3.14*, *apoa1b, si:ch211-222121.1, si: dkeyp-73d8.9, apoa2*, and *agxtb*; the downregulated genes were *lygl1, si:dkey-30j10.5, anxa3b, MFAP4, lgals2a,si:dkey-5n18.1, c1qb, gnr1, c1qc,* and *ccl34a.4*.<br>viii) In lymphocytes, PSNAP exposure upregulated *BX901920.1, CU914776.1, ins, NC-*	

				002333.4, FQ323156.1, hbba1.1, CR753876.1nfkbiaa, ccl20a.3 and *egr3* while *si: dkey21e2.12.1, vtg1, si: dkeyp-75b4.10, icn, BX908782.2,si:ch211-14a17.10, mmp13a.1, lect2l, lyz,* and *grn2* genes were downregulated<br>ix) In non-parenchymatic liver cells (stellate cells, cholangiocytes, endothelial cells, epithelial cells, and erythrocytes) the genes upregulated after PSNAP exposures were *ins, pik3r1, deptor, ulk2,* and *hmgb1a*, which may activate the hepatic stellate cells and promote liver fibrosis.<br>x) The PPAR signaling pathways was upregulated in hepatocytes from both male and female zebrafish, while female-derived zebrafish were more sensitive to estrogen stimulus and mitochondria and male derived hepatocytes	

				altered functions related to lipid metabolism after PSNAP exposure.<br>xi) Specific immune related pathways and oxidation-reduction processes were disrupted in macrophage and oxidation-reduction process, ATP synthesis and DNA binding were the most altered pathways in lymphocytes after PSNAP exposure	
Zebrafish (adults)	PS (~50 nm)	Adults (male); 4 months old	5, 10, 15 mg/L; zebrafish were exposed waterborne for 30 days and depurated for 16 days; evaluation of the fish were made on 3, 6, 12, 18, 24, 30, 34, 38, 42, and 46 days.	i) The bioaccumulation of PSNAP in zebrafish was concentration, tissue, and time-dependent<br>ii) The amounts of PSNAP accumulated in the different tissues exposed for 30 days were in the following order: intestine>liver>gill>muscle>brain.<br>iii) After 16 days depuration, brain of zebrafish contained significant amount of PSNAP	Habumugisha et al., (2023)
Zebrafish (adults)	PS (80 nm) [coexposure with vitamin D (vit D)]	Adults	15 and 150 mg/L PS; 280 and 2800 IU/kg body weight (vit D for 21 days	i) PSNPs accumulated in the liver of adult zebrafish, creating substantial number of vacuoles and	Li et al., (2023a)

				lipid droplets in the liver cell matrices<br>ii) Hepatosomatic index (HIS) was increased by PSNP in a concentration-dependent manner; while the fish fed with vit D did not show any significant difference with controls (No PSNPs).<br>iii) Exposure with vit D decreased the number of lipid droplets in the liver<br>iv) PSNP induced triglyceride and total cholesterol content which are reduced by high vit D diet.<br>v) Lipidomics analysis showed that PSNPs changed the lipid molecular contents related to cell membrane function and lipid biosynthesis; high vit D diet reduced the contents of lipid molecules related to lipid biosynthesis and thus alleviating cell membrane damage and lipid droplet accumulation.<br>vi) Nonlinear increase of the gene hydroxy-3-	

					methylglutaryl-coenzyme A (*hmgcra*), sterol regulatory element binding protein (*srebp1*), diaceylglycerol aceyltransferase 1b (*dgat1b*), acetyl coenzyme A carboxylase (*acc*) and carbohydrate response element binding protein (*cvhrebp*) by PSNPs in liver; however, the expression of carnitine palmitoyl transferase 1 (*cpt1)* decreased significantly by PSNAPs.	
Zebrafish	PS (70 nm)	Adult male and females (5 months old; length 3.30-3.56 cm; bodyweight 0.373-0.427 g)	Adult male and females were exposed to PSNAP (2mg/l) and diethylstilbesterol (DES) (1,10, 100 ng/L) either alone or in combination for 21 days.	i)<br>ii)<br><br><br><br><br>iii)	The 96 h LC_{50} of DES was 3.19 mg/L<br>PSNAP alone and coexposed with DES (concentration-dependent) can decrease HSI and GSI in both male and female fish<br>PSNPS and DES alone or in coexposure induced lacunae in the testis and increased the number of spermatogonium and spermatocytes in the testis; moreover, deformation of	Lin et al., (2023)

				seminiferous tubules were observed.<br>iv) PSNAP and DES exposures alone showed more preovulatory oocytes and smaller mature oocytes than controls<br>v) Both PSNAP and DES (concentration-dependent) alone and coexposure decreased the level of E2 and T in both male and female zebrafish.<br>vi) There was no effect of PSNAP alone on the E2/T ratio of male and female fish, however, DES alone or in combination with PSNAP increased the E2/T ratio in a concentration-dependent manner in male fish. In females, a concentration-dependent reduction was observed in the E2/T ratio of coexposed fish.<br>vii) The VTG content of male fish remained unaltered after PSNAP exposure, however, DES alone or coexposed with PSNAP enhanced VTG content in	

				a concentration-dependent manner in males; however, in females, NPS alone or in combination with DES reduced VTG content in a concentration-dependent manner.<br>viii) PSNAP exposure has no significant effects on the T3 and T4 levels of both male and female fish; however, DES alone or in combination with PSNAP decreased both T3 and T4 contents in male and female fish in a concentration-dependent manner.<br>ix) Compared to controls, PSNAP and DES alone or in combination reduced fecundity, spawning events, fertilization, and hatchability of the embryos.<br>x) PSNAP and DES either alone or in combination induced abnormal development (teratogenic effects) of the larvae observed at 96 hpf (spinal curvature, pericardial	

				cyst, and growth retardation).	
Zebrafish (adults)	PS (134±2.9 nm)	Adults (3 months old, AB strain)	Exposed to 25 mg/L PSNAP at 28-, 29-, and 30 °C for 96 h	i) Increase of temperature with PSNAP significantly induced DNA damage (8-OHdG staining) accompanied by degeneration, necrosis, and hyperemia in liver histology.<br>ii) In gills, adhesion of lamellae, desquamation and inflammation in lamellar epithelium occurred after increase of temperature with PSNAP was observed<br>iii) In muscles, oxidative stress was altered in fish exposed to increasing temperature with PSNAP.	Senol et al., (2023)
Zebrafish (adults)	PS (80 nm). Coexposed with high (2800 IU/kg) and low (280 IU/kg) vit D	Adults	Exposed to PSNAP (15 and 150 µg/L) either alone or in combination of vit D (280-2800 IU/kg, via food) for 21 days	i) High vit D (2800 IU/kg) reduced the accumulation of PSNAP in the brain by 20%.<br>ii) CSI slightly increased in fish exposed to PSNAP alone (not significant)<br>iii) Accumulation of PSNAP in the intestine was concentration-dependent and presence of vit D	Teng et al., (2023)

				reduced the accumulation of PSNAP in the intestine<br>iv) The blood-brain barrier basement membrane was damaged by PSNAP in a concentration-dependent manner, while the damage was less when coexposed with vit D.<br>v) PSNAP exposure induced anxiety-like behavior, while vit D alleviated the process. However, the average velocity and average acceleration were unaffected by the treatment<br>vi) Decreased serotonin (5-HT), GABA, and dopamine level in the brain; vit D coexposure increased 5-HT content in the brain compared with the fish exposed to PSNAP alone.<br>vii) PSNAP enhanced cortisol content while oxytocin level decreased.<br>viii) SOD activity in the brain was increased by PSNAP, while coexposure with vit D alleviated the process.	

				ix) PSNAP exposure induced vacuolization in intestinal goblet cells and mitochondria and disorder in the arrangement of intestinal villi, while coexposure with vit D alleviated the process.<br>x) The SOD activity in the intestine increased by PSNAP in a concentration-dependent manner; coexposure with vit D alleviated the process<br>xi) The MDA content increased in fish exposed only to 15µg/L PSNAP; vit D alleviated the process.<br>xii) The immunoglobulin content (IgM) of the intestine was enhanced by PSNAP in a concentration-dependent manner, while vit D (higher dose) alleviated the process.<br>xiii) Diamine oxidase (DAO) activity was inconsistently enhanced in fish exposed with vit D and PSNAP	

				xiv) In serum significant decrease of D-lactic acid was observed in fish exposed only to 15 µg/L PSNAP<br>xv) PSNAP exposure decreased the expression of *IL-6* and increased the expression of nuclear factor kappa-B (*nf-κb*) in the intestine, while vit D alleviated the process<br>xvi) The expression of *IL-1β* in the intestine was upregulated by PSNAP (15 µg/L) while downregulated by higher concentration (150 µg/L); vit D can reverse the process<br>xvii) The expression of tight junction protein 2a (*tjp2a*) and *tjp2b, cyp1a1* and *cyp1b1* increased significantly in intestine of fish exposed to PSNAP (15 µg/L) which was alleviated by vit D<br>xviii) PSNAP reduced the diversity and abundance of the gut virome	
Zebrafish	nanoplastics (100 nm)	Adults (120 dph)	Exposed to 1mg/L nanoplastics with or	i) In parental generation, the accumulation of BPAF	Wang et al., (2023e)

			without BPAF for 45 days		was observed in intestine and other tissues and nanoplastics enhanced BPAF accumulation in intestine and other organs.<br>ii) In offspring, BPAF accumulation was observed in larvae of both BPAF and BPAF+nanoplastics exposed parents.<br>iii) BPAF alone or coexposed with PS decreased locomotor behavior (average speed and total distance travelled) of the parental fish. However, nanoplastics alone has no significant effect on locomotion.<br>iv) BPAF and coexposure with nanoplastics decreased fecundity, however no effect (slight decrease which is nonsignificant) in fish exposed to PS alone<br>v) The hatching rates of the embryos was reduced when parents were exposed either to BPAF alone or in combination with nanoplastics. No	

				effect on mortality index (embryo-larval) was observed.<br>vi) The body length of the larvae reduced when parents were exposed either to BPAF alone or in combination of BPAF+nanoplastics.<br>vii) The locomotor behavior (total distance travelled) of the offspring (6 dpf larvae) significantly reduced when parents were exposed either to BPAF alone or in combinations with nanoplastics.<br>viii) BPAF and BPAF+ nanoplastics exposure increased the expression of inflammatory genes (IL-10, IL-8, TGF β1 and TNFα) and the apoptotic genes (bax, bcl-2, caspase-3 and caspase-9).<br>ix) Parental nanoplastics exposure alone increased the expression of neurodevelopmental genes (*GFAP*), inflammatory genes (*TGF-β1, TNF-α*) and	

				apoptotic genes (*bcl-2*) in offspring<br>x) The oxidative stress-related genes in offspring *cat, Cu/Zn-Sod* and *Keap1* were significantly increased in the BPAF group and BPAF+ nanoplastics group.<br>xi) Nanoplastics alone was able to induce *cat* gene expression in zebrafish offspring.	
Zebrafish (adults)	PS (20 µm and 100 nm)	Adults	Exposed to 100 and 1000 µg/L PSMIP and PSNAP (fluorescently labelled) for 4 days and then transferred to clean water for 3 days	i) Did not cause any impacts on survival and observable quantitative health of the fish.<br>ii) Mainly accumulated in the gut heterogeneously<br>iii) Increased mucus secretion in the gut<br>iv) Excreted through fecal material within 2-3 days of depuration in a concentration-dependent manner.<br>v) PSMIP accumulated in the fore and mid-gut regions, while PSNAP accumulated throughout the gut.	Yang et al., (2023)
Zebrafish (adults)	PS (100 nm)	Adults	Exposed to PSNAP (1 mg/L), arsenic	i) Presence of PSNAP in the media enhanced the	Zhang et al., (2023)

			(As; 200 µg/L) either alone or coexposure for 30 days	accumulation of As in the brain<br>ii) Compared with controls, the level of ROS significantly increased in the brain of zebrafish exposed to PSNAP and As either alone or in coexposed conditions.<br>iii) The SOD activity significantly increased and the GSH content significantly decreased in the brain of coexposed fish (As +PSNAP) compared with controls.<br>iv) The MDA content in the brain of zebrafish, compared with controls, significantly increased in fish exposed to As alone or in combination with PSNAP.<br>v) Compared with controls, a small amount of micro thrombosis consisting of aggregated and dissolved red blood cells, and the mitochondria with damaged membrane and loss of cristae observed in the brain of the fish exposed to PSNAP and	

				As either alone or in combinations.<br>vi) The mitochondrial DNA copy number significantly reduced in fish exposed to PSNAP, As and also in combinations when compared with the controls.<br>vii) Genes related to mitochondrial synthesis (*pgc1-a* and *pgc1-b*) in brain of zebrafish significantly downregulated in fish exposed to As alone and in combination with As+PSNAP; however, no significant effect was observed in fish exposed to PSNAP alone.<br>viii) Compared with controls, the mitochondrial fusion-related genes (*mfn1a, mf1b*, and *opa1*) were downregulated in the brain of fish exposed to PSNAP, As, and in combinations.<br>ix) The expression of mitochondrial division-related genes (*drp1, mff, fis 1, mid49* and *mid51*)	

				were tended to be upregulated by PSNAP, As and in combinations when compared with controls.<br>x) The expression of genes related to mitophagy (*ulk1a*, and *parl*) were upregulated by PSNAP, and As exposure either alone or in combinations when compared with the controls. Moreover, other mitophagy genes (*parkin, pink 1* and *fundc1*) were upregulated in combined exposure groups when compared with controls. Also, the expression of *parkin* was also upregulated in fish exposed to As alone.<br>xi) Compared with controls, the neurotransmitter dopamine (DA) significantly decreased, and acetylcholine (ACh) increased in the brain of fish exposed only to As in combination with PSNAP. Other treatment groups (PSNAP, and As) did not show any significant	

				alterations when compared with controls.<br>xii) The neurotransmitter synthase gene (*th*) significantly downregulated and *chat* gene significantly upregulated in the brain of fish exposed to As+ PSNAP groups when compared with controls. The other two groups (PSNAP and As) did not induce any significant change when compared with controls.<br>xiii) The neurotransmitter catabolic gene *mao* was significantly downregulated in the brain of fish exposed to PSNAP, As, either alone or in combination when compared with the controls.<br>xiv) The activity of MAO was significantly decreased and the activity of AChE significantly increased in the brain of fish exposed to As in combination with PSNAP when compared with controls, while in	

				other two groups (PSNAP and As), MAO tended to decrease, while AChE remained unaltered.<br>xv) The expression of AChE mRNA in the brain of zebrafish was upregulated only in fish when As was present in the medium (fish exposed to As, and As+ PSNAP groups). PSNAP alone did not induce any significant alteration while tended to increase.	
Zebrafish (adult)	PS (100 nm)	Adults	Exposed to 1 mg/L PSNAP and coexposed with 1 mg/L As 30 days	i) Compared with controls, there was no significant difference in the mortality of the fish exposed to PSNAP, As, and PSNAP+As groups.<br>ii) The swimming speed was significantly decreased in fish exposed to PSNAP and As alone or in combinations compared with controls.<br>iii) The anxiety-like behavior (evaluated by open field test) showed the coexposure group and those exposed to PSNAP alone spent more time in the lower layer than the upper layer, while in controls and As groups spent uniform time	Zhang et al., (2024c)

				in both upper and lower layers.<br>iv) The learning memory ability (evaluated by T-maze test), showed control and PSNAP groups swam quickly in the feeding zone (F zone) and stayed there for long time, while the fish exposed to As and in combinations, stayed both in the F zone and stimulating zone (S zone)<br>v) Compared with controls, 5-hydroxytryptamine (5-HT) level in the brain significantly reduced in fish exposed to PSNAP and As; moreover, coexposure further promoted the reduction.<br>vi) The 5-HT levels in the serum remained unaltered in fish exposed to PSNAP and As and significantly reduced in coexposure groups when compared with controls.<br>vii) In intestines, 5-HT level tended to decrease in fish exposed to PSNAP and As alone or in coexposed fish.<br>viii) The activity of MAO (the catalytic enzyme of 5-HT) and the mRNA level of *mao* in the intestine tended to	

				decrease in fish exposed to PSNAP and As either alone or in combination when compared with controls	
Zebrafish (adult)	PS (50±3 nm)	Adults (4 months; 3.5±0.4 cm body length) (males and females)	Exposed to 1 mg/L PSNAP for 4 weeks	i) Compared with controls, PSNAP exposed fish (both males and females), took significantly longer time for their first entry to reach the food pellets and spend markedly less time in the reward zone in the T-maze task, indicating the occurrence of learning and memory deficits.<br>ii) Compared with controls, the β-galactosidase and lipofuscin levels (aging markers) are significantly higher in brain of zebrafish (both males and females) exposed to PSNAP<br>iii) Compared with controls, the brain of the zebrafish (both males and females) exposed to PSNAP accumulated higher levels of ROS and significantly lower levels of total antioxidant activity with higher levels of H_2O_2 and O_2^- in their brain.	Zhou et al., (2023d)

				iv) Compared with controls, the γ-H2AX levels, 8-hydroxydeoxyguanosine (8-OHdG), and MDA contents were significantly higher in the brain of male and female fish exposed to PSNAP.<br>v) Compared with controls, the ATP and cyclin-dependent kinase levels were significantly lower and p53 levels were significantly higher in the brains of male and female zebrafish exposed to PSNAP.	
Zebrafish	PS (0.05-0.1 µm). also fed with high fat diet	Juvenile	Exposed to 1000 µg/L and high fat diet (24% crude fat)	i) Three weeks fed high fat diet increased the body weight; however, one week exposed to PSNAP had no effect on body weight<br>ii) NP perturb the activities of CAT (increased), and MDA (decreased), while SOD remained unaltered in liver<br>iii) PSNAP perturb lipid metabolism and gut microbiota stability.<br>iv) PSNAP exposure downregulated the	Du et al., (2024)

				expression of *fasn* mRNA and upregulated the expression of *cpt1ab*; the expression of *hmgcra* remained unaltered by PSNAP, however, fed with high fat diet and PSNAP upregulated the expression<br>v) The combined effects of PSNAP and high fat diet resulted gastrointestinal injury (number of goblet cells reduced).	
Zebrafish	PS (50 nm); coexposed with homosolate (0.0262-262 µg/L)	Adults	Exposed to PS (1.0 mg/L) + homosolate (0.0262-262 µg/L) for 21 days	i) PSNAP enhanced (not significant) the accumulation of homosolate in the testis, ovary, liver, and brain of male and female fish<br>ii) GSI in both male and females remained unaltered in fish exposed to PSNAP alone or in combination with homosolate<br>iii) PSNAP alone was unable to alter the amount (percentage) of PO, LVO, CAO, and EVO in the ovary; however, coexposure with homosolate decreased the	Ye et al., (2024)

				number of PO and increased the number of LVO and CAO and EVO remained unaltered.<br>iv) PNS alone has no significant effect on the amount of spermatogonium, spermatocytes and spermatids and spermatozoa (percent); however, coexposure with homosolate showed testicular damage (lacunae in the seminiferous tubules) with decreased amount of spermatozoa and no effect on spermatogonia, spermatocytes, or spermatids.<br>v) Egg production and hatching rates remained unaffected by PSNAP alone; however, hatching rates reduced in coexposure with homosolate in a concentration-dependent manner<br>vi) PSNAP alone has no significant effect on F1 embryo mortality;	

				however, coexposure with homosolate enhanced F1 embryo mortality<br>vii) No significant effect of PSNAP alone in the malformation of F1 larvae (spinal curvature, swim bladder deformities, mandibular malformation, body edema, yolk sac edema, pericardial edema, tail deformity); however, coexposure with homosolate enhanced the malformation rates of the F1 embryos.<br>viii) No effect of PSNAP was observed in the expression of *sgk1* and *stc* mRNAs in the ovary of adult zebrafish; however, coexposure with homosolate enhanced the expression of both *sgk1* and *stc* mRNAs in the ovary<br>ix) No effect was observed in the the E2 level in the ovary and serum of the fish exposed to PSNAP alone, however, co exposure with homosolate enhanced the E2 content	

				in the ovary as well as in the serum<br>x) T content in the ovary did not alter in zebrafish after exposure with PSNAP alone or in combination with homosolate<br>xi) PSNAP alone was unable to alter the GnRH and FSH levels in the ovary; however, PSNAP attenuated the effects induced by homosolate alone (increased GnRH and FSH) in the ovary of zebrafish<br>xii) PSNAP did not exhibit any effect in the LH content in the ovary when exposed alone, however, coexposure with homosolate enhanced the LH content in the ovary<br>xiii) In males serum E2 and testis E2 levels and GnRH and FSH contents remained unalter in fish exposed to PSNAP alone, however, PSNAP attenuated the effects induced by homosolate alone (increased serum T and testis T, GnRH and	

				FSH in testis) in the zebrafish<br>xiv) The LH levels in testis were significantly reduced by PSNAP alone exposure and coexposure with homosolate aggravated the effect.<br>xv) The expression of *cyp17a2* and *hsdβ1* mRNAs in the ovary remained unaffected in fish exposed to PSNAP alone; however, coexposure with homosolate enhanced the expression.<br>xvi) In testis, homosolate-induced enhancement in the levels of *hsdβ1, cyp19a1*, and *cyp11a2* mRNAs were attenuated by PSNAP during coexposure<br>xvii) In female liver, PSNAP has no effect on the expression of *esr2b, vtg1*, or *vtg2* mRNAs, but coexposure with homosolate upregulated the expression of these mRNAs in a	

				concentration-dependent manner<br>xviii) In male liver, PSNAP alone has no effect on the expression of *esr2b* or *vtg2* mRNAs, however, coexposure with homosolate upregulated the expression of these mRNAs in the liver of male zebrafish.	
Zebrafish (adult)	PS (100 nm)	Adults	Exposed to PSNAP (1 mg/L) and As (1 mg/L) either alone or in combinations for 30 days.	i) Compared with controls, there was no significant difference in the mortality of the fish exposed to PSNAP, As, and PSNAP+As groups.<br>ii) The swimming speed was significantly decreased in fish exposed to PSNAP and As alone or in combinations compared with controls.<br>iii) The anxiety-like behavior (evaluated by open field test) showed the coexposure group and those exposed to PSNAP alone spent more time in the lower layer than the upper layer, while in controls and As groups spent uniform time in	Zhang et al., (2023)

				both upper and lower layers.<br>iv) The learning memory ability (evaluated by T-maze test), showed control and PSNAP groups swam quickly in the feeding zone (F zone) and stayed there for long time, while the fish exposed to As and in combinations, stayed both in the F zone and stimulating zone (S zone)<br>v) Compared with controls, 5-hydroxytryptamine (5-HT) level in the brain significantly reduced in fish exposed to PSNAP and As; moreover, coexposure further promoted the reduction.<br>vi) The 5-HT levels in the serum remained unaltered in fish exposed to PSNAP and As and significantly reduced in coexposure groups when compared with controls.<br>vii) In intestines, 5-HT level tended to decrease in fish exposed to PSNAP and	

				As alone or in coexposed fish.<br>viii) The activity of MAO (the catalytic enzyme of 5-HT) and the mRNA level of MAO in the intestine tended to decrease in fish exposed to PSNAP and As either alone or in combination when compared with controls.<br>ix) The mRNAs (*tph1a, tph1b*, and *tph2*) tryptophan hydroxylase (TPH), the rate-limiting enzyme for 5-HT synthesis, showed that *tp1a, tp1b* and *tph2* tended to be downregulated in fish exposed to PSNAP and As either alone or in combinations.<br>x) Among the 5-HT receptor mRNAs, *htr1aa, htr1ab*, and *htr2*c were significantly upregulated in the brain of fish exposed to PSNAP, As, either alone or in coexposure; while the expression of *htr1b* and *htr4* showed	

				downregulation in fish exposed to PSNAP and As either alone or in coexposure when compared with controls.<br>xi) The level of ROS in the intestine markedly increased and GSH content significantly decreased in fish exposed to PSNAP and As either alone or in combinations when compared with controls.<br>xii) The SOD activity and MDA content in PSNAP and As-exposed fish remained unaltered in the intestine, while significantly increased in fish exposed to PSNAP+As when compared with controls.<br>xiii) The mitochondrial DNA copy number significantly reduced in fish exposed to PSNAP, As either alone or in combinations when compared with controls.<br>xiv) The intestinal microbiota was also altered after PSNAP and As exposure either alone or in	

					combination, when compared with controls. The intestinal histophysiology indicated that the intestinal villi were swollen in the fish exposed to PSNAP or As either alone or in combinations when compared with the controls even though the height of the intestinal villi significantly decreased in all treatment groups. Moreover, the ratio of the villus height/crypt depth or the ratio of the villus height/villus width were also significantly decreased in all the treatment groups when compared with controls.	
Zebrafish	Polyvinyl chloride (PVC) (200 nm) alone and coexposed with B(a)P (10 µg/L)	Embryos (6hpf)	Exposed to $3X10^{10}$ particle/L of PVC alone or coexposed with B(a)P (10 µg/L) for 24 h	i)<br>ii)	No mortality or hatching delay of the embryos<br>Length of the larvae decreased by PVC coexposed with B(a)P.	Monikh et al., (2022)

Supplementary Figure 1

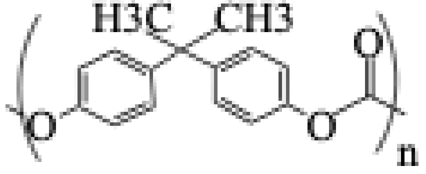

Polycarbonate

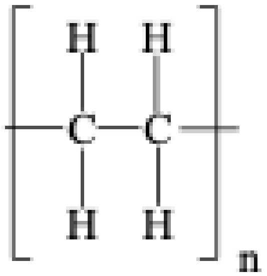

Polyethylene

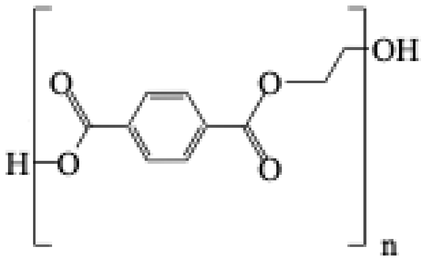

Polyethylene terephthalate

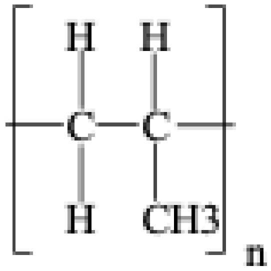

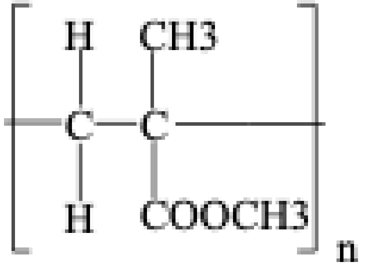

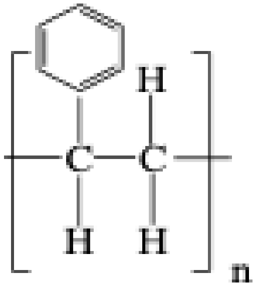

Polymethyl methacrylate

Polypropylene

Polystyrene

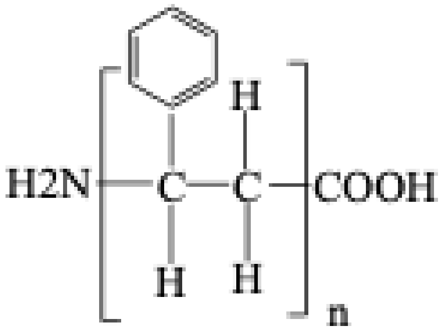

Polystyrene

(NH2 and COOH form)

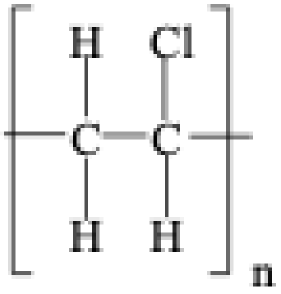

Polyvinyl Chloride
